# Diversity and Variability of NOD-Like Receptors in Fungi

**DOI:** 10.1093/gbe/evu251

**Published:** 2014-12-08

**Authors:** Witold Dyrka, Marina Lamacchia, Pascal Durrens, Bostjan Kobe, Asen Daskalov, Matthieu Paoletti, David J. Sherman, Sven J. Saupe

**Affiliations:** ^1^INRIA-Université Bordeaux-CNRS, MAGNOME, Talence, France; ^2^Institut de Biochimie et de Génétique Cellulaire, UMR 5095, CNRS-Université de Bordeaux, France; ^3^School of Chemistry and Molecular Biosciences, Institute for Molecular Bioscience and Centre for Infectious Disease Research, University of Queensland, Brisbane, Queensland, Australia

**Keywords:** NLR, fungi, NACHT, immunity

## Abstract

Nucleotide-binding oligomerization domain (NOD)-like receptors (NLRs) are intracellular receptors that control innate immunity and other biotic interactions in animals and plants. NLRs have been characterized in plant and animal lineages, but in fungi, this gene family has not been systematically described. There is however previous indications of the involvement of NLR-like genes in nonself recognition and programmed cell death in fungi. We have analyzed 198 fungal genomes for the presence of NLRs and have annotated a total of 5,616 NLR candidates. We describe their phylogenetic distribution, domain organization, and evolution. Fungal NLRs are characterized by a great diversity of domain organizations, suggesting frequently occurring combinatorial assortments of different effector, NOD and repeat domains. The repeat domains are of the WD, ANK, and TPR type; no LRR motifs were found. As previously documented for WD-repeat domains of fungal NLRs, TPR, and ANK repeats evolve under positive selection and show highly conserved repeats and repeat length polymorphism, suggesting the possibility of concerted evolution of these repeats. We identify novel effector domains not previously found associated with NLRs, whereas others are related to effector domains of plant or animals NLRs. In particular, we show that the HET domain found in fungal NLRs may be related to Toll/interleukin-1 receptor domains found in animal and plant immune receptors. This description of fungal NLR repertoires reveals both similarities and differences with plant and animals NLR collections, highlights the importance of domain reassortment and repeat evolution and provides a novel entry point to explore the evolution of NLRs in eukaryotes.

## Introduction

Detection and adequate response to nonself is essential for survival and development in all multicellular organisms. An important part of the innate immune detection in plants and animal lineages is ensured by a class of signal transducing proteins known as NB-LRR proteins in plants and nucleotide-binding oligomerization domain (NOD)-like receptors (NLRs) in animals (Maekawa et al. 2011).

Plant NB-LRR proteins sense the presence of fungal, oomycete, nematode, bacterial, or viral pathogens and trigger an immune response in the form of a localized cell death reaction termed the hypersensitive response (Jones and Dangl 2006; Jacob et al. 2013). NB-LRR proteins represent the resistance proteins involved in effector-triggered immunity as they sense strain-specific pathogen effectors or the modification of self, induced by these effectors. Plant genomes encode large repertoires of NB-LRR proteins with up to several hundred members. NB-LRR genes are typically highly polymorphic between individuals and subject to positive diversifying selection resulting from the host-pathogen arms race.

Animal NLRs, in turn, are activated by relatively invariant MAMPs (microbe-associated molecular patterns) and at least in mammals, the number of NLRs is more limited than in plant genomes (Kanneganti et al. 2007; Saleh 2011). Animal NLRs and plant NB-LRR receptors are collectively designated NLRs and are members of the family of STAND proteins (signal-transducing ATPase with numerous domains), (Leipe et al. 2004; Danot et al. 2009). These proteins typically comprise a central nucleotide binding and oligomerization domain (NOD) linked to an N-terminal effector domain and a C-terminal domain composed of superstructure-forming repeats such as LRR, WD, HEAT, ANK, or TPR motifs. One can distinguish two main classes of NOD domains: The NACHT (named after the NAIP, CIITA, HET-E and TP-1 proteins) and the NB-ARC domain. In general, plant NB-LRR proteins display an NB-ARC NOD domain whereas animal NLRs display a NACHT domain, although many instances of NB-ARC STAND proteins are described also in animal lineages. In most cases, the C-terminal domain of plant and animal NLRs corresponds to a LRR domain, but other types of repeat domains have been reported for instance in fish and marine invertebrates such as *Hydra* and the coral *Acropora digitifera* (Stein et al. 2007; Lange et al. 2011; Hamada et al. 2013). The N-terminal effector domains are variable and either correspond to coiled-coil or Toll/interleukin-1 receptor (TIR) domains in plants (Jacob et al. 2013), whereas CARD, BIR, PYD, death domain (DD), and DED are found in animals (Messier-Solek et al. 2010). In addition to these domains, a variety of other N-terminal domains, sometimes restricted to a given phylum, has been reported (Hamada et al. 2013; Yuen et al. 2014). In spite of the remarkable overall resemblance between these immune receptors in plant and animal lineages, it is unclear if this similarity is the result of evolutionary conservation (Ausubel 2005; Maekawa et al. 2011; Yue et al. 2012). It has been proposed that build-up of NLRs is the result of convergent evolution by association of a limited set of preexisting domains such as NOD and LRR domains. Remarkably, NLRs appear not only to be involved in the immune response to pathogenic nonself, but an emerging trend reveals that these receptors may also control other forms of biotic interactions, for instance between animal hosts and their symbiotic microbiome (Chu and Mazmanian 2013).

With an estimated 5.1 million species, the fungal kingdom represents a major eukaryotic lineage and a sister group of the holozoa (Blackwell 2011; He et al. 2014). Because of their overall organization, most cells in fungal organisms are in direct contact with their biotic environment. In addition to a variety of pathogenic and symbiotic interactions, fungi are also exposed to diverse adverse biotic interactions as hosts of a variety of pathogens and parasites such as mycoviruses, mycophagic bacteria, mycoparasitic fungi, and grazing nematodes (Leveau and Preston 2008; Pearson et al. 2009; Bleuler-Martinez et al. 2011; Druzhinina et al. 2011; Sabotic et al. 2012). In the recent years, the awareness for the existence and importance of fungal nonself recognition and defense systems is gradually increasing. Based on the common central role for STAND proteins as intracellular innate immune receptors in plant and animals, it is not unreasonable to suppose that STAND proteins may play similar roles in fungi. And indeed, there is evidence for the involvement of STAND proteins in the detection of nonself and the control of programmed cell death in fungi, thus stressing the analogy between animal and plant NLRs. The HET-E protein of *Podospora anserina*, one of the founding members defining the NACHT domain, is involved in a fungal nonself recognition and programmed cell death process termed heterokaryon incompatibility (Saupe et al. 1995; Koonin and Aravind 2000). Incompatibility is triggered when genetically distinct individuals belonging to the same fungal species undergo cell fusion and corresponds to a pleiotropic cellular response culminating in the programmed cell death of the fusion cell (Pinan-Lucarre et al. 2007; Bidard et al. 2013). HET-E has a tripartite domain organization typical of STAND proteins, with a central NACHT domain, a C-terminal WD40 repeat domain and an N-terminal HET domain. The HET domain is found in different proteins involved in fungal incompatibility and corresponds to a death effector domain (Smith et al. 2000; Paoletti and Clave 2007). HET-E is part of a larger gene family comprising ten members, termed NWD genes. Five of these proteins also comprise an N-terminal HET domain and two of those correspond to genetically identified incompatibility genes (HET-D and HET-R) (Paoletti et al. 2001, 2007; Chevanne et al. 2009). The five other members display different N-terminal domains. The WD repeat regions of the members of the gene family are hypervariable. The repeats show a high level of internal repeat conservation, and are undergoing concerted evolution, meaning that repeat shuffling and exchanges occur both within and between members of the gene family (Paoletti et al. 2007; Chevanne et al. 2010). In addition, the repeat region is subjected to positive diversifying selection operating specifically on four amino acid positions of each individual repeat, which map to the protein–protein interaction surface of the WD-repeat β-propeller structure. Another member of the gene family, termed NWD2, shows an N-terminal domain homologous to the prion-forming domain of the HET-s prion protein of *P. anserina*. It is proposed that NWD2 acts as an activator of the HET-S pore forming toxin by triggering transconformation of its prion-forming domain and subsequent activity of the HeLo toxicity domain (Greenwald et al. 2010; Daskalov et al. 2012; Saupe and Daskalov 2012; Seuring et al. 2012). This mode of signal transduction between a STAND protein and an trans-acting effector domain was proposed to be widespread in fungi and in addition to the [Het-s] prion-forming motif, two additional prion-like motifs (termed σ and PP) have been described (Daskalov et al. 2012). These motifs were found as N-terminal domains of STAND proteins of various types such as NACHT-WD, NACHT-ANK, or NB-ARC-TPR proteins. It was recently shown that the [Het-s] prion domain, and the N-terminal prion motif of NWD2 can functionally replace the PYD region in NLRP3-mediated CARD activation (Cai et al. 2014). Involvement of STAND proteins in incompatibility is not restricted to *P. anserina*, as the *vic 2* and *vic 4* loci of the chestnut blight fungus *Cryphonectria parasitica* were found to encode STAND proteins (Choi et al. 2012). Although fungal STANDs have been initially identified in the context of heterokaryon incompatibility (conspecific nonself recognition), it appears that the role of fungal STAND proteins is not limited to heterokaryon incompatibility, as the number of STAND-encoding genes greatly exceed the number of incompatibility genes. There are several reports indicating that STAND proteins are polymorphic and rapidly evolving and subject to extensive expansion in paralogous gene families in a variety of fungal species (Fedorova et al. 2008; Martin et al. 2008; Burmester et al. 2011; Kubicek et al. 2011; Zuccaro et al. 2011; Iotti et al. 2012; van der Nest et al. 2014). In *Tuber melanosporum*, an expanded *nank* (NACHT ANK) family is, in addition, characterized by a remarkable diversification mechanism based on alternative splicing of multiple codon-sized microexons (Iotti et al. 2012).

Based on the similarity between fungal STAND proteins and plant and animal NLRs and their involvement in nonself recognition and programmed cell death, we have proposed that STAND protein may also correspond to general nonself receptors in fungi (Paoletti and Saupe 2009). This proposed function could account for their high level of polymorphism and rapid diversification, and their expansion in certain species critically depends on interorganismal interactions. Although the genomics of NLRs in plant and animal species and lineages has been the subject of many studies, the overall distribution and organization of NLR-related genes in the fungal phylum has not been investigated systematically to date. The fungal phylum offers the advantage of an extensive genomic coverage with several hundred completed genomes currently available (Grigoriev et al. 2014). Herein, we have analyzed 198 complete fungal genomes (corresponding to 164 different species) for the presence of NLR related proteins. We report on the NLR domain architecture, variability and repertoire size in these 164 fungal species. We find evidence of extensive variation of NLR copy numbers both within and between species. Several NLR domain architectures appear presently restricted to the fungal phylum, whereas others also exist in animal or plant lineages. NLRs appear restricted to filamentous species and are missing from yeast genomes, suggesting that presence of NLRs is associated with multicellularity. Our data suggest an extensive modularity of domain associations, with recurring inventions of domain architectures. Finally, a proportion of the C-terminal domains of NLRs show strong internal conservation, as described for the rapidly evolving HNWD family of *P. anserina*. We find evidence for positive diversifying selection acting on C-terminal domains of the TPR and ANK type, as previously reported for the WD repeats. This overall picture of NLR protein repertoire in fungal genomes now highlights similarities and differences between nonself recognition strategies in different eukaryotic lineages and sheds new light on the evolutionary history of this type of receptors.

## Materials and Methods

### Identification

IR and functionally validated (FV) queries were obtained by extraction of NACHT and NB-ARC domains from the full-length sequences according to PfamA PF05729.7 and PF00931.17 profile matches (Finn et al. 2014). PSI-BLAST searches (Altschul et al. 1997) with three iterations and an *E* value cut-off of 10^−5^ were carried independently for each query sequence on the NCBI “nr” database (June 27, 2013), and then combined. The candidate set was pruned from sequences with multiple disjoint matches to the queries and from very short sequences (below 100 amino acids). Then it was limited to sequences from complete or draft whole-genome sequencing and resequencing projects, according to Genome OnLine Database ([Pagani et al. 2012], as of September 18, 2013), for which at least 2,000 sequences were available in the nr database. Intrastrain identical copies of sequences were removed, whereas interstrain identical sequences were kept. Boundaries of the NB domain were determined as the longest stretch of matches from all NACHT and NB-ARC queries in the PSI-BLAST search. Proportional Venn diagrams were generated using BioVenn (Hulsen et al. 2008).

Noncanonical P-loop variants were detected by inspecting single-residue changes at the four conserved positions of the motif in multiple sequence alignments of NB domains generated by Clustal Omega 1.1.0 (Sievers et al. 2011), with two iterations separately for nonredundant sets of 4596 NACHT and 1174 NB-ARC STANDs found in the entire nr database (not limited to whole-genome projects).

### Annotation

In-house signatures were generated using HMMER 3.0 (Eddy 2011) for the HET-s, PP, and σ prion-forming domains, and the NAD1, Goodbye, HeLo-like, sesA, and sesB domains. Representative sequences of prion-forming domains were aligned using several tools: ClustalW 2.1 (Larkin et al. 2007), ClustalOmega 1.2.0 (Sievers et al. 2011), Mafft 7.029b (Katoh and Standley 2013), and Muscle 3.8.31 (Edgar 2004). The best alignments in terms of the normalized Median Distance (norMD, [Thompson et al. 2001]) were used for the HMM training with default parameters (Durbin et al. 1998). A single representative sequence for each nonprionic domain was submitted to the HHsenser web tool (PSI-BLAST parameters: *E* value cut-off of 10^−^^3^, coverage of hits at least 50% [Soding et al. 2006]) to build a data set including at least 500 sequences in the “permissive” alignment. “Strict” alignments were retrieved and used in iterative HMM training. After each round of the training, sequences with the score below 25.0 or the score/bias ratio below ten were excluded from the alignments; the procedure was repeated until convergence. Finally, the in-house HMMs were included in a PfamA-style repository with their sequences and domain thresholds set to 25.0.

STAND sequences were scanned using PfamA and in-house signatures. Particular annotation was attributed to a given domain if the HMM profile match was entirely contained within domain boundaries extended by a 20 residue-wide envelope. In the case of overlapping annotations from the same PfamA clan, the hit with the lower *E* value was chosen, except for the P-loop NTP-ase clan (CL0023), where NACHT or NB-ARC annotations were always preferred (if above the PfamA threshold). Annotations from repeat-containing clans: Ankyrin (CL0465), Beta propeller (CL0186), and TPR (CL0020, includes HEAT repeats) were merged to three main categories: ANK, WD40, and TPR, respectively. Highly overlapping N-terminal annotations, as well as three prion-forming domain annotations were merged (see Results). Conflicting annotations from PfamA HeLo and in-house HeLo-like signatures were resolved in the favor of the former. Numerical suffixes of signature names were truncated and sequential occurrences of identical annotations were squeezed. Domain associations were visualized using the graphviz package (Gansner and North 2000).

Distribution of domain architectures was quantified by means of paralog and ortholog hits. Ortholog index counted number of species (distinguished by binomial name) in which a given architecture was found. Paralog index summed the number of sequences with a given architecture in all species (the average number was added if several strains were sequenced for the particular species).

### Phylogeny

All phylogenetic trees were calculated through the maximum likelihood estimation based on alignments of NB or N-terminal domains extracted from nonidentical sequences. In each case, the best alignment was selected according to the norMD score out of alignments generated by the same MSA tools as above. Then, the alignment was pruned from columns with more than 50% of gaps (using trimAl [Capella-Gutierrez et al. 2009]) and submitted to PhyML 3.0 (Guindon et al. 2010) with default options (model LG, tree topology search NNI). Interstrain identical sequences were added to the trees after estimation. Phylogenetic trees were drawn using the R project with the “ape” (version 3.0-8, [Paradis et al. 2004]) and “phangorn” (version 1.7-4, [Schliep 2011]) packages, and the TreeDyn editor (Chevenet et al. 2006; Dereeper et al. 2008).

Genes with no clear ortholog in all (“orphans”) or in some other strains (“semiorphans”) from the same species were identified according to co-phenetic distances between leaves in multistrain phylogenetic trees (using the R package ape). To detect highly homologous pairs of NB domain sequences associated with different N-terminal domains, BLASTP scores (Altschul et al. 1997) were calculated in the all-against-all manner for the entire data set of NB domains. A target was counted as highly similar to the query if the match scored at least 99% of the maximum score obtained by the query. To avoid false positives, only matches with at least 80% identity over 100 or more amino acids were counted; sequences with unknown N-terminal annotation were also excluded.

### Repeat Domain Analysis

Highly internally conserved repeats were detected using T-reks (Jorda and Kajava 2009) with customized parameters (PSIM = 0.85, kmeans = 10, overlapfilter on, external MSA: ClustalW 2.1, and Muscle 3.6); repeat regions shorter than 100 amino acids were filtered out. Sequences from dikarya, metazoan, and viridiplantae belonging to ANK, WD40, and TPR clans were extracted according to designation in the Pfam repository (27.0) and availability in the nr database (June 27, 2013). The content of highly internally conserved repeats was calculated as above. “Skipredundancy” from the EMBOSS package (Rice et al. 2000) was used to obtain the nonredundant count of the highly conserved repeats.

For analysis of *P. anserina* ANK and TPR motifs, genes-encoding STAND proteins with individual repeats displaying over 85% internal conservation were analyzed, excluding *hnwd* family members, resulting in a set of ten genes. They code for TPR, ankyrin, or HEAT repeats. For each gene, the repeat-encoding DNA was polymerase chain reaction (PCR)-amplified from five wild isolates from the Wageningen collection (Wa94, Wa 96, Wa97, Wa99, and Wa100) (van der Gaag et al. 1998), gel-purified, cloned in the XL-PCR-TOPO plasmid (Invitrogen, Life Technologies) and sequenced. Sequences were manually assembled before further analysis. In addition, sequences from the S strain were extracted from the *P. anserina* genome sequence and were added to the data set.

Protein repeats were identified using RADAR (Heger and Holm 2000). Individual repeats were then aligned using ClustalW and a neighbour-joining tree constructed using MEGA5 (Tamura et al. 2011). Sequences clustering together with a high bootstrap support were analysed further, and the other were discarded from the data set. For each data set, sequences in duplicates were then discarded, so that a single copy of each repeat sequence was maintained. To detect signs of positive selection, five analyses (SLAC, FEL, REL, MEME, FUBAR) were conducted for each data set using the HYPHY suite (Pond and Frost 2005; Pond et al. 2005). The cut-off was set at 95% confidence interval for SLAC, FEL, MEME, and FUBAR analyses, and over 100 for REL analysis. We considered codons as being submitted to positive selection when they were detected as such by at least three of these approaches. As recombination can lead to false-positive identification by these methods, we also ran PARRIS, which account for the possibility of recombination and is proven to be more robust in these conditions (Scheffler et al. 2006). Also, only positions where three or more codons were identified were considered to be under positive diversifying selection. Homology modeling of TPR, ANK, and HEAT repeats was performed using HHPred (Soding et al. 2006), and protein structure graphics were obtained using Polyview (Porollo et al. 2004).

## Results

### Identification of the Fungal STAND NLR Repertoires

To identify NLR-like proteins in the different complete fungal genomes, we have used NACHT and NB-ARC NOD domains from previously identified STAND proteins as queries. We have defined three different query sets. The first set comprised a list of fungal STAND proteins previously identified in the context of the study of fungal incompatibility. This query set we termed IR (incompatibility-related) includes the *P. anserina* HET-E, HET-D, and HET-R incompatibility genes and the fungal STAND proteins comprising a putative prion-forming domain (Paoletti et al. 2007; Daskalov et al. 2012). A second set, termed FV, was constituted of plant and animal proteins that have been validated as bona fide NLRs in functional studies and include, for instance, human NOD1 and NOD2, the NALP receptors and *Arabidopsis* RPP1, 8 and 13 and RPS 2, 4, and 5. A third set, termed PD (phylogenetically diverse), comprised an ensemble of STAND proteins with NACHT and NB-ARC Pfam-A annotations with a large phylogenetic distribution, ranging from bacteria to plants and animals and included sequences from different major lineages (supplementary file S1, Supplementary Material online). The NB-ARC and NACHT sequences were extracted from the different query set and used in PSI-BLAST searches with three iterations and an *E* value cut-off of 10^−^^5^ on the complete annotated genome sequences of 198 strains of 164 fungal species (corresponding to the complete fungal genomes deposited at NCBI at the time of the study, supplementary file S2, Supplementary Material online). The IR, FV, and PD query sets recovered 5,571, 1,053, and 4,657 hits, respectively (supplementary fig. S1, Supplementary Material online). The IR recovered the most hits, whereas the FV set led to the lowest number of hits, but FV hits were almost entirely included in the IR and PD sets. The FV query did recover only a very limited number of NACHT domain STAND proteins, but was more efficient in the identification of NB-ARC STAND proteins (supplementary fig. S1, Supplementary Material online). We included all hits in our candidate set, which thus adds up to 5,616 sequences (supplementary file S2, Supplementary Material online) corresponding to 4,476 (79.7%) and 1,144 (20.4%) NACHT and NB-ARC hits, respectively (four sequences were hit by both NACHT and NB-ARC queries). Hits were found in 122(101) of the 198(164) strains (species). In these 122 strains, there is mean number of STANDs per genome of 46, with a median of 37.

### Fungal NLR Domain Annotation

**Fig. 1.—:**
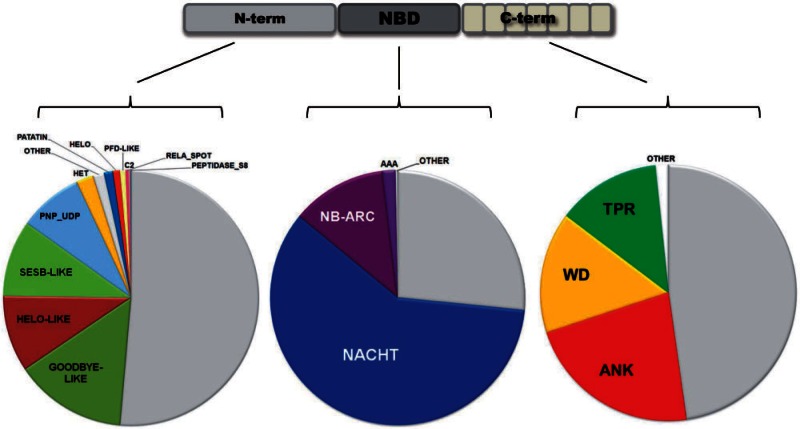
Domain annotation in the fungal NLR set. Pie charts show the distribution of domain annotation in the N-terminal, NOD, and C-terminal domains, respectively. In each pie chart, the light gray corresponds to the fraction of domains with no annotation.

Next, we have annotated the hit sequences using Pfam and in-house annotation tools. Among the NOD domains, NACHT were more frequent than NB-ARC domains, but both categories were abundant (fig. 1 and supplementary fig. S2, Supplementary Material online). The NACHT to NB-ARC ratio is 5:1. This contrasts with the situation observed in *viridiplantae*, where NB-ARC largely predominates (NACHT to NB-ARC ratio based on Pfam annotations is 1:180). In bacterial STAND proteins, both types are common; however, NB-ARC domains are also more abundant than NACHT domains (approximately in a 2:1 ratio). The higher occurrence of NACHT versus NB-ARC makes the fungal NLR candidate set more animal-like, because in metazoans NACHT domains are more frequent than NB-ARC (in a 17:1 ratio). A remaining 28% of the NOD regions picked up in the BLAST searches were neither annotated as NACHT nor NB-ARC by Pfam. Among the candidates were also a number of sequences showing noncanonical P-loop motifs with recurrent variations around the canonical GXXXXGKT/S motif (supplementary table S1, Supplementary Material online), a situation also described in plant NLRs (Bonardi et al. 2012).

NLRs have a typical tripartite domain organization, with a central NOD flanked N-terminally by an effector domain and C-terminally by an autoinhibitory/ligand-binding domain, often composed of superstructure-forming repeats (Leipe et al. 2004). For annotation of the N-terminal domains in addition to the Pfam annotation, we have generated HMM signatures for a series of additional domains that have been found previously as N-terminal domains of fungal STAND proteins (Paoletti et al. 2007; Daskalov et al. 2012). Signatures were generated for the HET-s, PP, and σ prion-forming domains, the NAD1, Goodbye, HeLo-like, sesA, and sesB domains (Daskalov et al. 2012). HMM signatures were generated starting from a relevant individual sequence or a sequence alignment in the case of the short prion-forming motifs (see Materials and Methods). After annotation of the hit sequences, a strong overlap between the sesA and HeLo-like annotated set as well as the NAD1 and Goodbye annotated set was noticed, indicating that these domains are in fact related. For the sake of simplicity, we chose to merge these annotations using the Goodbye and HeLo-like designation for the NAD1/Goodbye group and sesA/HeLo-like group, respectively. It was noted previously that the sesB domain is related to lipases with α/β hydrolase fold (Graziani et al. 2004; Daskalov et al. 2012), and not surprisingly, there was also some level of overlap between the sesB annotation and Pfam annotation related to α/β hydrolases. In this case also, we chose to merge the sesB and the α/β hydrolase Pfam annotations into a single category. We also merged the three PFD signature (HET-s, PP, and σ) into a single category. These motifs are unrelated in primary structure but have similar presumed functions. Among the Pfam annotations, we retained for these analyses only annotations that occur at least ten times in the set. A variety of other N-terminal and C-terminal annotations occur in a very limited number of NLR candidates (supplementary file S3, Supplementary Material online) and were not analyzed further. We end up this way with 12 annotation categories for the N-terminal domains (fig. 1 and table 1). Among the annotated domains, the most frequent domains encountered as N-terminal effector domains are the Goodbye-like, HeLo-like, sesB-like, and PNP_UDP domains (each in the range of 20%). Then, the HET, Patatin, HeLo, and PFD domains are still relatively common (in the 4–1% range), while the other domains represent less than 1% of the annotations (fig. 1 and supplementary fig. S2, Supplementary Material online). The PNP_UDP domain has been previously identified as an N-terminal effector domain in NLR proteins from the coral *A. digitifera* (Hamada et al. 2013), and a sesB-related α/β hydrolase fold was found in a putative NLR in a bryophyte (Xue et al. 2012). Globally, roughly half of the sequences show no annotation in the region N-terminal to the NOD domain. In particular, in the basidiomycota, our annotation of the N-terminal domain is very limited with about only 15% of the sequences receiving an annotation (supplementary fig. S2, Supplementary Material online).

**Table 1:** List of the 12 Annotations Classes Retained for the N-Terminal Domains of Fungal NLRs

Designation	Putative Function	Reference and/or PFAM ID.
C2	Membrane targeting	PF00168
Goodbye-like	Unknown	Daskalov et al. 2012, this study
HeLo	Pore formation	Seuring et al. 2012/PF14479
HeLo-like	Unknown	Graziani et al. 2004, this study
HET	Unknown	Smith et al. 2000/PF06985
Patatin	Phospholipase	PF01734
Peptidase S8	Serine protease	PF00082
PFD	Signal transduction	Daskalov et al. 2012/PF11558
PKinase	Protein kinase domain	PF00069
PNP_UDP	Phosphorylase	PF01048
RelA_SpoT	ppGpp synthesis	PF04607
sesB-like	Lipase, esterase	Graziani et al. 2004, this study

In the domain C-terminal of the NOD domains, again only 52% of the sequences matched a Pfam A annotation. Ankyrin, WD-40 and TPR motifs corresponded to, respectively, 42, 29, and 25 % of the annotated sequences (fig. 1 and supplementary fig. S2, Supplementary Material online). In ascomycota, ANK repeats were more abundant whereas WD40 repeats prevailed in basidiomycota. No LRR motifs were found in agreement with a previous study (Soanes and Talbot 2010).

We conclude that fungal genomes encode a variety of NLR-like proteins with a great diversity of N-terminal and C-terminal repeat domains. Whereas the NACHT and NB-ARC, and ANK, WD, and TPR domains have been previously found in plant and animal STANDs, only a fraction of the N-terminal domains (like the PNP_UDP) have also been found in NLRs from other phyla. A large fraction (roughly 50%) of the N-terminal and C-terminal domains do not respond to known annotations.

### Diversity and Plasticity in Domain Architectures

**Fig. 2.—:**
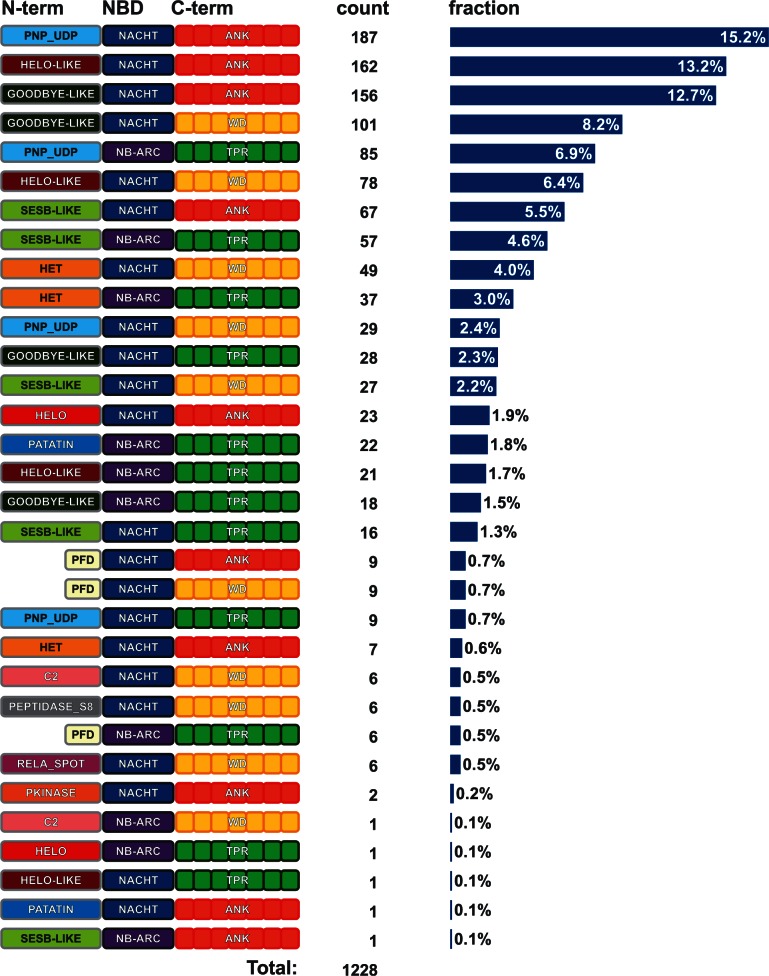
Domain architectures of fungal NLRs. The figures list the domain architectures found in 1,228 NLR candidates with tripartite annotation. For each of the architectures, the total count and percentages are given.

**Fig. 3.—:**
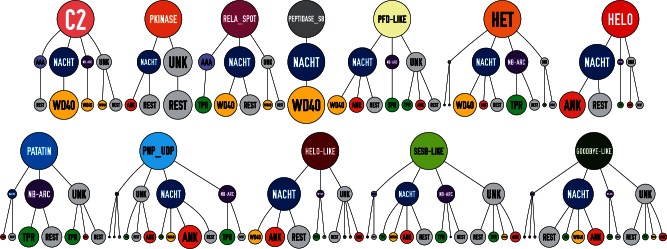
Diagram of preferential domain associations in fungal NLRs. For each of the 12 annotation classes for the N-terminal domains of the fungal NLRs, the type of NOD, and C-terminal domain that are found associated with it are shown. The size of the disk is proportional to the abundance of a given architecture. For the NOD domains, “UNK” denotes unknown (nonannotated) domains. For the C-terminal domains, “REST” denotes unknown (nonannotated) domains and other annotations (distinct from WD, TPR, ANK).

Next, we analyzed the domain architectures of the fungal NLR candidate set. Globally, there is a great diversity of domain architectures. To illustrate this aspect, we focused our analysis on the 1,228 sequences for which all three domains (N-, NOD, C-) have an annotation. The 12 annotated effector domains and NACHT and NB-ARC NOD domains can in principle lead to 24 (12 × 2) domain associations, and of those, 21 occur in our candidate set. Similarly, all six combinations of NACHT and NB-ARC with WD, TPR, and ANK motifs are found in the set. Globally, of the 72 possible tripartite domain architectures (12 effector domains × 2 NOD domains × 3 repeat domain), 32 are actually found in the set (fig. 2). In general, for a given N-terminal domain, a type of architecture for the NOD and C-terminal domain predominates. Some domains show a strong bias in association, for instance HeLo-like and Patatin are almost invariably associated with NACHT and NB-ARC, respectively. Others like HET have a more equilibrated association with either NACHT or NB-ARC. This preferential combinatorial domain association is presented for the 12 N-terminal effector domain types (fig. 3). There is also a preferential association between NOD types and C-terminal repeat type; NACHT is preferentially followed by ANK or WD whereas NB-ARC preferentially by TPR (supplementary fig. S3, Supplementary Material online). These preferential association trends always suffer exceptions, as a small fraction of the NB-ARC domains are associated with ANK or WD, and a small fraction of the NACHTs is followed by TPRs. The fact that in our sequence set some domain architectures are encountered only once suggests that some of the missing architectures might be identified by analyzing additional species.

When inspecting the distribution of annotated N-terminal domains in phylogenetic trees based on the NOD domains, it appears that phylogeny of the N-terminal domains is frequently distinct from that of the NODs. This is apparent in two ways. First, in the global candidate set, the phylogenic trees based on the N-terminal domains are not congruent with the phylogenies of the NODs (supplementary fig. S4, Supplementary Material online). Then, when generating phylogenetic trees from the NLR complement from a given species based on the NOD sequences, domain architectures based on N-terminal domains do no form monophyletic groups but rather are to some extent scattered in different branches of the tree. For instance, in the phylogenetic tree based on the NOD domain of the NLR complement of the species *Bipolaris maydis*, the HET domain is found in different branches of the tree. The same is true for PNP_UDP, Goodbye, and HeLo-like domains (supplementary fig. S5, Supplementary Material online). This distribution and the observed combinatorial domain association suggest that de novo generation of specific domain architectures can occur by domain fusion events between N-terminal domains and a different lineage of NODs. In order to explore this aspect, we analyzed our NLR candidate set for situations in which a given NOD is highly similar to a NOD embedded in a distinct domain architecture. Table 2 lists such situations in which highly similar NODs (between 80% and 99% identity) are associated with totally distinct N-terminal domains. Such situations can be explained by envisioning relatively recent domain fusion events, in which an N-terminal domain was swapped for another.

**Table 2:** Pairs of NLRs with Highly Homologous NOD Domains and Distinct N-Terminal Domains

Gi Ident 1	Tax Name 1	N-Term 1	NOD 1	C-Term 1	Gi Ident 2	Tax Name 2	N-Term 2	NOD 2	C-Term 2	Score	Identity [%]
156035777	*S. sclerotiorum* 1980 UF-70	**HELO-LIKE**	NACHT	WD40	156060563	*S.sclerotiorum* 1980 UF-70	**SESB-LIKE**	NACHT	WD40	240	97.5
156044028	*S. sclerotiorum* 1980 UF-70	**HELO-LIKE**	NACHT	WD40	156060563	*S.sclerotiorum* 1980 UF-70	**SESB-LIKE**	NACHT	WD40	267	99.2
156050803	*S. sclerotiorum* 1980 UF-70	**HELO-LIKE**	NACHT	UNK	156060563	*S. sclerotiorum* 1980 UF-70	**SESB-LIKE**	NACHT	WD40	238	92.4
451851214	*B. sorokiniana* ND90Pr	**HET**	NACHT	WD40	189211806	*P. tritici-repentis* Pt-1C-BFP	**HELO-LIKE**	NACHT	WD40	354	88.3
189209021	*P. tritici-repentis* Pt-1C-BFP	**HET**	NACHT	WD40	189211806	*P. tritici-repentis* Pt-1C-BFP	**HELO-LIKE**	NACHT	WD40	350	86.8
189209021	*P. tritici-repentis* Pt-1C-BFP	**HET**	NACHT	WD40	482814165	*S. turcica* Et28A	**HELO-LIKE**	NACHT	WD40	349	84.8
225559733	*A. capsulatus* G186AR	**PNP_UDP**	NACHT	WD40	159124379	*Aspergillus fumigatus* A1163	**HELO-LIKE**	NACHT	WD40	306	82.3
242760112	*Talaromyces stipitatus* ATCC 10500	**HELO-LIKE**	UNK	UNK	212547165	*T. marneffei* ATCC 18224	**PNP_UDP**	NACHT	TPR	219	89.3
242760112	*T. stipitatus* ATCC 10500	**HELO-LIKE**	UNK	UNK	212547167	*T. marneffei* ATCC 18224	**PNP_UDP**	NACHT	TPR	219	89.3
322704939	*M. anisopliae* ARSEF 23	**SESB-LIKE**	NACHT	ANK	342868671	*Fusarium oxysporum* Fo5176	**PNP_UDP**	NACHT	ANK	317	80.8
322704939	*M. anisopliae* ARSEF 23	**SESB-LIKE**	NACHT	ANK	475672654	*F. oxysporum* f. sp. cub. race 4	**PNP_UDP**	UNK	ANK	319	80.8
347826932	*B. fuckeliana* T4	**HET**	NB-ARC	TPR	472238659	*B. fuckeliana* BcDW1	**SESB-LIKE**	NB-ARC	TPR	563	90.0
353243899	*Piriformospora indica* DSM 11827	**HELO-LIKE**	NACHT	UNK	353245097	*Pi. indica* DSM 11827	**SESB-LIKE**	NACHT	WD40	309	80.8
402073505	*G. graminis* var. *tritici* R3	**HELO-LIKE**	NACHT	UNK	402073554	*G. graminis* var. tritici R3	**RELA_SPOT**	NACHT	UNK	339	83.6
402073505	*G. graminis* var. *tritici* R3	**HELO-LIKE**	NACHT	UNK	402073555	*G. graminis* var. tritici R3	**RELA_SPOT**	NACHT	WD40	339	83.6
402073505	*G. graminis* var. *tritici* R3	**HELO-LIKE**	NACHT	UNK	402085097	*G. graminis* var. tritici R3	**RELA_SPOT**	NACHT	WD40	338	83.6
429861644	*C. gloeosporioides* Nara gc5	**GOODBYE-LIKE**	NACHT	UNK	429850945	*C. gloeosporioides* Nara gc5	**PNP_UDP**	NACHT	UNK	224	82.1
429861644	*C. gloeosporioides* Nara gc5	**GOODBYE-LIKE**	NACHT	UNK	429853607	*C. gloeosporioides* Nara gc5	**PNP_UDP**	NACHT	WD40	222	80.6
46130696	*F. graminearum* PH-1	**HELO-LIKE**	NACHT	WD40	46138235	*F. graminearum* PH-1	**PNP_UDP**	NACHT	UNK	338	85.1

Note.—Gi Ident, GenBank identification.

Together, these observations suggest the existence of a combinatorial assortment of the N-terminal, NOD, and C-terminal repeat domains in fungal STAND proteins that resulted in a large diversity of domain architectures. The fact that domain architecture types do not represent a monophyletic group and the existence of highly similar NODs associated with distinct N-terminal domains, suggest that domain architecture invention events are not limited to a ancestral founding events but may reoccur frequently.

### Highly Conserved WD, ANK, and TPR Domains Are Enriched in Fungal NLRs

**Fig. 4.—:**
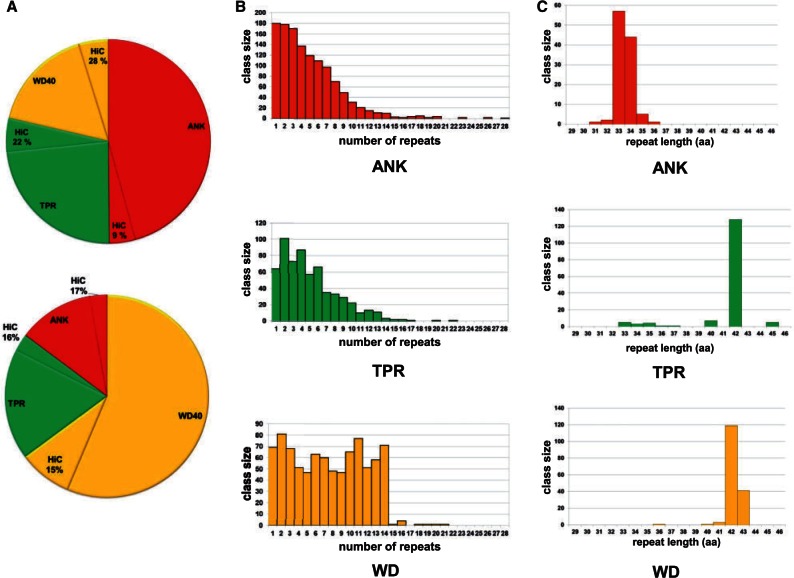
Superstructure-forming repeat domains of fungal NLRs. (*A*) Pie chart of repeat type found in ascomycete (top) and basiodiomycete (bottom) NLR candidates. For each repeat type, the fraction of repeats showing high internal conservation (HiC, 85% identity over at least 100 amino acids) is shown. (*B*) Distribution of the number of repeats in fungal NLR candidates for ANK, TPR, and WD repeats (Pfam signatures PF00023, PF13374, and PF00400, respectively). (*C*) Repeat length distribution in fungal NLR candidates for highly conserved ANK, TPR, and WD repeats.

The analysis of STAND protein evolution in *Podospora* has revealed the existence of a NACHT-WD gene family (*nwd*), characterized by WD-repeats showing a high level of internal repeat conservation, meaning that the individual WD-repeats of a given gene are highly similar to each other (with about 85% identity at the amino acid level) (Saupe et al. 1995; Paoletti et al. 2007; Chevanne et al. 2010). This internal repeat conservation is associated with a concerted evolution of the repeats, caused by constant reshuffling and exchanges of repeats both within a given gene or between different members of the gene family, which allows for rapid diversification (Paoletti et al. 2007; Chevanne et al. 2010). To determine if the presence of highly conserved repeats is a more general occurrence in fungal NLR proteins, we analyzed the NLR set for the presence of internally conserved repeats. Globally, 16% of the annotated repeats were found to show high internal conservation (over 85% identity over a minimum total length of 100 amino acids); respectively, 10%, 21.2%, and 21.6 % of ANK, TPR, and WD-repeats showed high internal conservation (the proportions varied somewhat between ascomycetes and basidiomycetes), (fig. 4*A*). These observations indicate that the internal repeat conservation noted for WD repeats in *P. anserina* is a common property of a significant proportion of the NLR-like proteins and that this phenomenon is also encountered with ANK and TPR motifs both in ascomycetes and basidiomycetes. We have analyzed the occurrence of such highly conserved repeats in ANK, TPR, and WD-type repeats in plants, metazoan, and fungi (supplementary table S2, Supplementary Material online). We found that the fraction of repeats with high internal conservation is globally very low (0.4%, 0.8%, and 1.2% in viridiplantae, metazoan, and dikarya, respectively). There is thus a specific enrichment for highly conserved repeats in fungal NLR proteins. In dikarya, occurrence of highly conserved ANK, TPR, and WD repeats occurs mainly in NLR-like proteins, which globally account for 60-70% of the occurrence of highly conserved repeats. We conclude that highly conserved ANK, TPR, and WD repeats are highly enriched in fungal NLRs, as compared with their global occurrence.

The distribution of the number of repeats per gene was different in ANK and TPR, compared with WD repeats. There was a gradual decrease in the class size with increasing number of repeats per protein in the case of ANK and TPR, while in the case of WD, class sizes were relatively constant from 1 to 14 repeats but then dropped sharply above 14 repeats (enough for the formation of two β-propellers; fig. 4*B*). This difference might be related to the fact that ANK and TPR motifs form open-ended superstructures (Javadi and Itzhaki 2013) rather than closed circular structures (β-propellers) in the case of WD-repeats (Stirnimann et al. 2010). In the case of the TPR motifs, there is also apparently a preference for an even number of repeats. The maximum number of WD repeats was 21, which corresponds to the highest number of WD-repeats identified so far in a WD β-propeller domain and could allow for formation of a triple β-propeller. The occurrence of a low number (<6–7) WD repeats, which a priori do not allow for formation of a closed β-propeller, might be due to the presence of cryptic repeats too degenerate to match Pfam signatures. The size distribution of the repeats corresponded to a very narrow range, typically 33-34 and 42-43 for ANK and WD repeats, respectively. Most TPR motifs were 42 amino acids in length, with only a minor fraction corresponding to in the canonical 34 amino acid length (fig. 4*C*).

Next, we analyzed whether or not highly conserved repeats are randomly associated with the different N-terminal effector domains. All frequent N-terminal domains can be found associated with highly conserved repeats, but it appears that certain N-terminal domains are preferentially associated with highly conserved repeats, as for instance the HET domain but also the prion-forming domains, whereas others like the Goodbye domains are very seldom associated with this type of repeats (supplementary table S3, Supplementary Material online).

### Phylogenetic Distribution

**Fig. 5.—:**
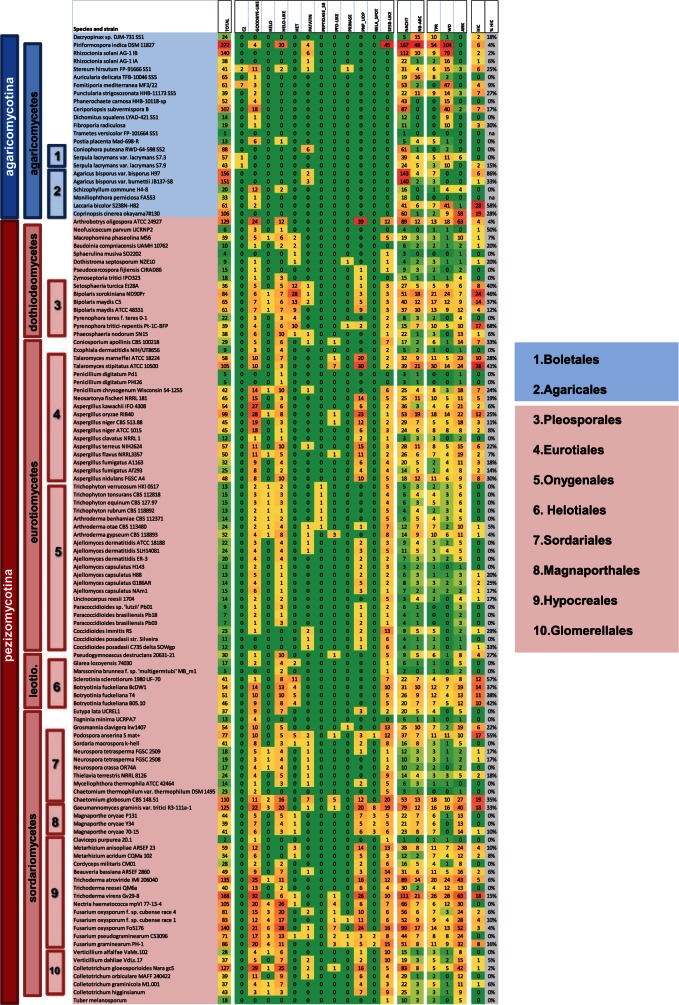
Phylogenic distribution of fungal NLRs. The list of species and strains in which NLR candidates were identified is shown together with their phylogenetic position. For each strain/species, the total count of NLR candidates and of the different N-terminal domains, NOD domains, and C-terminal repeat domains is given, as well as the count and the fraction of the repeat domains that show high internal conservation (HiC).

Next, we analyzed the phylogenetic distribution of NLRs in fungi (fig. 5 and supplementary file S2, Supplementary Material online). NLRs were absent from certain lineages; in particular, no hits were found in any of the 38 analyzed Saccharomycotina genomes, or in the Schizosaccharomycetes. Similarly, we found no hits in early branching lineages of the microsporidia, chytrids, and mucorales. In contrast, hits were abundant in major basidiomycetes (agaricomycetes, 1,589 hits in 22 species, 72 hits per species) and ascomycetes lineages (pezizomycotina, 3,955 hits in 98 species, 40 hits per species). When comparing the annotation of the ascomycota and basidiomycota ensembles, three main trends are apparent. The ratio of NACHT to NB-ARC is slightly different in both lineages, with NB-ARC being rarer in basidiomycota (with a 1:8 ratio of NB-ARC to NACHT, compared with 1:4 in ascomycota). The abundance of the different types of repeat motifs also differs in both lineages: WD, ANK, and TPR account for 27, 9 and 8% of the C-terminal domain annotations in basidiomycota, compared with 10, 27, and 14% in ascomycota. The higher abundance of NB-ARC and TPR motifs in ascomycotina is expected, considering the preferential association of NB-ARC with TPR motifs (supplementary fig. S3, Supplementary Material online). The level of annotation of the N-terminal domains is very different in both lineages, with only 13% of the sequences receiving an annotation in the basidiomycota, compared with 63% for the ascomycota. This difference is probably related to the fact that our in-house annotations derive from ascomycete sequences.

Variation in the number of NLRs per genome is extreme, ranging from 1 (or 0) to 274 in the endophytic basidiomycetes species *Piriformospora indica.* In that species, NLR-like proteins correspond to 2.3% of the total proteins. Fifteen species show more than 100 NLR genes. There can be strong variations in the number of hits even between related species. For instance, within the *Aspergillus* genus, NLR numbers range from 12 to 99. The same is true even between strains belonging to the same species, as discussed below. Yet, in certain lineages of the pezizomycotina, there appears to be some group-specific increase or decrease in the number of hits. In particular, the hypocreales containing several Trichoderma species have significantly higher numbers of NLRs than the rest of the pezizomycotina (78 genes per species as opposed to 40, *P* = 0.006). The onygenales group containing several dermatophytes shows less hits than the rest of the pezizomycotina (16 genes per species, *P* = 0.018).

We compared the occurrence of the 12 different N-terminal domains in the different species and there again the diversity between species is considerable. None of the 12 annotated domains has a universal distribution in all species displaying NLRs but some are found in a large fraction of species like the Goodbye-like, HeLo-like, and sesB-like domains found in NLRs of 88, 73, and 75 species, respectively. Other domains are found in a narrow species range, like the C2 domain found only in a few basidiomycota. As already noted for the total NLRs numbers, there is a high variability in the number of domain occurrences, even for closely related species, with for instance the number of PNP_UDP NLRs ranging from 2 to 23 in different species of the *Aspergillus* genus. Some domains show a strong tendency for marked expansions, while other are usually found as a single occurrence. We calculated a paralog-to-ortholog index, corresponding to the ratio of number of occurrences of the domain to the number of species in which the domain is encountered. The domains showing the highest number of occurrences per species were PNP_UDP and Goodbye, with a mean occurrence of 7.5 and 7.4 per species, respectively, while in contrast HeLo and Patatin domains showed the lowest occurrence (1,4 and 1,7) (supplementary table S4, Supplementary Material online). These two domains are most generally found as one or two occurrences per species, but some rare exceptions of marked expansion occur as for instance for the HeLo domain in the *fusaria*.

When considering the C-terminal repeat domains, the fraction of repeats with high internal conservation varies dramatically between species from 0 to up to 58% in *Laccaria bicolor*. 72 strains, among the 122 displaying NLRs proteins, have at least one gene with internally conserved WD, TPR, or ANK repeats (fig. 5 and supplementary file S2, Supplementary Material online). Species in which such NLR-like proteins with high conserved repeats are particularly abundant are *L. bicolor, B. maydis*, and *Talaromyces stipitatus.*

HSP90 and its co-chaperones SGT1 and RAR1 play important roles in NLR function both in plants and animals (Kadota et al. 2010). We analyzed the complete fungal genomes for presence of putative SGT1 and RAR1 homologs and found SGT1 matches in all analyzed complete genomes and RAR1 matches in 111 out of 122 strains displaying NLR matches.

### Intraspecific Variation Reveals Extensive Polymorphism of the Fungal NLR Repertoire

Previous reports suggest that fungal STAND proteins show high level of intraspecific variation (Paoletti et al. 2007; Fedorova et al. 2008; Burmester et al. 2011; Iotti et al. 2012). In addition, the extensive variation in STAND copy numbers in different species and the specific expansion of certain domain architectures in certain lineages suggest a death-and-birth evolution of these genes in fungi (fig. 5), (Martin et al. 2008; Kubicek et al. 2011; Zuccaro et al. 2011; van der Nest et al. 2014). In order to document this aspect, we chose to assess intraspecific variability in NLR proteins in our candidate set. We have thus specifically compared the NLR complement in 15 species for which the sequences of several strains are available. To compare the gene complement in each strain, phylogenetic trees were constructed based on the NOD domains only, and the trees were inspected for conservation of orthologous pairs (or triplets) between strains (supplementary fig. S6, Supplementary Material online). In all 15 analyzed species, some level of polymorphism in the NLR complement is observed. A variable fraction of the NLR sequences lack a clear ortholog in the other analyzed strain(s). Table 3 presents, for each of the 15 species, the number of NLR proteins that are polymorphic, including the number of NLRs that are orphans (defined as a gene that does not show a clear ortholog in other strains from the same species) or semiorphans (when a pair of orthologous genes are found in two strains but not in a third). We find that NLR proteins are polymorphic between strains of the same species, and the fraction of polymorphic NLRs is systematically higher than for the total proteome (of note however is the fact that the level of polymorphism in the total proteome varies dramatically in different species, as the fraction of polymorphic proteins varies from 9.6% in *Penicillium digitatum* to 100% in *Rhizoctonia solani*). In addition, in many species, a significant proportion of the NLR candidates do not have a conserved ortholog in the other strain(s) (i.e., orphans or semiorphans). For instance, in *Aspergillus niger* CBS 513.88, ten sequence show no ortholog in the other strain (ATCC 1015) with a cut-off distance value of 1, which corresponds to about 50% identity. Inspection of the phylogenetic trees reveals the existence of numerous such orphans as well as strain-specific expansion in certain branches of the tree, arguing for a birth-and-death evolution of these sequences (supplementary fig. S6, Supplementary Material online). This strain-specific expansion is for instance evident *Fusarium oxysporum* Fo5176.

**Table 3:** Polymorphism of NLR-Like Proteins in Different Strains from the Same Species

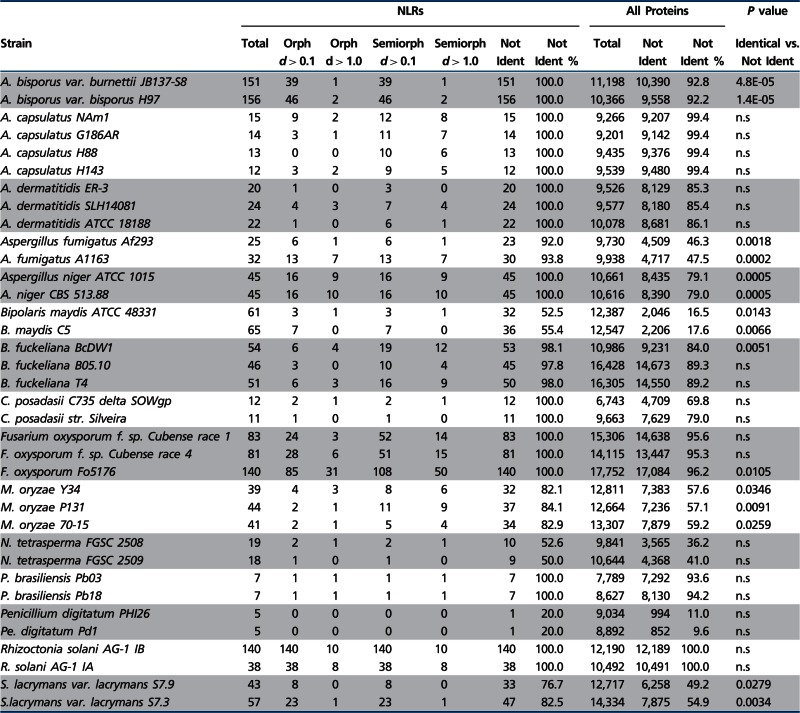

Note.—Orph, orphan; Ident, identification; Semiorph, Semiorphan.

### Relation of HET Domain to TIR Domains

The HET domain acquired this designation because it was found in different proteins involved in nonself recognition in the form of heterokaryon incompatibility in fungi (Smith et al. 2000). In particular, this domain constitutes the N-terminal effector domain of the HNWD family members, which includes the *het-e*, *het-d*, and *het-r* incompatibility genes. Functional studies have identified this domain as being a cell death and incompatibility effector domain in *P. anserina* (Paoletti and Clave 2007). We now find that the HET domain is relatively frequent as N-terminal domain of fungal NLR-like proteins and that it is often found associated with highly conserved repeats, potentially capable of rapid diversification. Because this study shows that many species display HET domain NLR-like proteins, we analyzed this domain further.

**Fig. 6.—:**
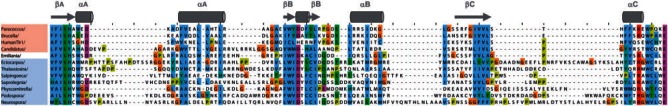
Alignment of fungal HET domains with TIR domain proteins. The TIR domains of two bacterial proteins of known structure and of the human TLR1 TIR domain (boxed in red) are aligned with the HET domains of *P. anserina* HET-e1 and *Neurospora crassa* TOL (boxed in blue) together with related sequence of diverse phylogenetic origin annotated as HET domains in Pfam. On top of the alignment, the elements of secondary structure of *Brucella* TcpB are shown. Sequence designations are as follows: Paracoccus, *Paracoccus denitrificans,* gi|500070302; Brucella, *Brucella melitensis*, gi|516360271; Human Tlr1, *Homo sapiens*, gi|194068387; Candidatus, *Candidatus Accumulibacter*, gi|589611804; Emiliania, *Emiliania huxleyi*, gi|551574256; Ectocarpus, *Ectocarpus siliculosus,* gi|298709304; Thalassiosira, *Thalassiosira pseudonana*, gi|224000455; Salpingoeca, *Salpingoeca rosetta*, gi|514691135, Physcomitrella, *Physcomitrella patens,* gi|168042266; *Podospora*, *P. anserina*, gi|3023956 (HET-e1); Neurospora*, Neurospora crassa*, gi|553134703 (TOL).

We first conducted PSI-BLAST searches in the nr (“nonredundant”) database with the HET-e1 HET domain by excluding fungal sequences and found that homologs of this domain are also found outside of the fungal kingdom in Stramenopiles, Haptophyceae, Choanoflagellates, green algae, and bryophytes. Next, we used Hidden Markov model searches to identify remote homologs of the HET-e1 HET domain. Both algorithms that we used (HHpred and JackHHmer) identified similarity to TIR domains. In particular, the two best hits in HHpred were to structure-based profiles constructed from the TIR domain of PdTIR from *Paracoccus denitrificans* (Chan et al. 2009) and of the TcpB protein from *Brucella melitensis* (Kaplan-Turkoz et al. 2013; Alaidarous et al. 2014; Snyder et al. 2014). Figure 6 presents an alignment of fungal HET domain proteins with bacterial and human TIR domains of known structure and related HET domains from phylogenetically diverse origins. The region of similarity of roughly 100 amino acids encompasses three main conserved blocks. These blocks of similarity map to the elements of secondary structure of the TIR domain α/β fold; the alignment, however, does not include the entire TIR domain, as similarity drops off after the region corresponding to helix αC. TIR domains function as adaptor domains in cell death and immune defense signaling cascades and function by interacting with partner TIR domains (O'Neill and Bowie 2007). This potential homology between HET and TIR domains suggests that HET domains may function by recruiting HET domain proteins and signaling downstream.

### ANK and TPR Motifs of NLR Proteins of P. *anserina* Show Repeat Length Polymorphism and Positive Diversifying Selection

Superstructure-forming repeats with high internal conservation are enriched in fungal NLRs. These repeats belong to three types of superstructure-forming repeats, WD, ANK, and TPR motifs. We have previously shown that WD repeats of NLR-like proteins show extensive repeat size polymorphism in *Podospora* and are subject to concerted evolution and positive diversifying selection (Paoletti et al. 2007; Chevanne et al. 2010). We extended this analysis to ANK and TPR motif NLR proteins of *Podospora,* in order to determine whether repeat size polymorphism and diversifying selection was a common property of such repeat domains. We selected eight *P. anserina* NLR-encoding genes showing highly conserved ANK and TPR motifs, and PCR-amplified the repeat region from genomic DNA from five different wild isolates. For each locus, sequence analysis revealed repeat number polymorphism (RNP) (table 4). ANK repeat numbers ranged from 7 to above 16, whereas TPR motif numbers ranged from 2 to above 14. The RNPs observed suggest frequent recombination between repeats within a locus, and possibly between loci encoding the same type of repeats, as previously reported for WD-repeats (Paoletti et al. 2007; Chevanne et al. 2010).

**Table 4:** Repeat number polymorphism in ANK and TPR Repeat Domains of NLR Proteins from *Podospora anserina*

	Pa_2_8180 PNP-UDP/ NACHT/ ANK	Pa_3_8560 PNP-UDP/ NACHT/ ANK	Pa_2_10340 sesB-like/ NB-ARC/ TPR	Pa_3_9910 PFD/ NB6ARC/ TPR	Pa_5_8060 PFD/ NB-ARC/ TPR	Pa_6_7270 sesB-Like/ NACHT/ TPR(HEAT)	Pa_6_7950 sesB-like/ NACHT/ TPR	Pa_7_3550 UNK/ NB-ARC/ TPR
S	10	7	11	4	4	10	2	11
Wa94	ND	8	>15	9	7	3	13	ND
Wa96	8	12	>14	9	8	6	11	>13
Wa97	13	>14	ND	11	9	12	9	>13
Wa99	>16	11	2	10	7	ND	ND	>13
Wa100	10	10	7	ND	ND	10	10	>13
Total	>57	>62	>49	43	35	41	45	51
Unique	22	36	39	19	21	19	29	29

Note.—ND, not determined.

**Fig. 7.—:**
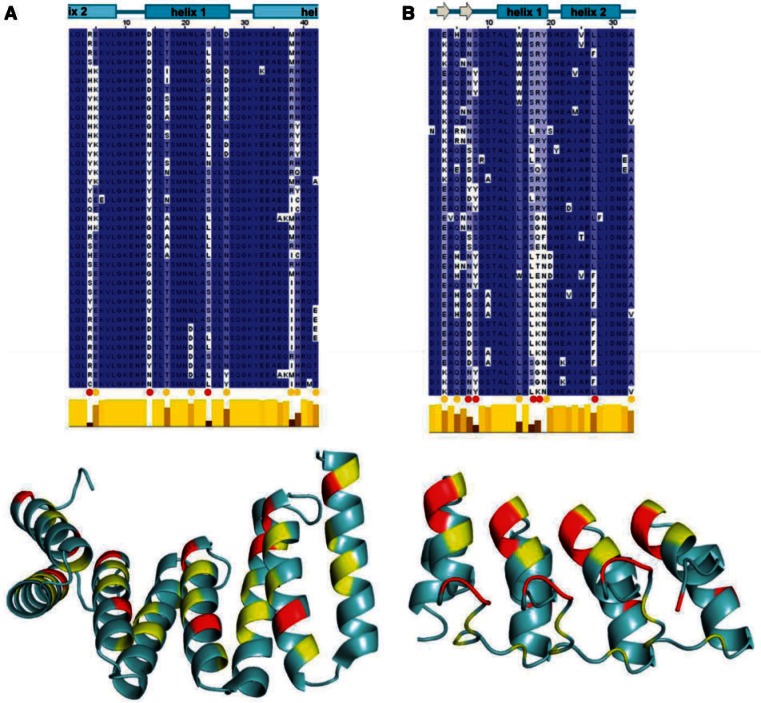
Hypervariable sites in *P. anserina* TPR and ANK repeats of NLRs. (*A*) Alignment of individual TPR motif sequences found in different alleles of *Pa_2_10340* (sesB-like/NB-ARC/TPR) is shown. Positions under positive selection are marked with a red dot; other highly variable positions are marked with a yellow dot. The TPR domain of Pa_2_10340 was modeled using the human kinesin light chain 2 structure as (PDB ID 3EDT) as the template. Color coding of the positive selection and variable sites is as above. (*B*) Alignment of individual ANK repeat sequences found in different alleles of *Pa_3_8560* (PNP_UDP/NACHT/ANK) is shown. Positions under positive selection are marked with a red dot, other highly variable positions are marked with a yellow dot. The ANK repeat domain of *Pa_3_8560* was modeled using the structure of the artificial ANK repeat domain of the engineered protein OR264 (PDB ID 4GPM) as the template. Color coding of the positive selection and variable sites is as above.

Next, we selected one ANK repeat locus and one TPR motif locus for which we had sequenced the highest number of repeats (Pa_3_8560 and Pa_2_10340, respectively) and analysed the variability of the repeats from individual loci. For each locus, individual repeat sequences were aligned and analysed for position under positive selection (see Materials and Methods) (fig. 7). Five positions showed signs of positive selection in the ANK repeats and three in the TPR motifs. To locate the positive selection and polymorphic sites on the repeat domain structure, the repeats were homology-modeled to ANK and TPR domains of known structure. The TPR motif domain of Pa_2_10340 was modeled using the human kinesin light chain 2 structure (Protein Data Bank [PDB] ID 3EDT) as template. In the TPR motifs, all positive selection sites as well as the other polymorphic position mapped to the concave side of the TPR structure in the α-helical regions. The ANK repeat domain of Pa_3_8560 was modeled using the structure of artificial ANK repeat domain of engineered protein OR264 (PDB ID 4GPM) as template. In the ANK repeats, with one exception, the positive selection and polymorphic site also mapped on the concave surface of the ANK repeat domain in the inner helices and the β-hairpin/loop region, which correspond to the binding interface of ANK repeats based on cocrystal structures (Javadi and Itzhaki 2013).

We also analysed two putative proteins from different species to determine whether this localization of the polymorphisms might be common to other ANK and TPR motifs. We chose the ANK and TPR proteins with the highest number of highly conserved ANK and TPR motifs, gi116208038 from *Chaetomium globosum* (PNP_UDP/NACHT/ANK) and gi255934897 from *Penicillium chrysogenum* (UNK/AAA/TPR), with, respectively, 21 ANK repeats and 21 TPR motifs. By comparing the repeats and mapping the variable positions onto a homology model (PDB ID 4GPM for ANK and 3EDT for TPR), we found that polymorphisms map to the same positions in the α-helices of the concave surface of the TPR domain and to the inner helices and β-hairpin/loop region of the concave interface of the ANK domain (supplementary fig. S7, Supplementary Material online). Based on the localization of these polymorphic sites, it can be inferred that if repeat contraction/expansion/shuffling occurs in these genes, these events will lead to ANK and TPR arrays with modified binding interfaces.

Collectively, these analyses suggest that the evolution of ANK and TPR motifs of *Podospora* NLR candidates is analogous to the evolution of highly conserved WD repeats of NLR-like proteins, which has been described previously (Paoletti et al. 2007; Chevanne et al. 2010).

## Discussion

In plants and animals, NLRs are essential components of innate immunity. Work on fungal incompatibility revealed the existence of NLR homologs in fungi with functions in the detection and response to nonself. Herein, we have analysed close to 200 fungal genomes for the presence of NLR candidates and describe the identified sequences. We find that multicellular pezizomycetes and agaricomycete generally encode large and diverse repertoires of NLR-like genes.

### Diversity of N-Terminal Effector Domains

Many of the N-terminal effector domains of fungal NLRs remain completely uncharacterized, in particular in basidiomycotina. We have nevertheless defined 12 main annotation classes for these N-terminal domains that roughly accounts for 50% of the sequence set. For some of these domain classes, functional information is available, although it is in most cases fragmentary. One of the domains, that was previously identified as an effector domain in animal NLRs is the PNP_UDP domain. Indeed, this domain was found as N-terminal domain of NLRs in the coral *A. digitifera* (Hamada et al. 2013), and also as an effector domain associated with a DD in sponge (Yuen et al. 2014)*.* In addition, we reveal a remote similarity between the HET domain and the TIR domain, originally identified in Toll-like receptors in mammals and also found as the N-terminal domains of a large fraction of plant NLRs. Considering this similarity, it might be hypothesized that HET domain fungal NLRs are functionally analogous to plant TIR-NB-LRR proteins. TIR domains regulate immune responses by homo and heterodimerization; HET-domain containing NLRs like the *P. anserina* HET-e, HET-d, and HET-r proteins may therefore mediate the incompatibility response by interaction with downstream HET domain proteins acting as adaptor domains.

A large fraction of the N-terminal domains is related to the HeLo domain identified in the HET-s prion protein of *P. anserina* (Greenwald et al. 2010; Seuring et al. 2012). This domain is a cell death execution domain that can be activated following prion transconformation of the PFD region of HET-S. The HeLo domain is then translocated to the cell membrane, where it functions as a pore-forming toxin (Mathur et al. 2012; Seuring et al. 2012). The HeLo domain is found as the N-terminal domain of NLRs in many different species, but even more frequent is a variant form of this domain that we term HeLo-like, which could potentially play a similar role in cell death execution. Another abundant class is the sesB-like domain, which corresponds to a predicted lipase domain (Graziani et al. 2004; Daskalov et al. 2012). This lipase domain is found in the human SERAC1 protein, which was found to be involved in a metabolic disease (Wortmann et al. 2012). Human SERAC1 displays phospholipid esterase activity and is able to modify lipid composition of the plasma membrane. It might be that sesB-like domains induce specific plasma membrane modification in response to nonself. Our annotation list contains another lipase domain, namely the Patatin domain. Interestingly, the Patatin lipase domain was involved in the control of PCD and defense in plants (Cacas et al. 2009; La Camera et al. 2009; Kim et al. 2014). Based on the fact that one of the incompatibility genes of the fungus *C. parasitica* encodes an NLR with a Patatin domain, it can be reasonably inferred that Patatin-like domains might also function in the control of cell death in fungi. Considering that the C2 domain, found as N-terminal effector domain in basidiomycete NLR candidates, is a lipid-binding domain (Corbalan-Garcia and Gomez-Fernandez 2014), it appears that a significant fraction of the identified N-terminal domain of fungal NLRs target membranes or lipids.

The RelA_SpoT domain was so far only described in bacterial and plant chloroplast proteins (Atkinson et al. 2011); we now identify it as the N-terminal effector domain of fungal NLRs. This enzymatic activity-carrying domain is responsible for the synthesis of the ppGpp bacterial alarmone, which mediates the stringent response in bacteria (Boutte and Crosson 2013). One possible explanation of the presence of this domain as an N-terminal domain of fungal NLRs would be that fungi exploit the prokaryotic signaling ppGpp cascade to manipulate bacterial pathogens, competitors, or symbionts. The same might be true for the PNP_UDP class. Based on the analysis of the PNP_UDP domain in NLRs from *P. anserina*, these domains are predicted to be methylthioadenosine/*S*-adenosylhomocysteine nucleosidases, which are involved in the synthesis of quorum-sensing molecules like Al-2 (Parveen and Cornell 2011). Maybe these effector domains manipulate prokaryotic signaling in the context of adverse or beneficial interactions.

Globally, when one considers domains found N-terminal to the NOD domain in this NLR collection, two main categories emerge. Class 1 domains correspond to domains that have a proposed enzymatic function or a potential direct role in cell death induction. In this first class, one finds the proposed PNP_UDP, RelA_SpoT, sesB-like, and Patatin lipase domains and also the HeLo and HeLo-like proposed pore-forming toxin domains. In this class, the N-terminal domain is believed to represent a direct effector of the NLR activation. Class 2 domains correspond to domains that more likely have an adaptor function, a situation more typical of plant and animal NLRs, where domains such as CARD, PYD, and TIR recruit effectors by homotypic domain interactions to signal downstream, rather than representing the terminal effector of the immune cascade. CARD and PYD mediate NLR signaling by a prion-like mechanism, involving formation of higher-order complexes (Wu 2013; Cai et al. 2014; Lu et al. 2014). The three prion-forming domains (HET-s, PP and σ) associated with fungal NLRs correspond to this second class (Daskalov et al. 2012). The HET domain, possibly homologous to the TIR domain, also likely corresponds to this second class. Many of the fungal NLR-related proteins fall into class 1, while apparently in plant and animal lineages this situation is less frequent, although as mentioned previously PNP_UDP NLRs have been described in corals (Hamada et al. 2013). In complex (multicellular) bacterial STAND proteins, the presence of N-terminal domains with predicted enzymatic activity such as a metacaspase domain is common, in particular in cyanobacteria (Leipe et al. 2004; Asplund-Samuelsson et al. 2012). It might be that at the base of STAND protein evolution the all-in-one architecture is ancestral and that incorporation of adaptor domains between the NLR receptor and the effector represents a further sophistication of the signaling process.

### Superstructure-Forming Repeat Domains in Fungal NLR-Related Proteins

We failed to identify NLR candidates with LRR motifs, a situation already reported in a study specifically tailored to the identification of LRR pattern-recognition receptors in fungal genomes (Soanes and Talbot 2010). Instead, STAND proteins displayed ANK, TPR, and WD motifs. ANK, TPR, and WD motifs were found associated with NLRs in the coral *A. digitifera* (Hamada et al. 2013)*.* Similarly, in their analysis of the repertoire of NLRs in the sponge *Amphimedon queenslandica,* Yuen et al. (2014) also reported that in several holozoan and nonholozoan genomes NACHT domain proteins are associated with ANK, TPR, and WD repeats, but no LRR motifs were found (Yuen et al. 2014). Based on the unified NLR nomenclature proposed in 2008, these authors stated that such non-LRR STAND proteins should not be designated NLR and that this designation should be restricted to proteins encompassing LRR motifs. In that sense, all candidates identified in the fungal genomes would not represent bona fide NLRs. We do however consider that given the combinatorial association of different repeat domains with NACHT or NB-ARC domains in fungi and other lineages, it is reasonable to assume that regardless of the type of superstructure-forming repeats they harbor, these NLR-related genes display related functions. Although a restrictive nomenclature offers the advantage of simplicity, because it is based on domain architecture, it may not be optimal for a global understanding of the role and evolution of NLR-related genes across phyla. As another illustration of this principle, the DEATH-NACHT domain proteins are found in the cnidarian *Hydra magnipalpillata* that lack LRR motifs but cluster with vertebrate NLRs (Yuen et al. 2014). Similarly, Hamada et al. (2013) also favor the notion that different NOD/WD, ANK, TPR, and LRR associations are ancestral and that in certain lineages, NOD/LRR architectures have flourished whereas other architectures were lost (Hamada et al. 2013). Following this plausible model, it might be proposed that the NOD/LRR architecture was specifically lost in the fungal lineage while NOD/TPR, ANK, and WD architecture were expanded. NLR loss in certain lineages is not uncommon; nematodes and arthropods are apparently devoid of NLRs (Maekawa et al. 2011; Hamada et al. 2013) and TIR-NB-LRRs have been reduced or lost in monocotyledon plants (Joshi and Nayak 2013).

A significant fraction of the superstructure-forming repeat domains in fungal NLRs show strong internal conservation, a situation we have previously described for the WD-repeat domains of the *nwd* gene family of *Podospora* (Saupe et al. 1995; Paoletti et al. 2007; Chevanne et al. 2010). We have found that this internal conservation corresponded to the concerted evolution of the repeats both within and between members of the gene family, and was typically associated with repeat number polymorphism. In addition, these WD-repeats show positive diversifying selection at specific codon positions, corresponding to amino acid positions defining the ligand-binding interface of the WD β-propeller structure (Paoletti et al. 2007). Due to the high conservation of the repeats, these sequences are prone to RIP (repeat induced point mutation), a genomic defense mechanism that mutates and methylates repeated sequences premeiotically in fungi (Selker 1990). At least in *Podospora*, the effect of RIP on these repeat regions might represent a mechanism of hypermutation, allowing a rapid diversification of these sequences. We have proposed that the combination of these evolutionary mechanisms constitutes a process for generating extensive polymorphism at loci that require rapid diversification. This study now suggests that this regimen of concerted evolution and positive diversifying selection might be of general relevance to the evolution of a fraction of fungal NLRs. We find that many superstructure-forming repeat domains in fungal NLR show strong internal repeat conservation and that in *Podospora,* ANK and TPR motifs also show RNP and signs of positive selection at positions predicted to be located in the interaction surfaces in the ANK and TPR structures. In the context of nonself recognition, rapid diversification of the receptors might be particularly critical; it appears that the modularity and plasticity properties of superstructure-forming repeats might have been exploited in many fungal species, to allow diversification of their NLR repertoires.

Among the three superstructure-forming repeat types, ANK repeats were the most common in fungal NLRs candidates. The involvement of ANK repeats in host-symbiont or host–parasite interaction was highlighted by previous studies, showing that ANK repeat proteins are enriched in symbiotic and obligate intracellular bacterial species, as compared with free-living species (Jernigan and Bordenstein 2014). Similarly, a rapidly evolving family of ANK repeat proteins was found to control host–parasite interaction in *Wolbachia* (Siozios et al. 2013). ANK repeat domain proteins were also found to be specifically enriched during expression changes associated with nonself recognition in *Podospora* (Bidard et al. 2013). Thus, across phyla, ANK repeat domains appear often to be involved in the regulation of inter-organismal interactions.

### Architectural Diversity of Building Blocks in NLRs

One of the marked characteristics of the fungal NLRs is the extensive domain architecture diversity. Studies of the NLR repertoires in lower animals already hinted at this diversity in domain architecture (Lange et al. 2011; Hamada et al. 2013; Yuen et al. 2014). The description of the fungal NLRs further illustrates this diversity. Even with the very partial annotation, we establish a great variety of architectures, revealing a combinatorial association of different N-terminal, NOD and repeat domains. This diversity is evident both in the phylum and within a given species, which can display tens of different NLR domain architectures. Importantly, in many cases a given domain architecture does not have a monophyletic ancestry. Rather, it appears that reoccurring domain fusion events lead to multiple independent inventions of the same architectures. These domain associations appear not to be limited to ancestral events, as suggested by the fact that NOD with 99% identity can be found associated with totally distinct N-terminal domains. These observations, as well as the species or strain-specific expansions of paralogs, are compatible with the notion that fungal NLRs evolve by a birth-and-death regimen. Others have previously documented the role of birth-and-death evolution in fungal *het* gene homologs in the basidiomycetes (van der Nest et al. 2014). This apparent plasticity of the NLR repertoire, based on the combinatorial association of a variety of effector, NOD and C-terminal receptor domains, might represent a mechanism that allows a rapid adaptation of the NLR repertoire in the arms-race with the variable biotic environment. The combinatorial build-up of an immune repertoire from a limited set of elementary domain is also a general characteristic of the immune-related proteins in plants and animals (Palsson-McDermott and O'Neill 2007).

### Phylogenetic Distribution of NLRs and Possible Functions in Immunity and Beyond

Our analysis of the phylogenetic distribution of NLR homologs in fungi indicates that their presence is apparently restricted to filamentous multicellular fungi. We found no NLR homologs in yeast species. The simplest interpretation of this lack of NLR homologs in yeast species is that this gene family was lost in unicellular fungi, because the constraints on the management of biotic interactions are fundamentally different for multi and unicellular organisms. *Soma* and *germen* are essentially one and the same thing in the latter organisms, therefore the maintenance of a machinery aimed at protection of the *soma* against parasitism may not be required in yeasts, in particular when considering that one common outcome of NLR-controlled defense in animals, plants, and fungi is programmed cell death. We also failed to identify NLR-related genes in early branching non-dikarya fungal lineages of the chytrids, microsporidia, and mucorales and also in some dikarya basidiomycete lineages such as the tremellomycetes and the pucciniomycotina, in agreement with previous studies (van der Nest et al. 2014). This could indicate that NLR-like genes were lost in these lineages or that the level of divergence of the NACHT and NB-ARC domains used in our search prevented their detection.

Within the filamentous agaricomycotina and pezizomycotina, the number of NLR homologs varies dramatically between species. One may attend to establish a relationship between the species ecology and the constitution of the NLR homolog repertoire (supplementary file S2, Supplementary Material online). This can only be made with extreme caution, because in many cases, the information available on the species ecology is at best fragmentary and many species have multiple habitats and life-styles. In some groups, there is a significant enrichment or scarcity of NLRs. For instance, animal dermatophytes of the onygenales have in general few NLR genes. But it is difficult to determine whether this is related to the phylogenetic position or to ecology. If the function of NLR homologs in fungi is related to innate immunity, the prediction might be that fungi potentially in relation to diverse pathogens or competitors or hosts should be particularly enriched in terms of NLR repertoire, and reciprocally, that in fungi living in less populated niches, smaller repertoires could be sufficient. This prediction might be verified in some instances as, for example, in the case of the highly versatile pathogens like *Fusarium* species or mycoparasitic Trichoderma species, in which the repertoire is large. In the thermophile *Chaetomium thermophilum,* the citrus fruit pathogen *Pe. digitatum* or the “whisky fungus” *Baudonia compniacensis* have small repertoires and inhabit restrictive niches. Similarly, specialized pathogens, such as *Claviceps purpurea*, might be protected against microbial competitors by their host immune system, which could explain the low number of NLRs.

The current view of the role of the NLRs in the animal lineage is expanding. Initially viewed as immune receptors whose role is to detect and respond to pathogenic nonself, it is becoming apparent that these receptors are also critical for the management of other nonpathogenic biotic interactions, notably with the symbiotic microbiome (Chu and Mazmanian 2013). For instance, the human NOD2 NLR is required for the establishment of a commensal microbiome in the intestine (Petnicki-Ocwieja et al. 2009). Similarly, it has been proposed that the expanded NLR repertoires in the coral *A. digitifera* could be devoted to the interaction with an obligate dinoflagellate endosymbiont (Hamada et al. 2013). In the fungal kingdom, it has been emphasized that pathogenesis and symbiotic interaction are based on similar mechanisms (Veneault-Fourrey and Martin 2011). It might thus be proposed that part of the NLR repertoires found in fungi might function in the control of a variety of biotic interactions and not be strictly devoted to an immune function per se (understood as the response to pathogenic nonself). These proteins might be involved in the control of nonself recognition in the context of fungal pathogenicity, or symbiosis in the form of ECM formation, endophytic growth, lychen formation, or interaction with symbiotic endobacteria. As already mentioned, NACHT domain proteins are specifically expressed during mycorhizal symbiosis in *L. bicolor,* and in *T. melanosporum,* an expanded family of NACHT-ANK proteins is characterized by a remarkable mechanism of diversification based on alternative splicing of codon-sized mini exons (Martin et al. 2008; Iotti et al. 2012). In this study, the species showing the highest number of NLRs is *Pi. indica,* which is an endophytic fungus (Zuccaro et al. 2011).

## Conclusion

Fungal NLR homologs have been shown, in two species, to be involved in nonself recognition and in the control of PCD (Saupe et al. 1995; Choi et al. 2012). We now report that filamentous fungi possess variable repertoires of NLR homologs, which show similarities and differences with NLRs in plant and animal lineages. This glimpse of fungal NLR diversity represents a further opportunity in comparative immunology for a more complete understanding of the build-up and evolution of immunity in eukaryotes. Although viridiplantae NLR repertoires are characterized by their considerable size (NLR repertoires with several hundreds of NB-LRR genes are not uncommon), mammalian NLR repertoires are fixed and reduced, most likely due of the presence of an adaptive immune system (Maekawa et al. 2011). In lower animals, NLR repertoires appear more extended, with again up to several hundred NLR genes in certain species (Lange et al. 2011; Hamada et al. 2013; Yuen et al. 2014). The fungal NLR repertoires similarly appear highly variable, but only exceptionally reach the complexity found in lower animals and land plants. The common occurrence of rapidly evolving ANK, TPR, and WD nonetheless may entail these repertoires with the plasticity required to cope with a complex and changing biotic environments. Animal and plant NLRs employ mechanistically distinct strategies for defense, in the form of intracellular PAMP detection in animals and ETI (effector-triggered immunity) in plants (Maekawa et al. 2011). It will be of interest to determine which strategies have been adopted in the fungal lineage. The involvement of NLR-like proteins in incompatibility, in which cell death is triggered by the recognition of an allelic variant of an endogenous protein by an NLR is compatible with a model of effector-triggered immunity (Paoletti and Saupe 2009; Choi et al. 2012; Bastiaans et al. 2014). Fungi possess extremely diverse lifestyles involving a variety of obligate or facultative biotic interactions; further functional studies are now required to understand which role these fungal NLR homologs play in the management of these diverse interorganismal interactions and which mechanistic strategies underlie NLR function in fungi.

## Supplementary Material

Supplementary files S1–S3, figures S1–S7, and tables S1–S4 are available at *Genome Biology and Evolution* online (http://www.gbe.oxfordjournals.org/).

Supplementary Data
