# Supplementary material for: Diversity and Variability of NOD-Like Receptors in Fungi
Source: Genome Biol Evol. 2014 Dec 8;6(12):3137–58. doi: 10.1093/gbe/evu251 (PMC4986451; doi:10.1093/gbe/evu251)
Supplement: Supplementary Data [file supp_6_12_3137__index.html]

Diversity and variability of NOD-like receptors in fungi — Diversity and Variability of NOD-Like Receptors in Fungi — Supplementary Data 

# Diversity and Variability of NOD-Like Receptors in Fungi

## Supplementary Data

files

**Files in this Data Supplement:**

- Supplementary Data - pdf file
- Supplementary Data - pdf file
- Supplementary Data - pdf file
- Supplementary Data - pdf file
- Supplementary Data - docx file
- Supplementary Data - xls file
