# Supplementary material for: Diversity and Variability of NOD-Like Receptors in Fungi
Source: Genome Biol Evol. 2014 Dec 8;6(12):3137–58. doi: 10.1093/gbe/evu251 (PMC4986451; doi:10.1093/gbe/evu251)

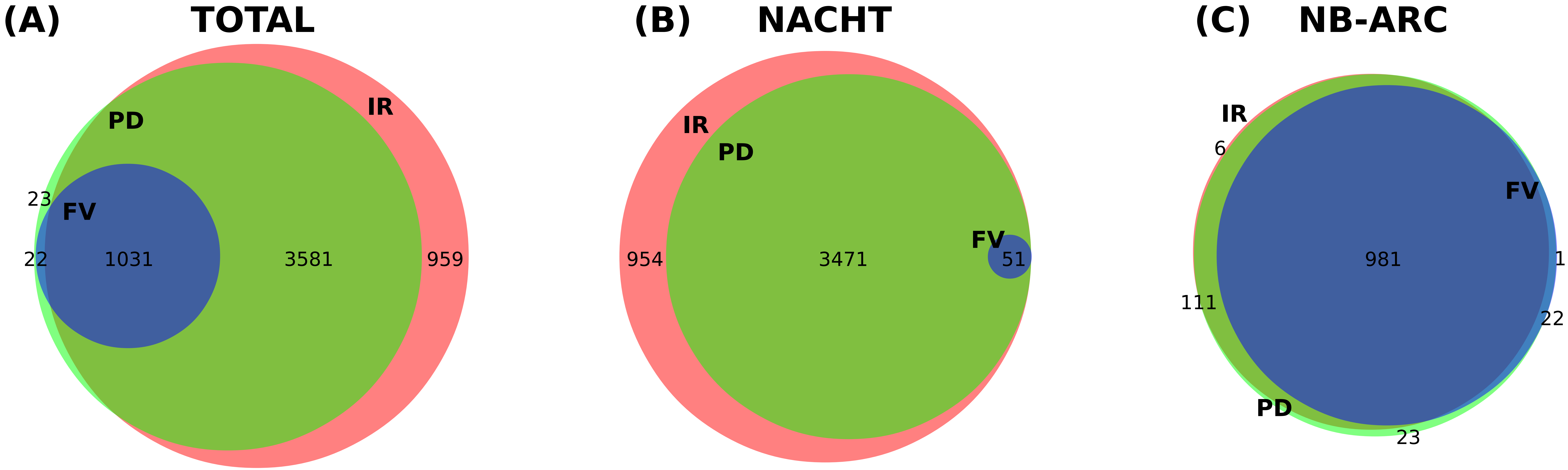

(A)
TOTAL
IR
PD
FV
23
22
1031
3581
959
(B)
NACHT
IR
PD
FV
954
3471
51
(C)
NB-ARC
IR
6
FV
981
1
111
22
PD
23

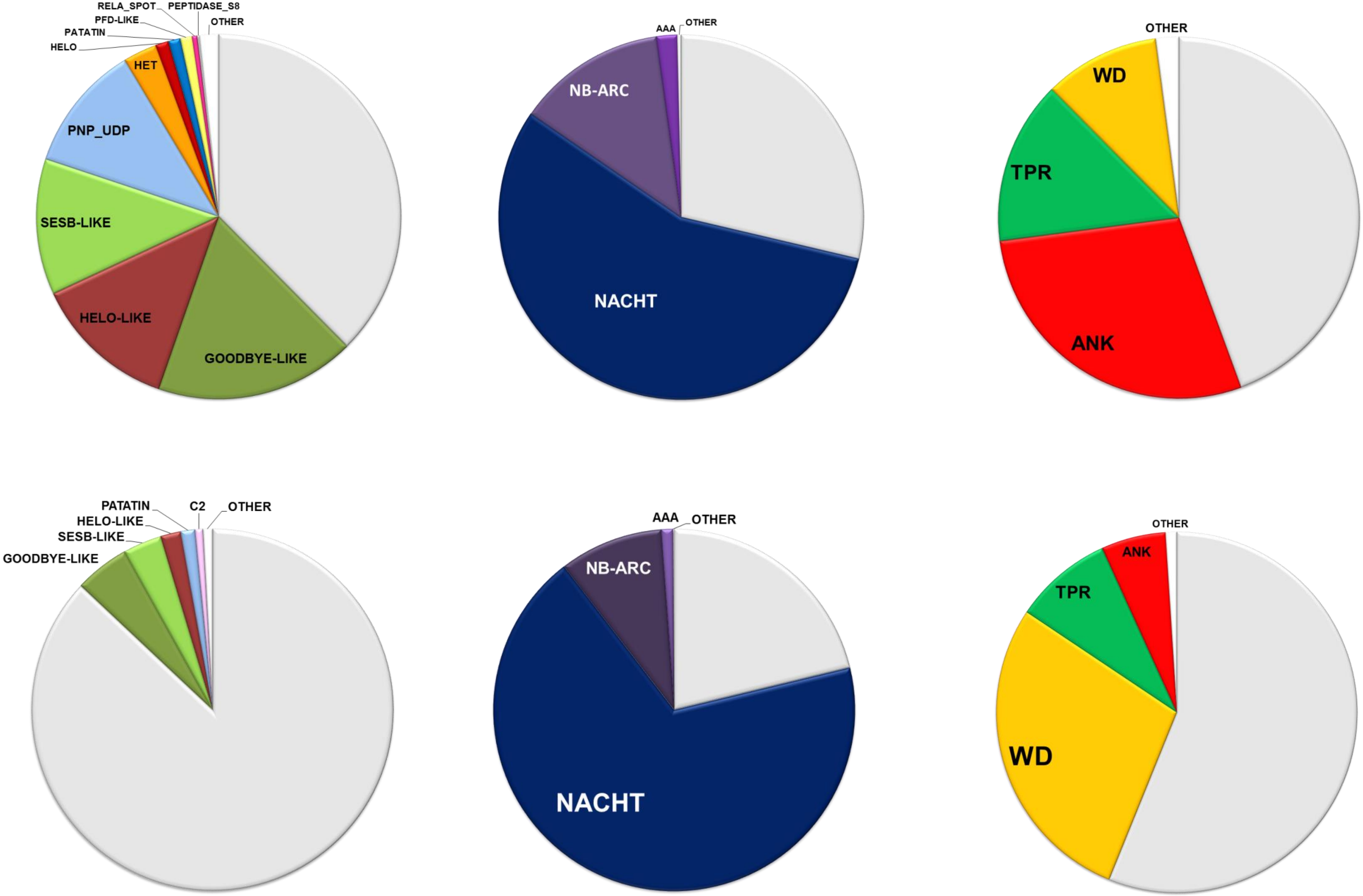
RELA_SPOT
PEPTIDASE_S8
PFD-LIKE
OTHER
PATATIN
HELO
HET
PNP_UDP
SESB-LIKE
HELO-LIKE
GOODBYE-LIKE
AAA
OTHER
NB-ARC
NACHT
OTHER
WD
TPR
ANK
PATATIN
C2
OTHER
HELO-LIKE
SESB-LIKE
GOODBYE-LIKE
AAA
OTHER
NB-ARC
NACHT
OTHER
ANK
TPR
WD

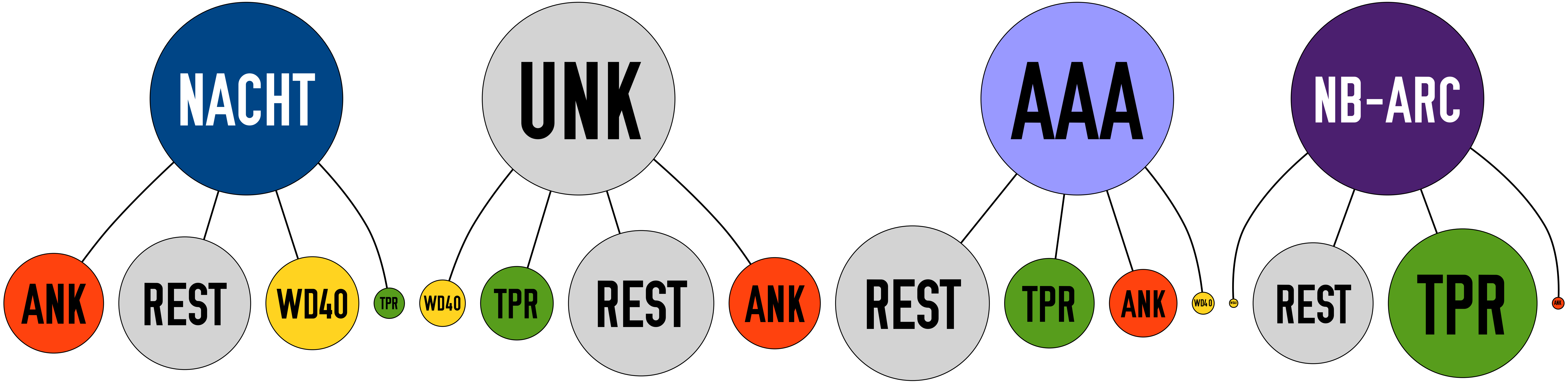
NACHT
ANK
REST
WD40
TPR
UNK
WD40
TPR
REST
ANK
AAA
REST
TPR
ANK
WD40
NB-ARC
REST
TPR

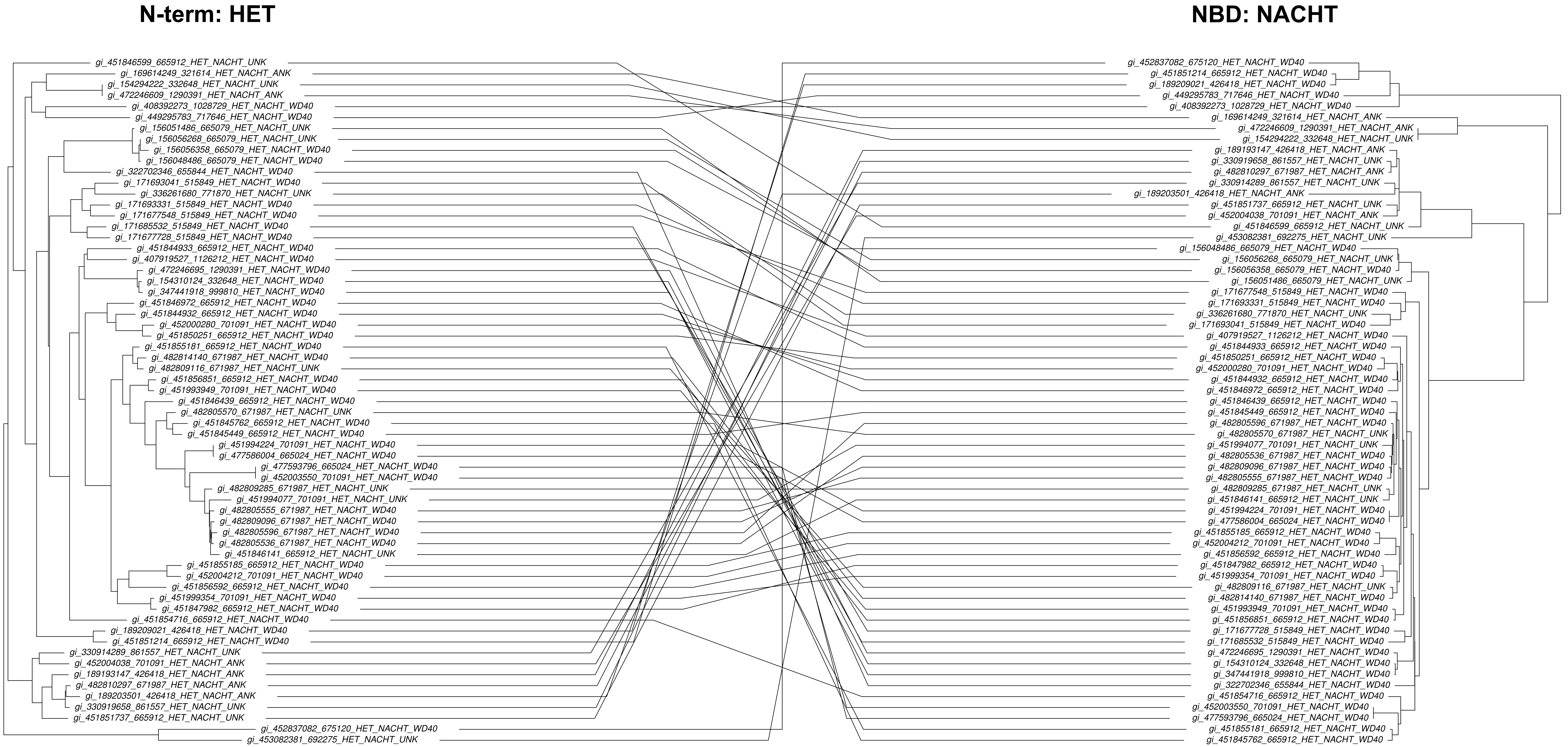
N-term: HET
NBD: NACHT
gi_451846599_665912_HET_NACHT_UNK
gi_169614249_321614_HET_NACHT_ANK
gi_154294222_332648_HET_NACHT_UNK
gi_472246609_1290391_HET_NACHT_ANK
gi_408392273_1028729_HET_NACHT_WD40
gi_449295783_717646_HET_NACHT_WD40
gi_156051486_665079_HET_NACHT_UNK
gi_156056268_665079_HET_NACHT_UNK
gi_156056358_665079_HET_NACHT_WD40
gi_156048486_665079_HET_NACHT_WD40
gi_322702346_655844_HET_NACHT_WD40
gi_171693041_515849_HET_NACHT_WD40
gi_336261680_771870_HET_NACHT_UNK
gi_171693331_515849_HET_NACHT_WD40
gi_171677548_515849_HET_NACHT_WD40
gi_171685532_515849_HET_NACHT_WD40
gi_171677728_515849_HET_NACHT_WD40
gi_451844933_665912_HET_NACHT_WD40
gi_407919527_1126212_HET_NACHT_WD40
gi_472246695_1290391_HET_NACHT_WD40
gi_154310124_332648_HET_NACHT_WD40
gi_347441918_999810_HET_NACHT_WD40
gi_451846972_665912_HET_NACHT_WD40
gi_451844932_665912_HET_NACHT_WD40
gi_452000280_701091_HET_NACHT_WD40
gi_451850251_665912_HET_NACHT_WD40
gi_451855181_665912_HET_NACHT_WD40
gi_482814140_671987_HET_NACHT_WD40
gi_482809116_671987_HET_NACHT_UNK
gi_451856851_665912_HET_NACHT_WD40
gi_451993949_701091_HET_NACHT_WD40
gi_451846439_665912_HET_NACHT_WD40
gi_482805570_671987_HET_NACHT_UNK
gi_451845762_665912_HET_NACHT_WD40
gi_451845449_665912_HET_NACHT_WD40
gi_451994224_701091_HET_NACHT_WD40
gi_477586004_665024_HET_NACHT_WD40
gi_477593796_665024_HET_NACHT_WD40
gi_452003550_701091_HET_NACHT_WD40
gi_482809285_671987_HET_NACHT_UNK
gi_451994077_701091_HET_NACHT_UNK
gi_482805555_671987_HET_NACHT_WD40
gi_482809096_671987_HET_NACHT_WD40
gi_482805596_671987_HET_NACHT_WD40
gi_482805536_671987_HET_NACHT_WD40
gi_451846141_665912_HET_NACHT_UNK
gi_451855185_665912_HET_NACHT_WD40
gi_452004212_701091_HET_NACHT_WD40
gi_451856592_665912_HET_NACHT_WD40
gi_451999354_701091_HET_NACHT_WD40
gi_451847982_665912_HET_NACHT_WD40
gi_451854716_665912_HET_NACHT_WD40
gi_189209021_426418_HET_NACHT_WD40
gi_451851214_665912_HET_NACHT_WD40
gi_330914289_861557_HET_NACHT_UNK
gi_452004038_701091_HET_NACHT_ANK
gi_189193147_426418_HET_NACHT_ANK
gi_482810297_671987_HET_NACHT_ANK
gi_189203501_426418_HET_NACHT_ANK
gi_330919658_861557_HET_NACHT_UNK
gi_451851737_665912_HET_NACHT_UNK
gi_452837082_675120_HET_NACHT_WD40
gi_453082381_692275_HET_NACHT_UNK
gi_452837082_675120_HET_NACHT_WD40
gi_451851214_665912_HET_NACHT_WD40
gi_189209021_426418_HET_NACHT_WD40
gi_449295783_717646_HET_NACHT_WD40
gi_408392273_1028729_HET_NACHT_WD40
gi_169614249_321614_HET_NACHT_ANK
gi_472246609_1290391_HET_NACHT_ANK
gi_154294222_332648_HET_NACHT_UNK
gi_189193147_426418_HET_NACHT_ANK
gi_330919658_861557_HET_NACHT_UNK
gi_482810297_671987_HET_NACHT_ANK
gi_330914289_861557_HET_NACHT_UNK
gi_189203501_426418_HET_NACHT_ANK
gi_451851737_665912_HET_NACHT_UNK
gi_452004038_701091_HET_NACHT_ANK
gi_451846599_665912_HET_NACHT_UNK
gi_453082381_692275_HET_NACHT_UNK
gi_156048486_665079_HET_NACHT_WD40
gi_156056268_665079_HET_NACHT_UNK
gi_156056358_665079_HET_NACHT_WD40
gi_156051486_665079_HET_NACHT_UNK
gi_171677548_515849_HET_NACHT_WD40
gi_171693331_515849_HET_NACHT_WD40
gi_336261680_771870_HET_NACHT_UNK
gi_171693041_515849_HET_NACHT_WD40
gi_407919527_1126212_HET_NACHT_WD40
gi_451844933_665912_HET_NACHT_WD40
gi_451850251_665912_HET_NACHT_WD40
gi_452000280_701091_HET_NACHT_WD40
gi_451844932_665912_HET_NACHT_WD40
gi_451846972_665912_HET_NACHT_WD40
gi_451846439_665912_HET_NACHT_WD40
gi_451845449_665912_HET_NACHT_WD40
gi_482805596_671987_HET_NACHT_WD40
gi_482805570_671987_HET_NACHT_UNK
gi_451994077_701091_HET_NACHT_UNK
gi_482805536_671987_HET_NACHT_WD40
gi_482809096_671987_HET_NACHT_WD40
gi_482805555_671987_HET_NACHT_WD40
gi_482809285_671987_HET_NACHT_UNK
gi_451846141_665912_HET_NACHT_UNK
gi_451994224_701091_HET_NACHT_WD40
gi_477586004_665024_HET_NACHT_WD40
gi_451855185_665912_HET_NACHT_WD40
gi_452004212_701091_HET_NACHT_WD40
gi_451856592_665912_HET_NACHT_WD40
gi_451847982_665912_HET_NACHT_WD40
gi_451999354_701091_HET_NACHT_WD40
gi_482809116_671987_HET_NACHT_UNK
gi_482814140_671987_HET_NACHT_WD40
gi_451993949_701091_HET_NACHT_WD40
gi_451856851_665912_HET_NACHT_WD40
gi_171677728_515849_HET_NACHT_WD40
gi_171685532_515849_HET_NACHT_WD40
gi_472246695_1290391_HET_NACHT_WD40
gi_154310124_332648_HET_NACHT_WD40
gi_347441918_999810_HET_NACHT_WD40
gi_322702346_655844_HET_NACHT_WD40
gi_451854716_665912_HET_NACHT_WD40
gi_452003550_701091_HET_NACHT_WD40
gi_477593796_665024_HET_NACHT_WD40
gi_451855181_665912_HET_NACHT_WD40
gi_451845762_665912_HET_NACHT_WD40

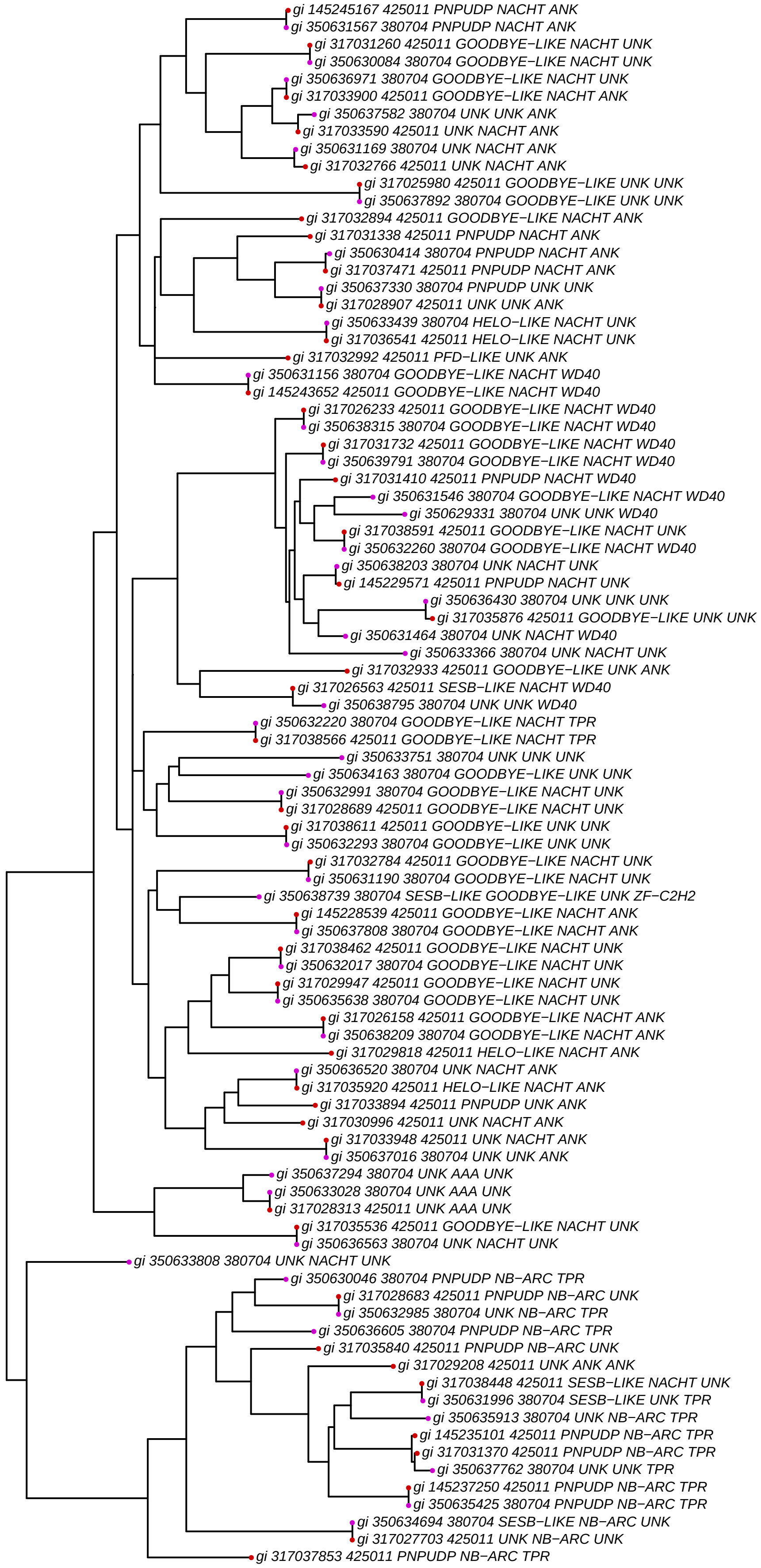
gi 145245167 425011 PNPUDP NACHT ANK
gi 350631567 380704 PNPUDP NACHT ANK
gi 317031260 425011 GOODBYE−LIKE NACHT UNK
gi 350630084 380704 GOODBYE−LIKE NACHT UNK
gi 350636971 380704 GOODBYE−LIKE NACHT UNK
gi 317033900 425011 GOODBYE−LIKE NACHT ANK
gi 350637582 380704 UNK UNK ANK
gi 317033590 425011 UNK NACHT ANK
gi 350631169 380704 UNK NACHT ANK
gi 317032766 425011 UNK NACHT ANK
gi 317025980 425011 GOODBYE−LIKE UNK UNK
gi 350637892 380704 GOODBYE−LIKE UNK UNK
gi 317032894 425011 GOODBYE−LIKE NACHT ANK
gi 317031338 425011 PNPUDP NACHT ANK
gi 350630414 380704 PNPUDP NACHT ANK
gi 317037471 425011 PNPUDP NACHT ANK
gi 350637330 380704 PNPUDP UNK UNK
gi 317028907 425011 UNK UNK ANK
gi 350633439 380704 HELO−LIKE NACHT UNK
gi 317036541 425011 HELO−LIKE NACHT UNK
gi 317032992 425011 PFD−LIKE UNK ANK
gi 350631156 380704 GOODBYE−LIKE NACHT WD40
gi 145243652 425011 GOODBYE−LIKE NACHT WD40
gi 317026233 425011 GOODBYE−LIKE NACHT WD40
gi 350638315 380704 GOODBYE−LIKE NACHT WD40
gi 317031732 425011 GOODBYE−LIKE NACHT WD40
gi 350639791 380704 GOODBYE−LIKE NACHT WD40
gi 317031410 425011 PNPUDP NACHT WD40
gi 350631546 380704 GOODBYE−LIKE NACHT WD40
gi 350629331 380704 UNK UNK WD40
gi 317038591 425011 GOODBYE−LIKE NACHT UNK
gi 350632260 380704 GOODBYE−LIKE NACHT WD40
gi 350638203 380704 UNK NACHT UNK
gi 145229571 425011 PNPUDP NACHT UNK
gi 350636430 380704 UNK UNK UNK
gi 317035876 425011 GOODBYE−LIKE UNK UNK
gi 350631464 380704 UNK NACHT WD40
gi 350633366 380704 UNK NACHT UNK
gi 317032933 425011 GOODBYE−LIKE UNK ANK
gi 317026563 425011 SESB−LIKE NACHT WD40
gi 350638795 380704 UNK UNK WD40
gi 350632220 380704 GOODBYE−LIKE NACHT TPR
gi 317038566 425011 GOODBYE−LIKE NACHT TPR
gi 350633751 380704 UNK UNK UNK
gi 350634163 380704 GOODBYE−LIKE UNK UNK
gi 350632991 380704 GOODBYE−LIKE NACHT UNK
gi 317028689 425011 GOODBYE−LIKE NACHT UNK
gi 317038611 425011 GOODBYE−LIKE UNK UNK
gi 350632293 380704 GOODBYE−LIKE UNK UNK
gi 317032784 425011 GOODBYE−LIKE NACHT UNK
gi 350631190 380704 GOODBYE−LIKE NACHT UNK
gi 350638739 380704 SESB−LIKE GOODBYE−LIKE UNK ZF−C2H2
gi 145228539 425011 GOODBYE−LIKE NACHT ANK
gi 350637808 380704 GOODBYE−LIKE NACHT ANK
gi 317038462 425011 GOODBYE−LIKE NACHT UNK
gi 350632017 380704 GOODBYE−LIKE NACHT UNK
gi 317029947 425011 GOODBYE−LIKE NACHT UNK
gi 350635638 380704 GOODBYE−LIKE NACHT UNK
gi 317026158 425011 GOODBYE−LIKE NACHT ANK
gi 350638209 380704 GOODBYE−LIKE NACHT ANK
gi 317029818 425011 HELO−LIKE NACHT ANK
gi 350636520 380704 UNK NACHT ANK
gi 317035920 425011 HELO−LIKE NACHT ANK
gi 317033894 425011 PNPUDP UNK ANK
gi 317030996 425011 UNK NACHT ANK
gi 317033948 425011 UNK NACHT ANK
gi 350637016 380704 UNK UNK ANK
gi 350637294 380704 UNK AAA UNK
gi 350633028 380704 UNK AAA UNK
gi 317028313 425011 UNK AAA UNK
gi 317035536 425011 GOODBYE−LIKE NACHT UNK
gi 350636563 380704 UNK NACHT UNK
gi 350633808 380704 UNK NACHT UNK
gi 350630046 380704 PNPUDP NB−ARC TPR
gi 317028683 425011 PNPUDP NB−ARC UNK
gi 350632985 380704 UNK NB−ARC TPR
gi 350636605 380704 PNPUDP NB−ARC TPR
gi 317035840 425011 PNPUDP NB−ARC UNK
gi 317029208 425011 UNK ANK ANK
gi 317038448 425011 SESB−LIKE NACHT UNK
gi 350631996 380704 SESB−LIKE UNK TPR
gi 350635913 380704 UNK NB−ARC TPR
gi 145235101 425011 PNPUDP NB−ARC TPR
gi 317031370 425011 PNPUDP NB−ARC TPR
gi 350637762 380704 UNK UNK TPR
gi 145237250 425011 PNPUDP NB−ARC TPR
gi 350635425 380704 PNPUDP NB−ARC TPR
gi 350634694 380704 SESB−LIKE NB−ARC UNK
gi 317027703 425011 UNK NB−ARC UNK
gi 317037853 425011 PNPUDP NB−ARC TPR

AGABI 597362, 936046

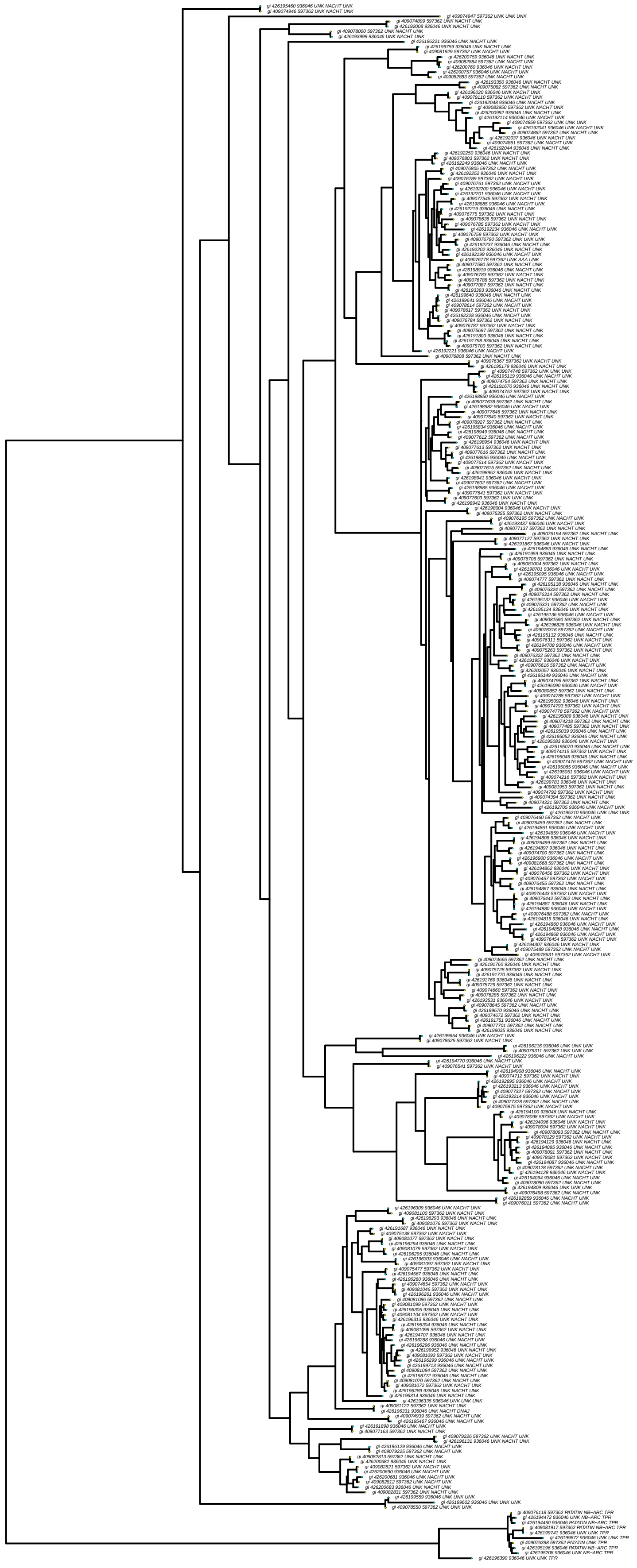

AJECA 339724, 447093, 544711, 544712

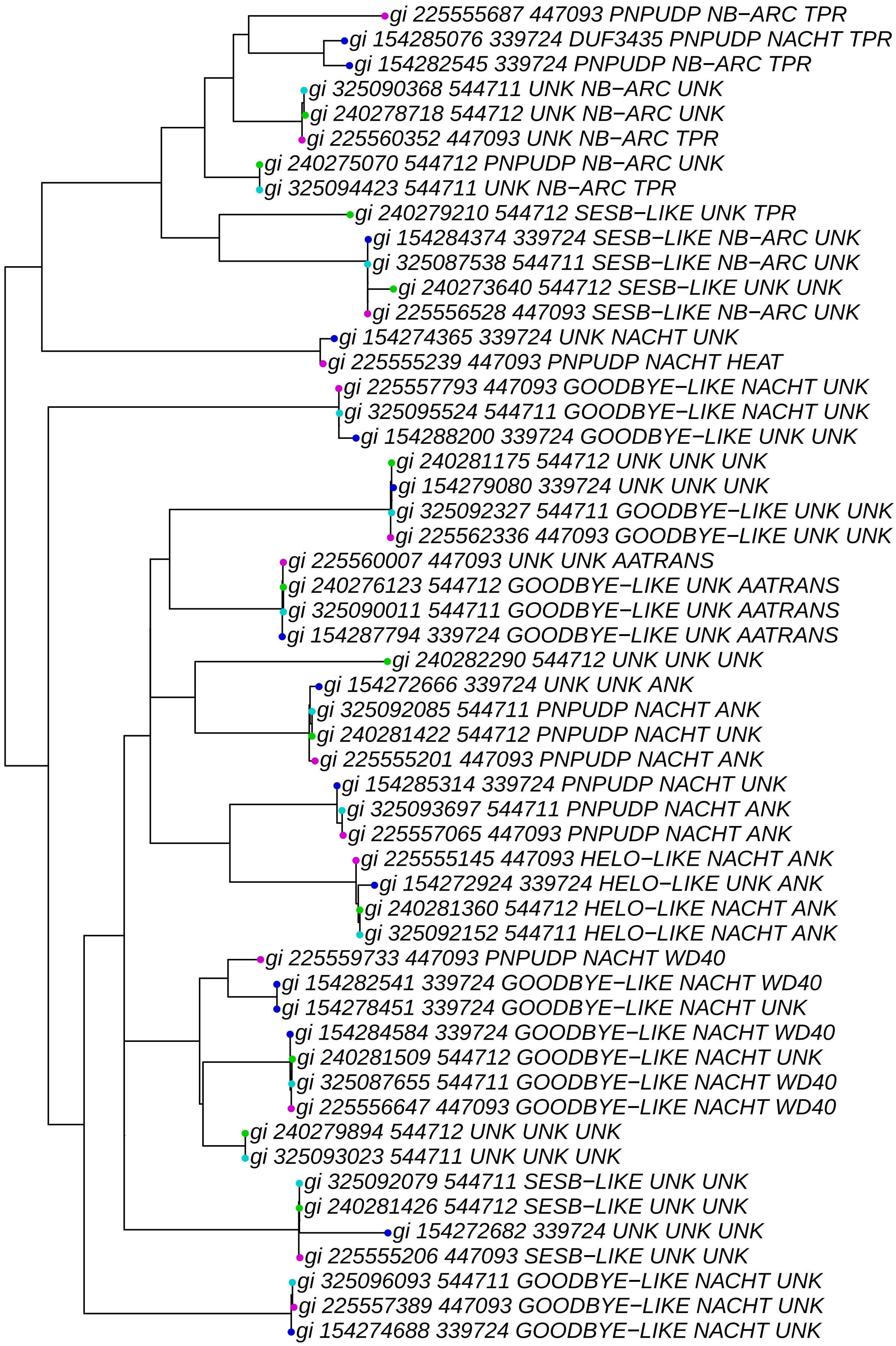

AJEDE 559297, 559298, 653446

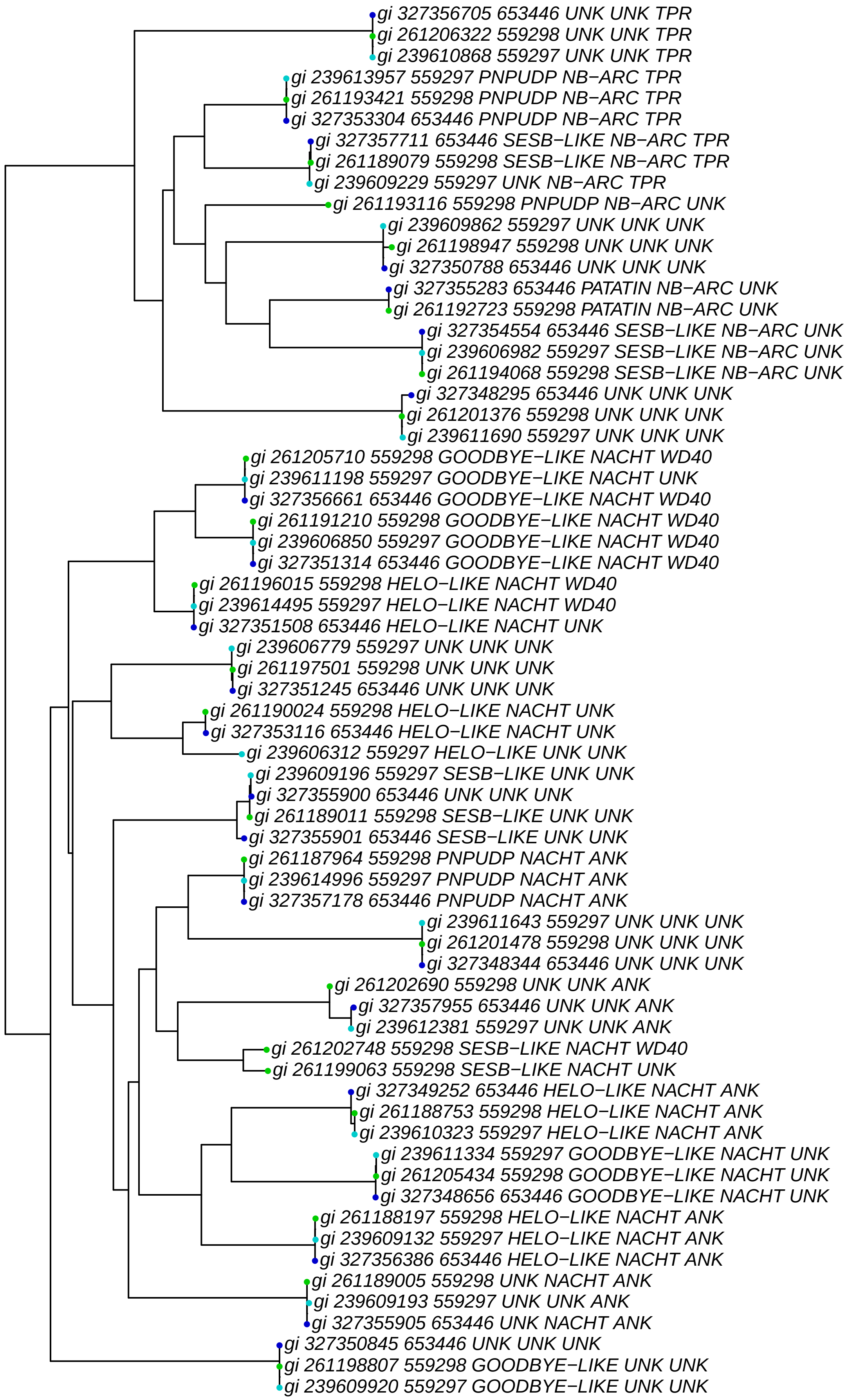

ASPFU 330879, 451804

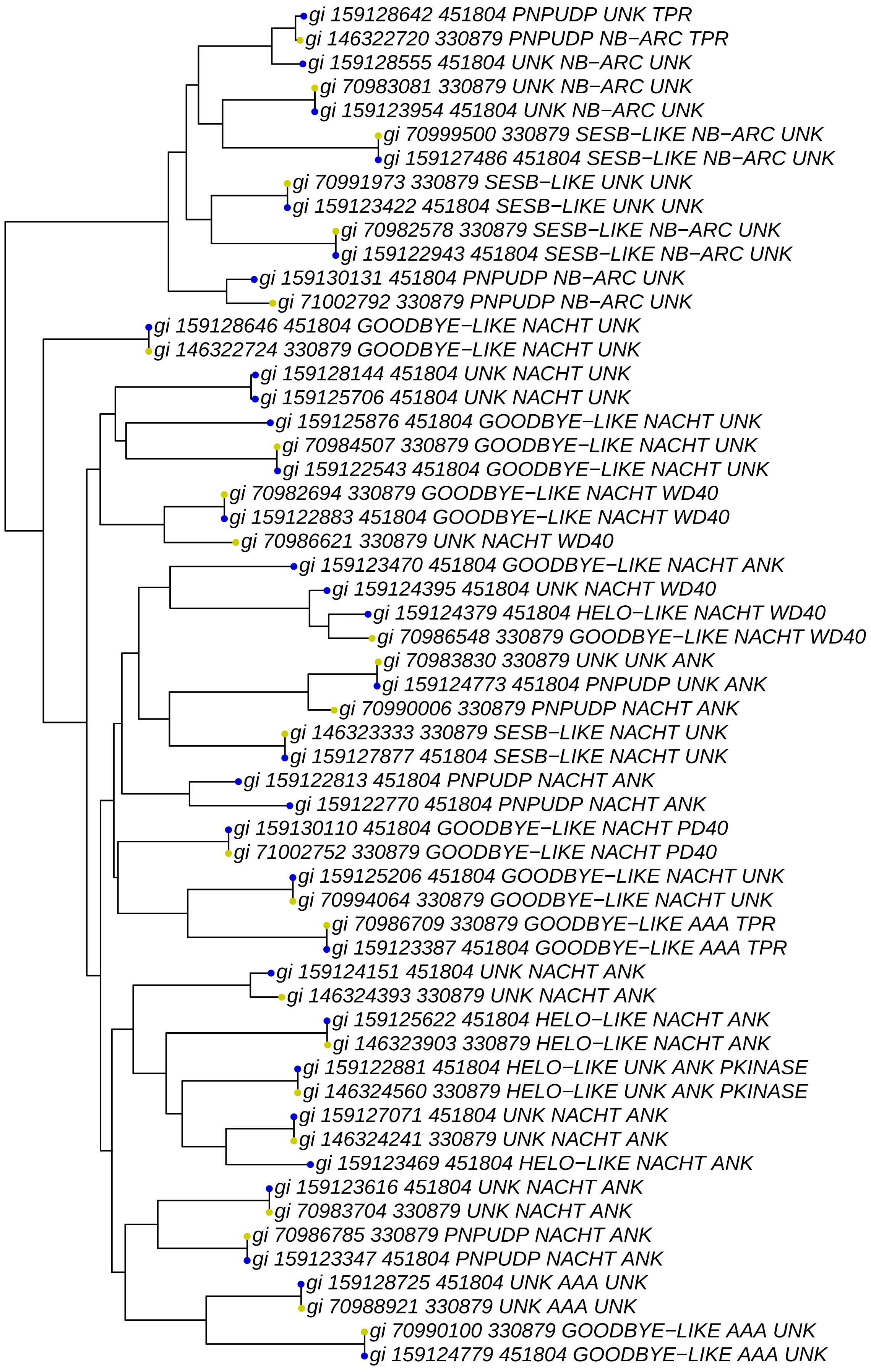

ASPNI 380704, 425011

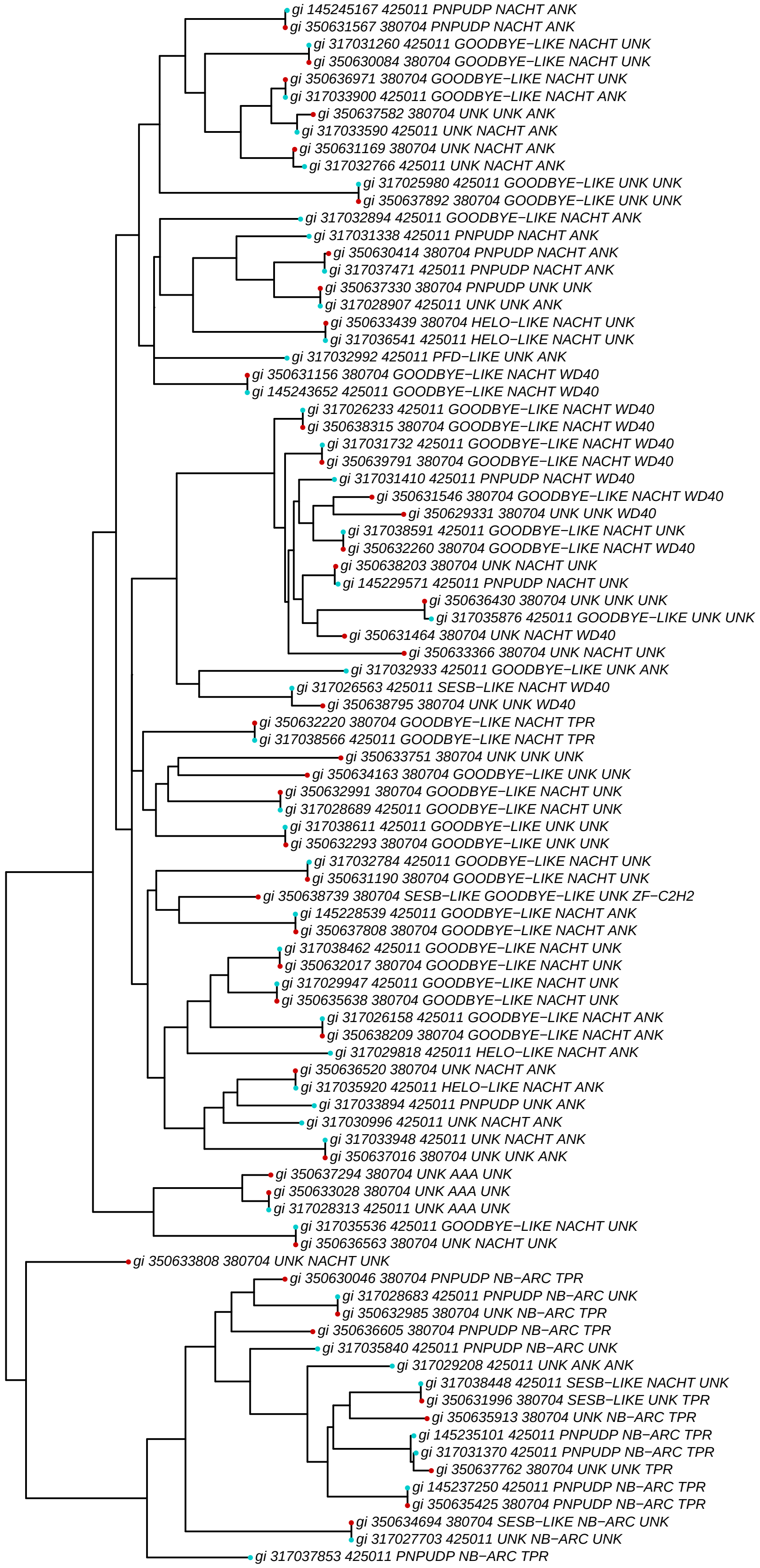

BIPMA 665024, 701091

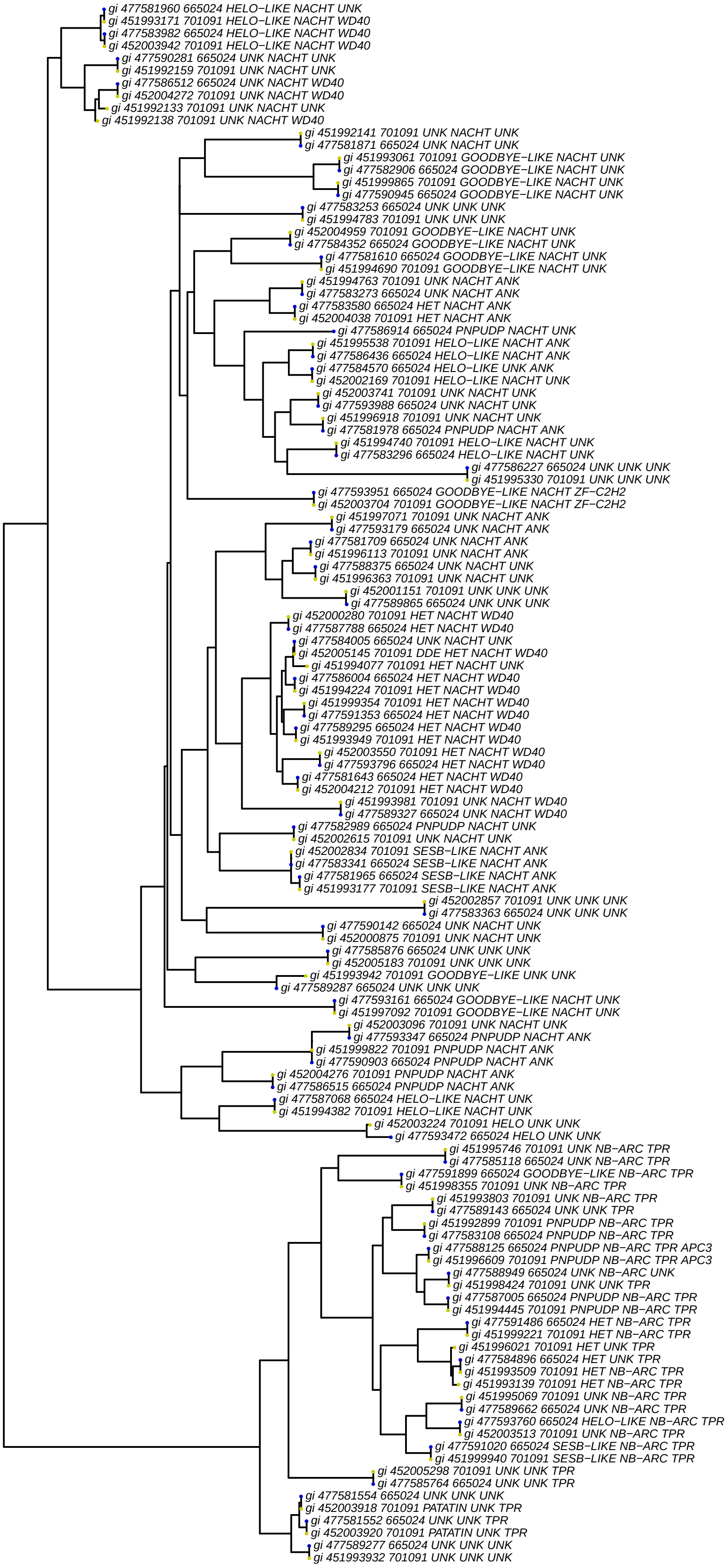

BOTFU 1290391, 332648, 999810

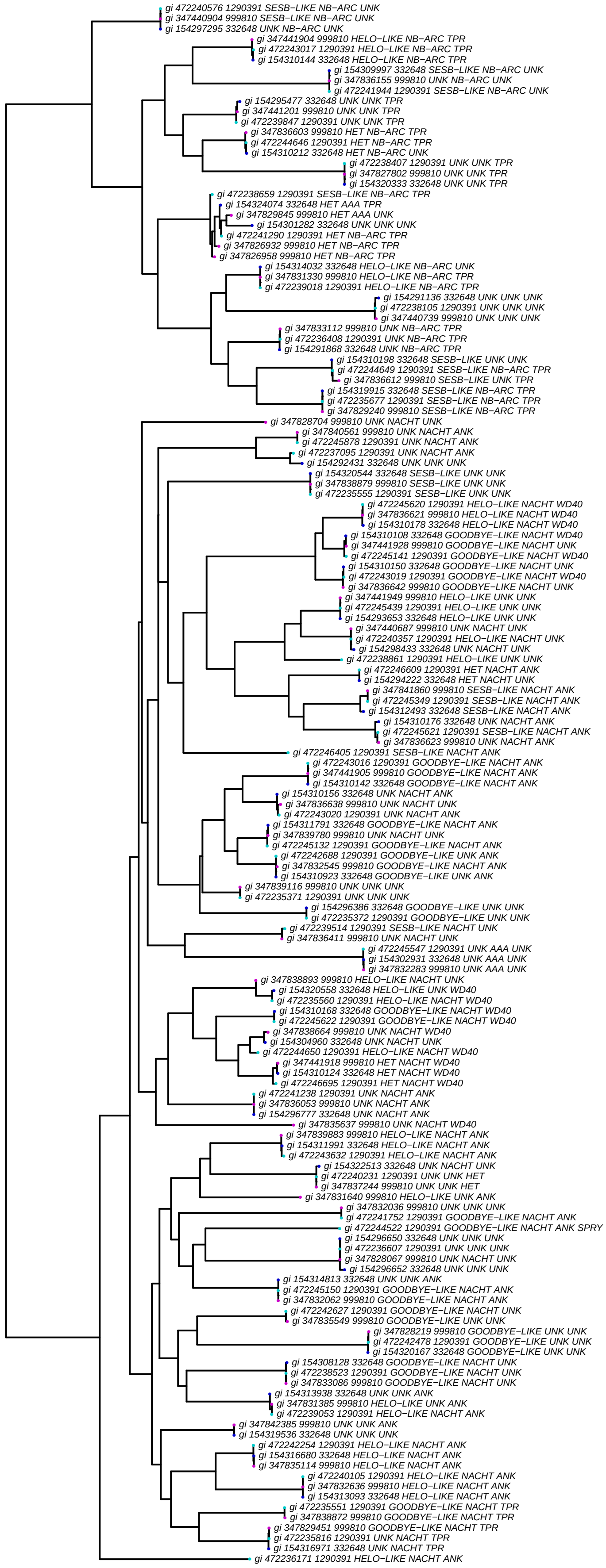

COCPO 222929, 443226

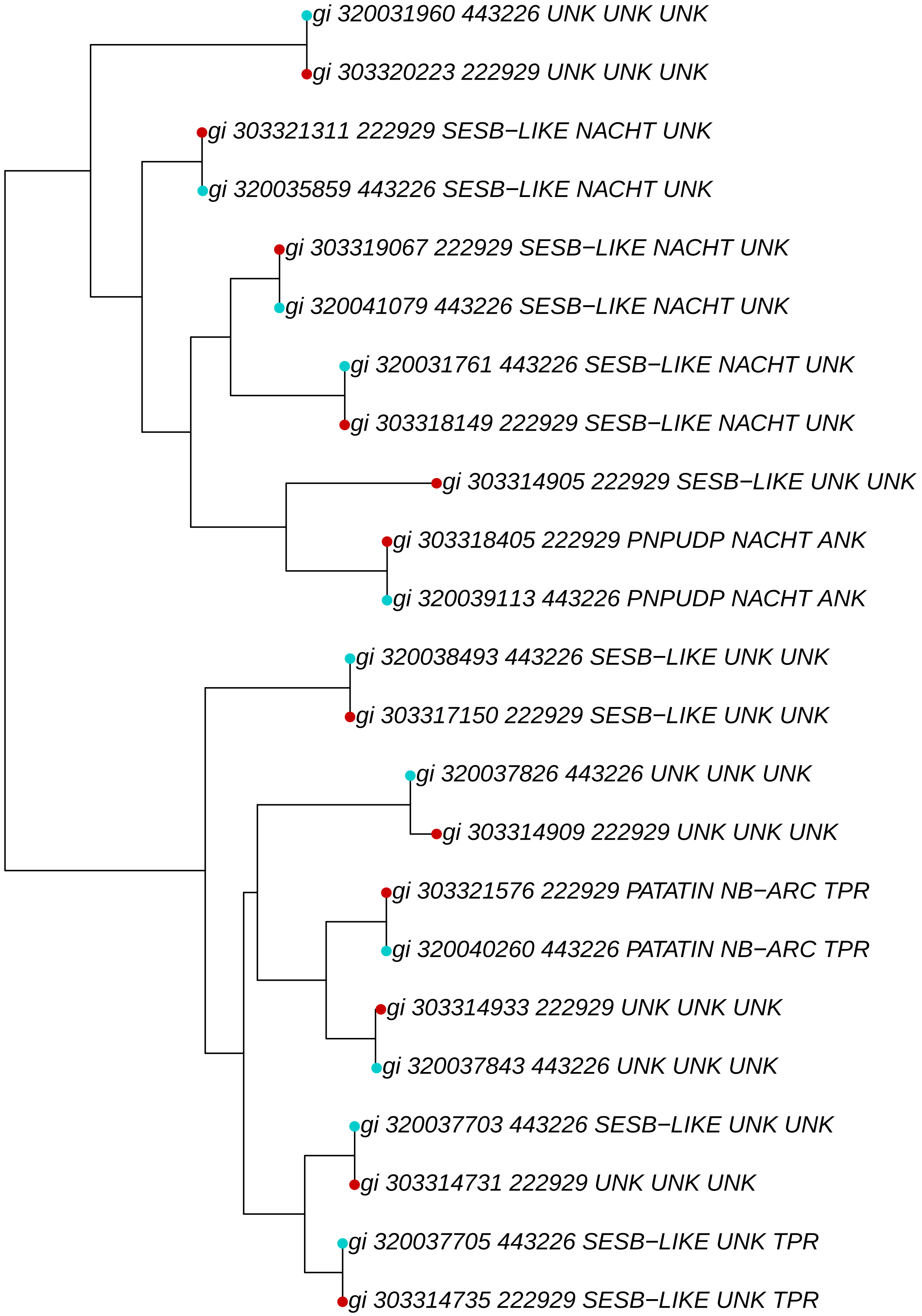

FUSOX 1229664, 1229665, 660025

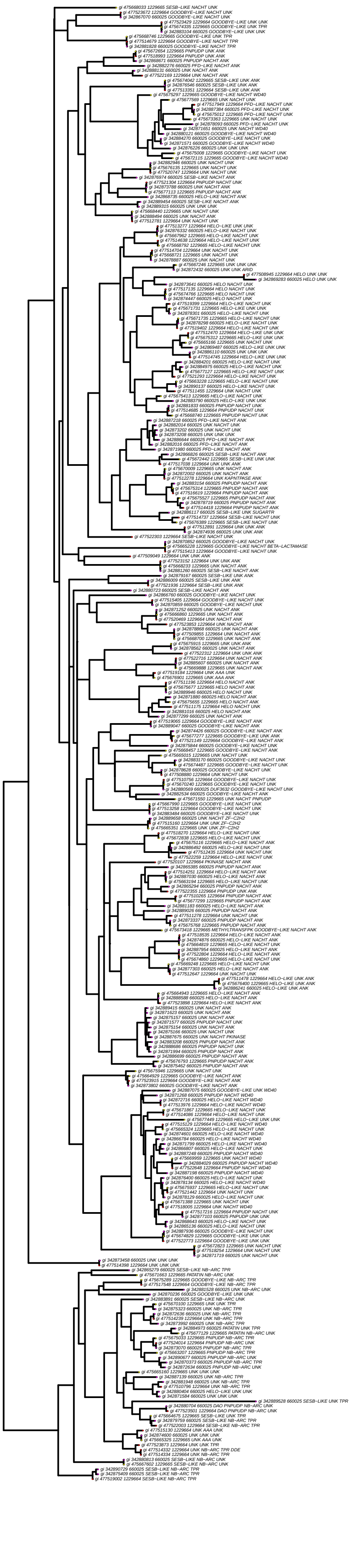

MAGOR 1143189, 1143193, 242507

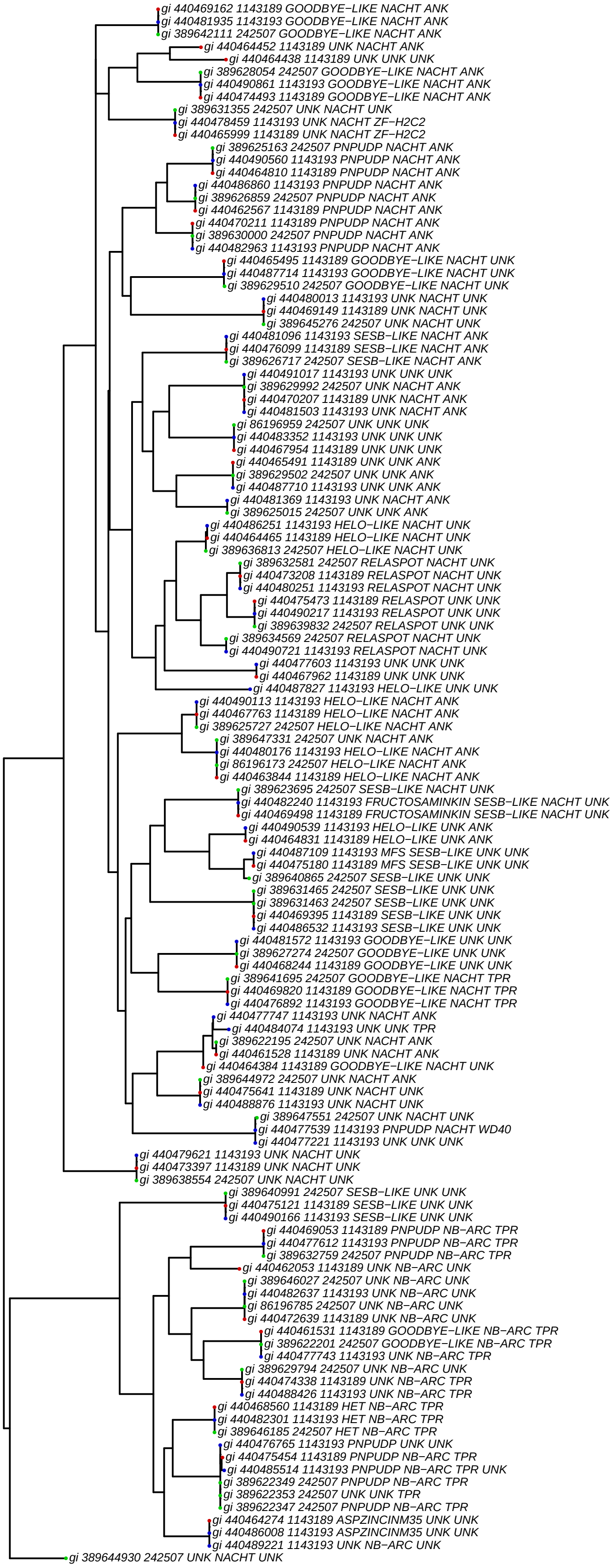

NEUTE 510951, 510952

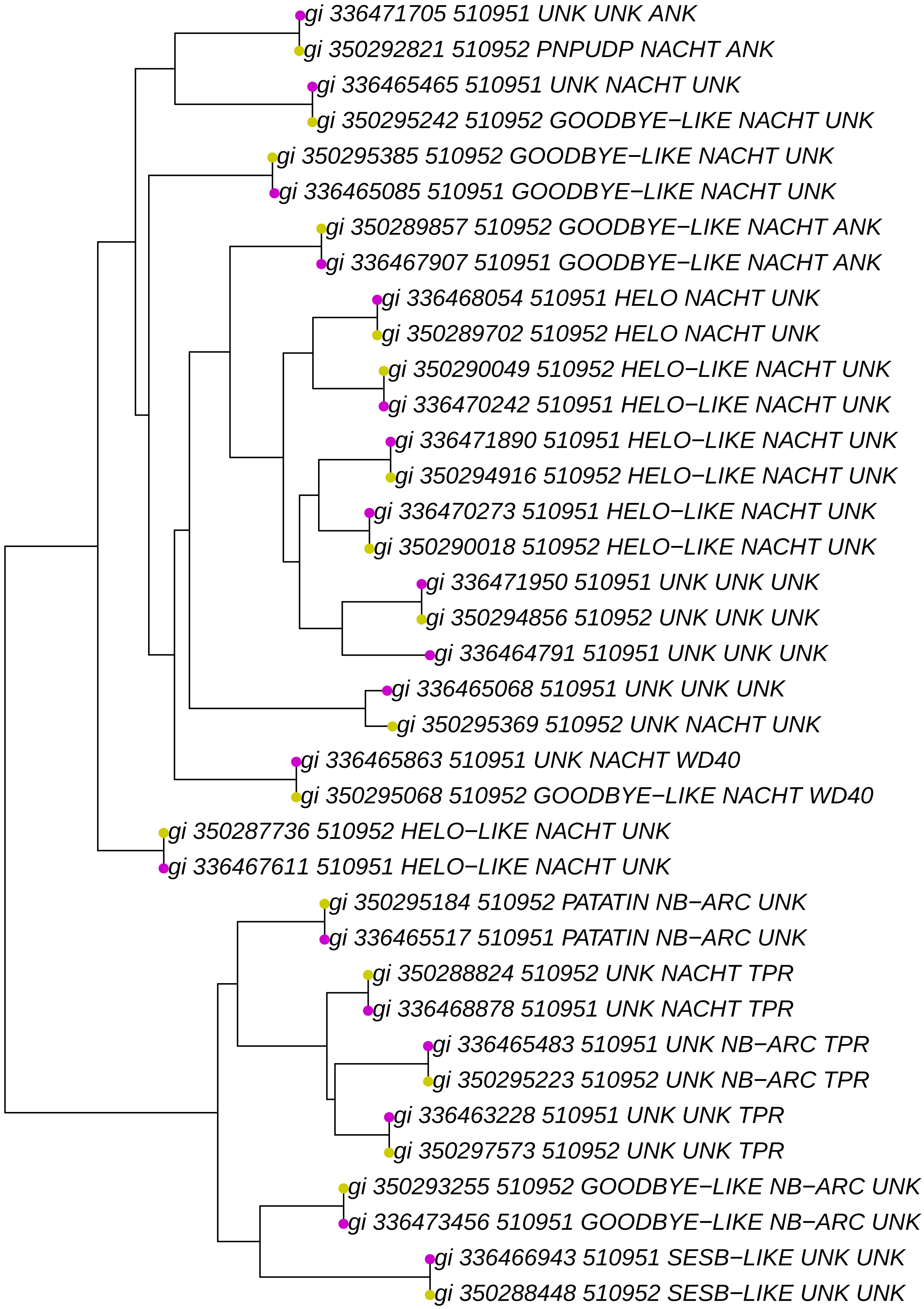

PARBR 482561, 502780

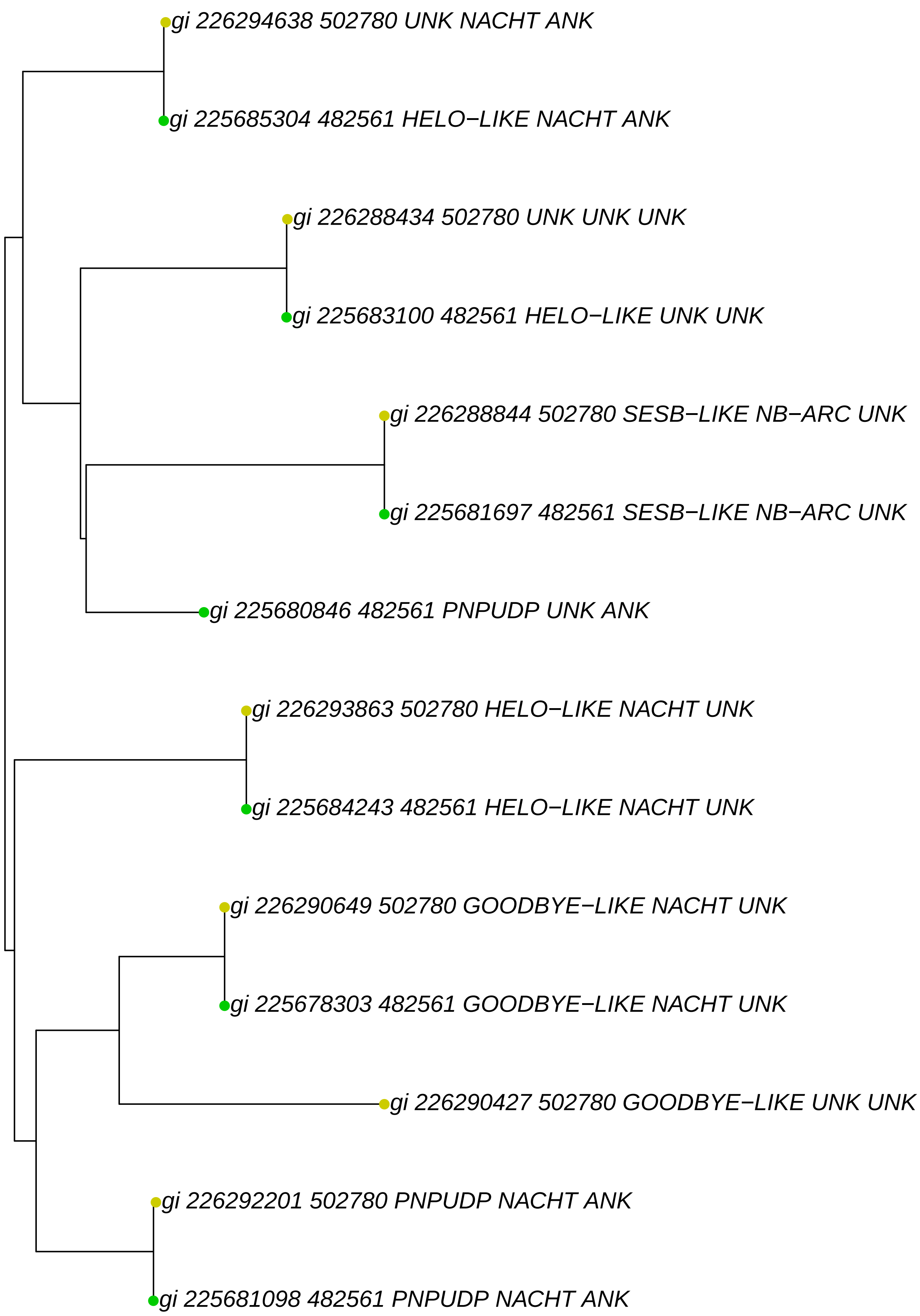

PENDI 1170229, 1170230

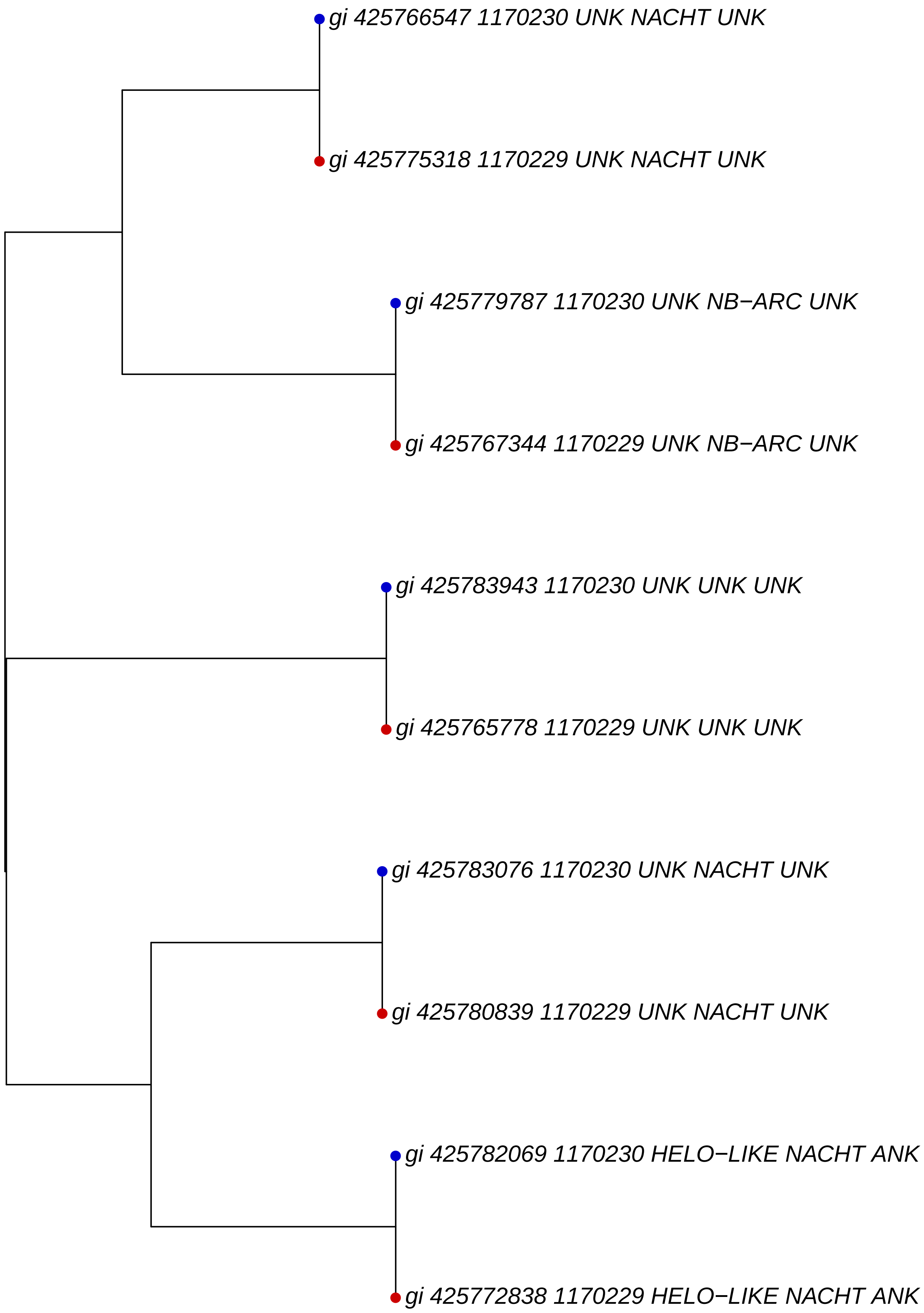

RHISO 1108050, 983506

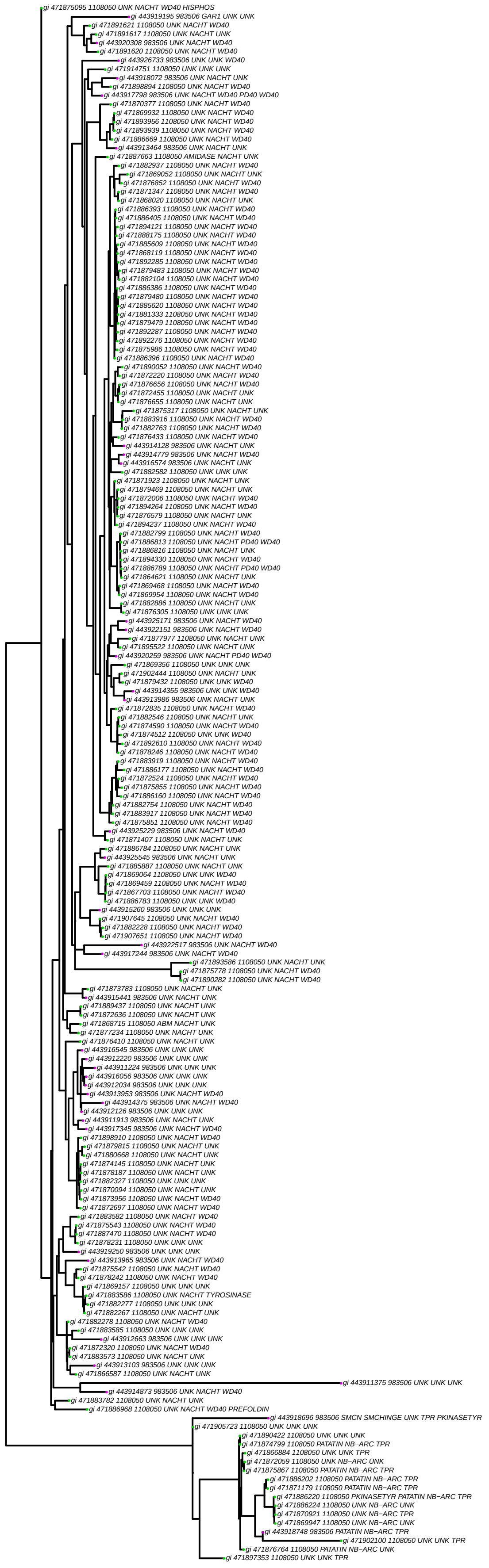

SERLA 578457, 936435

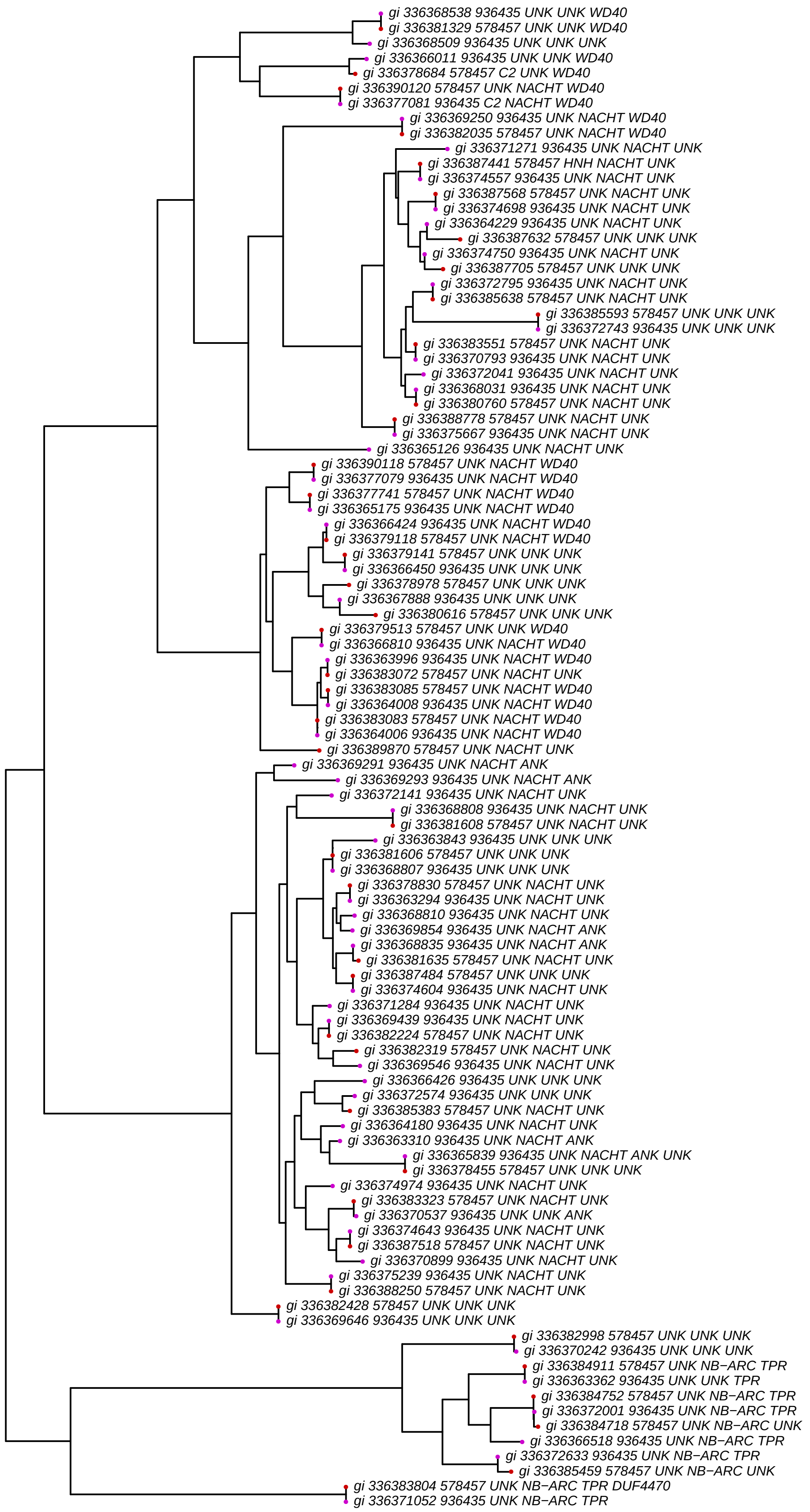

A

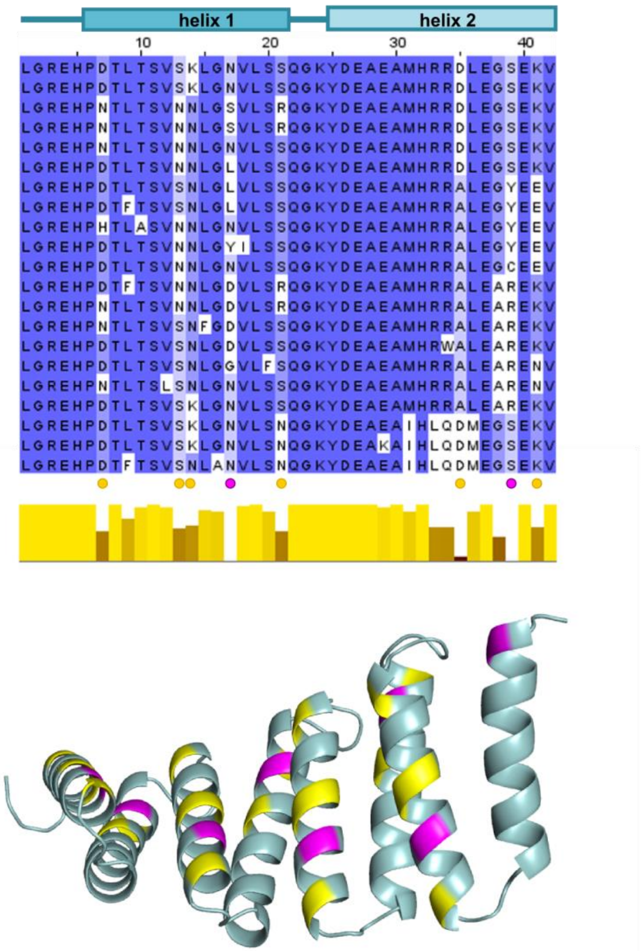

B

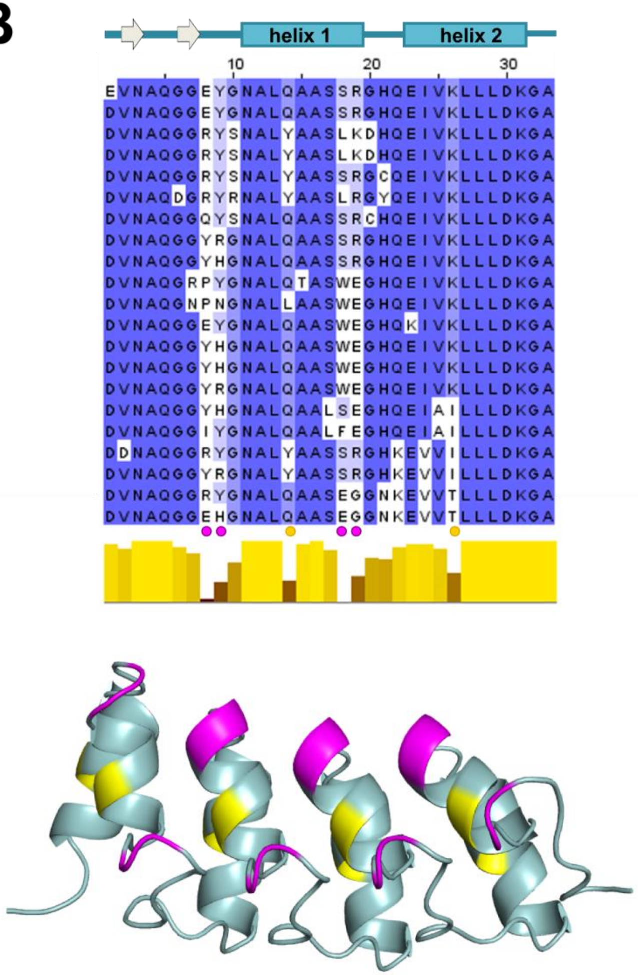

Supplement: Supplementary Data [file supp_evu251_supplementary_figures.pdf]
