# Supplementary material for: Diversity and Variability of NOD-Like Receptors in Fungi
Source: Genome Biol Evol. 2014 Dec 8;6(12):3137–58. doi: 10.1093/gbe/evu251 (PMC4986451; doi:10.1093/gbe/evu251)
Supplement: Supplementary Data [file supp_evu251_supplementary_file_S1_Query_sets.pdf]

#IR NACHT

```
>CAL30199__Podospora_anserina_nwd2__NACHT_WD40_Het-s_PFD_73_218
FWLNGMAGTGKSTISRTVAKSFADDGILGASFFFKRGEGDRGKATLFFPTIASQLVRKIP
ALEAFVREAINNNPDVARKALRDQFEKLILQPLDRIHHAIAVIVVDALDECDGDNDVKVI
ISLLSQAKELRSPGLRIFITSRPELP
>XP_390777_Fusarium_graminearum_FG10601__NACHT_WD40_Het-s_PFD_82_233
LWIKGDAGKGKTMLICAIIENLEHDDRNHVSYFFCQAGVPTTDNATSVLRGLVYGLVRYH
PHNATVLSHVRKEFDSISERMFEGPNLWQIIRKILVTILKDPLMNHTVFVIDALDECEGN
QAELLKFIVETNTQLGIKWLVSSRKDPVIENR
>XP_388320__Fusarium_graminearum_FG08144__NACHT_WD40_Het-s_PFD_93_266
LLWIRGDPGKGKTMLLCGIIDELRPSTRLENSESHISLSYFFCQATNSALNNATAVIRGL
IYLLVVQQPCLLSHLRANIHWDSRVAIEDLFRKIVADPVLEEAYLIVDALDECREDLEFL
LEMLSSPIPRIKWIVSSRNRVEIKEALKQSSSRLALSLELNEESVSQAVRHFID
>EEU47147_Nectria_haematocca_EEU47147__NACHT_WD40__Het-s_PFD_79_234
RLLWIKGDSGKGKTMLVCSIINWLRTNEKATLAYFFCQKSDENLSSSAAVLRGLVYMLAR
EHDTIRELVQDEYKNAGRSLFWDRNAWYALKEILNTALQREGLGDIVIIVDALDECVTGI
HQLLDLVIRLSSKTSRVRWIVSSRNEPEIEQHFDEF
>EEU38120_Nectria_haematocca_EEU38120__NACHT_WD40_Het-s_PFD_79_253
LLWIKGDPGKGKTMLFCGLLDELGPTTRLVDQEAKTLLSYFFCQAADSRINSATAVLRGL
IYLLVVQQDSLASHVQKRYKDVGKQLFSDENAWVALHDIFNNVLSDPSLEETYIMIDALD
ECVAGLPDLLNLIVKSTSSRVKWIVTSRNWPSIAERLDVATEVTSLSLERNQEFI
>EEU42176_Nectria_haematocca_EEU42176__NACHT_WD40_Het-s_PFD_92_267
LLWIRGDPGKGKTMLLSGIINEQEPSTRLENSETDTSLSYFFCQATNPGLNNATAILRGL
AYLLVKQQPCLISYVRDKASLNPEHWNSGVAIRDILSKILGDSALQNVIFVVDALDECVT
DLELLLDFISSTTSTKVRWLVSSRNIWEIEQRLKQTQTKLALSLELNAESVSQAVN
>Fusarium_verticillioides_FVEG13488__NACHT_WD40_Het-s_PFD_92_245
RLLWIRGDPGKGKTMLLCGLVNELQPLTRLDGSQNSKTMSYFFCQATNSGLNNYTAILKG
LIYLLVIQHPPAVSHLDDEEDKDHWNYKVSLESTFRKILDEPGLGETYLLVDALDECVED
LPLLLALISSTSSRAKWIATSRNRCEIEELFGEA
>EEQ35356_Arthroderma_otae_EEQ35356__NACHT_WD40_Het-s_PFD_103_276
RLLWVRGDPGKGKTMLLCSIIDELKKSNSEKHNLSFFFCQATNSQYNNATAVLRSLIRQM
VNQQPSLIKHVRKEYDNYRDIFKRINAWITVCDIFKNILEDRTLNHNFLIIDALDECTTG
LTDLLNLVRQTSATYSNLKWIVSSRHWPSIEDSLNMAAQKSLLSLELNEESISA
>CAL30203_Podospora_anserina_hnwd1_HET_NACHT_WD40__292_454
RLLWIKGDPGKGKTMLLCGIINELEGIIIADGHCRNLAYFFCQATDSRINNAIAVLRGLI
YLLAHQQPRLISHIRKYTDKAKSLSDANAWFVFSDILGGMLGDPNLKPTYLVIDALDECM
GDLPRLLKFIVGMSSTFPCVKWVVSSRNWPNIEESLEAAEKKI
>CAL30202_Podospora_anserina_hnwd2;_het-R_HET_NACHT_WD40__294_442
RLLWIKGDPGKGKTMLLCGIIDELEQPITASGGNLAYFFCQATDSRINSAIAVLRGLIYL
LARRQPGLLLYLPENTYASDDAMAWVVLSKVLRRMLEDPDLKETYLVIDALDECVIDLPK
LLDFVVISPGRVKWLLSSRNEVLIEEKLK
>CAL30201_Podospora_anserina_hnwd3_HET_NACHT_WD40__305_467
RLLWIKGDPGKGKTMLLCGIIDELEVSTPTSLLSFFFCQATDPRINSATAVLRGLVYILI
KQQPTLISHVRQHYDPAGGRGFEDPNTWVVLCEILTSILQDPVVRIAYLFIDALDECVTD
LPRLLKLIVEMSSTSSRVKWIVSSRNWTQIEEELEMAAQNARL
>CAL30216_Podospora_anserina_hnwd4;_het-D_HET_NACHT_WD40__293_449
RLLWINGDPGKGKTMLLCGIINELQGAIVADGHCRNLAYFFCQATDSRINNAIAVLRGLI
YLLAHQQPRLISHVRKYTDAGKSLSDANAWFALSDILVGMLGDPNVKPTCLVVDALDECV
IDLPKLLDFIVCISSDRIKWLLTSRNETIIEKKLKSN
```

>CAL30215_Podospora_anserina_hnwd5;_het-E_HET_NACHT_WD40__294_488
RLLWINGDPGKGKTMLLCGIIDELKKSTPPGLLSFFFCQATDSRINNATAVLRGLIYLLV
SQQPALISHVRRPYDHAGKKMFEGPNVWIVLCEIFTSILQDPGLRMTYLIIDALDECVTD
LPQLLELITRTSCTSSPIKWIVSSRNWPDIEEQLETATQKARLSLELNAESISTAVNAFI
QNRIDQLASKTKHDA
>CAL30200_Podospora_anserina_nwd1_NAD_NACHT_WD40__86_261
RLLWIKGDPGKGKTMLLCGIIDELKKGPNSLLSYFFCQATEAQLSNAASVLRGLIYLLIL
QQPSLISHVRSKHDVAGEKLFQGINVWVSLVEIFTDMLKDPTLKDAVLIIDALDECTTDR
PKLLDFIIQSLTISSSPVKWIVSSRNWQDIEEKLDRIEQKVRLQLELNQDSISKAV
>CAP64781_Podospora_anserina_nwd3_SESA_NACHT_WD40__227_387
LLWIKGGAGKGKTMLLATIIKQLQSRTKPDHTATSSLLSFFFCQNTDDRLNNAVAILKGL
IYLLLIQDVNLVLYLKTDYDRMGREIFDATKNVNAFDVLSNVFRQMIQHSRSETVYLAVD
ALDECEDGLPNLLGLIRDTVLQQNRLKWIVTSRNGVDIDEG
>CAP62256__Podospora_anserina_nwd4_SESB_NACHT_WD40__394_544
VLWLVGLPGTGKSTLAAKTIDHIQQTLHESNCQYHFFSESDPLKRDLSLCLRAIALQLAI
AHPPLAGRLIKFHRDTNFSAAEQTFARIWDTIFENIIFPIDFGHSLHWVFDGLDEANQPH
FLVRRLMLMQCRTTIKILFISRPQRNLTAIL
>CAP61990_Podospora_anserina_nwd5_RelSpo_NACHT_WD40__376_524
FWLNGMAGTGKSTISRTVAQLFADQNLLGASFFFKRGEKDRSNAALLFTTIATQLIVKEP
SLVSYIKAAIEADPYVTSKRLEEQFKKLILKPLENLKGSLDDIKTIVLVIDALDECERDD
DIRVIISLLSQAKSLISVRLRAFLTSRPE
>AAS80312_Nectria_haematocca_het-eN__NACHT_ANK_σ_-_motif_35_183
LLWIKGKPGSGKSTLLRNVLNNATPRLKTEEGALILSFFFHGRGSELQKTPLGLFRSLLH
QLLRQASKALEDKDLLSIFQQRCETIGKPGEKWQWHPSELPRLFESSLPKVLETHPVWLF
VDALDECGKENAVKLVKVFKSLLQGLPSS
>EEQ35657_Arthroderma_otae_MCYG_08476__NACHT_ANK_σ_-_motif_107_234
MITADPGCGKSVLAKYLIDDKLPELSSATICYFFFKDQDQNTLNQATCALLHQLFSHKPL
LIRHAMPEYSKNGPDLVNITSSLWNVLVAAGRDPEAGPVIFILDALDECRGSDLRNLLQR
LKNQFTQN
>EFR01951_Arthroderma_gypseum_MGYG_04952__NACHT_ANK;_WD40_σ_-
_motif_115_272
VFWISGSPGKGKTMLLCGIIDHLSDPGAKHIPPSFFFFQESDSQLNNATAVLRGLIYLLA
IQHRSFLEYLQERYSLSGGTLFADPDNFFALSNVFRNMLSIQTTNPIYLIVDALDECQTD
LRKLLDLICQTSSTPSNCVKWIVSSRNLYDIKEVLGPL
>EFR03973_Arthroderma_gypseum_MGYG_06975__NACHT_ANK_σ_-_motif_98_239
LLWIQGKPGSGKSTLLHYALKNVNTTAAKKDNTIFLSFFFHSRGGELQKSLLGFFQSLLH
QLLQHLPEAPADLQTDFDEKCKNYGEPGQQWKWHLTELRGYFRSSLKEALKSRSIWLFVD
ALDECGKDGANKLVKDFKSWLQ
>EFR02206_Arthroderma_gypseum_MGYG_05209__NACHT_HEAT_σ_-
_motif_108_259
RILIRGRAGVGKSTLCKKIVHEYIHNGMWNQHFDWLVWVPLRILKNIETHGPYTLGDLFT
QYFSTNPNTKDLAECAWRTITGPAKDRTLFVLDGLDEVFGQWNEGEPIQVFLDSLLAQDY
VIITTRPYTLDRERLEPIDLELETIGFRPEQV
>EAA64853_Aspergillus_nidulans_AN2021__NACHT_WD40_PP_-_motif__90_239
LLWIKGGAGKGKTMLSIGLIEQLARAQDDSTVVIYSFCQNADYELNTLEAILKGLILQLA
NRQPELKESLRRRWDTIQESFSEDVTSWQSLWNILFEMLARCKYSRVYMVVDALDECQDN
DMVDFLKSIVRKGLDQPGKVKWMLTSRPWD
>XP_001824279___Aspergillus_oryzae_AOR_1_1386094__NACHT_ANK_σ_-
_motif_102_259

MLWVTADPGCGKSVLAKYLADSVLTSHAGRTVGYFFFKDDFEDQRSIVKALCCIFHQLFY
QNRDLLTAHIIEQFEMDERIVDSFGSLWNVLISAAKQTDTTEIICLLDAFDECEPDGRSQ
LTVALERLYTDESRHNFNLKFLITSRLYGDIRQGFQPM
>XP_389348_Fusarium_graminearum_FG09172__NACHT_ANK_σ_-_motif_62_218
GSGKSILISLIIHSMLQKAQENSKLACVYFYFNHGDTLQPSVSQLWAALLEQLLEQNSDE
NIAQEVQSIFDRSRYGVISIPPIEYFNLFKAQAVLFETVYLIIDSLNICADYNDDTSHQT
LLETFQKLPDNVRVIFSSRDGSLANHLLVQKKIKVKP
>EFE34609_Arthroderma_benhamiae_ARB_06372__NACHT_ANK;_WD40_σ_-
_motif_119_277
VFWISGSPGKGKTMLLCGIIDHLSNPGVKHIPPSFFFFQEGDSQLNNATTMLRGLIYLLA
LQHRSFLEYLQERYSLSGGTLFADPENFFALSNVFRNMLSIQTTKPIYLIVDALDECQTD
LRKLLDLICQTSSTPSNCVKWIVSSRNLYDIKEVLGPFE
>EFE37924_Trichophyton_verrucosum_TRV_07414__NACHT_ANK;_WD40_σ_-
_motif_119_276
VFWISGSPGKGKTMLLCGIIDHLSNPGVKHIPPSFFFFQEGDSQLNNATTMLRGLIYLLA
LQHRSFLEYLQERYSLSGGTLFADPENFFALSNVFRNMLSIQTTKPIYLIVDALDECQTD
LRKLLDLICQTSSTPSNCVKWIVSSRNLYDIKEVLGPF
>EGE01227_Trichophyton_equinum_TEQG_00280__NACHT_ANK;_WD40_σ_-
_motif_119_276
VFWISGSPGKGKTMLLCGIIDHLSNPGGKHIPPSFFFFQEGDSQLNNATAVLRGLIYLLA
LQHRSFLDYLQERYSLSGGTLFADPENFFALSNVFRNMLSIQTTKPIYLIVDALDECQTD
LRKLLDLICQTSSTPSNCVKWIVSSRNLYDIKEVLGPF
>EGD95927_Trichophyton_tonsurans_TESG_03388__NACHT_ANK;_WD40_σ_-
_motif_119_276
VFWISGSPGKGKTMLLCGIIDHLSNPGGKHIPPSFFFFQEGDSQLNNATAVLRGLIYLLA
LQHRSFLDYLQERYSLSGGTLFADPENFFALSNVFRNMLSIQTTKPIYLIVDALDECQTD
LRKLLDLICQTSSTPSNCVKWIVSSRNLYDIKEVLGPF
>EGD85581_Trichophyton_rubrum_TERG_01851__NACHT_ANK;_WD40_σ_-
_motif_119_277
VFWISGSPGKGKTMLLCGIIDHLSNPGVKHIPPSFFFFQEGDSQLNNATAVLRGLIYLLA
LQHRSFLDYLQERYSLSGGTLFADPENFFALSNVFRNMLSIQTTKPIYLIVDALDECQTD
LRKLLDLICQTSSTPSNCVKWIVSSRNLYDIKEVLGPFE
>EAU30332_Aspergillus_terreus_ATEG_09195__NACHT_ANK_σ_-_motif_94_280
LLWIKGKPGAGKSTLMAFLYGQFIKPGPAKPGITLEFFFSARGTELQHTPLGMLRSLLNQ
LYRRDPDIRPAVRKIYQDKCDAFGRSKKSWEWQQPELERILHELIVTSARKQSLTVFVDA
LDEAGQKSAQRLVRYFHQVNESIARVSALGKLCISCRHYPVIARISGTEISVDEYNHDDI
TAYIRDF
>EFY96195__Metarhizium_anisopliae_MAA_08306__NACHT_ANK_σ_-
_motif_35_195
LLWIKGKPGSGKSTLLRHALDDAKKKRNVKENLLILSFFFHGRGTELQRTPEGFYRSLLH
QLSKTPDAVSDVVSTFQEKCETFGEVGKAWQWHPKQLRELFESSIREALKLRPICLFVDA
LDECGEKDATMLAQRFKDLLDSLRSTAYNSFHICFTCRPYP
>EED12256__Talaromyces_stipitatus_TSTA_003180__UDP_NACHT_ANK__381_535
RILWLRGNPGTGKSTMAITLTEELPNQPYFSGKNKAFAYFFCDSSAENRRTATAILRGIL
FQLINEWPILMKYLFGKYEGRKEKLFTSFDALWAVLIDMGHDSTHSGIYCVIDALDECEP
ESQQIILNQMNQTFNSRNSKHSTPSNIHILITSRP
>EED44734__Aspergillus_flavus_AFLA_114360_UDP_NACHT_ANK;_WD40__378_53
4
RLLWVSANPGCGKSVLAKYLADFLPKLDVSRAVGYFFFKDDYEDQKSITNALCCILHQLF

DKKRHLLDDTILEQFEMNESVTSSFSELWIILLKAASKENAGEIVFLLDALDECEQHGSS
QFMEALRRLYTDESQHNFNLKFLITSRPYSHIRQGFQ
>EAW16767__Neosartorya_fischeri_NFIA_001140_UDP_NACHT_WD40__376_531
LLWIKGDAGKGKTMLMIGIIKELLKPKSSKLLAYFLCQGTDRNLNNATAVLRGLIYMLIT
KQPHLISRLRQRYDTEGQRLFEGSNAFYSLSTVFENMIEHLQQAPVHLLIDALDECKVDL
ENLLKLIAKTMSMSSVRVKWIVSSRNMGYIEKILNP
>EDN26745__Botryotinia_fuckeliana_BC1G_06991_NAD_NACHT_ANK__439_591
LVTADPGCGKSVLAKYLIDDGLPKLSSATTICYFFFKDQDQDTVKQALCALLHQLFSHKP
SLIRHAMPEFRDNGPSLAGITELLWKILEKAGADPEAGPVIFVLDALDECEGKQFDVLVS
MLNRHIQKDKKEIGKMKFLLTCRPYDSLTSQFH
>EFE37059_Arthroderma_benhamiae_ARB_04586_Goodbye_NACHT_TPR__325_485
VSGNPGAGKSYLASNMITYLNGEYPQLVQHSSRVSVGYFFFKDDNPKTMSIHQALRDIAY
QISQNDPVYAKYIVTNALTAEDVSTIESAWRTLFVNFFVKKSNVASSVYLLLDGVDEAFD
AEIQPFFDLLKDINEVTDNPRIQLAIVGRPQLGELISESLE
>EFX04723_Grosmannia_clavigera_CMQ_1651_SESB__NACHT_ANK__344_502
LMWIKGKPGSGKSTLLKHALGNQRILSSAKKDDLVLSFFFHDRGDSLQRTPLGFSRSLIH
QVLSQSPVALSDLIKTFKRKCVEIGEPGNKWDWHQGELQRCFESSISAVLKERSVWLFVD
ALDECGRDSAVELFQWFKSLLQTLPQTLRFHICVTCRHY
>EGU75419_Fusarium_oxysporum_Fo5176_FOXB_14068__NACHT_ANK_?_80_264
LLWIKGKPGSGKSTLLRYVLDHIIEIPNTRKGALIVSFFFHGRGSELQKTPMGLFRSLLY
QLLRQVPEALTYIMDTFQQRCETVGKPGEKWQWHPRELPRFFESSLPKVLEARPVWLFVD
ALDECGQRNAVKLVREFKSLLQGLPFTGSQFHICFTCRHYPILDQTCQFEICLEEENRQD
ISTYV
>EGU82782__Fusarium_oxysporum_Fo5176_FOXB_06703__NACHT_ANK_σ_-
_motif_134_281
LLWIKGKPGSGKSTLIRYVLDHVMPIPNTTEGALILSFFFHGRGSELQKTPLGLFRSILH
QLLRQVPEALTDLVATFQQRCETVGKPGEKWQWHPHELPRLFESSLPKVLETRPVWLFVD
ALDECGKENAVNLAEEFNALLQRLPSCG
>EGU82780_Fusarium_oxysporum_Fo5176_FOXB_06701__NACHT_ANK_σ_-
_motif_180_328
LLWIKGKPGSGKSTLLRYVLDHVMPIPNTRGGALILSFFFHGRGTELQKTPLGLFRSILH
QLLRQVPEALTDLVATFQQRCETVGKPGEKWQWHPHELPRLFESSLPKVLETHPVWLFID
ALDECGKENAVRLNRDFKSLFRGLPSCGL
>EGU86408_Fusarium_oxysporum_Fo5176_FOXB_03074__NACHT_ANK_σ_-
_motif_128_271
LLWIKGKPGSGKSTLLRYILNHAMAISNTGKGALILSFFFHGRGTELQKTSLGLFRSLLY
QLLRHVPETLTDLVATFQQRCETVGKPGEQWLWHPRELKRFLEPSLRKVLEARTVWLFID
ALDECGKKNAVRLVREFKLLLQEL
>EGU86797_Fusarium_oxysporum_Fo5176_FOXB_02687__NACHT_ANK_σ_-
_motif_115_258
LLWIKGKPGSGKSTLLRYVLDHVMEIPNTREGALILSFFFHGRGSELQKTPFGLFRSLLH
QLLDQVPEALTDLVATFKQRCETIGKPGEQWQWHTRELQRFLESSLPKVLEARTVWLFVD
ALDECGEENAAKLAREFKSLLQGA
>Pa-1-15610_123_316
LHISGKPGAGKSTLMKFLFEHETTYEKLGEWVGDVRKLVLGKFFFWRQGTPLQKSLSGLK
RALLYSILKQCSGFIETIFPQHWDPSEYQPGIQPSALSIDNKEISDGLNQLLCSPDVYSH
QRLALFIDGLDEFEDDHQSHHDLLDAIMEWVDSSRGCLKLCVSSREDRVFMERFRAQQRF
RLHELDAADILAVI
>Pa-3-10930(NWD1)(upx3.08)_380_555

RLLWIKGDPGKGKTMLLCGIIDELKKGPNSLLSYFFCQATEAQLSNAASVLRGLIYLLIL
QQPSLISHVRSKHDVAGEKLFQGINVWVSLVEIFTDMLKDPTLKDAVLIIDALDECTTDR
PKLLDFIIQSLTISSSPVKWIVSSRNWQDIEEKLDRIEQKVRLQLELNQDSISKAV
>Pa-7-5020(upx5.81)_240_390
RLLWIHGVPGVGKTVLSSYIVEQYKGFKQPYAFYYCSSTDEKGEKWERDETAHCLGWILA
RLCLQIKSVPAFLKPLKDRGMYPTVEDLFKGIEAVLSFLNGRRAYVVIDALDESRTSNLV
KAVATLATDTKYKNLFLAVTSRRQADVQKAL
>Pa-3-9600(upx3.44)_443_597
RFLWLNGPPGSGKSVASTHVIKYLESFNLDCAYFFFKNNEKPSLTQLLLSLALQMAESNF
QVRHTFLSMIEEGEVVDCHSDHVMVWNNIFLGRIFKMAFSQPQYWVIDALDECQSRLLAT
LVAMLSRIEPTVPLRILITSRPNGHVERLLNQERV
>Pa-2-4800(nc)_294_459
IFGKPGSGKSTLLKHLLKHPAVQNMLEGWAGGKALIKCSFFFWKGGSVGQKTFSGLYRSL
LCSVFKQCPELVPSIFPSLWQLCLQGGNAQLTDAEARRAFLEIMERDEVFTHRKFAFFID
GLDEFEGDDTGLVRTFLGWTRLRPDNIKICVSSRELPLFQERFSSY
>Pa-7-3110(HNWD3)(nc)_305_467
RLLWIKGDPGKGKTMLLCGIIDELEVSTPTSLLSFFFCQATDPRINSATAVLRGLVYILI
KQQPTLISHVRQHYDPAGGRGFEDPNTWVVLCEILTSILQDPVVRIAYLFIDALDECVTD
LPRLLKLIVEMSSTSSRVKWIVSSRNWTQIEEELEMAAQNARL
>Pa-4-1920(het-E)(nc)_294_488
RLLWINGDPGKGKTMLLCGIIDELKKSTPPGLLSFFFCQATDSRINNATAVLRGLIYLLV
SQQPALISHVRRPYDHAGKKMFEGPNVWIVLCEIFTSILQDPGLRMTYLIIDALDECVTD
LPQLLELITRTSCTSSPIKWIVSSRNWPDIEEQLETATQKARLSLELNAESISTAVNAFI
QNRIDQLASKTKHDA
>Pa-1-7130(up2.87)_289_419
TLICQGKLGSGKSVLLANMVADLGLHVGSAKCSIVYFFCRHNIDESLKAQTIIGSIVRQL
LTRVADFTEIERQIVEDYSPRSSLAVDEGYRMLQDCFPAGLEAFIILDGLDECTEKEKAT
VYDQLQTLQKR
>Pa-3-2560(nc)_51_244
ISGKPGSGKSTLMKYLIEHTATSGHLNVWATVAQFFFWRPGTEPQRSLGGLYRTLLHNVF
QKCPELISAVMPSFWQRLQSMPWQLSSDLEVTESTVKAAFNRLISSEVAEAFPNHCFCLF
IDGLDEYEAIIQTDHTDLAALLNSWAEAGLSSRNIKLCVSSREYNSFMNLFSDDRRLRLH
ELTHSDMVACVRDK
>Pa-2-8310(upx5.44)_152_304
LWIKGVPGAGKSVMAASIIRHLETTENCPVLFFFFRNIVAANYSPRALLQDWLAQLLPFS
PKLQLALQSQLNTDLAIISNNDLFDHFLSGISCVPRLYCVADALDEMNTDSRPFLDKFNR
LATHRPGSLKLLLTSRPKQYLQSALRDTSIVHI
>Pa-3-8560(upx3.13)_412_531
LWMRGKAGAGKSTMMKFVYLEMKKGPKKPSVAVVSFFFNARGDYLERSISGMYRSLLSQL
LHEFSDLQSVLDNTDIVPRNQQDCPDLNALKDLLSNAVMALGQRCFTCFIDALDECDEQE
>Pa-5-340(nc)_193_370
FWISGKPGSGKSTLMKFLLQNHRTRKHLELAYPGALIVLHFFWLAGQDMERSIKGLLCSL
LHQLISEREQGYIDIVQHLGFGKVKLKEGCSDWSTKELQETVFHVLASSSAPVLIFLDGL
DEVDPSDGPLTLLKLVNDICALPNIKVCTTSRPEPIYSRQLSMKPSFRVQDLTSHDIK
>Pa-5-640(upx6.03)_125_282
GKTVLTAKTIRYLQSQGQTLLFAFLSYRDERKSKPVKIFQSLVFQLLEEQSMLHPLLHDI
YLTNYRKLISNPDFVGDLLGKLLQTSGPTIIVIDGVDEAAESDKSYLVRSLLRVTKSGPN
VKLFVSSRMDSAISKELMRSSTELHVEDHNEGDIHELI
>Pa-5-12100(upx4.85)_340_490

VLWLVGLPGTGKSTLAAKTIDHIQQTLHESNCQYHFFSESDPLKRDLSLCLRAIALQLAI
AHPPLAGRLIKFHRDTNFSAAEQTFARIWDTIFENIIFPIDFGHSLHWVFDGLDEANQPH
FLVRRLMLMQCRTTIKILFISRPQRNLTAIL
>Pa-3-9890(nc)_182_335
RILWLSGRPGTGKSIASTHVIKYLESHKLDCSFYFFRHNDRSGANVASLLRSLAFQMAEA
NYQARQAIMRMVEDEVVIDEDDHYTLTSKVFTNGILGIDLQSPQFWVIDAVDECAQESLS
LVVSMISKLEKTVPLHIFLTSRPGGELQRLFNHE
>Pa-3-630(NWD2)_73_218
FWLNGMAGTGKSTISRTVAKSFADDGILGASFFFKRGEGDRGKATLFFPTIASQLVRKIP
ALEAFVREAINNNPDVARKALRDQFEKLILQPLDRIHHAIAVIVVDALDECDGDNDVKVI
ISLLSQAKELRSPGLRIFITSRPELP
>Pa-2-6680(het-r)(upx3)_294_442
RLLWIKGDPGKGKTMLLCGIIDELEQPITASGGNLAYFFCQATDSRINSAIAVLRGLIYL
LARRQPGLLLYLPENTYASDDAMAWVVLSKVLRRMLEDPDLKETYLVIDALDECVIDLPK
LLDFVVISPGRVKWLLSSRNEVLIEEKLK
>Pa-4-6090(upx37)_137_319
YWITGKAGSGKSTLMKFISQHPQLMKHLQVWSNGAPVTIASFYFWASGTAMQASSEGLFR
SLLYQLLDQHKHIIPKLAPQTWEGTYLFGAPMPMVQADDLRRMLGFTVREVGKTSRICLI
IDGLDEFGGSTDDVLDTIEAFTSHSIKVCAASRPWVAFEESFYQKPQLLLQNLTRQDMEE
YTQ
>Pa-2-7940(het-d)(upx3)_293_449
RLLWINGDPGKGKTMLLCGIINELQGAIVADGHCRNLAYFFCQATDSRINNAIAVLRGLI
YLLAHQQPRLISHVRKYTDAGKSLSDANAWFALSDILVGMLGDPNVKPTCLVVDALDECV
IDLPKLLDFIVCISSDRIKWLLTSRNETIIEKKLKSN
>Pa-3-8260(nc)_137_216
RVLWIKGNPGKGKTMLLCGMINELSRLNTGNGKGTCLAYFLCQATDSRISGVTSVLRGLI
YLLIEQNPSLISYIREKYDL
>Pa-5-12630(upx4.11)_120_304
FWVSGKPGSGKSTFIKFIADNTNTQKLLTQWSKGQEVILAAHYFTIYGTPIQRSLEGLFR
SLIYKILSQEPTLIRKVLPQRYKNEKIQEPWKQSELQSVLKKLARELVHSRLCFFIDGLD
EYAGDHLDICETLQDLSRSPCVKLCVSSRPWNVFENALGGDTDSKLYMQDVTRADIQQYT
KTMLR
>Pa-4-1190(HNWD1)(upx2.8)_292_454
RLLWIKGDPGKGKTMLLCGIINELEGIIIADGHCRNLAYFFCQATDSRINNAIAVLRGLI
YLLAHQQPRLISHIRKYTDKAKSLSDANAWFVFSDILGGMLGDPNLKPTYLVIDALDECM
GDLPRLLKFIVGMSSTFPCVKWVVSSRNWPNIEESLEAAEKKI
>Pa-5-10620(nc)_83_270
FWISGKPRSGKSTLVKFLLDDHRTRANFEVNLPHATIISHFFWLPGNAMDRSTKGMLCSL
NHQLLSNSNNNNNYSNVFHHPTFPSIKLKDNPSDWSIKELQDIFVYLLRKSARPILIFLD
GLDEIDPSDGPFFLLSLLDTVCKIPGVKVYTSSRPEPTLQQYFNMTPSFRVQDFTVRDIR
NYARKRLR
>Pa-5-1230(upx3.6)_376_524
FWLNGMAGTGKSTISRTVAQLFADQNLLGASFFFKRGEKDRSNAALLFTTIATQLIVKEP
SLVSYIKAAIEADPYVTSKRLEEQFKKLILKPLENLKGSLDDIKTIVLVIDALDECERDD
DIRVIISLLSQAKSLISVRLRAFLTSRPE
>Pa-1-10530(nc)_35_225
FWVSGKAGSGKSTLMKFPADHETTQTMLEEWAKPFKLLTARHYFWAAGTHMQKSHEGLFK
TLLYEIFCACPSLIPAACPLRWRQTCPERTEQQSTEWTTRELSEALHHLGRNQDLHIRHC
FFIDGVDEFDGDHLMLCEILSNLSQSPNSKLCLSSRPWNVFIDAFGNNIARKISIEDLTR

EDILRYAERRL
>Pa-1-21670(upx56)_246_392
AIDEAKTRHRTLFVFVSHIYQNSTTARSILQSLLFQLAFDVKDAQSLLVESNERELLGST
RHVLGLLRTLLGTPTAGPTYIILDGLDEMEVVERGILLQQLTDLESCPELKILISSRRED
DIASILGAKATEIRVDKRNSDSIQAYI
>Pa-2-8180(upx6.11)_382_555
TLFCPGIPGAGKTILTSVVLDHLGSKFHNDPKIGIAYIYFNFQRQDKQKIDDLLASVLKQ
IAESQPSVPGSVKDLFDKHKAKRTRPSLDEILRVLQSVAATCSCVFIVVDALDECQTSES
CRERFLSELFNLQKMHGINIFATSRSIMEIVDRFKTSISLEIRASTADVAQYLE
>Pa-0-150(upx2.4)_299_447
LWLHAIPGAGKTILSSSIINYLQEKVQDQNTGLAYFYCDYKDSQKQDPAKILSTILAMLA
KQNSGVFENLQDFFLEQLRLAPTFTAEFDELLADFNTFMSDHFETVIIVIDALDETSPSS
WETLTAALRSLHEQCPRLKILVTSRNELP
>Pa-7-10370(upx4.04)_274_400
LWMRGKAGSGKSTLIKSTLRSFQNSKSSKGTTVIHFFFNARGAELEKTTRGMYRALLVQL
LEILQKKMPHFQRDVFKSAAIKTWNVREGREWSVAILEELFENAILSIGQEEVTCFIDAL
DECDDEQ
>Pa-7-3660(down-8.35)_334_516
LLWLKGKPGAGKSTVMKYALAQLECLEGSRSNVASFYFNARGNAMEKSPLGLLRSVLHQL
CLQDCQILATFSQTYQRRQSCDGGSYLPWSGLELESFFEKAFKESSTRRTFIFIDALDEC
SEESVREAVYFIDRLAQTALEGEVLLNICLSSRHYPAIRILNCPEVVVEAHNDSDISTYI
RDK
>Pa-5-7320(nc)_108_301
KAGSGKSTLMKLIAGHSTTRRELERWSGERTLVVSHFFAWRAGDAMQQSLHGLYRSILFS
VLANCPSLIRDVFPTACQVFDTTTFEPHIDEPYFRFPAKLQEGFQNLIEVSVPRNTCFCF
MIDGLDEFKHDPAHRHSHQDLVAQLRSRSQHANIKMLVGSRPEMAFLNLVLCELRVNLHE
LTRWDILRVVQNTF
>Pa-6-7270(upx4.83)_459_624
RRILIHGRAGVGKTTLCKKMVNEFTRRSGEFRKWNELFDRILWVPLRRLKGWSSPPYKLE
GLFCYEYFDQHLNYSILAKELFRTVDSNGQKTLFILDGLDEISQLLDDNHPKFSLLKHLL
NQPSVIITSRPHVSLPRGVHSPDLKLETVGFYPAQVVEYLRATFID
>Pa-5-3370(down-2.12)_227_387
LLWIKGGAGKGKTMLLATIIKQLQSRTKPDHTATSSLLSFFFCQNTDDRLNNAVAILKGL
IYLLLIQDVNLVLYLKTDYDRMGREIFDATKNVNAFDVLSNVFRQMIQHSRSETVYLAVD
ALDECEDGLPNLLGLIRDTVLQQNRLKWIVTSRNGVDIDEG
>Pa_2_8450_768_927
LWLNGFAGCGKSVLSSTAIQLAERHRQQQSRPGKITSGLAYFYFTFNDESKQDAIAMLRS
AILQLAGQIKTGEEHLKRLEDSFVDATPPDDELLETLNAIIREFEDVYIIVDALDESPKD
RHRDDLLDAVQVIREWSEPRLHLLVSSRDEQDIRECLSPS

#IR NB-ARC

>EEQ27528_Arthroderma_otae_EEQ27528__NB-ARC_TPR_PP_-_motif__64_266
KIKEKLEAPGSRVALVGMGGVGKSQIAIQYAYCLENQLSDTWIFWIYASNSARFQESLEE
IAHRGKIPGRDDPKRNTLGLVRNWLLDDKRKWVVILDNADDLDFLKDTSTEEGTNNKRLD
KYLPKTQNGSIFVTSRSREAALQLVDRRNIINVHPMDETQAVSLFRKKLNEGYDDKVIAE
LAAELGYLPLAIVQAASYISLRE
>EFQ97059_Arthroderma_gypseum_MGYG_00103__NB-ARC_TPR_PP_-
_motif__93_302
SWVALVGMGGVGKSQLAIEYAYRLRDQLQLPDTWVFWIYASNSARFRESLEEIAEHAKIP
GRDDPEPGILKLVYRWLDDEKRKWVIILDNADDLDFLEDTSVKGGKPNKSLTKYIPKTEN
GSVFITSRSRETASALVDNKHIIKVNPMDEAHAIDLFQKKLPKPDDKLIAELAAELEFLP
LAIVQAASYISHKEPRCSVQEYLDDFRKMR
>EEA22328_Penicillium_marneffei_PMAA_061070__NB-ARC_TPR_PP_-
_motif__84_309
LEKGSASPSRIALVGLGGVGKSQLVIEYSYQVRDRSPDTWVFWVHTSNAARFEQSYREIA
DRVKIPGRKDPKTNIFNLVSEWLHNEGKGKWMLILDSIDDDRFLHEIPLSSRDESDSRIP
AQPLFNYLPLNAHGSIIITTRSRGVAAKMVEDGDIIAVESMDQIHAQTLFEKKLGKQENQ
EDIIELIAALEFMPLAIVQAAAYIKQRAPRSSVKQYLTEFQKNDYR
>EEA25208__Penicillium_marneffei__PMAA_063270__UDP_NB-
ARC_TPR__348_624
RQKELERLWHHLQPQNSNSRTVAILQGLGGIGKTQLALRFARDHKADYTTILWVNGRSRG
TLLQSLSAILPRLASHSQTFAVTDKEEVEQYARQVLKWLALPGNSRWLLIFDNVDEYSPE
TDDGYDIQKYFPTADHGSILITSRLQSLTEVGRSFAVPTLDNNEAILLLWQSRNLRTPEI
ITENEVDQDTNDLIDRLGGLPLAITIAGAFMRETGTSIREYLQYYQKSWHALQLRARPTR
HYQQGNLLQTWLISYNEIQKRDAHVAELMLLLAHFNN
>EAW19385_Neosartorya_fischeri_NFIA_093530_SESA_NB-ARC_TPR__245_483
ITKVLCPNHKAQRQQRLVLGGMGGIGKTQLAIAYAESVRGSYSSVFWLNAVSEAALKDSF
RSIAGLIFDIEEPGLLEDKQVVRRVHQWLCTPENTGWLLIFDNYDDLDQFQIDCYYPPAS
HGAIVVTSRRPDHVAGTPLHIKPLQNVEDSLAILQTRSKRENVQSDPHAKRLAERLAGLP
LALATAGTYLQRSTFTFERYLKEYEKRWNINPRRPLQLKEYQERTLYTTWDLSYSALEK
>Pa_6_8860_(no_change_in_VI)_10_216
KAAIVGLGGVGKTQVALQLAYWTKKHRPEFSIFWVPALSSATFEQAFAAMARKLPIQSGG
DDDDLKQSVRRYLSSEAAGPWLLVVDNVDDSDIFFGSAGMPGSISEYLPESDDGLTLFTT
RSREVAVSVAGSDVIDLYEMDPLEAAEFLEKSLIYKDMLHDEEAAAELLKELTYLPLAIT
QAAAYINIKQVPLAEYVELLHGTQQDI
>Pa_1_11380_(Patatin,_1TPR,_up_x4)_406_638
CLHGLGGVGKTQIALKAANIFRHGDPELGIPRKRRFPHILYVDGTDKTTISQSYASIARD
EFGIEASGNSDQLMRQALQKMERLDEDWFLIYDNCNQEDRRELLPKGDTGNVLFTTRNRV
VRNQMRDECVYDVEVLEELDAIRLLLTASGSKYADLNAWDQSIGKEIVEELGYLPLAIDQ
AAAYIREAPCPLDGYLEVFRKQRVELLRNPKFKGSLAQNQAVYTTFEASYKAI
>Pa_2_10340_(tpr,_no_change_in_VI)_263_456
DKDDCQRTAIEGLGGVGKTQVALEVAFRVSNEHPNCSVFWVPAVDVTSFENAYRTIGQQL
TVPGIDEVKADIKALVKTALSRESTGSWLLIIDNADDRKLLLSDTALTDYLPFSRKGSIL
FTTRTHEVAVKLVGPKSHIISVEEMSRDEAFKLLQKGLKGDQIRDTASTIALLEFLTNLP
LAIQQASAYMAEKQ
>Pa_7_3550_(TPR,_no_change_in_VI)_53_245
YAVSLYGLGGVGKSQLALDYAEKHKHDYNPILWIDATDEETVRSSFKICAAELGLTVEGG
ENQGSIIMDAGVRAVLRWLCDRSEADDEWLLIVDNADDVSWGIQKVMPRGNRGRVIITSR
DEQSTKLVGGTCESVRVGDMSPPEGRALLLRHLQLDEELAPGGIKDDCDRVVKKLEFLAL

AIDIAGAYIGSHS
>Pa_2_5390__(No_repeats,_no_change_in_VI)_1_133
MGGVGKTQIATQYAYQSLDKFDAVLWMASDNAIAIGQSFRTVAGGLGLLGTDEETKDAAG
ASIETLTCSDTTCLIIFDNADDLTSLKTAWPGSISASILVATRDLSVATASTAQHIQVNA
LGDDEGTRCFSRL
>Pa_6_7950_(TPR,_no_change_in_VI)_12_203
EDDCQRTAIEGLGGVGKTQIALETAYRIRDVQPECSVFWVPAIDTTAFENAYRAIGQQLK
VPGINEEKADVKALIKSVLGRESMGNWLLIIDNADDEKLLFGDTALADYLPFSRKGSILF
TTRNHKLGLRLVESENHIIAVEEMSRDEALKLLGKNLKGSQMSDTRSNNALLEFLTNLPL
AIRQASAYMAKE
>Pa_5_12660_151_368
LAIRGMGGVGKTELAREFAFSRQHYFDAVIWVEAEEATQLSEGFELFATYLGANTGQDRV
VSRNIAREWLNNPTKRPKARPKDATAPTEGDDGAEDTSNKKASWLLIFNNADDLSLLEDY
WPDNPDGSILLTSRNLEAQVGRLTLDLEPFEREEAADLLRQLTPGIDHTLPQNVQASLDI
AERLGGLPLAITQIAAFITKRDKSLPEFVAFYDRYSVE

#FV NACHT

```
>C2TA_HUMAN_414_583
RVIAVLGKAGQGKSYWAGAVSRAWACGRLPQYDFVFSVPCHCLNRPGDAYGLQDLLFSLG
PQPLVAADEVFSHILKRPDRVLLILDGFEELEAQDGFLHSTCGPAPAEPCSLRGLLAGLF
QKKLLRGCTLLLTARPRGRLVQSLSKADALFELSGFSMEQAQAYVMRYFE
>NALP6_HUMAN_197_365
TVVLQGPAGIGKTMAAKKILYDWAAGKLYQGQVDFAFFMPCGELLERPGTRSLADLILDQ
CPDRGAPVPQMLAQPQRLLFILDGADELPALGGPEAAPCTDPFEAASGARVLGGLLSKAL
LPTALLLVTTRAAAPGRLQGRLCSPQCAEVRGFSDKDKKKYFYKYFRDE
>NAL11_HUMAN_149_315
VFLMGERASGKTIVINLAVLRWIKGEMWQNMISYVVHLTAHEINQMTNSSLAELIAKDWP
DGQAPIADILSDPKKLLFILEDLDNIRFELNVNESALCSNSTQKVPIPVLLVSLLKRKMA
PGCWFLISSRPTRGNNVKTFLKEVDCCTTLQLSNGKREIYFNSFFKD
>NAL12_HUMAN_211_381
RTVVMQGAAGIGKSMLAHKVMLDWADGKLFQGRFDYLFYINCREMNQSATECSMQDLIFS
CWPEPSAPLQELIRVPERLLFIIDGFDELKPSFHDPQGPWCLCWEEKRPTELLLNSLIRK
KLLPELSLLITTRPTALEKLHRLLEHPRHVEILGFSEAERKEYFYKYFHNA
>NALP5_HUMAN_280_443
RTVVLHGKSGIGKSALARRIVLCWAQGGLYQGMFSYVFFLPVREMQRKKESSVTEFISRE
WPDSQAPVTEIMSRPERLLFIIDGFDDLGSVLNNDTKLCKDWAEKQPPFTLIRSLLRKVL
LPESFLIVTVRDVGTEKLKSEVVSPRYLLVRGISGEQRIHLLLE
>NALP9_HUMAN_146_311
HTVVLEGPDGIGKTTLLRKVMLDWAEGNLWKDRFTFVFFLNVCEMNGIAETSLLELLSRD
WPESSEKIEDIFSQPERILFIMDGFEQLKFNLQLKADLSDDWRQRQPMPIILSSLLQKKM
LPESSLLIALGKLAMQKHYFMLRHPKLIKLLGFSESEKKSYFSYFF
>NLRC3_HUMAN_139_304
RVSITIGVAGMGKTTLVRHFVRLWAHGQVGKDFSLVLPLTFRDLNTHEKLCADRLICSVF
PHVGEPSLAVAVPARALLILDGLDECRTPLDFSNTVACTDPKKEIPVDHLITNIIRGNLF
PEVSIWITSRPSASGQIPGGLVDRMTEIRGFNEEEIKVCLEQMFPE
>NLRX1_HUMAN_160_320
QTVVLYGTVGTGKSTLVRKMVLDWCYGRLPAFELLIPFSCEDLSSLGPAPASLCQLVAQR
YTPLKEVLPLMAAAGSHLLFVLHGLEHLNLDFRLAGTGLCSDPEEPQEPAAIIVNLLRKY
MLPQASILVTTRPSAIGRIPSKYVGRYGEICGFSDTNLQKL
>NAL14_HUMAN_178_345
IVVLQGAAGVGKTTLVRKAMLDWAEGSLYQQRFKYVFYLNGREINQLKERSFAQLISKDW
PSTEGPIEEIMYQPSSLLFIIDSFDELNFAFEEPEFALCEDWTQEHPVSFLMSSLLRKVM
LPEASLLVTTRLTTSKRLKQLLKNHHYVELLGMSEDAREEYIYQFFED
>NAL13_HUMAN_230_401
TIVLVGRAGVGKTTLAMQAMLHWANGVLFQQRFSYVFYLSCHKIRYMKETTFAELISLDW
PDFDAPIEEFMSQPEKLLFIIDGFEEIIISESRSESLDDGSPCTDWYQELPVTKILHSLL
KKELVPLATLLITIKTWFVRDLKASLVNPCFVQITGFTGDDLRVYFMRHFDD
>NAL10_HUMAN_168_331
LVVLQGSAGTGKTTLARKMVLDWATGTLYPGRFDYVFYVSCKEVVLLLESKLEQLLFWCC
GDNQAPVTEILRQPERLLFILDGFDELQRPFEEKLKKRGLSPKESLLHLLIRRHTLPTCS
LLITTRPLALRNLEPLLKQARHVHILGFSEEERARYFSSYFTDE
>NALP8_HUMAN_205_371
TVAIQGAPGIGKTILAKKVMFEWARNKFYAHKRWCAFYFHCQEVNQTTDQSFSELIEQKW
PGSQDLVSKIMSKPDQLLLLLDGFEELTSTLIDRLEDLSEDWRQKLPGSVLLSSLLSKTM
LPEATLLIMIRFTSWQTCKPLLKCPSLVTLPGFNTMEKIKYFQMYFG
```

```
>NLRC5_HUMAN_222_382
RVTVLLGKAGMGKTTLAHRLCQKWAEGHLNCFQALFLFEFRQLNLITRFLTPSELLFDLY
LSPESDHDTVFQYLEKNADQVLLIFDGLDEALQPMGPDGPGPVLTLFSHLCNGTLLPGCR
VMATSRPGKLPACLPAEAAMVHMLGFDGPRVEEYVNHFFSA
>NALP7_HUMAN_173_339
TVVLHGPAGVGKTTLAKKCMLDWTDCNLSPTLRYAFYLSCKELSRMGPCSFAELISKDWP
ELQDDIPSILAQAQRILFVVDGLDELKVPPGALIQDICGDWEKKKPVPVLLGSLLKRKML
PRAALLVTTRPRALRDLQLLAQQPIYVRVEGFLEEDRRAYFLRHFGD
>NALP4_HUMAN_149_317
RTVIIQGPQGIGKTTLLMKLMMAWSDNKIFRDRFLYTFYFCCRELRELPPTSLADLISRE
WPDPAAPITEIVSQPERLLFVIDSFEELQGGLNEPDSDLCGDLMEKRPVQVLLSSLLRKK
MLPEASLLIAIKPVCPKELRDQVTISEIYQPRGFNESDRLVYFCCFFKD
>NALP3_HUMAN_220_389
HTVVFQGAAGIGKTILARKMMLDWASGTLYQDRFDYLFYIHCREVSLVTQRSLGDLIMSC
CPDPNPPIHKIVRKPSRILFLMDGFDELQGAFDEHIGPLCTDWQKAERGDILLSSLIRKK
LLPEASLLITTRPVALEKLQHLLDHPRHVEILGFSEAKRKEYFFKYFSDE
>NALP1_HUMAN_328_497
RIVILQGAAGIGKSTLARQVKEAWGRGQLYGDRFQHVFYFSCRELAQSKVVSLAELIGKD
GTATPAPIRQILSRPERLLFILDGVDEPGWVLQEPSSELCLHWSQPQPADALLGSLLGKT
ILPEASFLITARTTALQNLIPSLEQARWVEVLGFSESSRKEYFYRYFTDE
>NOD2_HUMAN_293_462
DTVLVVGEAGSGKSTLLQRLHLLWAAGQDFQEFLFVFPFSCRQLQCMAKPLSVRTLLFEH
CCWPDVGQEDIFQLLLDHPDRVLLTFDGFDEFKFRFTDRERHCSPTDPTSVQTLLFNLLQ
GNLLKNARKVVTSRPAAVSAFLRKYIRTEFNLKGFSEQGIELYLRKRHHE
>NLRC4_HUMAN_164_315
PCIIEGESGKGKSTLLQRIAMLWGSGKCKALTKFKFVFFLRLSRAQGGLFETLCDQLLDI
PGTIRKQTFMAMLLKLRQRVLFLLDGYNEFKPQNCPEIEALIKENHRFKNMVIVTTTTEC
LRHIRQFGALTAEVGDMTEDSAQALIREVLIK
>NALP2_HUMAN_208_374
TVVLYGPAGLGKTTLAQKLMLDWAEDNLIHKFKYAFYLSCRELSRLGPCSFAELVFRDWP
ELQDDIPHILAQARKILFVIDGFDELGAAPGALIEDICGDWEKKKPVPVLLGSLLNRVML
PKAALLVTTRPRALRDLRILAEEPIYIRVEGFLEEDRRAYFLRHFGD
>NOD1_HUMAN_197_367
TIFILGDAGVGKSMLLQRLQSLWATGRLDAGVKFFFHFRCRMFSCFKESDRLCLQDLLFK
HYCYPERDPEEVFAFLLRFPHVALFTFDGLDELHSDLDLSRVPDSSCPWEPAHPLVLLAN
LLSGKLLKGASKLLTARTGIEVPRQFLRKKVLLRGFSPSHLRAYARRMFPE
>BIRC1_HUMAN_465_617
VMCVEGEAGSGKTVLLKKIAFLWASGCCPLLNRFQLVFYLSLSSTRPDEGLASIICDQLL
EKEGSVTEMCVRNIIQQLKNQVLFLLDDYKEICSIPQVIGKLIQKNHLSRTCLLIAVRTN
RARDIRRYLETILEIKAFPFYNTVCILRKLFSH
```

#FV NB-ARC

```
>APAF_HUMAN_129_410
RKKLVNAIQQKLSKLKGEPGWVTIHGMAGCGKSVLAAEAVRDHSLLEGCFPGGVHWVSVG
KQDKSGLLMKLQNLCTRLDQDESFSQRLPLNIEEAKDRLRILMLRKHPRSLLILDDVWDS
WVLKAFDSQCQILLTTRDKSVTDSVMGPKYVVPVESSLGKEKGLEILSLFVNMKKADLPE
QAHSIIKECKGSPLVVSLIGALLRDFPNRWEYYLKQLQNKQFKRIRKSSSYDYEALDEAM
SISVEMLREDIKDYYTDLSILQKDVKVPTKVLCILWDMETEE
>gi_6648973_gb_AAF21315.1__disease_resistance_protein_A5__Capsicum_an
nuum__1_205
GMGGVGKTTIAKAMFDTLSYQFKAACFLADVKENSRKNELHSLQNTLLSELLRKKDNYIN
NKLDGKCLIPSRLCSMNVLIVLHNDHLEYLAGDLCWFGIGSRVVVTTRNRHLIEKDDAIY
EVPALPDPEAMQLCNQHALKKEISDECFKMFSLEVVNIAKGLPLALKVWGSFLHRRDITE
WRNAIEQMKYNSNSEIIQKLKISYD
>gi_6456755_gb_AAF09256.1_AF202179_1_disease_resistance_protein_BS2__
Capsicum_chacoense__156_438
DDQRKQLLEDLTRSYSGEPKVIPIVGMGGIGKTTLAKEVYNDESILCRFDVHAWATISQQ
HNKKEILLGLLHSTIKMDDRVKMIGEAELADMLQKSLKRKRYLIVLDDIWSCEVWDGVRR
CFPTEDNAGSRILLTTRNDEVACYAGVENFSLRMSFMDQDESWSLFKSAAFSSEALPYEF
ETVGKQIADECHGLPLTIVVVAGLLKSKRTIEDWKTVAKDVKSFVTNDPDERCSRVLGLS
YDHLTSDLKTCLLHFGIFPEDSDIPVKNLMRSWMAEGFLKLEN
>gi_38489219_gb_AAR21295.1__bacterial_spot_disease_resistance_protein
_4__Solanum_lycopersicum__205_469
NAHLEKLKSKLQIEINDVRILGIWGIGGVGKTRIAKAIFDTLSYQFEASCFLADVKEFAK
KNKLHSLQNILLSELLRKKNDYVYNKYDGKCMIPNRLCSLKVLIVLDDIDHGDQMEYLAG
DICWFGNGSRVIVTTRNKHLIEKDDAIYEVSTLPDHEAMQLFNMHAFKKEVPNEDFKELA
LEIVNHAKGLPLALKVWGCLLHKKNLSLWKITVEQIKKDSNSEIVEQLKISYDGLESEEQ
EIFLDIACFFRGEKRKEVMQILKSC
>gi_6164969_gb_AAF04603.1_AF195939_1_disease_resistance_protein_Gpa2_
_Solanum_tuberosum__gi_5911745_emb_CAB55838.1__NBS_LRR_protein__Solan
um_tuberosum_subsp._andigenum__145_420
RENEFEMMLDQLARGGRELEVVSIVGMGGIGKTTLAAKLYSDPYIMSRFDIRAKATVSQE
YCVRNVLLGLLSLTSDEPDYQLADQLQKHLKGRRYLVVIDDIWTTEAWDDIKLCFPDCDN
GSRILLTTRNVEVAEYASSGKPPHHMRLMNFDESWNLLHKKIFEKEGSYSPEFENIGKQI
ALKCGGLPLAITLIAGLLSKISKTLDEWQNVAENVRSVVSTDLEAKCMRVLALSYHHLPS
HLKPCFLYFAIFAEDERIYVNKLVELWAVEGFLNEE
>gi_37781226_gb_AAP44390.1__nematode_resistance_protein__Solanum_tube
rosum__199_456
ESHMHKVYKMLGIGSGGVHFLGILGMSGVGKTTLARVIYDNIRSQFQGACFLHEVRDRSA
KQGLERLQEILLSEILVVKKLRINDSFEGANMQKQRLQYKKVLLVLDDVDHIDQLNALAG
EREWFGDGSRIIITTKDKHLLVKYETEKIYRMKTLNNYESLQLFKQHAFKKNRPTKEFED
LSAQVIKHTDGLPLALKVLGSFLYGRGLDEWISEVERLKQIPENEILKKLEQSFTGLHNT
EQKIFLDIACFFSGKKKD
>gi_350536521_ref_NP_001233995.1__hero_resistance_protein__Solanum_ly
copersicum__gi_26190258_emb_CAD29728.1__hero_resistance_protein__Sola
num_lycopersicum__528_807
ARIIGQLLDEHESKLDVISIVGMPGVGKTTLANKVYNNTLVASHFKIRAKCTVSQNFNKS
KVLREILQQVTASETNRSEDDLAEKLRVALLDKRYLIVLDDVWDIATGEMLIACFPKVER
GNRVILTSRSGEVGLKVKCRSDPVDLQVLTDEKSWELFEKRVFRDEGSCPAELLDIGHQI
VEKCKGLPLALVLIAGVIVRGREGKEKEKEKDFWVKIQNNLDSFTSSNINSQIMNVMQSS
```

YDHLPYQLKPLLLYFARLQKSERTPVSMLMQLWMAEGLVD
>gi_4689223_gb_AAD27815.1_AF118127_1_disease_resistance_protein_I2__S
olanum_lycopersicum__173_456
RQSEIEDLIDRLLSEGASGKKLTVVPIVGMGGQGKTTLAKAVYNDERVKNHFDLKAWYCV
SEGFDALRITKELLQEIGKFDSKDVHNNLNQLQVKLKESLKGKKFLIVLDDVWNENYNEW
NDLRNIFAQGDIGSKIIVTTRKDSVALMMGNEQIRMGNLSTEASWSLFQRHAFENMDPMG
HPELEEVGRQIAAKCKGLPLALKTLAGMLRSKSEVEEWKRILRSEIWELPHNDILPALML
SYNDLPAHLKRCFSFCAIFPKDYPFRKEQVIHLWIANGLVPVKD
>gi_350537949_ref_NP_001234063.1__root_knot_nematode_resistance_prote
in__Solanum_lycopersicum__gi_3449380_gb_AAC67238.1__root_knot_nematod
e_resistance_protein__Solanum_lycopersicum__526_803
EEETNLILRKLTSGPADLDVISITGMPGSGKTTLAYKVYNDKSVSRHFDLRAWCTVDQGY
DDKKLLDTIFSQVSGSDSNLSENIDVADKLRKQLFGKRYLIVLDDVWDTTTLDELTRPFP
EAKKGSRIILTTREKEVALHGKLNTDPLDLRLLRPDESWELLDKRTFGNESCPDELLDVG
KEIAENCKGLPLVADLIAGVIAGREKKRSVWLEVQSSLSSFILNSEVEVMKVIELSYDHL
PHHLKPCLLHFASWPKDTPLTIYLFTVYLGAEGFVEKT
>gi_126571551_gb_ABO21407.1__TMV_resistance_protein_N__Nicotiana_taba
cum__192_449
DTHLEKIESLLGLEINDVRIMGIWGMGGVGKTTIARGMFDTLLGRRDSSYQFDGACFLKD
IKENKHGMHSLQNILLSNLLREKANYNNEEEGKHQMASRLRSKKVLIVLDDIDDKDHYLE
YLAGDLDWFGDGSRIIVTTRDKNLIEKNDVIYEVSALPVHESIQLLNQYAFGKKVPDEHF
KKLSLEVVNYAKGLPLALKVWGSLLHNLRLTEWRSAMEQMKNNSNSEIVEKLKISYDGLE
PIQQEMFLDIACFLRGEE
>gi_3947733_emb_CAA08797.1__NL25__Solanum_tuberosum__203_459
DTHLKEVKSLLEMESGDVRILGIWGMGGVGKTTLARAVFDTLSPRFQYASFLENVKETNI
NEIQNKLLSELLREDKKHVDNKTEGKRLMAKRLRFMKVLIVLDDINHCDHLEYLAGDLCW
FGSGSRIIATTRNREILGMNNVVHQVTTLLEPDAIQLFNHYAFKGLFSPDEHMKKLALEA
VSHAKGLPLALKLWGIWLNNKDKTLWREAVDMIRRESSEDVVNNLKISFEGLQDKEKTIF
LDIACFFRGMRKDKTIE
>gi_3947735_emb_CAA08798.1__NL27__Solanum_tuberosum__201_457
DTHFKNIRSLLAELQMSGVLIVGIWGMPGVGKTTIARAIFDRLSYQFEAVCFLADIKENK
CGMHSLQNILLSELLKEKDNCVNNKEDGRSLLAHRLRFKKVLVVLDDIDHIDQLDYLAGN
LDWFGNGSRIIATTRDKHLIGKNVVYELPTLHDHDAIKLFERYAFKEQVSDKCFKELTLE
VVSHAKGLPLALKVFGCFFHERDITEWRSAIKQIKNNPNSEIVEKLKISYDGLETIQQSI
FLDIACFLRGRRKDYVM
>gi_350535589_ref_NP_001234202.1__NRC1__Solanum_lycopersicum__gi_8363
0761_gb_ABC26878.1__NRC1__Solanum_lycopersicum__161_440
DDEAQTVIDRLLEGSGDLEVIPVVGMPGLGKTTLATKIFKHPKIEYEFFTRLWLYVSQSY
KTRELYLNIISKFTGNTKHCRDMSEKDLALKVQEILEEGGKYLIVLDDVWSTDAWDRIKI
AFPKNDKGNRVLLTTRDHRVARYCNRSPHDLKFLTDEESWILLEKRAFHKAKCLPELETN
GKSIARKCKGLPLAIVVIAGALIGKSKTIKEWEQVDQSVGEHFINRDQPNSCDKLVRMSY
DVLPYDWKACFLYFGTFPRGYLIPARKLIRLWIAEGFIQY
>gi_164598916_gb_ABY61745.1__resistance_protein_PSH_RGH6__Solanum_tub
erosum__146_420
ENEFEMMLDQLVRGGRELEVVSIVGMGGIGKTTLATKLYSDPCIMPRFDIRAKATVSQEY
CVRNVLQGLLSSISDEPDDQLADRLQKHLKGRRYLVVIDDIWTTETWDDIKLCFPDCNNG
SRILLTTRNVEVAEYASSGKPPHHMRLMNFEESWNLLYKKIFEKEGSYSPEFENIGKQIA
LKCGGLPLAITVIAGLLSKISKTLDEWQNVAENVSSVVSTDLEAQCMRVLALSYHHLPSH
LKSCFLYFAIFAEDEQIYVNNLVELWGVEGFLNEE

>gi_75248609_sp_Q8W1E0.1_R1A_SOLDE_RecName__Full_Late_blight_resistan
ce_protein_R1_A__Short_Protein_R1_gi_17432423_gb_AAL39063.1_AF447489_
1_late_blight_resistance_protein__Solanum_demissum__548_825
EDVIENLRKKLLNGTKGQDVISIHGMPGLGKTTLANSLYSDRSVFSQFDICAQCCVSQVY
SYKDLILALLRDAIGEGSVRRELHANELADMLRKTLLPRRYLILVDDVWENSVWDDLRGC
FPDVNNRSRIILTTRHHEVAKYASVHSDPLHLRMFDEVESWKLLEKKVFGEESCSPLLKN
VGLRIAKMCGQLPLSIVLVAGILSEMEKEVECWEQVANNLGSYIHNDSRAIVDKSYHVLP
CHLKSCFLYFGAFLEDRVIDISRLIRLWISEAFIKSSE
>gi_256260668_gb_ACU65456.1__R2_protein__Solanum_demissum__165_447
QDVVQKLLAQLLKAEPRRTVLSIHGMGGLGKTTLARKLYNSSAILNSFPTRAWICVSQEY
NTMDLLRNIIKSVQGRTKETLDLLERMTEGDLEIYLRDLLKERKYLVMVDDVWQKEAWDS
LKRAFPDSKNGSRVIITTRKQDVAERADDIGFVHKLRFLSQEESWDLFRKKLLDVRSMVP
EMENLAKDMVEKCRGLPLAIVVLSGLLSHKKGLNQWQKVKDHLWKNIKEDKSIEISNILS
LSYNDLSTALKQCFLYFGIFPEDQVVKADDIIRLWMAEGFIPR
>gi_57233497_gb_AAW48299.1__potato_late_blight_resistance_protein_R3a
__Solanum_tuberosum__180_462
RQNDIEDLIDRLLSEDASGKKRTVVPIVGMGGLGKTTLAKAVYNDERVQIHFGLKAWFCV
SEAFDAFRITKGLLQEIGSFDLKADDNLNQLQVKLKERLKGKKFLIVLDDVWNDNYNKWD
ELRNVFVQGDIGSKIIVTTRKESVALMMGNEQISMDNLSTESSWSLFKTHAFENMGPMGH
PELEEVGKQIAAKCKGLPLALKTLAGMLRSKSEVEEWKRILRSEIWELPHNDILPALMLS
YNDLPAHLKRCFSFCAIFPKDYPFRKEQVIHLWIANGLVPQED
>gi_264820947_gb_ACY74346.1__blight_resistance_protein_RGA2__Capsicum
_annuum__155_436
DKDKDKIVEILTKDVSGLQELSVLPILGMGGIGKTTLAQMVFNDQRVTEHFNPKIWICVS
EDFDEKRLIKAIVESIEGLLGAMDLAPLQKKLQELLNRERYFLVLDDVWNEDQQKWDNLR
AALNVGANGASVLTTTRLEMVGSIMGTLRPCKLSNLSEDHCWSLFRQRAFGNQEEISPSL
EAIGKKIVKKCGGVPLAAKTLGGLLRSKKEVRQWENVRDSEIWNLPQDENSILPALRLSC
HHLPVDSRRCFAYCATFIKDTKMEKKNLITLWMAHGYLEVED
>gi_46576968_sp_Q7XBQ9.1_RGA2_SOLBU_RecName__Full_Disease_resistance_
protein_RGA2__AltName__Full_Blight_resistance_protein_RPI__AltName__F
ull_RGA2_blb_gi_32693281_gb_AAP86601.1__putative_disease_resistant_pr
otein_RGA2__Solanum_bulbocastanum__gi_39636705_gb_AAR29069.1__blight_
resistance_protein_RPI__Solanum_bulbocastanum__155_434
DKEKDEIVKILINNVSDAQHLSVLPILGMGGLGKTTLAQMVFNDQRVTEHFHSKIWICVS
EDFDEKRLIKAIVESIEGRPLLGEMDLAPLQKKLQELLNGKRYLLVLDDVWNEDQQKWAN
LRAVLKVGASGASVLTTTRLEKVGSIMGTLQPYELSNLSQEDCWLLFMQRAFGHQEEINP
NLVAIGKEIVKKSGGVPLAAKTLGGILCFKREERAWEHVRDSPIWNLPQDESSILPALRL
SYHQLPLDLKQCFAYCAVFPKDAKMEKEKLISLWMAHGFL
>gi_15242354_ref_NP_199338.1__TIR_NBS_LRR_class_disease_resistance_pr
otein__Arabidopsis_thaliana__gi_5459305_emb_CAB50708.1__disease_resis
tance_protein_RPS4__Arabidopsis_thaliana__gi_10176996_dbj_BAB10246.1_
_disease_resistance_protein_like__Arabidopsis_thaliana__gi_332007838_
gb_AED95221.1__TIR_NBS_LRR_class_disease_resistance_protein__Arabidop
sis_thaliana__211_472
EQRLKDLEEKLDRDKYKGTRIIGVVGMPGIGKTTLLKELYKTWQGKFSRHALIDQIRVKS
KHLELDRLPQMLLGELSKLNHPHVDNLKDPYSQLHERKVLVVLDDVSKREQIDALREILD
WIKEGKEGSRVVIATSDMSLTNGLVDDTYMVQNLNHRDSLQLFHYHAFIDDQANPQKKDF
MKLSEGFVHYARGHPLALKVLGGELNKKSMDHWNSKMKKLAQSPSPNIVSVFQVSYDELT
TAQKDAFLDIACFRSQDKDYVE

>gi_206604098_gb_ACI16480.1__NBS_LRR_resistance_protein__Solanum_bulb
ocastanum__156_435
DKEKDEIVKILINNVSNAQTLPVLPILGMGGLGKTTLAQMVFNDQRVIEHFHPKIWICVS
EDFNEKRLIKEIVESIEEKSLGGMDLAPLQKKLRDLLNGKKYLLVLDDVWNEDQDKWAKL
RQVLKVGASGASVLTTTRLEKVGSIMGTLQPYELSNLSQEDCWLLFMQRAFGHQEEINLN
LVAIGKEIVKKCGGVPLAAKTLGGILRFKREERQWEHVRDSEIWKLPQEESSILPALRLS
YHHLPLDLRQCFTYCAVFPKDTEMEKGNLISLWMAHGFIL
>gi_74040324_gb_AAZ95005.1__late_blight_resistance_protein_Rpi_blb2__
Solanum_bulbocastanum__540_814
NLILRKLTSGPADLDVISIIGMPGLGKTTLAYKVYNDKSVSSHFDLRAWCTVDQVYDEKK
LLDKIFNQVSDSNSKLSENIDVADKLRKQLFGKRYLIVLDDVWDTNTWDELTRPFPDGMK
GSRIILTTREKKVALHGKLYTDPLNLRLLRSEESWELLEKRAFGNESCPDELLDVGKEIA
ENCKGLPLVVDLIAGIIAGREKKKSVWLEVVNNLHSFILKNEVEVMKVIEISYDHLPDHL
KPCLLYFASAPKDWVTTIHELKLIWGFEGFVEKTD
>gi_256260670_gb_ACU65457.1__Rpi_protein__Solanum_bulbocastanum__165_
447
QDVVQTLLAQLLKAEPRRSVLSIYGMGGLGKTTLARKLYTSPDILNSFPTRAWICVSQEY
NTMDLLRTIIKSIQGCAKETLDLLEKMAEIDLENHLRDLLKECKYLVVVDDVWQREAWES
LKRAFPDGKNGSRVIITTRKEDVAERVDHRGFVHKLRFLSQEESWDLFRRKLLDVRAMVP
EMESLAKDMVEKCRGLPLAIVVLSGLLSHKKGLNQWQKVKDHLWKNIKEDKSIEISNILS
LSYNDLSTALKQCFLYFGIFPEDQVVKADDIIRLWMAEGFIPR
>gi_215401993_gb_ACJ66596.1__late_blight_resistance_protein__Solanum_
venturii__208_484
EDDFNTLQAKLLDHDLPYGVVSIVGMPGLGKTTLAKKLYRHVCHQFECSGLVYVSQQPRA
GEILHDIAKQVGLTEEERKENLENNLRSLLKIKRYVILLDDIWDVEIWDDLKLVLPECDS
KIGSRIIITSRNSNVGRYIGGDFSIHVLQPLDSEKSFELFTKKIFNFVNDNWANASPDLV
NIGRCIVERCGGIPLAIVVTAGMLRARGRTEHAWNRVLESMAHKIQDGCGKVLALSYNDL
PIALRPCFLYFGLYPEDHEIRAFDLTNMWIAEKLIVV
>gi_5524754_emb_CAB50786.1__Rx_protein__Solanum_tuberosum__145_420
RENEFEMMLDQLARGGRELEVVSIVGMGGIGKTTLATKLYSDPCIMSRFDIRAKATVSQE
YCVRNVLLGLLSLTSDEPDDQLADRLQKHLKGRRYLVVIDDIWTTEAWDDIKLCFPDCYN
GSRILLTTRNVEVAEYASSGKPPHHMRLMNFDESWNLLHKKIFEKEGSYSPEFENIGKQI
ALKCGGLPLAITVIAGLLSKMGQRLDEWQRIGENVSSVVSTDPEAQCMRVLALSYHHLPS
HLKPCFLYFAIFTEDEQISVNELVELWPVEGFLNEE
>gi_15418709_gb_AAG31013.1__tospovirus_resistance_protein_A__Solanum_
lycopersicum__538_816
EKQAEELIDYLTRGTNELDVVPIVGMGGQGKTTIARKLYNNDIIVSRFDVRAWCIISQTY
NRIELLQDIFSQVTGFNDNGATVDVLADMLRRKLMGKRYLIVLDDMWDCMVWDDLRLSFP
DVGIRSRIVVTTRLEEVGKQVKYHTDPYSLPFLTTEESCQLLQKKVFQKEDCPLELQDVS
QAVAEKCKGLPLVVVLVAGIIKKRKMEESWWNEVKDALFDYLDSEFEEYSLATMQLSFDN
LPHCLKPCLLYMGMFSEDARIPASTLISLWIAEGFVENT
>gi_119866045_gb_ABM05492.1__Tm_2_ToMV_resistance_protein__Solanum_tu
berosum__164_440
DAFNTLQAKLLDQDLPYGVVSIVGMPGLGKTTLAKKLYRHVRHQFESSGLVYVSQQPRAG
EILHDIAKQVGLPEEERKENLENNLRSLLKIKRYVILLDDIWDVEIWDHLKLVLPERDSK
IGSRIIITSRNSNVGRYIGGDFSIHVLQPLDSENSFELFTKKIFTFDNNNNWDSASPDWV
DIGRSIVGRCGGIPLAIVVTAGILRARERTERAWNRVLDSMGHKVQDGCAKVLALSYNDL
PIALRPCFLYFGLYPEDHEIHAFDLTNMWIAEKLIVV
>gi_16944811_emb_CAC82811.1__resistance_gene_like__Solanum_tuberosum_

subsp._andigenum__209_456
KKVNSLLEMKIDDVRIVWIWGMGGVGKTTIARAIFDILSSKFDGACFLPDNKENKYEIHS
LQSILLSKLVGEKENCVHDKEDGRHLMARRLRLKKVLVVLDNIDHEDQLKYLAGDLGWFG
NGTRIIATTRDKHFIRKNDAVYPVTTLLEHDAVQLFNQYAFKNEVPDKCFEEITLEVVSH
AEGLPLALKVWGSSLHKKDIHVWRSAVDRIKRNPSSKVVENLKVSYDGLEREDQEIFLDI
ACFLRGRK
>gi_21536933_gb_AAC05834.2__cyst_nematode_resistance_gene_candidate_l
ike_protein__Aegilops_tauschii__gi_22252946_gb_AAM94164.1__go35_NBS_L
RR__Aegilops_tauschii__210_480
FSVIGIHGVSGSGKSTLAQFVYAHEKNDKQDNKEDHFDLVMWVHVSQDFSVWGIFKELYE
AASDPKVPCPQFNNLNALEEELERKLDGKRFLLVLDDVWCNADVGNQELPKLLSPLKKGK
KGSKILVTTRSKYALPDLCPGVRYTAMPITEVDDTAFFELFMHYALEDGQDQSMFQNIGV
EIAKKLKGSPLAARTVGGNLRRQQDVDHWRRVGDQDLFKVWTGPLWWSYYQLGEQARRCF
AYCSIFPRRHRLYRDELVRLWMAEGFIRNTD
>gi_7110565_gb_AAF36987.1_AF234174_1_viral_resistance_protein__Arabid
opsis_thaliana__170_456
EQSVTELVCHLVENDVHQVVSIAGMGGIGKTTLARQVFHHDLVRRHFDGFAWVCVSQQFT
QKHVWQRILQELQPHDGDILQMDESALQPKLFQLLETGRYLLVLDDVWKKEDWDRIKAVF
PRKRGWKMLLTSRNEGVGIHADPTCLTFRASILNPEESWKLCERIVFPRRDETEVRLDEE
MEAMGKEMVTHCGGLPLAVKVLGGLLANKHTVPEWKRVSDNIGSQIVGGSCLDDNSLNSV
YRILSLSYEDLPTHLKHRFLFLAHFPEDSKITTQELFYYWAAEGIYD
>gi_33302327_gb_AAQ01784.1__resistance_protein_LR10__Triticum_aestivu
m__gi_195975980_gb_ACG63536.1__resistance_protein_RGA2__Triticum_duru
m__174_455
HELVKWLRNGEDESVHQQKVVSIVGCAGLGKTTLAKQVYDELRINFEYRAFVSISRSPNM
ATILKCVLSQFHAQDYSSDESEIPKLVDQIRDLLQDKRYFVIIDDIWDMKTWDVLKCALC
KNSCGSVIMTTTRIYDVAKSCCSSNGDLVYNIQPLSVADSEELFLNRVFGHEKGFPPELK
EVSKDVLRKCGGLPLAINAISSLLAAEKIEEWDRVGLSNVFAQGEKSDIDAMKYKLSLCY
FDLPLHLRSCLLYLIMFPEDCLIEKERLVHRWISEGFIRNED
>gi_270267805_gb_ACZ65507.1__MLA1__Hordeum_chilense__186_457
EKRLKKVSIVGFGGLGKTTLARAVYDKIKGDFDCGAFVPVGQNPDMKKVLRDILIDLGNP
LSDLAMLDANQLIKKLRDFLENKRYLVIIDDIWDDKLWEAINFAFSKRNNLGSRLIITTR
IVSVSNSCCSSAHDSVYQMKPLSTDDSRRLFHKRIFPDDSGCPNEFEQVSEDLLKKCGGV
PLAIITIASALAGGQQMKPKCEWDILLQSLGSGLTEDNSLEEMRRILSFSYYDLPYHLRT
CLLYLCIYPEDSQIDRDRLIWKWVAEGFVHCG
>gi_33943720_gb_AAQ55541.1__MLA10__Hordeum_vulgare__191_463
NKRLKKVSIVGFGGLGKTTLARAVYEKIKGDFDCRAFVPVGQNPDMKKVLRDILIDLGNP
HSDLAMLDANQLIKKLHEFLENKRYLVIIDDIWDEKLWEGINFAFSNRNNLGSRLITTTR
IVSVSNSCCSSDGDSVYQMEPLSVDDSRMLFYKRIFPDENACINEFEQVSRDILKKCGGV
PLAIITIASALAGDQKMKPKCEWDILLRSLGSGLTEDNSLEEMRRILSFSYSNLPSNLKT
CLLYLCVYPEDSMISRDKLIWKWVAEGFVHHEN
>gi_28565622_gb_AAO43441.1__MLA12__Hordeum_vulgare_subsp._vulgare__19
1_463
NKRLKKVSIVGFGGLGKTTLARAVYEKIKGDFDCRAFVPVGQNPDMKKVLRDILIDLGNP
HSDLAMLDANQLIKKLHEFLENKRYLVIIDDIWDEKLWEGINFAFSNRNNLGSRLITTTR
IVSVSNSCCSSDGDSVYQMEPLSVDDSRMLFYKRIFPDENACINEFEQVSRDILKKCGGV
PLAIITIASALAGDQKMKPKCEWDILLRSLGSGLTEDNSLEEMRRILSFSYSNLPSNLKT
CLLYLCVYPEDSMISRDKLIWKWVAEGFVHHEN
>gi_12957124_emb_CAC29241.1__MLA6_protein__Hordeum_vulgare_subsp._vul

gare__gi_12957126_emb_CAC29242.1__MLA6_protein__Hordeum_vulgare_subsp._vulgare__191_463
NKRLKKVSIVGFGGLGKTTLARAVYEKIKGDFDCRAFVPVGQNPDMKKVLRDILIDLGNP
HSDLAMLDANQLIKKLHEFLENKRYLVIIDDIWDEKLWEGINFAFSNRNNLGSRLITTTR
IVSVSNSCCSSDGDSVYQMEPLSVDDSRMLFSKRIFPDENGCINEFEQVSRDILKKCGGV
PLAIITIASALAGDQKMKPKCEWDILLRSLGSGLTEDNSLEEMRRILSFSYSNLPSHLKT
CLLYLCVYPEDSMISRDKLIWKWVAEGFVHHEN
>gi_86361429_gb_ABC94599.1__NBS_LRR_type_R_protein__Nbs4_Pi__Oryza_sativa_Indica_Group__174_462
LEMIDTNANDGPAKVICVVGMGGLGKTALSRKIFESEEDIRKNFPCNAWITVSQSFHRIE
LLKDMIRQLLGPSSLDQLLQELQGKVVVQVHHLSEYLIEELKEKRYFVVLDDLWILHDWN
WINEIAFPKNNKKGSRIVITTRNVDLAEKCATASLVYHLDFLQMNDAITLLLRKTNKNHE
DMESNKNMQKMVERIVNKCGRLPLAILTIGAVLATKQVSEWEKFYEHLPSELEINPSLEA
LRRMVTLGYNHLPSHLKPCFLYLSIFPEDFEIKRNRLVGRWIAEGFVRP
>gi_114329518_gb_ABI64281.1__CC_NBS_LRR_Pi36__Oryza_sativa_Indica_Group__191_462
NLNTSNRKTKIVYVVGMGGLGKTTLATAVYEKIKVGFPLNAFVPIGQNPNMKAILWNILH
RLGSEKYLNCPNIEMLTVQELIGELKQFIKGKRFFIVIDDIWDKPSWQILESGLQDNDYG
SKILVTTRKSEVATIISDVYNMKPLSHDNSKELLYTRTGSEGKSLDSSSTEACDKILKKC
AGVPLAIITIASLLASRSGLDWSEVYRAIDFGEEDNYEMANTKRILSFSYYDLPSHLKNC
LLYLSMFPEDYKIDKNHLIWMWIAEGFVPEKQ
>gi_6172381_dbj_BAA85975.1__Pi_b_protein__Oryza_sativa__japonica_cultivar_group___gi_37777009_dbj_BAA76281.2__Pib__Oryza_sativa_Japonica_Group__gi_37777304_dbj_BAA76282.2__Pib__Oryza_sativa_Japonica_Group__174_709
QLINSEDQDLKVIAVWGTSGDMGQTTIIRMAYENPDVQIRFPCRAWVRVMHPFSPRDFVQ
SLVNQLHATQGVEALLEKEKTEQDLAKKFNGCVNDRKCLIVLNDLSTIEEWDQIKKCFQK
CRKGSRIIVSSTQVEVASLCAGQESQASELKQLSADQTLYAFYDKGSQIIEDSVKPVSIS
DVAITSTNNHTVAHGEIIDDQSMDADEKKVARKSLTRIRTSVGASEESQLIGREKEISEI
THLILNNDSQQVQVISVWGMGGLGKTTLVSGVYQSPRLSDKFDKYVFVTIMRPFILVELL
RSLAEQLHKGSSKKEELLENRVSSKKSLASMEDTELTGQLKRLLEKKSCLIVLDDFSDTS
EWDQIKPTLFPLLEKTSRIIVTTRKENIANHCSGKNGNVHNLKVLKHNDALCLLSEKVFE
EATYLDDQNNPELVKEAKQILKKCDGLPLAIVVIGGFLANRPKTPEEWRKLNENINAELE
MNPELGMIRTVLEKSYDGLPYHLKSCFLYLSIFPEDQIISRRRLVHRWAAEGYSTA
>gi_47027820_gb_AAT08955.1__CC_NBS_LRR__Helianthus_annuus__170_449
EGEKKRLLNQLFVGESSKENFIIVPIVGMGGVGKTTLARMLYNDTRVKVHFELMAWVCVS
DEFDIFKISQTTYQSVAKESKQFTDTNQLQIALKEKLEGKRFLVVLDDVWNENYDDWENL
VRPFHSGATGSRVIMTTRQQQLLKKMGFNHLDLLESLSHDDALSLLARHALDVDNFDSHE
TLKPLGEGIVEKCGCLPLALKAIGRLMRAKTEEEEWSDVLNSEIWDLESADEIVPALRLS
YHDLSADLKRLFAYCSLFPKDFLFEKEELVLLWVAEGYLN
>gi_37624724_gb_AAQ96158.1__powdery_mildew_resistance_protein_PM3b__Triticum_aestivum__181_458
EDKKNIIGILVDEASNADLTVVPVVAMGGLGKTTLAQLIYNDPEIQKHFQLLLWVCVSDT
FDVNSLAKSIVEASPNKNVDTDKPPLARLQKLVSGQRYLLVLDDVWDNKELRKWERLKVC
LQHGGMGSAVLTTTRDKRVAEIMGADRAAYNLNALEDHFIKEIIVDRAFSSENGKIPELL
EMVGEIVKRCCGSPLAASALGSVLRTKTTVKEWNAIASRSSICTEETGILPILKLSYNDL
PSHMKQCFAFCAVFPKDYKIDVAKLIQLWIANGFIPEH
>gi_5702196_gb_AAD47197.1_AF107293_1_rust_resistance_protein__Zea_mays__214_477

GLAIVGLGGMGKSTLAQYVYNDKRIEECFDIRMWVCISRKLDVHRHTREIIESAKKGECP
RVDNLDTLQCKLRDILQESQKFLLVLDDVWFEKSHNETEWELFLAPLVSKQSGSKVLVTS
RSKTLPAAICCEQEHVIHLKNMDDTEFLALFKHHAFSGAEIKDQVLRTKLEDTAVEIAKR
LGQCPLAAKVLGSRLCRKKDIAEWKAALKIGDLSDPFTSLLWSYEKLDPRLQRCFLYCSL
FPKGHRYESNELVHLWVAEGFVGS
>gi_15231371_ref_NP_187360.1__disease_resistance_protein_RPM1__Arabidopsis_thaliana__gi_29839510_sp_Q39214.1_RPM1_ARATH_RecName__Full_Disease_resistance_protein_RPM1__AltName__Full_Resistance_to_Pseudomonas_syringae_protein_3_gi_6729011_gb_AAF27008.1_AC016827_19_disease_resistance_gene__RPM1___Arabidopsis_thaliana__gi_963017_emb_CAA61131.1__RPM1__Arabidopsis_thaliana__gi_332640971_gb_AEE74492.1__disease_resistance_protein_RPM1__Arabidopsis_thaliana__177_465
APKGKLIGRLLSPEPQRIVVAVVGMGGSGKTTLSANIFKSQSVRRHFESYAWVTISKSYV
IEDVFRTMIKEFYKEADTQIPAELYSLGYRELVEKLVEYLQSKRYIVVLDDVWTTGLWRE
ISIALPDGIYGSRVMMTTRDMNVASFPYGIGSTKHEIELLKEDEAWVLFSNKAFPASLEQ
CRTQNLEPIARKLVERCQGLPLAIASLGSMMSTKKFESEWKKVYSTLNWELNNNHELKIV
RSIMFLSFNDLPYPLKRCFLYCSLFPVNYRMKRKRLIRMWMAQRFVEPI
>gi_15231449_ref_NP_190237.1__disease_resistance_protein_RPP13__Arabidopsis_thaliana__gi_29839653_sp_Q9M667.2_RPP13_ARATH_RecName__Full_Disease_resistance_protein_RPP13__AltName__Full_Resistance_to_Peronospora_parasitica_protein_13_gi_7229449_gb_AAF42830.1_AF209730_1_RPP13__Arabidopsis_thaliana__gi_6523056_emb_CAB62323.1__putative_protein__Arabidopsis_thaliana__gi_14335000_gb_AAK59764.1__AT3g46530_F12A12_50__Arabidopsis_thaliana__gi_25090228_gb_AAN72257.1__At3g46530_F12A12_50__Arabidopsis_thaliana__gi_332644648_gb_AEE78169.1__disease_resistance_protein_RPP13__Arabidopsis_thaliana__169_450
DAKILLEKLLDYEEKNRFIISIFGMGGLGKTALARKLYNSRDVKERFEYRAWTYVSQEYK
TGDILMRIIRSLGMTSGEELEKIRKFAEEELEVYLYGLLEGKKYLVVVDDIWEREAWDSL
KRALPCNHEGSRVIITTRIKAVAEGVDGRFYAHKLRFLTFEESWELFEQRAFRNIQRKDE
DLLKTGKEMVQKCRGLPLCIVVLAGLLSRKTPSEWNDVCNSLWRRLKDDSIHVAPIVFDL
SFKELRHESKLCFLYLSIFPEDYEIDLEKLIHLLVAEGFIQG
>gi_15239876_ref_NP_199160.1__disease_resistance_protein_RPP8__Arabidopsis_thaliana__gi_30694301_ref_NP_851124.1__disease_resistance_protein_RPP8__Arabidopsis_thaliana__gi_29839585_sp_Q8W4J9.2_RPP8_ARATH_RecName__Full_Disease_resistance_protein_RPP8__AltName__Full_Resistance_to_Peronospora_parasitica_protein_8_gi_3901294_gb_AAC78631.1__rpp8__Arabidopsis_thaliana__gi_8843900_dbj_BAA97426.1__disease_resistance_protein_RPP8__Arabidopsis_thaliana__gi_332007584_gb_AED94967.1__disease_resistance_protein_RPP8__Arabidopsis_thaliana__gi_332007585_gb_AED94968.1__disease_resistance_protein_RPP8__Arabidopsis_thaliana__169_455
EQSVKELVGHLVENDVHQVVSIAGMGGIGKTTLARQVFHHDLVRRHFDGFAWVCVSQQFT
QKHVWQRILQELQPHDGDILQMDEYALQRKLFQLLEAGRYLVVLDDVWKKEDWDVIKAVF
PRKRGWKMLLTSRNEGVGIHADPTCLTFRASILNPEESWKLCERIVFPRRDETEVRLDEE
MEAMGKEMVTHCGGLPLAVKALGGLLANKHTVPEWKRVFDNIGSQIVGGSWLDDNSLNSV
YRILSLSYEDLPTHLKHCFLNLAHFPEDSEISTYSLFYYWAAEGIYD
>gi_62632823_gb_AAX89382.1__NB_LRR_type_disease_resistance_protein_Rps1_k_1__Glycine_max__160_444
REKDREAIIKLLSEDNSDGSEVSVVPIVGMGGVGKTTLAQLVYNDENLKEKFDFDFKAWV
CVSQEFDVLKVTKTIIQAVTGNPCKLNDLNLLHLELMDKLKDKKFLIVLDDVWTEDYVDW

SLLKKPFQCGIIRRSKILLTTRSEKTASVVQTVQTYHLNQLSNEDCWSVFANHACLSLES
NENTTLEKIGKEIVKKCDGLPLAAQSLGGMLRRKHDIGDWYNILNSDIWELSESECKVIP
ALRLSYHYLPPHLKRCFVYCSLYPQDYEFDKNELILLWMAEDLLK
>gi_62632825_gb_AAX89383.1__NB_LRR_type_disease_resistance_protein_Rps1_k_2__Glycine_max__160_445
REKDKEAIIKLLSEDNSDGREVSVVPIVGMGGVGKTTLAQLVYNDENLKQIFDFDFKAWV
CVSQEFDVLKVTKTIIEAVTGKACKLNDLNLLHLELMDKLKDKKFLIVLDDVWTEDYVDW
RLLKKPFNRGIIRRSKILLTTRSEKTASVVQTVHTYHLNQLSNEDCWSVFANHACLSTES
NENTATLEKIGKEIVKKCNGLPLAAESLGGMLRRKHDIGDWNNILNSDIWELSESECKVI
PALRLSYHYLPPHLKRCFVYCSLYPQDYEFEKNELILLWMAEDLLK
>gi_15236112_ref_NP_194339.1__disease_resistance_protein_RPS2__Arabidopsis_thaliana__gi_30173240_sp_Q42484.1_RPS2_ARATH_RecName__Full_Disease_resistance_protein_RPS2__AltName__Full_Resistance_to_Pseudomonas_syringae_protein_2_gi_22087185_gb_AAM90869.1_AF487807_1_RPS2__Arabidopsis_thaliana__gi_22087187_gb_AAM90870.1_AF487808_1_RPS2__Arabidopsis_thaliana__gi_22087191_gb_AAM90872.1_AF487810_1_RPS2__Arabidopsis_thaliana__gi_548086_gb_AAA21874.1__RPS2__Arabidopsis_thaliana__gi_549979_gb_AAA50236.1__RPS2__Arabidopsis_thaliana__gi_4538938_emb_CAB39674.1__disease_resistance_protein_RPS2__Arabidopsis_thaliana__gi_7269460_emb_CAB79464.1__disease_resistance_protein_RPS2__Arabidopsis_thaliana__gi_26449528_dbj_BAC41890.1__putative_disease_resistance_protein_RPS2__Arabidopsis_thaliana__gi_29029056_gb_AAO64907.1__At4g26090__Arabidopsis_thaliana__gi_332659756_gb_AEE85156.1__disease_resistance_protein_RPS2__Arabidopsis_thaliana__159_439
TTMMEQVLEFLSEEEERGIIGVYGPGGVGKTTLMQSINNELITKGHQYDVLIWVQMSREF
GECTIQQAVGARLGLSWDEKETGENRALKIYRALRQKRFLLLLDDVWEEIDLEKTGVPRP
DRENKCKVMFTTRSIALCNNMGAEYKLRVEFLEKKHAWELFCSKVWRKDLLESSSIRRLA
EIIVSKCGGLPLALITLGGAMAHRETEEEWIHASEVLTRFPAEMKGMNYVFALLKFSYDN
LESDLLRSCFLYCALFPEEHSIEIEQLVEYWVGEGFLTSSH
>gi_15221252_ref_NP_172686.1__disease_resistance_protein_RPS5__Arabidopsis_thaliana__gi_334182494_ref_NP_001184970.1__disease_resistance_protein_RPS5__Arabidopsis_thaliana__gi_46396675_sp_O64973.2_RPS5_ARATH_RecName__Full_Disease_resistance_protein_RPS5__AltName__Full_Resistance_to_Pseudomonas_syringae_protein_5__AltName__Full_pNd3_pNd10_gi_10086512_gb_AAG12572.1_AC022522_5_resistance_to_Pseudomonas_syringae_protein_5__Arabidopsis_thaliana__gi_3309620_gb_AAC26126.1__resistance_to_Pseudomonas_syringae_protein_5__Arabidopsis_thaliana__gi_34849895_gb_AAQ82844.1__At1g12220__Arabidopsis_thaliana__gi_62319935_dbj_BAD94018.1__NBS_LRR_disease_resistance_protein__Arabidopsis_thaliana__gi_77632414_gb_ABB00204.1__disease_resistance_protein__Arabidopsis_thaliana__gi_332190730_gb_AEE28851.1__disease_resistance_protein_RPS5__Arabidopsis_thaliana__gi_332190731_gb_AEE28852.1__disease_resistance_protein_RPS5__Arabidopsis_thaliana__161_442
EIMLEKAWNRLMEDGSGILGLYGMGGVGKTTLLTKINNKFSKIDDRFDVVIWVVVSRSST
VRKIQRDIAEKVGLGGMEWSEKNDNQIAVDIHNVLRRRKFVLLLDDIWEKVNLKAVGVPY
PSKDNGCKVAFTTRSRDVCGRMGVDDPMEVSCLQPEESWDLFQMKVGKNTLGSHPDIPGL
ARKVARKCRGLPLALNVIGEAMACKRTVHEWCHAIDVLTSSAIDFSGMEDEILHVLKYSY
DNLNGELMKSCFLYCSLFPEDYLIDKEGLVDYWISEGFINEK
>gi_1842251_gb_AAB47618.1__rust_resistance_protein_M__Linum_usitatiss

imum__256_533
DDHVEVILEMLSLDSKSVTMVGLYGMGGIGKTTTAKAVYNKISSHFDRCCFVDNVRAMQE
QKDGIFILQKKLVSEILRMDSVGFTNDSGGRKMIKERVSKSKILVVLDDVDEKFKFEDIL
GCPKDFDSGTRFIITSRNQNVLSRLNENQCKLYEVGSMSEQHSLELFSKHAFKKNTPPSD
YETLANDIVSTTGGLPLTLKVTGSFLFRQEIGVWEDTLEQLRKTLDLDEVYDRLKISYDA
LKAEAKEIFLDIACFFIGRNKEMPYYMWSECKFYPKSN
>gi_13517468_gb_AAK28805.1_AF310960_1_resistance_like_protein_P2_A__L
inum_usitatissimum__193_453
ERLLAMDKLDDTCIIGLWEMGGVGKTTLAEACYDRVTSSNKGIKHLFVRNVNEICEKHHG
VEKIVHKLYSKLLDENNIDREDLNIGYRRERLSRSRVFVVLDNVETLEQLALGYVFNLSK
VFAAGSRIIITTRNKKVLQNAMAKIYNVECLNDEESTRLFSLHAFKQDRPQDNWMGKSRL
ATSYCKGNPLALKILGGALYGEDIHYWRSFLTGLRQPGNLGIENILRRSYDKLGKEEKKI
FMDVACLLYGMSRSRLIDYMA
>gi_30692151_ref_NP_190034.2__TIR_NBS_LRR_class_disease_resistance_pr
otein__Arabidopsis_thaliana__gi_332644385_gb_AEE77906.1__TIR_NBS_LRR_
class_disease_resistance_protein__Arabidopsis_thaliana__280_535
DMLEQLLRLDLDEVRMIGIWGPPGIGKTTIARFLFNQVSDRFQLSAIMVNIKGCYPRPCF
DEYSAQLQLQNQMLSQMINHKDIMISHLGVAQERLRDKKVFLVLDEVDQLGQLDALAKET
RWFGPGSRIIITTEDLGVLKAHGINHVYKVEYPSNDEAFQIFCMNAFGQKQPHEGFDEIA
WEVTCLAGELPLGLKVLGSALRGKSKREWERTLPRLKTSLDGKIGSIIQFSYDVLCDEDK
YLFLYIACLFNGESTT
>gi_27466164_gb_AAN86124.1__TIR_NBS_LRR__Arabidopsis_thaliana__190_44
7
EAHLTELKSLLSLESDEVKMIGIWGPAGIGKTTIARALFDRLSSIFPLICFMENLKGSLT
GVADHDSKLRLQNQLLSKILNQENMKIHHLGAIRERLHDQRVLIILDDVDDLEQLEVLAE
DPSWFGSGSRIIVTTEDKKILKAHRIKDIYHVNFPSKKEALEILCLSTFKQSSIPDGFEE
LANKVAELCGNLPLGLRVVGSSLRGESKQEWELQLSSIEASLDGKIETTLKVGYERLSKK
NQSLFLHIACFFNNQEVD
>gi_145334739_ref_NP_001078715.1__putative_WRKY_transcription_factor_
52__Arabidopsis_thaliana__gi_10176997_dbj_BAB10247.1__disease_resista
nce_protein_like__Arabidopsis_thaliana__gi_332007840_gb_AED95223.1__p
utative_WRKY_transcription_factor_52__Arabidopsis_thaliana__170_421
IGIRCVGIWGMPGIGKTTLAKAVFDQMSSAFDASCFIEDYDKSIHEKGLYCLLEEQLLPG
NDATIMKLSSLRDRLNSKRVLVVLDDVRNALVGESFLEGFDWLGPGSLIIITSRDKQVFC
LCGINQIYEVQGLNEKEARQLFLLSASIKEDMGEQNLQELSVRVINYANGNPLAISVYGR
ELKGKKKLSEMETAFLKLKRRPPFKIVDAFKSTYDTLSDNEKNIFLDIACFFQGENVNYV
IQLLEGCGFFPH

#PD NACHT

>E2Q8G7/312-475
RRTLLRGEAGAGKTTLVWWLAAHAARGTLPDRLDALNGLVPFVVPLRSVHAHGRGFPAPD
ALVHAAGLSVGTPPEGWAERVLASGRALLLVDGFDELPRAHRADARRWLAGLLRRYGATR
VLATVRPGAVEAKWLADEGFADLLLLPMSDGDIEMFIAAWHRAA
>B5GW02/336-505
GLLAVTGLAGTGKTSLLGRLALTSLPSWRETLGLDLDPATLPRAGTIHAALSCRGQSARS
LAAHLREVLADIDGAPSMPEEPVTSEKFTDAFKELVGRAGSVNLVFDALDEALPDQAHVI
ARHVLGPLANTPGVRVIVGTRTQPRRRITAPAEEESLLDALELSVEPVVL
>B5GNN6/270-436
DRVLLRGGAGSGKTTLVQWLAVTAARQKYEEFGDHLDHLVGRIPFVLPLRRVIRDGRPPT
PDEFLRAVRSVLAGAEPPGWTDRVLSAGRALVLVDGIDEIPQRERETTRRWLRELMADFP
GNLWLVTARPSAVDADWLAAEGFTELTLATMSHDDVTRFVHRWHTAA
>E2Q5Y6/159-318
RLVVLGEPGAGKTVLLLQLALDLLAVRAPDDPVPVIVELAGWNPETQRFDDWLEERLIAG
YPATATRDGSGVTRARALVDQGMVLPLLDGLDELPAPLSHQAVLTLNRSLDHHRPVVLTC
RARDWAAVVEAADVLTSAAVVELRPLSWETVGTHLSRTAR
>E2PW53/77-214
LRQEAVRSAEDPEGPVPFLLPEHALADISGLSAPDSLLAACGSPLAMEEPPGWVERILLT
GRALLLIDGVEGFAPPVLEEARTVLAGLLERYPAVSVTVTARLDAVPGSWLAAEGFTEYR
LCPLAPDEISAFIDSWDF
>B5GRK3/160-297
LRQEAVRSAEDPEGPVPFLLPEHALADISGLSAPDSLLAACGSPLAMEEPPGWVERILLT
GRALLLIDGVEGFAPPVLEEARTVLAGLLERYPAVSVTVTARLDAVPGSWLAAEGFTEYR
LCPLAPDEISAFIDSWDF
>B5H3I0/248-411
RRTLLRGEAGAGKTTLVWWLAAHAARGTLPDRLDALNGLVPFVVPLRSVHAHGRGFPAPD
ALVHAAGLSVGTPPEGWAERVLASGRALLLVDGFDELPRAHRADARRWLAGLLRRYGATR
VLATVRPGAVEAKWLADEGFADLLLLPMSDGDIEMFIAAWHRAA
>E2Q164/249-415
DRVLLRGGAGSGKTTLVQWLAVTAARQKYEEFGDHLDHLVGRIPFVLPLRRVIRDGRPPT
PDEFLRAVRSVLAGAEPPGWTDRVLSAGRALVLVDGIDEIPQRERETTRRWLRELMADFP
GNLWLVTARPSAVDADWLAAEGFTELTLATMSHDDVTRFVHRWHTAA
>E2Q891/252-408
RLLLRGGPGAGKTVLAHWLAARAAADRDTGRIPFLVPLRLLGAQEAMPETVEEFLATAPG
LPLERLHDGWIDRVLAEGRGLVVVDGLDEVHTAVRAAVGRRLAELLAVYPGTRCVVLTRP
RAVRDGWLARHGFTEATLLPLTPDGVSELVRRWCADD
>D8I898/124-304
GRLVVVGGPGAGKSILATCLCIDLAAAAIGNPSGEPVPVKLSLLRFNPGGSPHDQPGSVA
ARLLEAWIVRSLTEMYGIEAKMAARLVGERRIVPVLDGLDEMDARQYRPARAAELVRALN
HFSSGELPRFVLTSRPGCFDRLTKPPGDQEAVPLQHATVIELEPLKRADVIAYLKYRFPD
P
>D8I0Z0/143-308
RLCVVVGTPGGGKTSLFRTHLIRAVEARKAGGLETPLPILVPAAALSGKPLAAALAEAAT
AELSSFGLLENLPDALFKECPYEDVPWLVMVDGLDEITDPVARRNLLAALEQISDGSRGR
QYRFVIATRPLPEWELAVLGAQAPVYELEPFTWSDLNRVSADWFRV
>D8I7C2/200-356
RRFVVVGHPGAGKSTFIRNLLYRVAGQEVETVSAPMIVELKDHPSPSDSYLGILAESLRV
VTQSELTADTLRDVLQLGLAVVVFDGLDEITDINLRRSAVTAIEMFSRRYPLVTVVVTSR

EEGYLRARLDSASFPVYFLPDFTDEQLQHYVERWFKI
>D8HQY7/277-439
ARILIRGEAGSGKSTLLRWLAVNAARGTFRDELAGLNGYVPFLIKLRSYSGRRLPSPEEF
LDGTAQALTALMPSAYVHRQLASGRALVLVDGVDELTADERGAVRAWLKDLCQAFPESRL
VLTSRPAAAAETWLSAEGFSSAWIEHMSQADVRALVRHWHKAV
>D8HVK3/179-313
RRLVIAGEPGMGKTTLAVLLVRELLDHPEPHDPVPVLLSMSGWNPDAESLHEWMSRRLAE
DYPALRATAFGPDAARSLVTQHRILPILDGLDELPAQTRPKIIVRLNEVATDPLVLTCRT
AEYEAAVAAPGGDVL
>D8HSW9/281-449
SQVLVLGDPGTGKTTLLRYLALRHARAVLKGESVQGRPARLPIYVRIGDYARQGYPRVGI
SDFLPDYLNRSECRLPGLANMLGQQLEAGRCLVLLDGLDEVASAELRREVVAAVVNFVAA
HSRSGNRFVVTSRVAGYQAAPLPQPFTAMRLDDMDDDTISQFLQVYCRQ
>D8HSM6/284-444
PRILIRGEAGSGKTTLLDWLAVTAARTGFTGKLAEWNGRVPFPIRLRSFAADPLPRPEEF
VRHIAPVLAPPEGWARRVLAAGRAMILVDGVDEVGANHRREVKAWLRELSLAFEDTLFVV
TSRTAAADQRWLAQEGFGCVLLEPMSSDDIEALVARWHKAA
>D8HPW9/322-484
RQLVVLGAPGSGKSVLALMLTLELLRTRTPDAPVPVLLSLASWDPRREHLDRWLAARLAD
DHPALLNAREYGTDAPTRLVLGGHVVPVLDGLDEMPADLRIAALDALDQTMGAGRSLVLT
CRSAEYEQATRESGTVLGAATVVRLEPVVRQEAITYLAARQGE
>A9GM89/418-567
VLTMLSPSGFGKTSLALRLRQRCEDEGRPVLLINFRSFGRLPKDDGENRGYVTFLNDLAR
VFCKGLKLPLPTQPLESPMDFEEVLMMALEDMPRLVLILDGVDRVIERPYAEEFFSSIRS
WIDAVHLSCSFVLCIATEPSDLMTDIARSP
>A9G5J6/572-722
QRVFLWGQAGTGKSTLLQWLACRAARASREIEEARLPVWIPRLHGRSGDDLPGRLITLAF
EALHLPIDRRSPLYRALEGRISSGRAHLFIDSLDEASNVAQALLTKLHSRIHVHVASRMT
EPVQGRFTEVELKGLPPSGTGSFVRAYFGDV
>A9GAC3/718-885
KMALVLGEFGLGKSTALAAWAERRWASAEGPRPLLVNLAGASPHASPEQLLLQAAGAPDL
PANRAALRLLIRHRLVVPCFDGFDEMATRLEASEFAGRRASLLEVAVGGGKVVVSSRDDH
FPADAHLPTTGEFGPSQTPGTSVGIRRFTILPFTEAQVHELVRQIRGE
>A9G4R3/336-482
SRAVIVAAAGFGKSVLLHALAYHRARSAWLPAHVPLVELAESKLPVLDFLLEHVNRRLAV
DVPWLHYCENGSALLLFDGLDELDQAARTRVLDRIKEFSSRFSEVPWLLTVRDAGALNAP
LNPPILTLEVLDQEGISAMCEAYLKAA
>A9GJ03/459-611
RAIALTGEPGAGKSHAAHRVALYLAQRDAVDFAFYFDFGAEPYSVDDILEMIAPVLGLAP
DRKAETRVALAKLRCCFALDDVDRAAHRANGAPGAAAGPLLEFVRELLAAGHVVVTIGAT
KAAALAPDFVELPVAPLSADEQQALAADELRAR
>A9GSI5/555-721
RFVLVLGDFGTGKTFLLHELARRLGNAGGPLTPVLIEMRALEKARDLNALIAQHLALAGL
GKIDLPAFRHMLAEGRIALLFDGFDELAFRVTYDRAVEHFDTLIQAAQGKAAKVVVTSRS
QHFISEQQVRSALAERAALLPGYRLVRLQPFTGAQIRRFLVNRLDSE
>A9ERF1/42-180
WLLLLGGPGVGKSAILTSLLRMLPAPRPPCHFIRRGMEGFDRPEVVVQSLCAQLERLYPE
HGSADLPSEMRLGELLKRLSTKELARRGQRLLLVIDGLDEAAGDDPLPRILPRVLPPGVV
ILCASRPIYPALSWLERDG

>A9FTS8/574-737
RFVLVLGDFGAGKTFLLHELARRMVREGHPLVPVLIEMSRLEKQRSLNVLVAQHFALADE
PIDPRAFGYMLAEGRIALLFDGFDELALRLTYDRALEHFETVMQAAEGIAKVVVTSRTQH
FLDDRQIEQELARRAEQVQGYRLVQLEGFGEPQIRRFLGNLIPE
>D0LS20/337-486
SYVVLRGQAGDGKSTLAVELARYLVHIERFARAVFLRPSTPLDIDAFEDALGSQLVADYA
ARAQQDRASARRTLQHELVDRTIVIVVDGVDHVPATREAAPMARETASSRFFALLDHWAT
QCATSLVFTSRTPLPTPFADRDVFVGRMSR
>D0LSJ4/225-395
KIVVVSADFGHGKSLTARRLARDTARAWLESDTPSPQNRYPVFIKCARDIRDASYKHDEV
ARRALWEAATEALGEESSSEEPQFQPPDNQHAALFILDGLDEVAFSPNQLEDLFRSLREK
LGKQQRAIIFTRPSTFDDRHGRPAENIPLISLLPFDELQIEEWLTRWNNNP
>D0LTC7/440-615
GAIILGMKGVGKSCLAARAVQRLAQGLADPSELGQVVLHGALDEFTVLEQFEMQAIRWGD
RDAEDILSDAREPLPRRLRRLLAGRWRTRRLVIVLDNFEHNLTPRAEGDALLHPEVAALL
DALVPACRTGHAKLLVTTTASFELPASARRSLPVIRLGPFEPSSLRKLWNRSRHDE
>D0LH82/472-643
RPLIIRGPGGVGKTTWLRWTLRRLFAEGAHPEVLPLFIEVRALMRDWQGKKEDERNLDSY
LAGRLAVYLGDDVRTTLRDAVPAWLDAESEGPRPVLFIDGWDEAGPLGCDLREKLLVLLT
RYPRVLAVVTSRPYGQDPPTSADGFELLDVQPLSDADIDAMSARFYREVFHE
>D0LHT8/801-999
RHLFILGEPGAGKTTALQKLLWTTLSADGRPAFDGSRIGLDKNTVPVLLRLRDLGPSQRR
QPLSAFIEAQLKLLGQPSENESSSNAGGAEEPLELPPGFGTWLWKRGHMLLLLDGLDEIA
DSVQRNEVCRYIENHLKAARAQGIDGIRVVVSSRYAGLYNTLSGDVPAIGFGQDFARLEL
CPLNDTQIEALIGNWFAAA
>D0LV07/575-739
LVFLAPPGSGKSTILRRIEVDAATAALRGGAPRITFFVQLNHFRPAEPDAPMPSPAAWLA
ERWHSRYPDLPPLPELLAEGRVTLLLDGLNEIPTPDDGVFRTCVRMWKDFAAEVTQRRGS
GEGNRLLFSCRSLDYSAPLSSPSLRVPQVRIEPMSNRQIRQYLKL
>D0LXH1/124-287
RPLVIRGPAGTGKTTWMQWTFRRLLERDDAVPCLVELRDLAKVWGEAKPARRTLDTYLRD
WVAEWVGGAWQESIDDMLRDLLEFDEGPRPVLLIDGWDEIGDLGSELRQKLAGFLKVFPR
VLAVVTSRPYGQGKPTDSDGFETVDIQPLSNGDIDTMAERFFRL
>D6TWY5/196-350
ELLILGEPGAGKTTLLLELTNHLLQQARQDALSPLPIVFNLSSWGKKRTSLHVWLLDELH
TKYQVPHQVARRWIEDEQLALLLDGLDEMQATHRLACIEAINQYRQVHGLVPVVVCSRTA
EYLNQPVRLVLNKAISIQPLSAQQVNKYVERGGEA
>D6TTL2/55-219
RLMLISAAAGFGKTTLLASWVRTFPPGHPPAAWVSLDAGDNAPVQFWTYVLTALEQCRPG
LSPLSVASLSEIPQPSWQAVLATLINNLARQSEPLVLVLDNYEEITEPAIHALLSYLIEH
LPPTLCVVLATRTDPPFSLARLRAQAQIQELRTEQLRATSEEMTA
>D6TTJ5/173-327
QALLILGEQGGGKSTLLLELAYHLVEQAKQDETQPLPFLLPLSSWPGDFKHLQHWMAQQI
TLLYKVPESLSKQWAKTASIVPLLDGLDEVDASDRATCIKAINTYRKERMSAIVVCSSGD
EYETASTHEQLDLHAAVVIQPLTREQVDTYLEEPG
>D6TBZ8/57-220
HVILVSAAAGFGKTTLLANWVRSFPPGHLPVAWVSLDAGDNVPVQFWTYVLTALEQCHPG
LSRLPFAALHETPRPSWKAMLAALINGLSRHNEHLVLVLDNYEEITDPSIQALLSSLFEH
LPPTLCVVLATRTDPPFSLARLRARGQILELRTEQLRATREEMI

>D6TC08/180-335
QEFLVLGEPGAGKSTLLLELAHHLIKQAEREHDHPLPVYLPLSTWATHRPPLQNWMIEQF
ASVYDISRNVSQRWIQAGLVLPLLDGLDEMEEAARPTCIAAINAYHRDHMSPLIVCSRTS
EYDHAATQEKLMLHTAVVVQPLSRVQVDTYLTTLGK
>D6U1Q9/110-263
GQLLILGEPGTGKTILLLELARNLLDKAVQDDSHPIPVIFDLRSWGKKRRSLAEWLVEEL
HDKYRVQEKVAQVWIRNNSLVVLLDGFDQLAVRVRQECIQAINAYLRQQGFVSIVICSRL
TDYLHQKVQLDIQRIITIQPLALQQVLTHLSQRR
>D6TGT5/288-454
RNCVIVGDPGAGKTTLLKRLTLSAIDGALEAMPVLPLYIKLHKVARKQQFDLFAASVDVL
VAYGFSRAQVTLLLEERMKAGDILLLLDALDETVIGETSQAAEESYRQVHEAILNMKRRF
NRIPVVVTARKAGYFQRGQLPGFTELEVLDFLPRDIEEFIGKWFRHG
>D6TGW6/205-359
ELLILGEPGSGKTTLLLELARHEIARARQYERYPLPIYFNLASWAERQLPLEQWLVDEIN
IKYRISPKLAQTWIEAEHIAVLLDGLDDVAPEYRTACIQEINTFRQKYGLAPVVVCCRSA
DYLAQPARLALNKAIVVNTLTAEQIDEFVAQAGQD
>D6TKV9/204-358
QELLILGEPGAGKSTLLLELAHALVAQADQDATSPLPVLLPLSTWVTKQPPLQDWLIEQF
ALIYDVHRTLSRQWIEAKLVLPLLDGLDEMDEAARPACVVAINTYHREHLSPLVVCSRTS
EYEQATLHERLALDTAVVVQPLLSAEVNAYLATLG
>D6TRQ9/405-572
QHFVVVGSPGSGKTTLLKHMVLTLASRRRLEQGSAIPTKLPVLLFLRDHAQTIKRRGERY
TLEEAAADHHKKYWRQSMPAGWLKQRLESGNCLIFLDGIDEVADLQARKQVVNWVQQQML
VYGQNRFIITSRPFGYRSNPLTGVTVLEMSVFTRAQVERFIYNWYQAN
>D6TTU6/282-451
SYLLLRGDAGSGKTTLMRWIAVQAASQCFPPELASWNGLVPFFIRLRRHVHLEGGGVPRW
PAPEDFPGLVAPAIAGAMPQGWVHRQLQDGRAIVLIDGIDEVPGAMRESVYTWLADLLES
YPASRFIATSRPYAASKNDLPAQERLKEAQVLPRSPAAIEDFITHWHDAI
>A9B8T7/147-335
PYLVLCGAPGSGKSTFAKHLVWALAQRGLDQINHHTGLLGWADKQRVLPVFMPLRTLAGA
LVGKDLGLNNTPHIGLLLDAVCAHLQTTYGLEQPRELLSAGLDRSRTVLLVFDGLDEVPL
EATDHSLDRRSLLTYVRLFANAYAARILITCRSRAWTEEYGQITQWPMVELAPLSGGQMT
QFIRTWFPL
>A9B8T8/149-336
HLVLCGAPGCGKSTFAHHLVWVLAQRGLDQINHHTGLLGWNDTQRLLPIVMPLRRLAGAL
VGTDVGLTDAMPNVGLLRDAVCAHMQTKYGIEKPHTLLDAGLARSLKVLLVFDGLDEVPL
EASSTSLDQRTVLRFIRRCAGLNVRILITCRSRAWTDEYRQITQWPMVKLAPLTGGQMTQ
FIHTWFPQ
>A9B8U0/203-367
GYVLIKSEAGEGKSSIIAKLIQDAGFAQTPHHFIALTTGRDYQLSLLRAVVAQLILKHRL
PVSYFPEESYSTMKGEFARILDELSKHGIQETIYLDGLDQLPPEGDRLIDLSFLPSQPPP
GIVIVLGSRPDEAFKPLHHLNKAAYHLPPMSEIDAFTVWRSVQSG
>A9B8X7/148-337
QYLILCGAPGSGKSTFAKHLVWALAQRGLDQINHQTHLRGWTDKRQLLPIFMPLRQLAGA
LAGNDLGLHAEPKIGLLLDALCDYLQTHYGLDEPRTLLTAGLNQRHKVLFVFDGLDEVPV
EANEHSLDRASLLRFLRIFADHQPNARMLITCRSRAWTSEYRMITQWPMHELAHLTGGQI
THFVHYWFPQ
>A9B1E6/137-273
RCLCINGLAGVGKTVIASQIAQSWGERCFWLSFTPTLSLSSEILIRQLALFLLSHGDDQV

EPLLHLPRDGEAGLSFERQLGLLINGLQHIPALLCFDNAQLLIDQPQLRLMLEQLAQKTT
SQILLLSREQFNLQGFS
>A9B8T6/149-337
PYLVLCGPPGSGKSTFAKHLVWALAQRGRDQINHHTGLLGWNDHQRVLPVFMSLRTLAGA
LIGKDLGLTDTPNIGLLLDAVCAHLQTKYGLEQPRELLKAGLKGSLTVLFVFDGLDEVPL
EATAASLDRRSLLTFVRLFASAYAARILITCRSRAWTEDYRQITQWPMVELAPLSGGQMT
QFINTWFPL
>A9B1W4/289-433
QLLTIMGAGGSGKTQLALTFGWKVVNEYLGASSNGVFYISLVSADQQPRLLDAEPVLLAI
VQTLNLPPPRTNDLVEHLILQLQQHELIIIIDNGELLATSARLALSSLIQHIPQLRLIIG
SRERMRLQNEYVLELAGLAYPQIND
>B4VKS8/191-343
DKLMVLGKPGAGKTTFLKYLAIQCAKSKVLTDKVPIFITLKQFAETQSQPSLTTYINQIF
DNCNVTEVQVAAFLKHGSGLILLDGLDEVREEDADRVLTQIQAFTEKYDANTFVITCRIA
AREYTFEKFTDVEVDDFDDKQIRTFATKWFQAK
>B4VY06/82-247
QRLMVLADPGMGKSALLRREAALTAASERQKLVDAQITVEDVVFPLFIRLVDLHETADDI
AVAIASLVQRDYPTTWADIQPWLQAKLRQGKCLLLFNALDEVPKRDRNALATKLNQFLET
HPCPIICTSRIVGYGGAFINGAKEVEIIPLSQPQIERFIHAWFKHG
>B4VMX1/288-451
PYLVILGDPGSGKSTLLQYLALIYAQSPLQNAISLAIPILIELRTYMRNRDLGQCQNFLE
FLHQSSGAICHLNQHQLQEQLKAGNVLMLFDGLDEVFDPGKREDVITDIHRFTNEYPDVQ
VIVTSRVIGYKPQRLRDGEFHHFLLQELDEKQIQDFIYRWHELT
>B4VXV2/208-359
SNLMVVGKPGSGKTTYLQRVVTECNQGNLQAYRIPVLIKLREFVDDGREFAYSLKRYLEQ
YWQLSNTETQLVLKQGRALVLLDGLDEVTGEDGKNITKEIKRFARTYPQVQVIVTRRTQS
QESRFERFDYVEVADFNEAQVRAFAEHWFKAI
>B4VVP5/281-437
PYLVILGDPGSGKSTLAQYVALDWAEKPTKTIPLLIELRNYARDRTLPKTFLEFIHQGAA
AICHLNQHRLDEVLEAGDAFVLFDGLDEIFDPVARDTLITEIIRFTNQYPQVRVMVTSRI
IGYKAQRLRDAQFRHFTLQDFEAEQIQAFVQKWHDLA
>B4VV30/202-367
RSLAIIAAPGSGKTTLLEHLTLTYAKNRHRHYHKQAPKLMPILVYLREMADAIASSDFTL
AELIEQQESIRKLSPPPQWFAGKLRHRDCLVMFDGLDEVADESQRQMISRWVNQQIQDYP
NTRFILTSRPFGYQSAPISSVKAILEVQPFNLQQMQQFIQNWYVQR
>B4VU47/82-233
NHVLLVGQPGSGKSTALIQFLVEQAKQALSHPQNPIPVLVQLRQFKPSETHHSGVLYLIQ
DFLEIHELLLEISDIKNLLRNRRLFLLLDGLNELPSNSARRDLKAFRQKYSHLPMIFTTR
NLGEGWDLGIRDHLEIEPLNPLQIKQFIHYSM
>B4W0D9/292-455
PYLVILGDPGSGKSTLLQYLALDWANSAPNDANLQPIPLLIELRTYMRNRDVGQCKNFLE
FFHDSSGIVCHLNQYQLVEQLKAGNALVMFDGLDEVFDPGKREDVITDIHRFTNNYPDVR
VIVTSRVIGYKPQRLRDAQFYHFMLQDLEADQIQDFITRWHDLT
>B4VVM7/260-417
IRLISGSPGSGKSSFAKMFAANLAETDKIPVLLIPLHHFNPADDLIDAVGKFVHTDGILH
NPLAADCRESRLLIIFDGLDELAMQGKIAEKIAQDFVREVQRTVDRLNQRELCLQVLIAG
RELAMQANETDFRKEGQILHVLPYFIPENKRKNYVDEN
>B4VVF0/342-496
KLLILGQPGAGKTITLLELARELLTRAETDVDEPIPVFLNLSSWKYNNQKIADWLVDEIS

DRYKFLSTKTVKLWLKASKLLPLLDSLDELESKRQEKCIQAINEFLASEYQLLPLVVCSR
CKEYQLHSTQLNLHGAIVLEPLNKLQVQGYLASIK
>B4VI70/287-450
RYLVILGDPGSGKSTLLQYLALDWANSTPNDANFQPIPLLIELRTYMRNRDVGQCKNFLE
FFHDSSGIVCHLNQHQLVEQLKAGNALVMFDGLDEVFDPGKREDVITDIHRFTNNYPDVR
VIVTSRVIGYKPQRLRDAEFHHFMLQDLEESQVKEFITRWHDLT
>B4VZK4/306-468
QILFIQAGPGRGKSVFCRMFANWVREHLHPIWTPILIRLRDIDAFENNIENTLRAAVKAD
FVKSNDGWLTDRNTRFLFVLDGFDELRMEGRTTGGIEKFLKQVGNFQTSCQQHSQLGHRF
LVTGRELALHGIERFLPGNLERVEIALMNDQLQQQWLEKWSNL
>B4VX86/308-472
QVMFIEGGPGRGKSVFCRMFADWVRQHLYPIWIPILIRLRDITTFDKDLEKTLQTAIGWD
FATSDSGWLTDGNTRFLFLLDGFDELLLQGRSDRGLKQFITQVAKFQQRCHENSEKGHRV
LITGRSLSLQSPSIELSMPNNLERVTIIPMDDKSQKKWLDKWQIQ
>B4VPA5/333-504
RIAVIEADGGCGKTSFCQMLAARVAQELYPQWMPVIIRLRDVTLGQTLEQTLETAFPVGR
FTDTDGWFSRSSPPCLLILDGLNELPRSPQTERHLWTFMDQVMRFHTQKNGSNSPRHKII
LTSRPVTLDGVLARRYRHSSLPPLQSRLQRIVIEPMAKEEFRKWFLQWAKLQ
>B4VVB9/86-236
TNLMVVGKPGSGKTTYLQRVVTECNAGNLQAHRIPVLIKLREFVEDGREVAYSLERYLER
CWRLSDAETQLVLDRGRALVLLDGLDEVTGEDGKNITKQIKRFARAYPQVQVIVTCRTQS
QESRFERFDYVEVADFNEPQVRAFAEHWFKT
>B4VX71/181-335
KLMVLGKPGAGKTTFLQHLAIQCNTGKFKPEQVPIFIRLKNFAEDACCENNGNLFNYITQ
EFERCQVTAPELETVLNQGRALLLLDGLDEVPDTWSDAVIRQIRQLADHYYKNQIIITCR
IAAKEYRFPGFTEVELADFKRPQIEQFVKNWFIAV
>B4VSZ4/181-336
SKLTLLGKPGAGKTTFLQYLALQCNAGHFKPDCIPVFISLRQFTKQARTTGNFSFLNYLS
TKWKTFGISPEQIELLLQHGKVLLLLLDGLDEVSKADTPELLQQIQLFADEYYQNQIIITS
RIAAQQYHFRGFTYVELADFDTHQIETFAKKWFTAT
>B4VKV4/1-119
MPIYIELRDLVQDANTLSLLDYICQEFSDRGVSGETVKTLLQEGKLLILLDGWDEISPLR
RFSLFNKLRWFCNRYAKNRYVITCRTGVELYRFGGFTEVELADWTLTQISHFARKYFVA
>B4W1B6/251-417
YRVVLLGNPGGGKSTFTQKFAHVLATCYSERPFAGRQVTPILVILRDYGAKKKQINCSIV
QYIETKANSDYQTPPPPRAFEYLLLTGRVVVIFDGLDELLDTSYRREISSDIELFCNLYP
SVPVLVTSREVGYDQAPLDPEKFEVFRLAPFDENQVKEYVTTWFKVN
>B4VNR5/129-284
PYLMVLGSPGSGKSTFLRRMGLEALKGKKGKFKHACIPVLIELKTFNQGEIDIEHKIAEE
FRICGFPSYQAFTAKALEQGKLLILLDGLDEVPTERMNQVIQHIQNFVDLHDKNRFIASC
RVAAYRHNFRRFTDVAMAEFDDDQIENFISNWFGRE
>B4VHK4/567-720
RSLLILGEPGSGKTTTLLELTRDLLDQADVDSNHPIPVVFNLSSWTGGKQTIADWLVNEL
HTKYQVSKDIGQTWIDSGELLLLLDGLDEVSITLREACVTAINQFTSEHGTTELVVCSRI
RDYQALQQRLNLQAAICLQPLTLVQIDRYLNSAG
>B4VWK9/412-554
AESSTVDLNKVGNGKIDNVIFPLFLRLSDLDETPQEIINAIPQLIQRDYPKNYPAIDTLL
KDKLENGKCYLLLDALDEVPKQNRTRLAEKLNRFARNYNCPMICTSRIVGYGGAFLSEAK
EVEIVPFSQKQTEAYIQTWFKNA

>Q8YRI1/373-555
EHISILGEFGTGKTWFVFHYAWTALQRYKDAQRRGVERPRLPLVITLRDFAKALNVENVL
AGFFFTQHNIRLNSEVFDQLNRMGKLLLIFDGFDEMAAKVDRQQMINNFWELAKVVVPGS
KVILTCRTEHFPEAKEGRALLNAELQASTNKLTGETPQFEVLELEKFNDEQIRQVLLYQA
EEA
>Q8YJU3/254-429
RSIAIIAPPGYGKTTLLEYLVLNYANNTYFQHNAKAPELIPIILYLREIQNSIIEEQPDL
PTLIEQQELVKKNQPLKSWFEDNLRLNINLTTRNYKTQKCLVMFDGLDEVELHQRQLISR
WVNKQIQEYPHAIFLLTSRPFGYRSAPVENIKAILEIHPFNSKQVQFFIQNWYLQN
>Q8YY92/293-447
TIAVIESEPGYGKSSFCQIWAAEVALKLYPHWMPILIRLQDIKYGKSLLETLNSGFTLNA
HVNLSTWLEQTNNRCVLLLDGLDELPASHQGNRAKKIFIQQLLQLQSQEQHKIVLTSRSQ
TVEEITSEIPLQWRRIKIQPLEINQLKQWFQQWAF
>Q8YRI2/246-411
QKFVLLGAPGSGKTTLLSYFVVMLAQKQIEQLNITASDYLPIIIPIRDFARQANISVIEY
VKQFVEKNLCVKTLPVGFFEYWLEDGHTFIFFDGLDEIAQENKRYDVVRKIENFLGQFPK
NCAVITSRPAGYKRDFFNTQEFAHYELLSFDDEKIEKFINCWYDSR
>Q8YX84/301-463
QVMFIQGGPGRGKSVFCRMFAYAVWRQLHPIWTPILIRLRDIDTFESRLENTIKAELKLG
FIQGDANWLTNANTRFLFILDGFDELHIETRNNLNLGDFIKQVAGFQKECKDYSEMGHRV
IITGRSMALQGIANLPRNLERVEIVEMDGQLQQQWLNKWEALQ
>Q8YVT6/305-468
KYVVILGDPGSGKSTLLQYLALNWSESPLDNVISQPIPLLIELRTYMRRRDGNECHNFLE
FLDKCSGAIEHLNQHQLHQQLQAGNALVMFDGLDEVFDPGKREDVITDIHRFTNEYPNVQ
VIVTSRVIGYKPQRLRDAEFRHFMLQDLNSAQIQDFIHRWHELT
>Q8YL12/132-283
KLLILGAPGSGKTTTQLELAQELVKRAEEQSDYPVPVLFNLSSWKDDRQSITDWLVAELK
SKYGVSKKLGQEWVDNHQLLPLLDGLDELEPQRQELCVHAINRFLGGEDRPLYLAICSRS
EEYSNYATQLQLNGAIYLQPLTNNQIYDYLTS
>Q8YUT9/184-338
SKLFVFGNPGAGKTTFLQSIATLCNQGKFQPQRVPIFISLKDFAENIDYDDKNPLLSYLI
EELSICKIAQQEVEELLHKGKVLILLDGLDEVADKYTDKINKTISTFLDIFYKNVVIISC
RIAFRHQKFKGFAEVQISKFTKSQISDFANKWFAA
>Q8YYH6/288-451
QYIVILGDPGSGKSTLLQFLALNWAETPLGNAIYQPLPLLIELRTYMRRRENNECSNFID
FFHKSSGIVHHLNQHKLHEQLKTGNALVMFDGLDEVFEQGKREDIITDIHRFTNQYPDVR
VIVTSRVIGYKPQRLRDAEFRHFMLQDLEPEQIQDFIHRWHELT
>Q8YL11/132-288
ELLILGAPGAGKTTTQLELAQALIKQAEENPTYPIPVLINLSSWKDKNQPISEWLVVELK
SKYGITKQLSQHWLINRQLLLLLDGLDEVKPIFQEACVKSINQLVRGEYRSSSIVICSRS
QEYQNYKTNLRINGAICLKPLTKKQINKYLIEINNAD
>Q8YV52/243-386
QRIAIVGEPGAGKTTFLQRIAAWVLDNTADLPVWISLADLQGKTLEQYLIQDWLPSAMRK
LRVSPELEDAFCEQFNQGRVWLLLDAVDEMAIESTSALAKIASFLKSWVGDATIILTCRL
NVWDGGKNALENFDTYCNLHFTYG
>Q8YXI1/206-360
SKMILLGKLGSGKTTFLQSVALSCTQGIFQPNYLPIFVNLKNFAEDAKDSRQLSLFKYIL
DHVMNFGITEGELRTVLSHGRALILLLDGLNEIIGQNHEKNINKIHGFIQKFYKNQIVITC
RTGTNYSNFHGFTEVEITDFDKIKITEFANKWFLR

>Q8YJU8/146-302
KLLILGNPGAGKTTTMLDLAKALIARAEQDADYPIPVLFNLSAWKYDKQSMRDWLVLELK
SKYVVRQDIGAKWLDDVKLLPMLDGLDELESARQEACVNNINKFVQNEWRSQYLVVCSRS
EEYTNYKINLQLNGAIFLQPLTDKQIQAYLTRLNQLE
>Q8YQN1/178-333
SKLMLLGKPGSGKTTFLQYLAMECNYGKLRPNQVPIFIRLKEFAEDTQRESELNLLQYLV
QEFRCNGVEEESTLAVLTGGKALILLDALDEVPLSHVDKVIREIRKFIQTFYKNQFVITC
RVSAQKYRFQGFTEVEIADFQEQQRDVFVKQWFMAV
>Q8YSU6/505-637
ATLLIWGEGGAGKTSLACTLAQWAMNDNESQRLSKHQMLPVLIEQELDHPLKETIGGQLQ
ALISETKTIDDELLTNLLRQRRILVIVDHFSEMSEATRNKIHPAEKDFPVNALIVTSRVE
EKLNEVPKTTIQP
>Q8YMK4/180-335
SKLRVLGKPGVGKTTFLQHLAIQSNQNTFAAHQVPIFITLRNFAEESKVTHEFSLLNYIR
QEFITSGISDPTVIETLLNAGRVLLLLDGMDEVLNQQSNAVLSEIRRFSDKYHKNQFVAT
CRTASQKLVLRGFTDVEIAPFTLEQIIAFAQKWFVA
>C7QVL9/113-279
PRIALLGPAGTGKTLTLQKIAHWILSQTEDLPIWLSPNQWKQAGVEDYIYHQWLAQAATN
YHSGKYPLKVWQESFEALLNRGQVWLLLDGIDHITLDHTESGLTSPLSVLADQLQGWTQQ
SCIVLTCQTQTWEDDLDGLSNFEIYQTQEFADFLGVRRFIQRWFEPK
>C7QW90/69-223
NKLMIWGKPGAGKTTFLKYLAIACIKNQFASEKVPIFVTLKQFAETEKQPTLLTYIFDQF
REVNVKEDEIKNILAEGRCLILLDGLDEVRPEHSTRIINQIQQFADRNNYRKNQLIITCR
IAAKDYTFQGFKEVEVADFDREQIEEFVNKWFQKK
>C7QRA8/179-333
NKLMILGQPGAGKTTFLKHLALQCSQGKYRRDVIPCFIELRSWLMETEHETANLWEYFHH
QAKKCGLSSEQAIMLLQEGKGLFLLDGLDEVEQEEREILAKTISQFTQVYHKNQFIITSR
PAAQLFHFQGFTYVEMAAFNRHQIEAFARQWFVAT
>C7QPN4/364-546
EHISILGEFGTGKTWFVFHYAWQKLQEYQKAKERGTQRPRLPLVIPLRDYAKAVTVESLL
SEFCFRKHEIGLPGYTAFEQLNRMGKLLIIFDGFDEMADRVDRQKMINNFWELAKVIVPG
AKAILTCRNEHFPEAKEGRALLNAELKASVANLTGEPPQFEILELEKFNNHQIRTVLEKR
TDG
>C7QY27/309-456
RTLLILGEPGSGKTTTLLQLTRHLINRANEGIDDRIPVVLNLSNWSVEKRNFTDWLVEEL
NSKYQVPKKVGKTWVQQQLLLLLDGLDEVTVKHREKCLQTINQFHQNYSPEIVVCSRFK
EYKELSSRLNFQKAIYLKPLQLTQVLDY
>C7QVH2/276-485
PCIVLEGAPGQGKSTITQYICQVHRMRLLNQEDLSKTIPKEYRQTETVQKLPQNHKNSAV
KLPFRIDLRDFAKWLSKKNPFNIDNDEVPENWEKSLESFLAALIKHRSGGCEFSVTDLLA
IAKISPVLLVFDGLDEVADISRRREVVDEISRGIRRLKVNTESLQVIVTSRPSAFANSPG
LQDDDFTYFQLESLTNPLIFDYTDKWLKAR
>C7QTA3/284-447
QFMVILGDPGAGKSTLLKYLAIRWTELPRKALTTQPIPLLIELRDYLQNYQKNECQNFLE
FIDKSSGWVGHLNQHSLDETLKRGDGLVMLDGLDEVFDLQQRRMIINQIHDLTQDYPKIK
VIVTSRIIGYEPQWLRDANFNHFMIQDFDDNQIEEFSEKWHQLT
>C7QMT8/163-341
RRIVIIADPGFGKTTLMRHLAYIYTTEPPQNTPQFIPILLRFRDIYHDIRPLNLDSENKE
TSLIDLISLIINHWKKQPEFDELQPSRNWLQDNLKEGNCLVILDGLDEVPKGQLETVRRW

TDGIMKKHKNTEFILTSRPHGFELQPNSPTSSIKIDLKLRIREFTNDQKAEFINKWYRT
>B4SAE4/278-445
RMLLVLGDPGAGKTTLLKYYALCALEQSSKLGFAVTPKLFYLPLRELVRDKTGNNFDSLP
ANLESWVARHHHTIQTPLFDKWLRSGVSLVLFDGLDEISNTRERKEVCRWIDRAWSGFGT
ARFVVTSRATGYRKEEGIELEVDHERADVQDFTPVQQERFLRNWFTAA
>B4SAB6/280-447
PLLLVIGDPGSGKTTLLKYYALSCLDNGRCTQFGFSEDVNVFYFPLRELKKGDSGYASLP
AILSGWCEKNYLTLPDTLFSGWLEEPSSLVLLDGLDEISDVDERIAVCSWVDRMVERFTS
ARFVVTSRSTGYRKGDGIELEASHLRADIMDFSKEQQAEFLQRWFKAA
>B4SAC5/296-464
PLLLVIGDPGSGKTTLLKYYALSCLDNGRHKEFGFNEPVHVFFLPLRELKQDEHGAYASL
PANLAVWSSKQFLKIEESLFSGWLERPSTLMLLDGLDEISDVHDRIAVCKWIDRTVGRFT
NAKFVVTSRSTGYRKGDGIEIESSHLRADIMDFSKEQQREFLHRWFRAA
>B4SEC3/414-579
RMLLVIGDPGSGKTTLLKYYALRALEDSARLGFSDPVNVFYLPLRELSSHAGHYDTLPAN
LASWSVMQHHAIKKECFVDWLQSGTSLVLLDGLDEISNTQERIEVCKWIDAALNTFTVAC
FVVTSRATGYRKDEGVELAADYERADVQDFTSEQQERFLRNWFKAA
>B4SDM3/279-446
LLLLVIGDPGSGKTTLLKYYALSCFDNKRYQEFGFREPVNVFFLPLRELKKSDTGYMSLS
ENLAAWSEKHFLKIEETLFSGWLDKPSTLVLLDGLDEISDVHDRIAVCGWIDRTVGGFTN
ARFVVTSRSTGYRKGDGIELEAGHLRADIMDFTKEQQAQFLQQWFKAA
>B4SET9/276-443
RMLLVIGDPGAGKTTLLKYYALCALEDFAKLGFSAPLNVFYLPLRDLVRDKEGRCTENLP
ATLAAWSEKHHQSIDAKVFNDWLNHGSSLVLLDGLDEISNIEERKEVCRWIDNAFSGFSK
AWFVVTSRATGYRKDEGIELASDYERADVQDFTPEQQERFLRNWFTAA
>B4SE39/418-588
TTLLVIGDPGSGKTTLMKFYALTCLGKNLPLSSTDLGFQEPTFVFYLPLRDLERGKSGYP
PLSSSLAGWAKRHSLPISSRVFREWLESRTSLVLLDGLDEVSEPERRKEICRWIKDMVGL
FSKARFVVTSRPTGYRQAEGIALDFQHQRVAVKDFSPSQQVTFLKKWYGAA
>B4SAC3/300-467
SLLLIIGEPGSGKTTLLKHYALCCLDEQQRERYGFKRAVTVFFLPLRELQKRGNRYRSLE
ENLAASSHKQLLTIKKEHFTQWLEQPSALVLLDGLDEISNVNDRIAACKWITRTVRRFTK
AKFVVTSRSTGYRKGDGIEIEAGHRRADILDFSLEQKREFLQRWFKAA
>B4SEU2/483-667
RLWLVLGDYGTGKSTLVERFAYELARNCENNSESPIPVEINLRQFPNAISLESLIREHLE
AELRTVLNPEIVLHLLEAGRVVLLLDSFDEMGVAQAGRSVEEQFRQLVRPTASSGRNPRA
NRVLITSRSHFFRDNSSARQAVQGGDKLFEADSALGKAARAFDATLDMLPVFTKEQIAEY
LQKRL
>B4SG18/440-605
RLLLIIGDPGSGKTTLLKHYALTSLDDRKRLGFTEPLLVVFLPLRDLIVENGDYAPISHN
LIAWSALHNLDIEEKHLSRWLQQRPTLLLFDGLDEISDPQQRIRACRWIDRIVASFKKAQ
VVVTSRSTGYRKAEGIELASRHTRADIMDFTSEQQQEFLEKWFRAA
>B4SDL9/276-443
HLLLVIGDPGSGKTTLLKYYALSCLDNGRCHEFGFSEPVNVFYLPLRDINKSDTGYPSLP
AALSAWSEKYFLKIKENLFAGWLGEDSTLVLLDGLDEISNVDDRKAACDWIDRTVSRFTN
ARFVVTSRSTGYRKGDGIELEASHLRADIMDFTKEKQAEFLHLWFSAA
>B4SEB9/424-597
RLLLVIGDPGQGKTTLLQHYALSCIDKERCKDFGFPEPVMVFYLQLRDLKKGDTGYSALP
VNILAWAHTVPSSEKERPENLETLIFESLCQKKSLVLLDGLDEISELEERKEVCEWIKNT

ITDFPKACFVVTSRPTGYRPVDDIEIQIPLKRADILDFKPVQQKTFLQNWFTEV
>C5LHC7/935-1117
RQFLVSGAPGSGKSCLLRRLVIYCLNSQNDLLPLVIPVRELAKNYVLLTQHTDERLSTSS
SSSAAAAGDNSCCLLDTDADGPDPETLLEAWMRSRLGDESSRLYLMRMMLASGRLLILMD
GLEEAGSAKCTIEKLLSLLAVNKHRVVCTVREGYLSDHSQESLTASGFEGSSLSLLDAEQ
CRF
>C5LBC3/946-1128
RQFLVSGAPGSGKSCLLRRLVIYCLNSQNDLLPLVIPVRELAKNYVLLTQHTDERLSTSS
SSSAAAAGDNSCCLFDADADGPDPETLLEAWMRSRLGDESSRLYLMRMMLASGRLLILMD
GLEEAGSAKCTIEKLLSLLAVNKHRVVCTVREGYLSDHSQESLTASGFEGSSLSLLDAEQ
CRF
>Q4N1I1/26-93
KQIWLVGPEGRGKTTLIHGILDHLKNNKAESERVFLDLNVRFPKFNQHISSFFNHLKNSN
VLKSNHKW
>Q22D00/970-1136
IISILAEGGSGKSMLLKKIEVEILNDNSKYKNDKRTDFIPFIIKCNSLDKEKPSIEDYLE
SLNIRRKDIDNLKKSERNKLIMLDGYDEYTGDYFKVYQKLNLNEWVNTLVIVTSRLEKIT
VSDAKGYFNYYDNQGNIGHRDSYAIFKLEKITNQDIEDYLEKYKNQQ
>Q229E8/912-1077
IISILAEGGSGKSMLLKKIEVELLNDNSKYKSDNRTDFIPFLIKCNSLDKEKPSIEDYLE
SLNIKRKDIDNLKKSERNKLIMLDGYDEYTGDYFKVYQKLNLNEWVNTLVIVTSRLEKIT
ISDAKVYFNYYDNQGNKGHSDSYGIFKLEKITNQDIEDYLEKYKNQ
>Q23FE3/446-606
RILGILAEGGAGKSMLLRQIEARLKEEDEDGLSILPIFIKCNSLDAQNPSIESYLESFDF
PIEQIEKLKQSDWNKLILLDGFDEYSGNYFNVYSQLKISEWQNTLVIVSSRQEKLSVDDA
SLCFSIKDDNFNVIESSYVLVKLLEFQKKDIEIYYQQFFRK
>Q22D01/1082-1247
IISILAEGGSGKSMLLKKLEVELLNRDSEYTRDTRSDFIPFIIKCNSLDRKQPSLEDYLE
SVKIKRKDIDMLKNSERNKLIMLDGYDEYTGEYFKVYEKLKLNEWVNTLVIVTSRLEKIT
ISDAKKYFNYYDNQGKQAKNNNSFGIFKLEKITDKDIEDYLEKYKK
>Q233I1/791-1004
NVVAIKGGPGMGKTHLSRELMRRLMKKIQETGSRNIAIPILVEMIEVENTSQHLDLESFL
NSSIERYAFRSSKELEYLKNSSIPKVIILDGLSEWSWGSPNTSFREWIRLREWNNTKLII
TYRKPFVRAEEFPYFFGQKCQQPKDIEEIRAYKKSINNPGPFIENHKNTIFTTNQNIPKQ
NANFQEDDRETCFEVFQILSLSQENVEDYVRQKE
>Q23UK4/1052-1218
IISILAEGGSGKSMLLKKIEVEILSDNSKYKSDNRTDFIPFIIKCNSLDNEKPSIEDYLE
SLNIRRKDIDNLKKSERNKLIMLDGYDEYTGDYFKVYQKLNLNEWVNTLVIVTSRLEKIT
VSDAKFYFNYYDNQGNIGHSDSYAIFKLEKITKQDIEDYLEKYKNQK
>Q22MF6/691-852
VVLVILAEGGSGKSMLLKKIQIDLTHDSSQLYFPIFIKCNQLNQKYPTIEAVLSSDEYSI
SNFDIIAIKKSSKRQKVILLDGYDEYTGDNFNIYKDLNLSEWNNTKVILTSRKEKLDEHS
IVQFVQVKEKNNNPEASFCILDLLPFNDDDIQIYCQNYIEKY
>Q23D30/917-1043
KLFVIVGEEGFGKSILLKKIELSLIQNEQNTQHLAIPIMISFSDLKKNSFSLEKTIEECT
YFLSSTNLSVETLKKSNYQKLILLDGFQDAQGVFNNLWQDLNLNSWKNTKIILACRDNDY
HAIYNKN
>Q245S3/563-730
IIGILAEGGTGKSMLFKRVETLLMKDKNQNFYTSNEKLNYITLLVKCNNLDSKNPSLDDY

LISQGLDQNQIKQLKKMQRNKLILLDGYDEYTGNYFKVYQKLGLQDWTNTLVIVSSRIEK
FSQSDAVAYFSIEDQYGSRDNNSYCIAKLKEFERKDIDEYCKKFYQKQ
>Q24G77/100-274
KILIQGQAGAGKTTLIQYIAHQWSQKNILSDRFSEIYFISLKTLLNQTWAEQYIIDQKYS
KEFQINPLKFLIHTNVSLMINNQGQQKQIQPEDIKLYNDKILLLIDGFNEITSVQEDHIV
CLILKQIFDQQNIILTSRTNTLSIYWKNKFDIILENIGFNKLQIHQYIENHIDQQ
>Q22D02/994-1159
IISILAEGGSGKSMLLKKLEVELLNRDSEYTKDTRSYFIPFIIKCNSLDRKQPSLEDYLE
SVKIKRKDIDMLKNSERNKLIMLDGYDEYTGEYFKVYEKLKLNDWVNTLVIVTSRLEKIA
ISDAKKYFNYYDNQGKQAKNNNSFGIYKLQKITDKDIEDYLEKYQK
>Q229E9/986-1111
IISILAEGGSGKSMLLKKIEVEVLNDNSKYKSDNRTDFIPFIIKCNSLDKEKPSIEDYLE
SLSTKRKDIDNLKKSERNKLIMLDGYDEYTGDYFKVYEKLNLNEWVNTLVVVTSRLEKIS
ISDAKV
>Q22D03/1113-1281
RIISILAEGGSGKSMLLKKLEVELLKGTDSEYTKDQRSDFIPFIIKCNSLDREKPSIEDY
LQSVNIKKEDVDLLKKSERNKLIMLDGYDEYTGDYFKVYQKLKLNEWVNTLVIVTSRLEK
ITVSDAKKYFSYYDNQGKQGQLNSYAIFKLEKITKQDIEDYLEKYKKQQ
>Q232S8/946-1112
ILSILAESGCGKSTLLKKLQVELLHRKSCYTKNNKSDFIPIIIKCKQLDRQKPSIEDYLE
SENLDKNFIEILKKSEKNKLIMLDGFDEYDGEYFQVYQKLKLNEWVNTLVIVTSRLEKIA
VTDAQNYFKYYNQYGNQGQSDSFTIVKLEKFTDSDIFEYLQKFEKQK
>B7GC09/373-506
KLVSLVGRSGVGKTHLARVFAWEWTKERDKNCTRFGFWLNAATESTLRESYETAIRRLRH
GTSLEEPSAKRRMVTIQSLALRLWETLAQLSLSFEWILVFDNVPAFVEALDGTKREGPLG
FQEWFLPRDWRNGR
>F0YPG5/865-1033
QPVLIMAEAGTGKSWMTKQIACAAAKAVQTGDPAGQFYPLVIYVQELAHLIRSRRDEVAE
GSAFVHLVEAFIRIKFGDQPATCAALLQCFQQRTLCVIVDGIDEAADLKALIENFIFREI
VPLGHRLLVTSRPEAIDAKRYAPSFVLLSLRKLDAEAQALAIRQQVEDN
>F0YI16/72-218
LPLVITGSTGCGKSALLANWVNRRRKMKHRDEFLFQHFVGCSPRSKQLAHLLYRLESALK
EHFQLREMEVPTSEERLRWSLNRFLAAAAKKQFPARIVIVMDAVNRLRGESSAADTLHWL
PTELPQGVRIIVSTVELEQNGSLPDDF
>F0W0Z1/547-714
RLVTISGEEGIGKTAVAYAVANYVGPRMTIEGGVKVISVAQIAQEEHDRTESLYVEKDVS
VRQGIVFKAAKALIERHLKQSSVYFMSGGRSNLLVLDGCDFLTIAGAYRQRFRCYLSHLL
TNNPSLKILLTARTSITDDEALTGVGERVFPLTRFSPKMSAQMLLGLM
>F0WXT3/670-830
RICNLHGAKGIGKSTIAIHVTKYVSQMQCFSPNIQYISLGDYERNILTEQDAADTEATYD
FNVALLHQLHLDIDELHTAVTIDDSRSSLLIFDGCDFHVEAPMCDFLSRILSQFPSFKII
TTSRKKLAISMKKPASIEYLEIHPLPDQSAAKLLIAYCEEP
>F0VZL4/88-239
VPLLLLGESGSGKSAAVANWVERRKRMHQNWQSSTELVFCHVIGCLRQSCLVSNLLERMM
REMKGYFELSIAIPNIEERLSWHLPDFLKAVSSEGRVIFIIDGLDRLCSNDGGSILCWIP
VEFPAYVRVILTGTCESRESTLTEPELRPPQS
>F0WNF2/243-405
HILTIIGEGGSGKSTLAGYICQKFDAKLHAYHFCQFDRKSRSSSRDVVLSLVSQFASKNP
LYKRQLTCLNLRYILKESNPLVMANKLLIEPLRAIPVSNSTRGFVLFDGIDQCLVENESN

DLLDLISHITQRFPSCIGFVVTSKASPAFDAKFKSKSIIHLHE
>D0N920/629-798
RLVTICGEEGIGKTAVAHAVANYVGPRITSEGGVRIFSVAKLAQDEMDEHVGMMRRNINI
ANGRCRVLTRLETMVSDHLKQHKARIEFNGQHMLMVMDGCDYLLRNDSRRDRFRVFLSDI
LTNNASLKIVLTARTSICADGAVRGHGERLYTLSKFDMKSATMMLVSLMS
>D0NX25/331-501
TLLAVVGEGGTGKSTFCGTVAQQFRGNLLAAHCCQFDRKSKSSPRNVLLSMVHQLVDSLP
SFKNQLARLNLKYVLEEADPFLLAGKVLVDPLNAVEEPMHATFILVDGIDQCAVGSNGRN
ELLELFAQVIPQLPSWVGFLVSSKPSSKLAKRLPVSSVLDFSAKNGAFVAD
>D0N2C9/76-264
GPVILLGESGMGKSAFLANWLVRRKKMFQNWQSSYPEFIFSHVVGCSRQSCSVSNLLERI
LREIKEYFELNKEIPDVEERLSWQFPRFLEAASKKGRVILVVDGLHRLHTNNGESILKWV
PLAFPPNVRIIFSGTLNYPTSKLSHTSLSAQMIERIKMEAGRRNWKLIHVLPLAEEDRRR
IVKRFISKS
>F2XX00/185-352
SLFLISGIAAIGKTWLLRKFLLDWSNGFIWKNIDLVFYLECKQLNLYENISNINELLDVF
YKDILKGYNISLDFMQSKPSIMFIIDGLNEFKYFDQLISNTHCSSQEIPILNVFTEIYKY
KAVISGRVNTISQYENVVTRYKDKLTIRVMGYNENGIKYYLRNNLIKK
>A7T3U9/319-487
KRVLLEGNPGVGKTTLCKKLVNSWALGVQRCKQTEHSSNSTSPFPAEVEIVLLLKCRDIV
DAEDWKDILRQEIPENYADEDKERIIEYICNNEKRVALVIDGYDELNTSEKALHKILCKK
ALPQSFILMTSRKNRMQAEVKNCFDASLEIKGIESSSGKQLILDFFRSS
>A7S6Z9/325-498
KPLVLYGDSGCGKTSLVAMTAKLVPSWTEYEPVVVLRFLGTTPDSSNIRLVLRSACLQLC
KAFGTSTKVVPQDYQDLLREFQDRLKMAKSNRPIVVILDSADQLTTTDDAHQMVWLPRKL
PKHVYVLVSTLKDPKYACYPALKKLLLDSNCFVEVSSLPSEVACNILTSWLSND
>A7SV40/356-521
SKVLLEGNPGVGKTSFCKNLVHLWALRVQGNKSNMPLSCIFPEEIKVVLLLHCRDIANAS
DWRDFLRQAIPENYSDEDKERIVRYICDNEKSVALVIDGYDELPASSRKSLNTILSKKAL
PQSCMVVTSRKNHLSELKNSFDRTLEITGWNFDDAKELFTSYFSSS
>A7S460/322-491
KRILLEGNPGIGKTTVSKKLVNSWASHWQGHSERASDDIPPENIFPPETEIVLLVVCHDL
ENPNDWKDVLRQSLPEDLSDSAKDIILSYISDNKERVAIVIDGYDELPASSTKAFDKIFN
SKVLQNSYVIVTSRPNTIKPDMRGFFRKLEIKGFDDLEVKEFISSYFTKS
>A7SMT6/320-492
RPLVIYGESGCGKTSVVSMVAMRSREWLRGDTCVVVRYIGTTPDSSLVRLLVRSVCQQIC
AVYEKDPSFIRNDIKELKEDFIRCLRMATVERPLLIVLDSLDQLSAEDGARQLSWLPMTL
PDHVHLVVSTIPEVKYECFPVLKAFLPPESFVLVPKLPQKDAAEILVSYLRME
>A7SNU5/137-256
RLLILKALPGAGKSAVAIQLAHYILKTQQYHGYQVVFVCLREQKTLACVCRGILHALDVT
ITGDPVSQTRHVLERLSQETLLILDNTEDIQKCDNDFAKFIQYLRQFGHNLQILLTSRLD
>A7SLI4/175-289
KRILIEGKAGVGKSTLVSEMAYDWATNENLAQFSMVFFIELKHVLGRISDAMFDLLPKDF
PITCEQLYKYVQGHQEQVLFILDGYDEIKPSRVGDVHDLISGKILRQSTVILTAF
>A7SA50/316-479
PIMLVQGVPGSGKSSLVAYCTLEAKKLDSLLFYHFVGSGPGSTSPLRVVNRLFAWLRDIT
HYTGNIYPLKHLKNVLNEAGKLNKKVVIIIDALNQLADADHGSSHLDWLQTGCPSNVRVI
VSAVESSRSVRMLINEDRKPCPCEVYIEELDETSRKEMVQHLLG
>A7SY65/330-491

KVLLEGDSGAGKTTLTKKLASDWAKGVLSGSSTFPEVELLLVLKCSEMKEGNEGGGIFQA
IQEQLLPEEITVEERLVIFQYIKHNQGKVMVILDGLDEIPSQANSVKESLNKVISRNALA
LSYILVTSRPDKTLYRHFVGGNILQIKGLCNVDDYISNFFPS
>A7S3R7/302-476
RIVVIHGESGCGKTSIMAKVAMVMRSWMTDPESCAVVYRFLGTSPDSSSTRLLLHSICSQ
LCRIAGVTLARVPEDLKSLSEYLPECLARASRQHKIVLVLDSLDQLTGDTFGQELDWWPK
QLPDNVFAVLSTLPGEEYRCLPYLKALLPETCFMDVPVLSFHEADVILEGWLQEA
>A7RUS0/314-485
SLCVVFGESGVGKTTVIAKAARELQERHSGWVVLFRFCGITPTCSTGRELTQSLCEQIKQ
IYDIQDDVPQDYSELCDAFPKLLAHATREKPLCILIDSLDQLTDEDGARRFLEWLPRHVP
PNVRLIVSTLPDVGGCLLRLKTFGIPEDNFIQVRRMSPCDGKVILDKMLHAA
>A7SZT3/372-523
RSVLLEGDSGAGKTTLCKKIAYDWATGVLAKTESFPQVELVLSLKCKGMSSKGFPQAIQD
QLFPEEFSVQDRVEVVQYIKENQNKVLIIILDGYDELPLEARESIHNVVCKKFYSSSYTLL
TSRPEKKLSKYFTGSNALEIKGFLDVDDFISI
>A7RTN5/645-764
DPYYNNKRAIIDTEDWKDILRQEIPENYAGEDKERIIEYICNNEKSVALVIDGYDELNTS
EKALHKILCKKALPQSFILMTSRRNRMQAEVKNCFDASLEIKGIESSSGEQLIQDYFGSS
>A7RNA8/307-481
RVVVLYGESGCGKTSLMAKIATQVKDWPEEDGVITVCRFIGTSPDSSSIRPLIRSICLQL
CKATGQVTADIPEVRMKALVEYFPECLEKASENQKVVLVLDSLDQLSVDDSGRQMEWLPR
SLPYNVYVIMSTLPGEQYQVLPNLRVRYNPQTLMEVPQIPMSEAGLILDNWLKAA
>A7SFC9/549-712
PPLLVIGDDGSGKSLLLSKWISLQQGSLPGWLLLSHFVGPMSSSSASPVLMLKRLTIQLM
RHVTSSLNLTCDPVHLEEEFPRWLEKVSSKLQGGITIVIDSADRLDGAMSHMQWLMDPLP
VGVRVILSVSQDTCPAAWRSWPSVNIGPLEESDRDGLLSALFDP
>A7STJ6/370-488
KVLLEGDSGAGKTTLTKKLASDWAKGVLSGSSTFPEVELLLVLKCSEMKEGNQGGGIFQA
IQEQLLPDEITVEERLVIFQYIKHNQGKVMVILDGFDEIPSQANSVKESLNKFFSVPLH
>A7RNA9/306-479
RITVLYGESGCGKTSIMAKICALVKEWLRKEAVAVVPRFVGISPDSSGIRTLLRSVCQQL
CRVSGENADNVPEDMKSLREYFPECLKSAATCKTIVLMLDSLDQLSPDDGGRQLDWLPKS
LPDNIYLVMSTLPGDEYECLPNLQAILPDACLKEVPSLPLAEADSILTNLLQAS
>A7SYV4/366-542
RPLVVYGASGSGKTSIMAVIAFRVKEWLGDNAVCIFRFLGTSPGTSNTLLTIKSVCEQIH
QVFGLESLKEDVAEDYTELVRHFHELLSTLSVSAERPLVLVLDSVDQLSPSYNAHLMNWL
PKSLSDHIKIVISVLPGYYEILPTLQRSLTDTNCYIEVPTLSRDTGHEILDAWLDSK
>A7SG70/311-483
QPLVLHGCSGCGKTSIMAMAAKCARDWIHPEACVVLRFLGTTPKSSTIRRLMKSICEQIK
CVYRLTTSIPQGLKDMVELFPSLLASATAKRPLLVLLDSLDQLSPEDGARLLDWLPKHLP
GHCKMIVSTLPGAQYRCFPKLKAMFKNDKLFISVPSLPHSDVSDILSMWLQQS
>A7SFL9/385-574
KGIIITGDPGSGKTSFIANVICSQFSSYSMNIRVLGYHMCISSYRSTKEPAKFVRSVAAM
ISSVIKEYSYITLNNIYIQQKLDTCVNDPVDCFDQVVLSPLRHILDPPSNQMFLVVDALD
ECILETGRKNEIVELLSKTLDQFPSWVKVLLTSQKVPSILTEFSSPHVSLVEIEQDNPNN
LYDIEQYVSH
>A7RTN7/953-1085
SSNSTSPFPAEVEIVLLLKCRDIVDAEDWKDILRQGIPENYADEDKERIIEYMCINEKRV
ALVIDGYDELNTSEKALHKILCKKALPQSFILMTSRRNRMQAEVKNCFDASLEIKGIESS

SGEQLILDYFRSS
>A7RTN7/414-467
QHVLLEGNPGVGKTTLCKKLVNCWALSVQKHEQTDYFVIHQIIKFLQFVKSKGL
>B4XWC0/129-266
RCILIEAEPGGGKTTFMSKEAIDAVSQKTELGRWYDIVLLIRLREVREGETIEEIVWDQC
VPETTEGVDVQTIRTILQRNESRVLFLLDGYDELRPEASADRQAIPKLLSGKVYPHSTIV
ITSRPSTQSCNDYQESLL
>B4XWB9/245-401
RCILIEGEAGGGKTMFLSKEALDAVSQKTELGRRHDIVLLIRLREVREGEAIEEMVWDQC
VPRRKDVNVEFIEAILERNKSRVLFLLDGYDELRPEARAARQAIPKLLSGKMYPNSTIVI
TSRPSAGVQQYTRPDCHVHIMGFSPEHAEKYVRKYFF
>Q94430/82-254
NTVWVVGPPGVGKTTLLKMMVKQILKHEFLPDTEYIFFINVKDIDFNKEMTLLEFLTTNS
RVKVNYTEEESKALITFLHNNPNVAIFFDGLDEASTNEFARIPHICKLDGKSKPVDIMKN
LFNLTLLPKAKIVVTSTLHQMYKLHPDYRPTSIFEVLGLLEEAKNNLGTQLCG
>Q94427/64-236
NTVWVVGPHGGGKTTLLKMMVNQILKHELLPDTEYIFFIYAKDIDFNKEMTLLEFLTTNS
RVKVNYTEEESKALITFLHNNPNVAIFFDGLDEASLKELVGGYSICKLDEKSKPVDIMKN
LFNLALLPKAKIVVTSTPDEMFNLQHCYRPTSIFEVLGFLEEAKNNLGTQLCG
>E4XFR2/1-112
MVLLAEAGNGKTLFARRILKIQQEKYPDTFTFFIHLKAFANKKECSLAEILLPAEFHGTS
PGSQEKRANALKLISDSPDECLFIIDSIEDFDERRKIIEALVTGNVCGDESN
>E4X019/41-240
RGFLLTGNAGAGKSAFIAELCSQKVSENCCSRIHSSVAAHHFVSPFQPRTRKLSFFLKSV
SDQLKTKFAELGDLPTSANGDVRSAANSWHSTLSAIAPLRQRHLLVVDGLELSTEIFNFL
SNLNLPGWLSVVYSVAKGHKRSREMIKLLDKLLQKVTLDDIRKEAVCKDSQQYILRRLES
NQNLRSAVNRDNAEFLSQLH
>Q1AN00/274-444
DTVLVSGEAGSGKSTLLQRLHLLWAREAALLEYLLLFPFSCRRLNTELSELSFKELLFQH
CCWPDRDQDEIFDFIQDHPHLILFTFDGLDELKQSFSDEHRLCCPTQRAPVHVLLFNLIQ
GSLLKGVRKVVTSRPSAVTPVLKKNLCKEVLLKGFSPSGIDCFVRKHHSDP
>B9ZZN5/342-484
QTLFLEGPPGSGKTTVAQFLAFSRAAGSSNAPSGAIDLSGVHLVVHLDCSKVKGHLLQEV
TRLLSAAEKVPMEEELRAALAGSSEVLLLLDGYREGNQVFDESLKRFLRDRSGCRVLITA
CPGQCRALKDTCTSAGVLRLHST
>Q86W24/177-346
QIVVLQGAAGVGKTTLVRKAMLDWAEGSLYQQRFKYVFYLNGREINQLKERSFAQLISKD
WPSTEGPIEEIMYQPSSLLFIIDSFDELNFAFEEPEFALCEDWTQEHPVSFLMSSLLRKV
MLPEASLLVTTRLTTSKRLKQLLKNHHYVELLGMSEDAREEYIYQFFEDK
>B0V3M1/185-353
YTVVLYGPAGLGKTTLAQKLMLDWAEDNLIHKFKYAFYLSCRELSRLGPCSFAELVFRDW
PELQDDIPHILAQARKILFVIDGFDELGAAPGALIEDICGDWEKKKPVPVLLGSLLNRVM
LPKAALLVTTRPRALRDLRILAEEPIYIRVEGFLEEDRRAYFLRHFGDE
>Q96MN2/149-318
RTVIIQGPQGIGKTTLLMKLMMAWSDNKIFRDRFLYTFYFCCRELRELPPTSLADLISRE
WPDPAAPITEIVSQPERLLFVIDSFEELQGGLNEPDSDLCGDLMEKRPVQVLLSSLLRKK
MLPEASLLIAIKPVCPKELRDQVTISEIYQPRGFNESDRLVYFCCFFKDP
>P59044/196-365
LTVVLQGPAGIGKTMAAKKILYDWAAGKLYQGQVDFAFFMPCGELLERPGTRSLADLILD

QCPDRGAPVPQMLAQPQRLLFILDGADELPALGGPEAAPCTDPFEAASGARVLGGLLSKA
LLPTALLLVTTRAAAPGRLQGRLCSPQCAEVRGFSDKDKKKYFYKYFRDE
>E9PE16/172-340
YTVVLHGPAGVGKTTLAKKCMLDWTDCNLSPTLRYAFYLSCKELSRMGPCSFAELISKDW
PELQDDIPSILAQAQRILFVVDGLDELKVPPGALIQDICGDWEKKKPVPVLLGSLLKRKM
LPRAALLVTTRPRALRDLQLLAQQPIYVRVEGFLEEDRRAYFLRHFGDE
>Q96KL4/414-585
RVIAVLGKAGQGKSYWAGAVSRAWACGRLPQYDFVFSVPCHCLNRPGDAYGLQDLLFSLG
PQPLVAADEVFSHILKRPDRVLLILDAFEELEAQDGFLHSTCGPAPAEPCSLRGLLAGLF
QKKLLRGCTLLLTARPRGRLVQSLSKADALFELSGFSMEQAQAYVMRYFESS
>Q7Z494/524-687
PPLLVSGGPGSGKSLLLSKWIQLQQKNSPNTLILSHFVGRPMSTSSESSLIIKRLTLKLM
QHSWSVSALTLDPAKLLEEFPRWLEKLSARHQGSIIIVIDSIDQVQQVEKHMKWLIDPLP
VNVRVIVSVNVETCPPAWRLWPTLHLDPLSPKDAKSIIIAECHS
>Q59EZ2/511-686
RLSLVTGQSGQGKTAFLASLVSALQAPDGAKVAPLVFFHFSGARPDQGLALTLLRRLCTY
LRGQLKEPGALPSTYRSLVWELQQRLLPKSAESLHPGQTQVLIIDGADRLVDQNGQLISD
WIPKKLPRCVHLVLSVSSDAGLGETLEQSQGAHVLALGPLEASARARLVREELALY
>B4DYY2/200-368
YTVVLHGPAGVGKTTLAKKCMLDWTDCNLSPTLRYAFYLSCKELSRMGPCSFAELISKDW
PELQDDIPSILAQAQRILFVVDGLDELKVPPGALIQDICGDWEKKKPVPVLLGSLLKRKM
LPRAALLVTTRPRALRDLQLLAQQPIYIRVEGFLEEDRRAYFLRHFGDE
>E7EQW0/464-618
SVMCVEGEAGSGKTVLLKKIAFLWASGCCPLLNRFQLVFYLSLSSTRPDEGLASIICDQL
LEKEGSVTEMCVRNIIQQLKNQVLFLLDDYKEICSIPQVIGKLIQKNHLSRTCLLIAVRT
NRARDIRRYLETILEIKAFPFYNTVCILRKLFSHN
>Q86W28/204-373
KTVAIQGAPGIGKTILAKKVMFEWARNKFYAHKRWCAFYFHCQEVNQTTDQSFSELIEQK
WPGSQDLVSKIMSKPDQLLLLLDGFEELTSTLIDRLEDLSEDWRQKLPGSVLLSSLLSKT
MLPEATLLIMIRFTSWQTCKPLLKCPSLVTLPGFNTMEKIKYFQMYFGHT
>B2RC97/218-387
HTVVFQGAAGIGKTILARKMMLDWASGTLYQDRFDYLFYIHCREVSLVTQRSLGDLIMSC
CPDPNPPIHKIVRKPSRILFLMDGFDELQGAFDEHIGPLCTDWQKAERGDILLSSLIRKK
LLPEASLLITTRPVALEKLQHLLDHPRHVEILGFSEAKRKEYFFKYFSDE
>E7EQE3/1162-1337
RLSLVTGQSGQGKTAFLASLVSALQAPDGAKVASLVFFHFSGARPDQGLALTLLRRLCTY
LRGQLKEPGALPSTYRSLVWELQQRLLPKSAESLHPGQTQVLIIDGADRLVDQNGQLISD
WIPKKLPRCVHLVLSVSSDAGLGETLEQSQGAHVLALGPLEASARARLVREELALY
>E9PHD1/302-456
SVMCVEGEAGSGKTVLLKKIAFLWASGCCPLLNRFQLVFYLSLSSTRPDEGLASIICDQL
LEKEGSVTEMCVRNIIQQLKNQVLFLLDDYKEICSIPQVIGKLIQKNHLSRTCLLIAVRT
NRARDIRRYLETILEIKAFPFYNTVCILRKLFSHN
>Q9Y239/196-368
ETIFILGDAGVGKSMLLQRLQSLWATGRLDAGVKFFFHFRCRMFSCFKESDRLCLQDLLF
KHYCYPERDPEEVFAFLLRFPHVALFTFDGLDELHSDLDLSRVPDSSCPWEPAHPLVLLA
NLLSGKLLKGASKLLTARTGIEVPRQFLRKKVLLRGFSPSHLRAYARRMFPER
>E7EPM2/200-368
YTVVLHGPAGVGKTTLAKKCMLDWTDCNLSPTLRYAFYLSCKELSRMGPCSFAELISKDW
PELQDDIPSILAQAQRILFVVDGLDELKVPPGALIQDICGDWEKKKPVPVLLGSLLKRKM

LPRAALLVTTRPRALRDLQLLAQQPIYVRVEGFLEEDRRAYFLRHFGDE
>Q7RTR0/146-314
HTVVLEGPDGIGKTTLLRKVMLDWAEGNLWKDRFTFVFFLNVCEMNGIAETSLLELLSRD
WPESSEKIEDIFSQPERILFIMDGFEQLKFNLQLKADLSDDWRQRQPMPIILSSLLQKKM
LPESSLLIALGKLAMQKHYFMLRHPKLIKLLGFSESEKKSYFSYFFGEK
>B3KN80/196-368
ETIFILGDAGVGKSMLLQRLQSLWATGRLDAGVKFFFHFRCRMFSCFKESDRLCLQDLLF
KHYCYPERDPEEVFAFLLRFPHVALFTFDGLDELHSDLDLSRVPDSSCPWEPAHPLVLLA
NLLSGKLLKGASKLLTARTGIEVPRQFLRKKVLLRGFSPSHLRAYARRMFPER
>C9JLH9/186-352
RVSITIGVAGMGKTTLVRHFVRLWAHGQVGKDFSLVLPLTFRDLNTHEKLCADRLICSVF
PHVGEPSLAVAVPARALLILDGLDECRTPLDFSNTVACTDPKKEIPVDHLITNIIRGNLF
PEVSIWITSRPSASGQIPGGLVDRMTEIRGFNEEEIKVCLEQMFPED
>C9JW09/139-305
RVSITIGVAGMGKTTLVRHFVRLWAHGQVGKDFSLVLPLTFRDLNTHEKLCADRLICSVF
PHVGEPSLAVAVPARALLILDGLDECRTPLDFSNTVACTDPKKEIPVDHLITNIIRGNLF
PEVSIWITSRPSASGQIPGGLVDRMTEIRGFNEEEIKVCLEQMFPED
>Q86UT6/160-325
QTVVLYGTVGTGKSTLVRKMVLDWCYGRLPAFELLIPFSCEDLSSLGPAPASLCQLVAQR
YTPLKEVLPLMAAAGSHLLFVLHGLEHLNLDFRLAGTGLCSDPEEPQEPAAIIVNLLRKY
MLPQASILVTTRPSAIGRIPSKYVGRYGEICGFSDTNLQKLYFQLR
>Q13075/464-618
SVMCVEGEAGSGKTVLLKKIAFLWASGCCPLLNRFQLVFYLSLSSTRPDEGLASIICDQL
LEKEGSVTEMCVRNIIQQLKNQVLFLLDDYKEICSIPQVIGKLIQKNHLSRTCLLIAVRT
NRARDIRRYLETILEIKAFPFYNTVCILRKLFSHN
>B3KTF0/163-316
SPCIIEGESGKGKSTLLQRIAMLWGSGKCKALTKFKFVFFLRLSRAQGGLFETLCDQLLD
IPGTIRKQTFMAMLLKLRQRVLFLLDGYNEFKPQNCPEIEALIKENHRFKNMVIVTTTTE
CLRHIRQFGALTAEVGDMTEDSAQALIREVLIKE
>E9PE50/328-497
RIVILQGAAGIGKSTLARQVKEAWGRGQLYGDRFQHVFYFSCRELAQSKVVSLAELIGKD
GTATPAPIRQILSRPERLLFILDGVDEPGWVLQEPSSELCLHWSQPQPADALLGSLLGKT
ILPEASFLITARTTALQNLIPSLEQARWVEVLGFSESSRKEYFYRYFTDE
>Q66X48/391-562
RVIAVLGKAGQGKSYWAGAVSRAWACGRLPQYDFVFSVPCHCLNRPGDAYGLQDLLFSLG
PQPLVAADEVFSHILKRPDRVLLILDGFEELEAQDGFLHSTCGPAPAEPCSLRGLLAGLF
QKKLLRGCTLLLTARPRGRLVQSLSKADALFELSGFSMEQAQAYVMRYFESS
>Q32MH9/172-340
YTVVLHGPAGVGKTTLAKKCMLDWTDCNLSPTLRYAFYLSCKELSRMGPCSFAELISKDW
PELQDDIPSILAQAQRILFVVDGLDELKVPPGALIQDICGDWEKKKPVPVLLGSLLKRKM
LPRAALLVTTRPRALRDLRILAQQPIYVRVEGFLEEDRRAYFLRHFGDE
>Q9HC29/293-463
DTVLVVGEAGSGKSTLLQRLHLLWAAGQDFQEFLFVFPFSCRQLQCMAKPLSVRTLLFEH
CCWPDVGQEDIFQLLLDHPDRVLLTFDGFDEFKFRFTDRERHCSPTDPTSVQTLLFNLLQ
GNLLKNARKVVTSRPAAVSAFLRKYIRTEFNLKGFSEQGIELYLRKRHHEP
>A8K9F8/163-316
SPCIIEGESGKGKSTLLQRIAMLWGSGKCKALTKFKFVFFLRLSRAQGGLFETLCDQLLD
IPGTIRKQTFMAMLLKLRQRVLFLLDGYNEFKPQNCPEIEALIKENHRFKNMVIVTTTTE
CLRHIRQFGALTAEVGDMTEDSAQALIREVLIKE

>E7ES59/1-68
MGPDGPGPVLTLFSHLCNGTLLPGCRVMATSRPGKLPACLPAEAAMVHMLGFDGPRVEEY
VNHFFSAQ
>Q9C000/328-497
RIVILQGAAGIGKSTLARQVKEAWGRGQLYGDRFQHVFYFSCRELAQSKVVSLAELIGKD
GTATPAPIRQILSRPERLLFILDGVDEPGWVLQEPSSELCLHWSQPQPADALLGSLLGKT
ILPEASFLITARTTALQNLIPSLEQARWVEVLGFSESSRKEYFYRYFTDE
>C9JK73/211-381
RTVVMQGAAGIGKSMLAHKVMLDWADGKLFQGRFDYLFYINCREMNQSATECSMQDLIFS
CWPEPSAPLQELIRVPERLLFIIDGFDELKPSFHDPQGPWCLCWEEKRPTELLLNSLIRK
KLLPELSLLITTRPTALEKLHRLLEHPRHVEILGFSEAERKEYFYKYFHNA
>Q29675/366-537
RVIAVLGKAGQGKSYWAGAVSRAWACGRLPQYDFVFSVPCHCLNRPGDAYGLQDLLFSLG
PQPLVAADEVFSHILKRPDRVLLILDAFEELEAQDGFLHSTCGPAPAEPCSLRGLLAGLF
QKKLLRGCTLLLTARPRGRLVQSLSKADALFELSGFSMEQAQAYVMRYFESS
>A8K9G6/204-372
YTVVLYGPAGLGKTTLAQKLMLDWAEDNLIHKFKYAFYLSCRELSRLGPCSFAELVFRDW
PELQDDIPHILAQARKILFVIDGFDELGAAPVALIEDICGDWEKKKPVPVLLGSLLNRVM
LPKAALLVTTRPRALRDLRILAEEPIYIRVEGFLEEDRRAYFLRHFGDE
>Q8WX94/172-340
YTVVLHGPAGVGKTTLAKKCMLDWTDCNLSPTLRYAFYLSCKELSRMGPCSFAELISKDW
PELQDDIPSILAQAQRILFVVDGLDELKVPPGALIQDICGDWEKKKPVPVLLGSLLKRKM
LPRAALLVTTRPRALRDLQLLAQQPIYVRVEGFLEEDRRAYFLRHFGDE
>Q7RTR2/139-305
RVSITIGVAGMGKTTLVRHFVRLWAHGQVGKDFSLVLPLTFRDLNTHEKLCADRLICSVF
PHVGEPSLAVAVPARALLILDGLDECRTPLDFSNTVACTDPKKEIPVDHLITNIIRGNLF
PEVSIWITSRPSASGQIPGGLVDRMTEIRGFNEEEIKVCLEQMFPED
>B3KTE7/211-381
RTVVMQGAAGIGKSMLAHKVMLDWADGKLFQGRFDYLFYINCREMNQSATECSMQDLIFS
CWPEPSAPLQELIRVPERLLFIIDGFDELKPSFHDPQGPWCLCWEEKRPTELLLNSLIRK
KLLPELSLLITTRPTALEKLHRLLEHPRHVEILGFSEAERKEYFYKYFHNA
>B4DZL7/184-352
YTVVLYGPASLGKTTLAQKLMLDWAEDNLIHKFKYAFYLSCRELSRLGPCSFAELVFRDW
PELQDDIPHILAQARKILFVIDGFDELGAAPGALIEDICGDWEKKKPVPVLLGSLLNRVM
LPKAALLVTTRPRALRDLRILAEEPIYIRVEGFLEEDRRAYFLRHFGDE
>C3VPR7/139-305
RVSITIGVAGMGKTTLVRHFVRLWAHGQVGKDFSLVLPLTFRDLNTHEKLCADRLICSVF
PHVGEPSLAVAVPARALLILDGLDECRTPLDFSNTVACTDPKKEIPVDHLITNIIRGNLF
PEVSIWITSRPSASGQIPGGLVDRMTEIRGFNEEEIKVCLEQMFPED
>B7ZLE8/464-618
SVMCVEGEAGSGKTVLLKKIAFLWASGCCPLLNRFQLVFYLSLSSTRPDEGLASIICDQL
LEKEGSVTEMCMRNIIQQLKNQVLFLLDDYKEICSIPQVIGKLIQKNHLSRTCLLIAVRT
NRARDIRRYLETILEIKAFPFYNTVCILRKLFSHN
>P59045/147-316
LNVFLMGERASGKTIVINLAVLRWIKGEMWQNMISYVVHLTAHEINQMTNSSLAELIAKD
WPDGQAPIADILSDPKKLLFILEDLDNIRFELNVNESALCSNSTQKVPIPVLLVSLLKRK
MAPGCWFLISSRPTRGNNVKTFLKEVDCCTTLQLSNGKREIYFNSFFKDR
>E9PJZ8/196-365
LTVVLQGPAGIGKTMAAKKILYDWAAGKLYQGQVDFAFFMPCGELLERPGTRSLADLILD

QCPDRGAPVPQMLAQPQRLLFILDGADELPALGGPEAAPCTDPFEAASGARVLGGLLSKA
LLPTALLLVTTRAAAPGRLQGRLCSPQCAEVRGFSDKDKKKYFYKYFRDE
>Q9H6Y0/1-68
MGPDGPGPVLTLFSHLCNGTLLPGCRVMATSRPGKLPACLPAEAAMVHMLGFDGPRVEEY
VNHFFSAQ
>A8K407/211-381
RTVVMQGAAGIGKSMLAHKVMLDWADGKLFQGRFDYLFYINCREMNQSATECSMQDLIFS
CWPEPSAPLQELIRVPERLLFIIDGFDELKPSFHDPQGPWCLCWEEKRPTELLLNSLIRK
KLLPQLSLLITTRPTALEKLHRLLEHPRHVEILGFSEAERKEYFYKYFHNA
>Q86WI3/222-383
RVTVLLGKAGMGKTTLAHRLCQKWAEGHLNCFQALFLFEFRQLNLITRFLTPSELLFDLY
LSPESDHDTVFQYLEKNADQVLLIFDGLDEALQPMGPDGPGPVLTLFSHLCNGTLLPGCR
VMATSRPGKLPACLPAEAAMVHMLGFDGPRVEEYVNHFFSAQ
>Q99973/1162-1337
RLSLVTGQSGQGKTAFLASLVSALQAPDGAKVASLVFFHFSGARPDQGLALTLLRRLCTY
LRGQLKEPGALPSTYRSLVWELQQRLLPKSAESLHPGQTQVLIIDGADRLVDQNGQLISD
WIPKKLPRCVHLVLSVSSDAGLGETLEQSQGAHVLALGPLEASARARLVREELALY
>B0V3L6/172-340
YTVVLHGPAGVGKTTLAKKCMLDWTDCNLSPTLRYAFYLSCKELSRMGPCSFAELISKDW
PELQDDIPSILAQAQRILFVVDGLDELKVPPGALIQDICGDWEKKKPVPVLLGSLLKRKM
LPRAALLVTTRPRALRDLQLLAQQPIYVRVEGFLEEDRRAYFLRHFGDE
>Q9ULI1/410-575
NPLIIYGGPCTGKTLLLAEVAKKAYGWLHEDTGPESDPVVIVRFLGTTDMSSDLRTLLLS
VCEQLAVNYRCLVQSYPKKIHDLCDLFINLLNESSLQRPLVIIFDALEQLSENDDARKLW
WLPAHLPRFVRIVLSTLPNKHGILQKLRCLIHEEDNYIELIPRDRK
>Q9NPP4/163-316
SPCIIEGESGKGKSTLLQRIAMLWGSGKCKALTKFKFVFFLRLSRAQGGLFETLCDQLLD
IPGTIRKQTFMAMLLKLRQRVLFLLDGYNEFKPQNCPEIEALIKENHRFKNMVIVTTTTE
CLRHIRQFGALTAEVGDMTEDSAQALIREVLIKE
>B0V3L8/172-340
YTVVLHGPAGVGKTTLAKKCMLDWTDCNLSPTLRYAFYLSCKELSRMGPCSFAELISKDW
PELQDDIPSILAQAQRILFVVDGLDELKVPPGALIQDICGDWEKKKPVPVLLGSLLKRKM
LPRAALLVTTRPRALRDLQLLAQQPIYVRVEGFLEEDRRAYFLRHFGDE
>B2RCA1/149-318
RTVIIQGPQGIGKTTLLMKLMMAWSDNKIFRDRFLYTFYFCCRELRELPPTSLADLISRE
WPDPAAPITEIVSQPERLLFVIDSFEELQGGLNEPDSDLCGDLMEKRPVQVLLSSLLRKK
MLPEASLLIAIKPVCPKELRDQVTISEIYQPRGFNESDRLVYFCCFFKDP
>P59046/211-381
RTVVMQGAAGIGKSMLAHKVMLDWADGKLFQGRFDYLFYINCREMNQSATECSMQDLIFS
CWPEPSAPLQELIRVPERLLFIIDGFDELKPSFHDPQGPWCLCWEEKRPTELLLNSLIRK
KLLPELSLLITTRPTALEKLHRLLEHPRHVEILGFSEAERKEYFYKYFHNA
>Q149M9/335-511
TPLVLFGPPGIGKTALMCKLAEQMPRLLGHKTVTVLRLLGTSQMSSDARGLLKSICFQVC
LAYGLPLPPAQVLDAHTRVVQFFHTLLHTVSCRNFESLVLLLDAMDDLDSVRHARRVPWL
PLNCPPRVHLILSACSGALGVLDTLQRVLLDPEAYWEVKPLSGNQGQQMIQLLLAAA
>Q96P20/220-389
HTVVFQGAAGIGKTILARKMMLDWASGTLYQDRFDYLFYIHCREVSLVTQRSLGDLIMSC
CPDPNPPIHKIVRKPSRILFLMDGFDELQGAFDEHIGPLCTDWQKAERGDILLSSLIRKK
LLPEASLLITTRPVALEKLQHLLDHPRHVEILGFSEAKRKEYFFKYFSDE

>Q8NF06/121-287
RVSITIGVAGMGKTTLVRHFVRLWAHGQVGKDFSLVLPLTFRDLNTHEKLCADRLICSVF
PHVGEPSLAVAVPARALLILDGLDECRTPLDFSNTVACTDPKKEIPVDHLITNIIRGNLF
PEVSIWITSRPSASGQIPGGLVDRMTEIRGFNEEEIKVCLEQMFPED
>B0V3L9/207-375
YTVVLYGPAGLGKTTLAQKLMLDWAEDNLIHKFKYAFYLSCRELSRLGPCSFAELVFRDW
PELQDDIPHILAQARKILFVIDGFDELGAAPGALIEDICGDWEKKKPVPVLLGSLLNRVM
LPKAALLVTTRPRALRDLRILAEEPIYIRVEGFLEEDRRAYFLRHFGDE
>P33076/414-585
RVIAVLGKAGQGKSYWAGAVSRAWACGRLPQYDFVFSVPCHCLNRPGDAYGLQDLLFSLG
PQPLVAADEVFSHILKRPDRVLLILDGFEELEAQDGFLHSTCGPAPAEPCSLRGLLAGLF
QKKLLRGCTLLLTARPRGRLVQSLSKADALFELSGFSMEQAQAYVMRYFESS
>A8MTQ2/211-381
RTVVMQGAAGIGKSMLAHKVMLDWADGKLFQGRFDYLFYINCREMNQSATECSMQDLIFS
CWPEPSAPLQELIRVPERLLFIIDGFDELKPSFHDPQGPWCLCWEEKRPTELLLNSLIRK
KLLPELSLLITTRPTALEKLHRLLEHPRHVEILGFSEAERKEYFYKYFHNA
>B7Z889/1-147
MVLDWCYGRLPAFELLIPFSCEDLSSLGPAPASLCQLVAQRYTPLKEVLPLMAAAGSHLL
FVLHGLEHLNLDFRLAGTGLCSDPEEPQEPAAIIVNLLRKYMLPQASILVTTRPSAIGRI
PSKYVGRYGEICGFSDTNLQKLYFQLR
>B0V3M0/183-351
YTVVLYGPAGLGKTTLAQKLMLDWAEDNLIHKFKYAFYLSCRELSRLGPCSFAELVFRDW
PELQDDIPHILAQARKILFVIDGFDELGAAPGALIEDICGDWEKKKPVPVLLGSLLNRVM
LPKAALLVTTRPRALRDLRILAEEPIYIRVEGFLEEDRRAYFLRHFGDE
>C9JZX9/211-381
RTVVMQGAAGIGKSMLAHKVMLDWADGKLFQGRFDYLFYINCREMNQSATECSMQDLIFS
CWPEPSAPLQELIRVPERLLFIIDGFDELKPSFHDPQGPWCLCWEEKRPTELLLNSLIRK
KLLPELSLLITTRPTALEKLHRLLEHPRHVEILGFSEAERKEYFYKYFHNA
>Q86W26/167-331
SLVVLQGSAGTGKTTLARKMVLDWATGTLYPGRFDYVFYVSCKEVVLLLESKLEQLLFWC
CGDNQAPVTEILRQPERLLFILDGFDELQRPFEEKLKKRGLSPKESLLHLLIRRHTLPTC
SLLITTRPLALRNLEPLLKQARHVHILGFSEEERARYFSSYFTDE
>Q86W25/229-402
QTIVLVGRAGVGKTTLAMQAMLHWANGVLFQQRFSYVFYLSCHKIRYMKETTFAELISLD
WPDFDAPIEEFMSQPEKLLFIIDGFEEIIISESRSESLDDGSPCTDWYQELPVTKILHSL
LKKELVPLATLLITIKTWFVRDLKASLVNPCFVQITGFTGDDLRVYFMRHFDDS
>B7ZKS9/220-389
HTVVFQGAAGIGKTILARKMMLDWASGTLYQDRFDYLFYIHCREVSLVTQRSLGDLIMSC
CPDPNPPIHKIVRKPSRILFLMDGFDELQGAFDEHIGPLCTDWQKAERGDILLSSLIRKK
LLPEASLLITTRPVALEKLQHLLDHPRHVEILGFSEAKRKEYFFKYFSDE
>C9J2Y8/200-376
TPLVLFGPPGIGKTALMCKLAEQMPRLLGHKTVTVLRLLGTSQMSSDARGLLKSICFQVC
LAYGLPLPPAQVLDAHTRVVQFFHTLLHTVSCRNFESLVLLLDAMDDLDSVRHARRVPWL
PLNCPPRVHLILSACSGALGVLDTLQRVLLDPEAYWEVKPLSGNQGQQMIQLLLAAA
>D6CHF9/77-247
DTVLVVGEAGSGKSTLLQRLHLLWAAGQDFQEFLFVFPFSCRQLQCMAKPLSVRTLLFEH
CCWPDVGQEDIFQLLLDHPDRVLLTFDGFDEFKFRFTDRERHCSPTDPTSVQTLLFNLLQ
GNLLKNARKVVTSRPAAVSAFLRKYIRTEFNLKGFSEQGIELYLRKRHHEP
>Q8TDZ4/302-456

SVMCVEGEAGSGKTVLLKKIAFLWASGCCPLLNRFQLVFYLSLSSTRPDEGLASIICDQL
LEKEGSVTEMCMRNIIQQLKNQVLFLLDDYKEICSIPQVIGKLIQKNHLSRTCLLIAVRT
NRARDIRRYLETILEIKAFPFYNTVCILRKLFSHN
>B5MDB6/139-305
RVSITIGVAGMGKTTLVRHFVRLWAHGQVGKDFSLVLPLTFRDLNTHEKLCADRLICSVF
PHVGEPSLAVAVPARALLILDGLDECRTPLDFSNTVACTDPKKEIPVDHLITNIIRGNLF
PEVSIWITSRPSASGQIPGGLVDRMTEIRGFNEEEIKVCLEQMFPED
>Q9NX02/207-375
YTVVLYGPAGLGKTTLAQKLMLDWAEDNLIHKFKYAFYLSCRELSRLGPCSFAELVFRDW
PELQDDIPHILAQARKILFVIDGFDELGAAPGALIEDICGDWEKKKPVPVLLGSLLNRVM
LPKAALLVTTRPRALRDLRILAEEPIYIRVEGFLEEDRRAYFLRHFGDE
>P59047/280-447
RTVVLHGKSGIGKSALARRIVLCWAQGGLYQGMFSYVFFLPVREMQRKKESSVTEFISRE
WPDSQAPVTEIMSRPERLLFIIDGFDDLGSVLNNDTKLCKDWAEKQPPFTLIRSLLRKVL
LPESFLIVTVRDVGTEKLKSEVVSPRYLLVRGISGEQRIHLLLERGIG
>F2Z321/211-381
RTVVMQGAAGIGKSMLAHKVMLDWADGKLFQGRFDYLFYINCREMNQSATECSMQDLIFS
CWPEPSAPLQELIRVPERLLFIIDGFDELKPSFHDPQGPWCLCWEEKRPTELLLNSLIRK
KLLPELSLLITTRPTALEKLHRLLEHPRHVEILGFSEAERKEYFYKYFHNA
>A0N0N9/414-585
RVIAVLGKAGQGKSYWAGAVSRAWACGRLPQYDFVFSVPCHCLNRPGDAYGLQDLLFSLG
PQPLVAADEVFSHILKRPDRVLLILDGFEELEAQDGFLHSTCGPAPAEPCSLRGLLAGLF
QKKLLRGCTLLLTARPRGRLVQSLSKADALFELSGFSMEQAQAYVMRYFESS
>Q6NVH7/6-129
PPLLLLGTPGSGKTALLFAAALEAAGEGQGPVLFLTRRPLQSMPRGTGTTLDPMRLQKIR
FQYPPSTRELFRLLCSAHEAPGPAPSLLLLDGLEEYLAEDPEPQEAAYLIALLLDTAAHF
SHRL
>B0V3L7/172-340
YTVVLHGPAGVGKTTLAKKCMLDWTDCNLSPTLRYAFYLSCKELSRMGPCSFAELISKDW
PELQDDIPSILAQAQRILFVVDGLDELKVPPGALIQDICGDWEKKKPVPVLLGSLLKRKM
LPRAALLVTTRPRALRDLQLLAQQPIYVRVEGFLEEDRRAYFLRHFGDE
>Q32MH8/172-340
YTVVLHGPAGVGKTTLAKKCMLDWTDCNLSPTLRYAFYLSCKELSRMGPCSFAELISKDW
PELQDDIPSILAQAQRILFVVDGLDELKVPPGALIQDICGDWEKKKPVPVLLGSLLKRKM
LPRAALLVTTRPRALRDLRILAQQPIYVRVEGFLEEDRRAYFLRHFGDE
>E9PBV1/129-305
TPLVLFGPPGIGKTALMCKLAEQMPRLLGHKTVTVLRLLGTSQMSSDARGLLKSICFQVC
LAYGLPLPPAQVLDAHTRVVQFFHTLLHTVSCRNFESLVLLLDAMDDLDSVRHARRVPWL
PLNCPPRVHLILSACSGALGVLDTLQRVLLDPEAYWEVKPLSGNQGQQMIQLLLAAA
>E1C894/1-146
MVVDWCHGLLPRFELVIPFSCEDLSHSHVPISLRRLITKKYQHLRDVVPLLGSSNLKVLF
ILNGLERLNLDFRLAGTELCCDANEPVPPSAIVVNLLRKYLLPEASIIVTTRPSAVRRIP
GKYVGRYAEICGFSDTNLQKLYFQLR
>Q5F3J4/168-335
ITVLVGASGMGKTMTIRKVMMEWVEGTLCTQFDYVFCIDCKELSFSKEVSMVDLISKCCP
QQRMPAGRILGNPEKILFIFDSFEALGLPLAQPKDELSTDPTEAKPLETTLLSLLRRTVL
PESSVLIATRPAALQSLGQCLEGKHYVEILGFSPAAREEYFHRYFGND
>E1BR67/201-373
ETIYVFGDAGIGKSILLQKIQSLWAKKQLDIGAKFFFHFRCRMFSCFKEDEAVCLKDLLF

RYNCYPDQDPTEVFHHILQFPHTVLFTFDGFDEIYSNFDLSSVPEMCSPDEPMHPLVLLV
SLLRGKLLKGSKKILTARTGTEIQRNIIRKKVLLRGFSNSNLKEYTAMFFKDV
>F1NLB4/69-235
RISVTTGVAGIGKSTLVKLFVGRWTKGLINRDIMFVLPLTFRELNTYEKLSAERLIRSSF
PHITEPNCISTGAARTLLILDGLDEFKTPLDFSNTVACTDPKKEIQVDNLITNIIRGNLL
QEASIWVTSRPTAASQIPGGLVDRMTEIRGFRATEMKDFLDQMFLDN
>E1BSU8/508-672
PPLLVSGGPGSGKSLLLSKWIHLQQKHSPNTLILYHFVGRPMSTSSESALIIKRLTLKLM
QHSWLVSPLTLDPAKLLEEFPRWLEKLSARHQGSIIIIIDSIDQIQQAEKHMKWLIDPLP
VNVRVIVSVNVETCPQAWRLWPTLHLDPLNSKDVKALISAECSSA
>F1NVZ4/168-335
ITVLVGASGMGKTMTIRKVMMEWVEGTLCTQFDYVFCIDCKELSFSKEVSMVDLISKCCP
QQRMPAGRILGNPEKILFIFDSFEALGLPLAQPKDELSTDPTEAKPLETTLLSLLRRTVL
PESSVLIATRPAALQSLGQCLEGKHYVEILGFSPAAREEYFHRYFGND
>F1NAC7/41-211
KVIVVLGKAGMGKSVLVQKICQDWSNGEFPQFEFVFWFDCKQISLPEKWYSLKDLLLDFF
VRPQEGSKEIFEYILQNSTKVLLVFDGLEGLHGHENSPHCSASQPNKDLCRVKELLSGLI
QKKILNGCTLLLTARTKEKVCQYVSKVDKTIEIVGFSPWQRELYISKYFEG
>F1NYZ9/234-396
KVIFLFGKPGTGKTMLMHRICQKWAEGVLHQFLFTFLFEFRQLNLLKRKLTLKELLFDLF
LQPEDSPDAVFQHLLENAQRTLIIFDGLDEFVGSLNRSSSMSISELFAELCHGNLLPGCT
VLVTSRPKRVPDFLINTVDLLAEVWGFDHEKVEEYVSHYFRHH
>Q9VIU8/479-613
QPLVVSGARGSGKSVLVSKIMENVHRWKPEAQLILRYANLSARSSDLTSLLGSMANQMSV
LETGHQCQVPHTLEGYSRVIREILDRQQRFFVLIIDGVDELQRDELLDWLPQQLGSAKMI
LTVSELADEGEDVDG
>Q9VEZ2/383-562
KPLVLFGDGGCGKTSLLSKSVSLVATEWFAHVRPINVIRFLGTTPDSSALTATLISICQQ
ISYNYMLPFENIPDDLVPLTAHFKQLLTYASPTQPLTIYLDSVDQLTGTQDSNKVSWIPT
RLPPHCKIIISCANEPANPTVSHEYHVLCKMIDVEENFIEVTALGEDLAMNVIKMWMKTA
>E9FZW8/26-125
NIHDCGNAFSRSLLRHLLKMGDNRVVVMLDGFDEISSQLQEKAIHLMKAIIANKSVQLIV
TTRPHMMDQLQLQLSQLAYSLENFTEKDQIDYLSEYWETN
>E9FRI6/1-73
MLDGFDEISSQLQEKAIHLMKAITFNKSAQLFVTTRPHMIDQLQFQLSQLAYSLENFTEK
DQIDYLSEYWETN
>E9HFF2/383-562
QPLTLFGAGGCGKTSLLARSGIPSAIKINKPIKPVLIMRYLGTTPDSSAITPMLISLCQQ
ISYNYMIPFDAIPDDLVPLTAYFKQLLALATREQPLLVFLDSVDQIGGAQDANKMAWLPT
RLPPFCKIVVSCVYEPENPEISKDCQTLRRMIDEEDNFIHVLALGEELASQVIRHWMRNS
>E9GG01/334-483
QRLLIMGETGYGKTTLLLRLLNDWSTQDPALGYLARFKLVYFLSCRDLSSQRVNLFGSPT
KEEAELAAALASDTENQTLFLLDGLDELNNWPEEMKDLLEGRLYPASTVLATSRPVPSAV
SHPAFHKRIIIHGFELVHVESFIRSFFATP
>E9GQ39/657-816
SVVIISGLAGTGKSTLLCHYYEEIKKASPDHWVIRINLVDYEAILKKDNIARSANIFVYQ
LHVVDEKSSFSRSLLRNRLERGDRIIFMFDGFDEVNDLCQNNAIQLMEAISKKKSIQLYV
TTRPHMLEDLQFWLSQLAYSLENFTEIDQIDYLTSNWAKE
>E9G0B2/237-400

SCFIITGVAGSGKSTALSRHYEHVKTANSNVWVITINLIKHSKHLSKLAINTASDAVDFL
VNFPGVTGDGAFARSLLKNRLINTGGVVLMLDGFDEINNQCKDTIIRLIKAISNGKTKLD
RLLITTRPHLAKRLQDELFQFAFSLENLGKDHQVEYLMKYWQNK
>E9HWR0/716-878
SIVIISGVAGTGKSTLLSHFYKEIKTKKPDYWVIKMNLVDRCEAMMKWYDGKPTNAADFF
VNYLHEIGNKDSFTISLLRHRLEKGDRIVIMLDGFDEIDARCQMGAIQLMKFIGKNKSIQ
LYVTTRPHSVDDLQNKLFQFAYTLEMFDENDQIKYLTKYWETK
>E9GQ43/599-760
SIVIIFGVAGTGKSTLLSYYYKQIKTAHPDHWVIRINLVDYEAVLKLDQITDLDVVDLFI
NRLHVVDSKSQFFRSLLRKRLETGDRIVVMFDGFDEINELCQKGAIEWMKAISKNKSIRL
YVTTRTHMSGDLQFQLSQLAYSLENFTEKDQIDYLTSYWIKE
>E9G0J1/256-420
GVVIISGAAGSGKSSILSHYYEEIKEKNPNHWVIIINFTDHSKDFLRLNLSEVNLSTTIE
CIINLSVVVGHSRLGRSMLRRRLNLGERITILFDGFDEIVKSQYQKTAIRMINVLKEKKS
VGLYVTTRSHMANNLQFWLSQLNFDLENFSKEDQINYLTSLWKQN
>E9FZM6/832-994
RVFIISDLAGTGKSTILSNYYEKIKRDNPDVWVIKIDLLDYSKELAEFNFSLVNDQSSAF
AFFANIFAKDSCFTRSLLSHRFQTDGRIVVMLDGFDEIDGKLHEQVLHLIKAITLTKFNA
IYITTRPHLMGKLQDEFFQFSYTFKNFSQEDQVDCLSKYWGSK
>E9FZW2/727-849
SVVIISSVAGTGKSTLLSQYYKEIKKAKPDHWVIRINLVDQHEAILKLGKTVKRSNVVDY
FVNQLHVIDEKRSFSRSLLRKRLETRERIVIMFDGFDEINDLCQEKAIELIKAITKESLA
NFE
>E9HWR1/614-777
SIVIISGVAGTGKSTLLSHFYKEIKTKKPDYWVIKMNLVDRCEAIVKWYDGKPTNAADFF
VNYLSETGNNDSSFTNSLLRYRLEKGDRIVIMLDGFDEIDARCQNSAIQLMKAISKNKSI
QLYVTTRPHSVDDLQNELFQFSYTLEIFDMNDQIRYLTQYWETK
>E9G0X8/553-713
DVVIISGVAGSGKSSILSHYYEVIKEKKPEHWVIVISLTDHSEAFSKLKSTEVNKTSVTD
FLLNLAIVDRSPFTCSLLKHRLETGDRMVIMLDGFDESHHQETVIEMIKIFKKMQLNGLF
VTTRSHMTSQIQLELSQLSYSLSNFSKRDQVEYLTSVWQRD
>E9HGG4/228-382
CRVLIEGNPGYGKTTLTLKMAFDWAAKKEYIDKFHLVFLIPLRDFQGDLQSYLFNEFFPQ
HESLEDREDWWNYIKENHQSILFILDGLDELSSEHRAPIDMLLKGNIFDKVSVVVTSRSV
SSDDNILSYFNRRIEIRGFGLGQTEQFIQNYFQIK
>E9GQG0/749-921
STAIITGVAGSGKSTILSRHYEHVKKNNSNVWVIKINLINHSKYLAELKIPASGSQEASY
STSTAIDILINFPGVTSQSEFARSLLKNRLRHSGGVVLMLDGFDEINFECQTNIICLMKA
LGNGKTKLDRLIITTRPHWTEKLQKALFQLAFSLEHLGKEHQVNYLTNYWQKK
>E9GQ45/603-764
SIVIIFGVAGTGKSTLLCHYYEEIKRAKPDHWVIRINLVDYEAVLKLDQITDLDVVDLFS
NRLHVVDHNSQFFRSFLRKRLEKGDRIVVMFDGFDEINELCQKGAIEWIKAISKNKSIRL
YVTTRTHMSGDLQFQLSQMAYSLENFAEKDQIDYLTSYWIKE
>E9G006/683-844
SVVIISGVAGTGKSTLLCHYYEVIKRAKPDHWIIRMNLVDYEDILKLDNITDFNVADLFI
NRLRVVDEKSSYSRSLLRNRLKTGDRIIFMFDGFDEVNKLCQDKAIELMKVITKNKSTQL
YVTTRPHMLDKLQFQLSQLSCNLENFTENDQIDYLASYWEKE
>E9GQ37/377-538
GVVIISGVAGTGKSSILSHYYEQIKNKDPGHWVILINLSDHSEAFSKLDYRSIDKLTVVH

```
FLINLSVVTGQSKFARLLLKHRLETGDRIVVMLDGFDESHYKDNVILVINTLKQMKSVGL
YVTTRSHTAGYLQLQLSQLAYNLDDFSKENQINYLTSLWKRE
>E9G2X3/716-881
KVVIVSDIPGIGKSDLLTHLYRLIKKARPYDWVVRLNFLDCFNLFDLNGNLDNVISAVDF
LTNNLIPSLKTDHFSNSLFRHRLLNKTTGGLILMFDGFDEMKDCYQNNAARLINMLLQTK
IDRIFVTTRPHMKENLEDNFFHFAYEIEDFNQEDQENFLTNYWVNK
>E9G004/713-876
KAVIISGVAGTGKTTILSYFYREIKKAKADHWVIRVNLTDNFQALSEFSANVTSLSNAIN
FFTDNLPAIVNNSWFARSLLRHRLETSDRIVLMLDGFDEIGGQLQEKVIRLLNIIQTKVA
RLFITTRNYFNGKLENQLFQFAYTLENFNKHAQADYLNDYWITN
>E9FZY4/787-885
IRDCGDAFSRSLLRHQLKMGDNRVVVMLDGFDEISSQLQEKVIHLMKAIIANKSVQLIVT
TRPHMMDQLQLQLSQLAYSLENFTEKDQIDYLSEYWETN
>E9GQ64/294-455
SIVIISGVAGTGKSTLLSRYYRKIKTVKPDHWVIRINLVDHYEAISKLDLIPSDPVEFFV
NQLHVVDDKSSFSRSLLRHRMETGDRIVVMFDGFDEINDQCQENAIQLMKAITKDRLIRL
YVTTRPHMLDELQFQLSQLAYNLENFTERDQIDYLTSYWEKE
>C4Q8P8/425-600
DPFVLYGQSGSGKTSVMAKLAVTARDWINEIIIVIIRFLGTSPGTSTLRQTLKYTCRQLV
ANINSIGSMSNSDTFTFIDPEVIRAQMIFRHVLIFFDAIDQLEPSDGAYFLGWIRVPLPR
YVKLIISTLPHIGGILDNFRSNFLSHNSLTYPNFIEVKVLDDSMCEQLLRTRLASN
>B3S9T1/655-828
SPLVITGASGMGKSALMSACVLTLQKMNFDVFYHFIGASPSSTSVHEMILRACFYMKDRY
REKLKASDEDIDSLSRISTSELFHYFRQWTEDIATAITSPFVLIFDALNQLGNCDLSWLP
KNLHGKVRILFSAIDGSAELSKLMEHPLQLQQLQLTQLDTNARKEIVVHILAQY
>B3RI54/356-506
QPLVLLGPSGCGKTSLISAATRECKKWLGSSGCVVVRYCGTSPESGNIRLLLRSVCQQFR
KIYNGDPATTPETYKELRDDLMKRLKMATTFFPQVLIIDGLDQLSDDDNAHEIQWLPKTL
PEYVKVILSVRSDSSSPALRSLRVNFIHCDL
>B3SEK7/1065-1224
NFVILSADAGFGKTALLINRINEWAGDENLESILSWKLYVHLPSQRFDDFQDLQTFVKKL
VRDFTLKDWQLKALLNDMEKEDRVELLFDGLDEIKDENKIITFNKLLTEISDTTAIVITT
RPYAAYKVDKPIKRNLGLYLTLARYTDEQRDKYIKMMIPK
>B3SEA3/760-916
NFSILLADAGHGKTAFCLNERYKWKEYEDEISWRIFISLPKIQLSSTPNLFSIFTTAATG
HEWENWQLEILQEDMKKTGKVFLLLDAFDEMKDQDQIDNLRLWLDIIPSSTSILITTRPY
AIKDIELPKFHQLVKYFRLQQYNKPQLKTYIKRYIEK
>B3RS36/500-667
PPLLIIGDQGSGKSLLSAKWIEEYQKLHPDCIILYHFVGLPNSISADSLLMLKRLTMQLM
RHTRQRNKPTNDPFKLQEDFLTNLEKVATERNSRVILLLDSADRFRSCETELKWLLDPFP
SEVKVILTCKEGTIPEAWRPWPSVYIRRMTEEHCKLLLKSLTDNIISF
>B3S5H5/773-929
NFSILLADAGHGKTAFCLNERYKWKEYEDEISWRIFISLPKIQLSSSPKLSSIFTSEATG
HEWENWQLEIIQKDMEETGKVFLLLDAFDEMKDQDQINNLRLWLDIIPSSTSILITTRPY
AIKDIKLSKNHQFGKYFQLQQYSEKQLKIYISRYIEK
>B3S822/242-401
NFVILSANAGYGKTALFINRLNEWAEDENSESIISWKLYIHLPSQKFDNSLDLETSIKPL
VRDFINKDWQLKALLNDMKKEGRVEFFLDGLDEIKDENKINSLNKLLALIPTSTTVVIAT
RPHAVHKVNKPRNRNLGLYLTLGKYTDQLRNEYIKRMFPK
```

>B3RLF8/403-521
SPLVVYGESGSGKTSIMAKCAQYTKNKYPSANLLLRFLGTSPDSSSIRKLLASLCIQLSR
LYDQDLNCIPNEFSQLIGYFSTLVNRIPKDKPLVIILDSLDQLLPSDGAHRMSWIPRIL
>B3SEA4/706-862
TRSIVLAKAGCGKTSLCLHIKYRWFQQKPSTITWMIYVHLPQLNLSSSPTPDSIFTKLAT
DIDWKSWELSALAQDMKTNSNVTLLLDGFDEIKDENQAQLINQWINDISDKVQVVITSRC
YAANKISLTKNVRINSYKLLEYTDSQRAQYIEEYVKA
>B3RLG1/399-519
LPLIIHGVSGSGKTSVMAKCTQWTGQSYPHCKIIARFLGTTPNSSSISKTIFSVCQQICR
IYDKDETQIPDGYSQLVAYFAMQIQNIPANKPLVIILDSLDQLLPVDGAHRMQWIPNRLP
S
>Q54KD0/648-852
PVFLIQGESGSGKSSLISNWLKQHKEQHPEDLVVSHWIGASPSSNKFTSILIRIMNEIKN
QIEIDQKIANGGSNSSSSSSSSMFSTTSTSSVSWLPEIPDETFESEKIVSEFPQFLQYVM
SHPSLNGKRLVLLIDGLDKLDPRENSQELIWFPRNFPHNVKVIVSSIQGSRQSEVLKKRG
SHILSILPFTEAERKSMVRLYLQKY
>Q54D26/458-632
PFLITSSIGGGKSTVLSNWVKQLSNSNEIDSKKTLVVCFFIGITANNKNRIELLRQLFSI
IKLQYQVSIPLSEDPSSLKEEINYWYGIATKDGRNLIIIYDSIDTIEKGANETLSEALLD
LIPSRYPENVKIVCSCYTDNYEELVQHFQLDKNQHTSVLTGFTDQQQILDFSKSY
>Q54CB5/994-1180
NTVVVTGKPGCGKSSLISYFINKAIPELYQKNKNSTSGKYVVISHFIGSTTDSIDIRKSL
LHICNQLNYKLGLGEQTVSPTTEYSELKVIFSSFLKKSALKASQNSQKVLLVIDGLDQLD
KKNRAHTLDWLPITSPIKLIVSTLDGDHTQSVLRRRRTPPTEIVVAPMEVKDRHSLVLGK
FDEFRKK
>D8SNL8/75-258
QELVVVGPKGTGKSCRLRAMAAALYARGPEKSVIVCVWNLESASSPAVLLRRLLDGLRFA
YSGADEHKPVLEALEALWRMGVSVARLQEFLRERAAAGDDFTFVADGAEALDTRDYSGTK
KVGSKELKDTLLTLEAIVSPYKIVWGASPEARQAEAADSERVAIYCVGGFTKEQREVFLL
HSVL
>D8SS59/155-323
AQLQVQGAKGTGKTTVMSSITGYMLGVQKKPVVYISTRRFVESPADVLQHALLVATRGDE
SVAEMTTLGELVGWCRRRINDPLLFVVDDWDKFEDFPSLMSSLKEAVSFRHILVKLSSTN
SRERRITTVTDKPLPSFDFDGPYNSSELETWIAQSSLDKTELKALERLT
>D8SHP0/81-209
RDVTMTGTAGSGKTMLLAMAVCNHYARGRQEGCRLVAIFDPPACTDPVSTVGTLQVVRRA
LLLAYADQEDKVDAISAMGKWTEIGAFLIRHRPVLVFDAYDVVHHLPDVQQEMDILLKHM
SFLKCLTAT
>D8S6E7/370-554
QELVVVGPKGTGKSCRLRAMAAALYARGPEKSVIVCVWNLERASSPTVLLRWLLDGLCFA
YSGADEHKPVLEALEALWRVGVSVARLQEFLRERAAAGDDFTFVADGAEALGTRDYRGIK
KVDCKEMKDTLLTLEAIVSPYKIVWGVSPEARQAEAADSERVAIYCVGGFTKEQREVFLR
HSMLG
>D8R438/76-248
APIYVTGAKGIGKSCTLRAIAAALINQSEENEKIICIWDMKWALYNHMEALRNALLFAYC
EDEAACRTISQLTSVQEVCNFLSDRRHRSGEVFYFVIDRAESLDRVRSNGEEMEHRNQMK
ALLCRVVRLGRCCIYGVSPAATDIGDRSLQQSQYHMSLGFKDASYKAFMDQSV
>A8HMF2/282-433
SLAFQLSSRFPVACQQLLQQDAVRVSQEVSAEAAFEALLAEPLRRVVAQPDFDGKQVVLL

LDALDEADPPPALVEAPARIDPLTGRPTTARPASARSTVAAAPPPCSNKVVQLLTSLLTR
LPPCVRFLLTTRPDAVGGSVCGLLDRTFAATG
>A9V4P8/976-1155
RPFVILGDSGSGCREAVAQFVTQIVEAGNESDSVMLVRFAGLTSIADSVTQTLRSLLEQA
AAALGALDTDEFQNMASMAVAECFLRRLTAACTLKRPVVVVLEDVDQFVDEPFKEEERAG
LSSWLSAQVPPGIKIILTAKPNGPAHRQLATFLKDSDFSSMDTLSLDEAQRLLSHSLARS
>A9V6Y5/317-482
RAYWINGGGGLGKSVLAAKLLDMWQKSDEYTTAAFFCRHNDARHNGVAGALEALALQLAD
QIPAFKKALERDLEECDDVRRLLGAPERDIKEKFDILLATPLDKVTTPIILVIDALDELR
PGTNRTLFLNMVGKLFSSLKSNVRIVVTSRLEDEIVEPMTRLKALG
>A9V2I3/306-474
GVTIVHGHVGSGKSSLLAKCAVKWDSEPETAVLYHFTGKGGSSDSLSNFSRRLFHFVARR
YHVKLDDLDFDLSLHAWHELLELALNDVPQSLKSPLVVLVDGADEFNKVKQLNDWLPSKK
LLHMSIIVSVVTPLPNAEQIPEATWSSTKMPLMTRRDREQFAKAFLQNY
>A9UXU8/450-626
NPVAVLGPSGSGKSALLANWLVAHETRHPRDRVFVHFCGCDGKSTALHNLLMRLCTACAS
PERAQLLVSASTEALVEQLPELLRSAAKSCQDQGQGRLLVVIDALNQLDNDELPWLGGAT
VHSLAWLPVDLPPNMQLIVSTLPGPCEDELKHRHYVALQTQPLDAPNKTAIAETILG
>A9UWP9/1182-1383
AILLLSGEAASGKTLVLAKSLQSYIRQPGCSDRNVIALIQHGLERPLDVATAFLHRLVDA
FDLASLLPESNRPLQGRLLASTLQSIIRHAVRMLPGHTKLILALDGLPQPGSTSSSKSAR
ERSSPLAELLDLLGGALPSRAMLVVALRAGATLTAAKRSMARYGVGLASSGSTGPPVVLE
VRVGPLSAADRRELVFRTLKRY
>F2UH56/863-1039
GPLVVTGPSGSGKSTVLAQVVTELAKDDATAVIARFAGFTAKAASLTHTLRLLTQQFLYL
VHPEMDSETIPTDFRAIISVLPQHLANLAKLDKRIVLIIDSLDEYVDDENRLDERVGLAA
WLPTVLPDNVRLIVSAREHDRAFEQLSELVQDDAVVTLGPMDEAESKAMFERILSER
>F2USK6/465-620
APFAICGPSGSGKTSLMAHIAGVIKEAHPAAVVVSRFLGTSRESGAARTLMANEPAAAPA
ADFEKLTKQFQAALKLGTADKPLVLVLDSLDQLSDEDQGRNLDWLPLASLPPNCAIVISS
LPDVGGCWEVLKATLPDTNQQHVAKISKDDADGIRS
>F2TVT6/600-763
HPLVVYGKSGCGKTTLMAAAAVETARACVRSSRRSGGKQPVVIARLLGTSASTADARSVM
ASVLEQLACMSSDLRNVLASRCATIAPSVRLLPFKQLSKLFHDMLIMFALDTAPLILFLD
SLDQLSDADDGRELAWLPMRWSSSDLRIIVSTLPDVGPCLASLR
>F2TZ36/404-582
APLVVEGESGSGKSALLANWVHQHTSLHAHDRVFCHFTGCDSASTALRNLLSRLLVFCDP
DMAESLSGMTTQQLCEALPACIASAADTARAQHAGARLVIVLDALNQLENETFPWLPQRL
VHRLDWLPTTFPEGVVLVCSTLPGLCQDALRARAYDRITTAPLSRHDCKAFLEQRLRLA
>F2USK2/466-660
RVFWVKGTGGLGKSVIAAQLIKRYGQASTSKQPLIAAHYFCKHDATNRNDPRKAINTLAF
RLATQLPELREEYKAILQGENEERRNNFTTHADGSEGTVEDAFNVVLAEPLKAALGEDSG
GKGSDVFILIDALDELRAGNSRAQFLRLVGKLLPSLPPCVRIVVTSRPEEDILALLKNLN
PFTIDKQDERQREDL
>F2UEX3/270-433
QPVLVRGPSGAGKSSLMSHIVHDLSRRKDTRCHFHFIGQTTQSGHYINVLQRLLHRLRVW
AGGAVTQDMPHSPNKMMEALPSWLAAASEALKGQTVYIVLDALNQLQQVDRATSLHWLPQ
VWPENFRVIATATSEVAWSHAVLEVPTMDARQRELLIASYLSTY
>F2U5G5/484-645

APFAICGPSGSGKTSLMAHIAGVIKEAHPAAVVVSRFLGTSRESGAARTLMANEPAAAPA
ADFEKLTKQFQAALKLGTARKPLVLVLDSLDQLSDEDQGRNLDWLPLASLPPNCAIVISS
LPDVGGCWEVLKATLPDTNQQHVAKISKDDADGILSTWLADA
>F2TXD6/488-651
QCKTSLMAHIAGTIKEAQPAAVVVSRFLGTSRESGAARTLMANVYRHIHRAVKGKEPAAS
PAADFEKLTKQFQAALKLGTADKPLVLVLDSLDQLSDEDQGRNLEWLPLASLPPNCAIVV
SSLPDVGGCWEVLKATLPDMNQQHVAKISKDDADGILSTWLADA
>F2U3J5/344-506
APFAICGPSGSGKTSLMAHIAGTMKEAHPNAVVVSRFLGTSRESGTARTLMANVYRHIHR
AVKGKEPAASPAADFEKLTEQFQAALKLGTARKPLVLVLDSLDQLSDEDQGRNLEWLPLA
SLPPNCAIVVSTLPDVGGCWEVLKATLPDTNQQHRSVVLGVYE
>F2UCY0/614-791
RVFWVEGTGGLGKSVIAAQFVKRYADGTSSHSTSDSTRPLLAAHFFCKHDHADRNDPRKA
IATLTFRLASQLPSLRQKLLDILKDEEQRDNFVAHAHGTKGSVGDAFDVLLADPLREALD
NRDTHDGDIFILVDALDELRDGNSRAQFLRLVGKQLPSLPPCVRIIVTSRPEEDILVF
>F2UD95/1028-1180
LYLLVQGGTGSGKSAFIRYLEFRLWADRDKDPSCPVPLYVPLAQTNNAETDLMSETLMSI
GLDEAGIEQLKREASFVVLLDGFDELGKPVNLYRSNKLDEWQAHVVVGARSQFLQSLPEY
QSLFTHATQSRVDERYILPFDQTQIEAFLERFV
>F2UTC0/262-463
RVFWIRGTGGLGKSVIAAQLVQRYGSEAQSGGDSHHGDDKPLIAAHFFCKHDDVARNNPL
KAINTLAFRLATQLPELREEYRAILQGANVERRNIFTEHAAGIHDCLKDAFENVLANPLK
AALKKRPQSAGDVFVLIDALDELRDGTSRTKFLRLVGGQLQLLPPCVRFVVTSRPEEDIL
AFFKNLDPFTIDRQDEQQRDDL
>F2U304/633-805
SSRQQRPLIAAHFFCRHGDAARSDARRAISTLAYRLASQLPELRQVYKMVLQDEEQRVSF
AAHADGSKGGVEEAFSVVLAEPLKAALQKRQQQQRQQPQHQHETGDVFILIDALDELCD
GSSREQFLRFITRLLPSLPPCVRLVVTSRPEENVLAFLTSLNPFTIGRQNERQ
>F2UA48/1308-1479
APLILAGVEGSGKSALLAKVATELTDGRAAVITRFLGHSTQGTNLPDVMRSVVFQILSAY
KQDIAFPESIEELKKRFKEVLTLASPSQPLTILFDGIDKLNTASGQFELGLWFPKQLPPH
VKVLLTVSSHAPGAWLECHSLVDKNAAEHFVEVPPLESDDVAAMVDGTLKRE
>F2U3T6/472-645
APFAICGPSGSGKTSLMAHIAGAIKKAHPAAVVVSRFLGTSRESGTARTLMANVYRHIHR
ALKGKKPAAAPAADFEKLTEQFQAALKLGTARKPLVLVLDSLDQLSDEDQGRNLEWLPLA
SLPPNCAIVVSSLPDVGGCWEVLKASLPDTNQQHVAKISKDDADGILSTWLADA
>F2U6S0/390-556
EPFVLYGRSGSGKTSILAKAAHEASAKYAKGGQHQLILRFIGTTSGSTTIVQLLQLICKQ
IRRHYGEPNPDAVKEELAELIQDFKTCLELATAEKPLMLFLDSLDQLSPEGGARQLRWLP
APTLPPHVHMVLSTLPEAKYECFPRLDAMYSVNFVEVVPLNMETDVP
>F2UE13/1327-1571
HACLVHSKAGLGKSAFLAKFCDMHASEHGWVHVLPFFVGAVPGSSSWSSVLSHLCRQLKL
RFGASSSATAQHGTAHDGDDDDDDDGDAGDGGSGNSGGGGGGGGGATRAAALKAKLEARL
KAASKAVPPSHTLVIALDGLNALSSTPTGRHGRRAATGASATGSGAGGGAGGGGGASTGA
QLLEWLPLPLPPNVKLVLSATEGPVTRALQRRLEAETRLRLAKVALRALSARDKAALARS
ILHRY
>D2VFN1/94-233
SNIILVGNSGIGKSMTYKNFVQSCQVFHPKVIPIYLTYEHICGNESFKKTVPIDLIFSEL
EREHSLKRKKSGYSDKLHELSTLLESSDKYLMLIIDNVEKVYELNTKDFPKAHAHIAWLS

TLAGDSSGRIPVTLSCSNFI

#PD NB-ARC
>B5GRA6/312-583
RAALVRELSEHLTAAGTAINTVTITGTGGIGKTTLAVQVAHRVREHFPGGQLYTDLQGAG
NQPVAAAAALGSFLRALGVRDDAIPDSLEDRTALYRSLLAGRRTLVLLDNARDAAQVRPL
LPGTANCAVLVTSRARMTGLEGARPVDLEVLTPGEALTLFTRITGRDRVAAEPEAARATA
AACGFLPLAIRIAASRLAARRTWTVSVLAAKLADEHRRIDELATGDLAVHATFTVGYSQL
EPETARAFRLLSLPDGPDISLDAASALLALQP
>E2Q792/96-358
DIDALTALRAGERPLIVVSGPAGVGKTTLVSRWLRTLDEDFPDGQLYADLRGHAPADPPP
AHHPDTPATQSEVLGRFLRALGAVSVPDDPTEQMALWRSSTADRRMVVMLDNAFTAAQIR
PLLPGGPGCLVTVTSRRMLSGLRLEGAEFHRLDALGTDDGIELLRRTIGNERVESEFPAV
REVVTLCAGLPLAVCLASARLASRPRQPVEALARALTPDSERLTALEVEGEATVRKALDA
SYDVLSEPAALLYRALGALPLRT
>B5GMX2/320-547
TAELDQVRERLAPSPGHRPTAFGPRRLLVSGMPGVGKTALAVQAAHTMAEEFPDGLLHLR
LRAADGTPADCGDVLTGLLRQLGESPERLRAPDGDRLRLDDLIHRCRTLTAGRKLLVLLD
DAVNELQLEPLLPATADAAVLVTSRNQFSMGPGSWTVTLEPLTTEESFGLLAAVIGDARV
RAEPEAAREVVEACARLPLALRAAATRLAVRPHWPVARLARRLADPAA
>E2Q1R4/286-505
HEDRVRELADWLADPGHPAVAVCALAGVPGVGKSTLAVHVAHAARHRFPDGQLYIDLQRT
EPRADRPGAVLGAFLHALGVPQSGIPEGREARAALYRSVLAGRRLLVLLDNARDAAQIRP
LLPGTAGCAVLVTSRTRMVDLAGARLIDLDVMSPEEALQLFTRIVGAERADAEPEAALHT
FAACGFLPLALRMAASRLAARRAWTVSDLAARLADESRRL
>E2PUG7/127-417
RSDDFERLVSVLREASAEASTAAVALCGPGGFGKTTLATQTCDDPRIRNSFSEILWVETG
EGCTAARVVELISDLCVHLAGNRPSFTDPEQAGFHLARLLQGRRALLVVDNVWSAADLSP
FLLGGENCVRLVTTRNVRVCPSAARVVQLGPMAPGEVRELLIRTVGTLDETESARLADVC
GGWPLLASIVGANVSQEVVSGVPAGRVVAETSATIRAYGPQAFDVWDSDQRKNAMGQALS
SSLRSLEESVSIAGHSDLRDRYLSLAVFPPSVPIPMSVLTHWWQTAHGWAP
>E2QAB3/291-539
AHRAEQPVRALITGIAGIGKSGLAAVAAHGAAHHYPDGQLHAELCHADGRPKTPGEVLVR
LLRALGEPRASADHSGFPALTGDVEELVRLYRTRTAGRRLLLLLDDATGDAQIDPLLPAG
TGTAVVVTSRTHLTSVPMAHTVVLDPMGEEEALQLLGATMGAQRMADQPEVARTLVRQCA
GLPLAVRIAGTRLASRPQWPLSELAGRLADPSRRLEELRFGDLGVSDGFRSSFGRLRPRT
RKTLPWLSM
>D5SL79/294-550
GATRLVEGDPGPIALIAGGAGVGKTALAVHWAHEHAADFPDGVLFADLCGYSGTPARQTT
AVLREFLSALGVPAQRLPDTGAGMGTLYRALTSGRRFLVVLDNAAASERVRPLLPGGGTC
TTLVTSRDRLGGLVASDAARPVRVDELPRSDCANLLEALLGPETVRAEPEATERLAVLCD
GLPLAVRITAARFLTGRHRSLAALAGQLGDEQRRLSLLDIEDTGIAAALGLTLAQLPEHA
RRMFRALGTHPGPRFDV
>E2Q034/307-561
ASFVQDLGTQLAAPDGPVMAVSALAGIGGVGKTTLAVHVAHAARPHFPDGQLYIDLQGAG
SHAAQPVAVLGAFLRALGTPDASIPDSLDERAALYRSTLAGRRLLVLLDNARDAAQIRPL
LPGTAGCAALVTSRTRMVDLAGAQLIDLDVMSPEEALQLFTRIVGAERVNAEREAALDVV
AACGFLPLAIRVAASRLAARRTWTVSVLAAKLADERRRLDELQAGDLAVKATFELGYGQL
DPDQARVFRLLGLAD
>B5H3Z0/24-230
ELTLLDRALARHRLITLVGCGGVGKSRLALRAARRVREAFRDGVAWAELAPLQGARLLVA

TVSDACDLSDHTPRTPVDALCEWLADKQILLVLDSCEHLLAPCRDLVGDLLTVVPGLTVL
STGREPLDLHLERVIEVEPLPEDGPEARELLRQRMASARPEPGPYDPVTAARICRRLEGI
PLALELAAGQAARTGTAAVAERLASRF
>B5GMD8/10-271
TTLEHLADSAETGGPSVAVVAGAPGLGKTAFAVHAAHRLAPRFPDGQYCLDLRAMDTEPV
RPDEALLRLLAAFGVAGQAVPRSLEDRAGLLRSLTATRRLLLVLDNAAHENQIRPLLPGS
GTSLTIITSRKSLTGLEAVHRVDLPLLRREEAVALLTRIVGPERVAREAQAARDLADRCG
RLPLALRIAGQRLAARPHETLGKLAALLSHEECRLDLLQTGDLRVRAAFTLSYQQLDDAS
RLLLRRCALAAGPDVSPETAAL
>B5GU15/119-377
IAAFVNEYVGGNVCLINGMAGVGKTALALRGIWNAAESFPDGCLFLNLGGDARDGRGVTP
HAALDALLRLLGVAGECIPPGFDARANLYRSVVQGKKIMIFLDNAYNAAQVIPLLPAEQK
CRVIVTSRNRLHALDDAMHLPMDVLAEEEAITLFRSVGGHRADCADRDTATRIVAHCGGL
PLAIRIAAARFRGNLAWTPRELEGLLADERTRLGVLDDGERSVAAALTVSCRALTGEQRR
LFALLTLYPGLEMSVGSVT
>D5SJQ0/1116-1374
LRQLSERLAGTGSGDAPAPPVLVLAGIGGVGKTCLAVQVAHETRGYFPDGQLYADLQGAG
ARATPPEEALGVFLRALGTPDSAIPDSLDERAALYRAVLDGRRVLVLLDNARDAAQVRPL
LPGTAGCATLVTSRTRMAELAGAHLVDLDAMSPEAALRLFTRIVGEERVAEEREAALDVV
AACGFLPLALRIAASRLAARRAWTISALAAGLADERRRLDELQMGDLAVKATFELGYGQL
EPARARAFRLLSLADGPDI
>B5H0J8/127-393
VADLARLAELLETEGEETEAVTLAVIAGTAGVGKTSLAVHWAHRIRDHFPDGQLYVNLRG
YDPVPPVGPDQVLDRFLRALGVPPARIPADTDDKAALYRSRLAGRRLLVLLDNAATAQQV
RPLLPGTPDCLTVVTSRNRMSGLVARDGARRVTVGVLDEAEAVELLRRTTKEYRRGDDPA
ELTELARLCAYLPLALRIAAERAASRPWMPLRELIGDLRDESALWDALTAEEGEEADAVR
TVFAWSYRALSPDAARLFRLLGLHPGP
>B5GWS0/266-531
RRAELEQLREIVLGLTRETRVAAVGGMPGVGKTFLAVRTAELVMRHFPDGVLYVDLHGYS
PGEPEPHARILVRILNDLGVSPATPTDDGMASAYRNALANRAVLLVLDNARDEDHVRPLL
PAPGASAAIITSRRKLHGLGVRESAGLVDLLPLDDGESAELLRIRLGEDRTRTALPFLPD
LVDHCGGLPLALCVVAARIAHHPARDVAGIVRELRQENTRLRSLDLASQSESVRLSLELS
HRQLPAPAARLLWQLAVHPGPTFSWQ
>B5GTN0/315-570
DEIVRDLTAAATPGSTASAVALAGLGGVGKTALAVHVAHRVRHHYPDGQLFVDLRGAGSE
PVPPGTALYAFLRALGAAASAVPEATPDRAAAFRSLLAGRRVLIVLDNARDFAQIRPLLP
GAPGCAVIVTSRSWLGGFADGRTHALSAMDPRESLALFARIAGERRTRAERAEAAAIVEL
CGHLPLAVRIAASRLCSRPRWPLERLRHRLADERGRLAQLRLGDLSVESVFALSHDRLDR
VDPARSRAFRLLSLLD
>E2PXE1/42-288
AGRGAPRARVLLIAGRPGSGRTALAERLIATVAADYPDGVLRARLTEPGGRRVPTEETAR
ELLDALGVQGPPGADGDELSHLFRQEAAGRRGVILLDDAADAEQVDPLLPDVSACLVVAV
SDGPLTGIPGVRPCTVGGLDPKAAIELLTGYAGSVRVTVDPQAAESVAEECGGQPAALVL
VGGWLAARPSLSVADATRQLRALPDEGEGPPGVARPLVRAFRLAYASLPRPTARMLRLLH
LAPAGLA
>B5GNJ0/323-577
ASFVQDLGTQLAAPDGPVMAVSALAGVPGVGKSTLAVHVAHAARHRFPDGQLYIDLQGAG
SHAAQPVAVLGAFLRALGTPDASIPDSLDERAALYRSTLAGRRLLVLLDNARDAAQIRPL
LPGTAGCAVLVTSRTRMVDLAGARLIDLDVMSPEEALQLFTRIVGAERADAEPEAALDVV

AACGFLPLAIRVAASRLAARRTWTVSVLAAKLADERRRLDELQAGDLAVKATFELGYGQL
DPDQARVFRLLGLAD
>B5GR02/131-340
GPVVLTQVLSGGGGVGKTQLAAAYAHHAQQEEHADLTVWITATEEQQIITAYAHAAALVQ
APGVGGSDPEADAQAFLAWLATTQRRWLIVLDDIADPAAVGRWWPSGRPGAGWTLATTRL
EDAALTGGGRTRVRVDTYTPQEADAYLRERFAHDDAAHLLDDHAGELIKALGFLPLALSH
ATAHMINQHLTTSEYLVQFTDIRTRLDHIL
>B5GUR9/365-643
RAQELEELTMLAAGARGGSGVVMIVGPAGSGKTALALRWCRTAREAFPDGQLFLDLRGHS
DERPMGAREALSGLLRGLGIPYQDIPFNPMQMAELYQRTLASRRVLIVLDNAESADQVRQ
LLPYEAGCLALVTSRNRLGALIVSHGAAMLPLRPLASDEAAELVRSVVGRARTEAEPEAL
ADLVTICGRSPLALRIAAANLALRRDFRIADHVRDLRSGNPLESLAVAGDPDSAVRRAFE
LSYCRLDPAAAEGFRLLGLLPGPATTVAAVAALWGRAVP
>D5SJK4/374-629
DEIVRDLTAAATPGSTASAVALAGLGGVGKTALAVHVAHRVRHHYPDGQLFVDLRGAGSE
PVPPGTALYAFLRALGAAASAVPEATPDRAAAFRSLLAGRRVLIVLDNARDFAQIRPLLP
GAPGCAVIVTSRSWLGGFADGRTHALSAMDPRESLALFARIAGERRTRAERAEAAAIVEL
CGHLPLAVRIAASRLCSRPRWPLERLRHRLADERGRLAQLRLGDLSVESVFALSHDRLDR
VDPARSRAFRLLSLLD
>E2Q0H1/421-696
REGLVAELAALLTAPCDPAVRICAISGIGGTGKTALAVHVAHLLRDRFPAGQLFVDLAGV
TENPADPRAVLGQLLHALGVPEAGVPDELDARAALFRTMLSEQRMLVVLDNAAGAEQIRP
LLPGYSGCAVVVTSRARLTAIPAHAVELEPFRPDEALELLASVIGRSRVAAEPEAARDLV
ATCGHLPLAVRVVACRMLARPGAAVADSLRRLSDERRRLDQLRTGDLDVVACFRLGYDQL
APEPARAFRLLSLPESKTVSLPMAAALLGTDEFEAE
>E2PZZ3/67-333
VADLARLAELLETEGEETEAVTLAVIAGTAGVGKTSLAVHWAHRIRDHFPDGQLYVNLRG
YDPVPPVGPDQVLDRFLRALGVPPARIPADTDDKAALYRSRLAGRRLLVLLDNAATAQQV
RPLLPGTPDCLTVVTSRNRMSGLVARDGARRVTGVLDEAEAVELLRRTTKEYRRGDDPA
ELTELARLCAYLPLALRIAAERAASRPWMPLRELIGDLRDESALWDALTAEEGEEADAVR
TVFAWSYRALSPDAARLFRLLGLHPGP
>B5GT95/327-582
GIIALQAVSGMPGVGKTLLACHAARRLEPFFPDGQIHLHLRAHVPGQPPLTAEEALTALL
RVLGVPPADIPDDRDALVGLWRTLLSSRRAVIVLDDVASAEQLDPLLPGPSPSLVIVTSR
RRITGIPGIRSIRLGVLPQQDAIALFRSVAGEDRTPHTGEVLELVRLAGCLPLALEIAAG
RLASRPTWTTTYFLHKLTNSESKLKEFRDGDRAVALTFDVSYRDLSTQEKEFFRFLGLRF
GIDVDVHVAAALAGLP
>D5SKK3/111-372
TTLEHLADSAETGGPSVAVVAGAPGLGKTAFAVHAAHRLAPRFPDGQYCLDLRAMDTEPV
RPDEALLRLLAAFGVAGQAVPRSLEDRAGLLRSLTATRRLLLVLDNAAHENQIRPLLPGS
GTSLTIITSRKSLTGLEAVHRVDLPLLRREEAVALLTRIVGPERVAREAQAARDLADRCG
RLPLALRIAGQRLAARPHETLGKLAALLSHEECRLDLLQTGDLRVRAAFTLSYQQLDDAS
RLLLRRCALAAGPDVSPETAAL
>E2Q2P9/18-224
ELTLLDRALARHRLITLVGCGGVGKSRLALRAARRVREAFRDGVAWAELAPLQGARLLVA
TVSDACDLSDHTPRTPVDALCEWLADKQILLVLDSCEHLLAPCRDLVGDLLTVVPGLTVL
STGREPLDLHLERVIEVEPLPEDGPEARELLRQRMASARPEPGPYDPVTAARICRRLEGI
PLALELAAGQAARTGTAAVAERLASRF
>D5SJ65/117-389

RAALVRELSEHLTAAGTAINTVTITGTGGIGKTTLAVQVAHRVREHFPGGQLYTDLQGAG
NQPVAAAAALGSFLRALGVRDDAIPDSLEDRTALYRSLLAGRRTLVLLDNARDAAQVRPL
LPGTANCAVLVTSRARMTGLEGARPVDLEVLTPGEALTLFTRITGRDRVAAEPEAARATA
AACGFLPLAIRIAASRLAARRTWTVSVLAAKLADEHRRIDELATGDLAVHATFTVGYSQL
EPETARAFRLLSLPDGPDISLDAASALLALQPP
>E2PXA3/506-764
MRDRLGGGMSVAAVLPQPQTLYGLGGVGKTQVAIEYVHRFMADYDLVWWISAEQPDDVIA
GLAELAVRLGAQGGEDMAAASQEAIDLLRRGAPHARWLLVFDNADDPEQLKRFFPPQGPG
HILVTSRNQTWSQYGDALAIDVFTREESVEHLQRRARGLTTEDADQVATAVGDLPLAVEQ
AAAWIAETATPVAEYLDRLREQATSVLALNQPAGYPEPVAATWNVSIEKLKERSPAAVRL
LQLCAFFAPEPISANLLYS
>E2Q9B8/317-568
RVLRGRNGQPPVRVLAVSGRAGAGKSALALRLAHRMAESFPDGRALVRLRGPHGRPLTTA
QVGAAVLRRLSGDDSGDRGTTPTDPAELAERIRQALHGRRILLVLDDVGSEEQIRPLLPP
TEGSAVILTGRRTPAALDGAEHLVLDAVRPDEAVELLMTAAGERIADDTAAAAEIARLCG
YLPLALRVAAAALTARPHTTADAFAERLRDERTRLGVLREGDLDLRSTLLTGYQEAGPAQ
RRAFRMLTLAPA
>B5GTA3/7-235
CTAARVVELISDLCVHLAGNRPSFTDPEQAGFHLARLLQGRRALLVVDNVWSAADLSPFL
LGGENCVRLVTTRNVRVCPSAARVVQLGPMAPGEVRELLIRTVGTLDETESARLADVCGG
WPLLASIVGANVSQEVVSGVPAGRVVAETSATIRAYGPQAFDVWDSDQRKNAMGQALSSS
LRSLEESVSIAGHSDLRDRYLSLAVFPPSVPIPMSVLTHWWQTAHGWAP
>E2PUK5/665-901
GAAPRLVVVTGIGGVGKTTLAVQVAHALGEEFPDGRLHADLGAGSSPVDPGGVLADFLGA
LGTPAARIPFDLGQRAALFRTVLADRRVLLVLDNARDAEQIRPLLPGTASAAVVVTTRAR
QLTVPGAHRIDLEVPSGDESLELLGAIAGPGRVAGAPETARALVERCGRLPLAVRIVGSR
LAAHPGRPLDRLAERLGDGPALLDELRSGELAVEPVFRLGYEALTPGDARAFRALAL
>D5SK96/320-549
TAELDQVRERLAPSPGHRPTAFGPRRLLVSGMPGVGKTALAVQAAHTMAEEFPDGLLHLR
LRAADGTPADCGDVLTGLLRQLGESPERLRAPDGDRLRLDDLIHRCRTLTAGRKLLVLLD
DAVNELQLEPLLPATADAAVLVTSRNQFSMGPGSWTVTLEPLTTEESFGLLAAVIGDARV
RAEPEAAREVVEACARLPLALRAAATRLAVRPHWPVARLARRLADPAARL
>E2PUY0/353-630
RAQELEELTMLAAGARGGSGVVMIVGPAGSGKTALALRWCRTAREAFPDGQLFLDLRGHS
DERPMGAREALSGLLRGLGIPYQDIPFNPMQMAELYQRTLASRRVLIVLDNAESADQVRQ
LLPYEAGCLALVTSRNRLGALIVSHGAAMLPLRPLASDEAAELVRSVVGRARTEAEPEAL
ADLVTICGRSPLALRIAAANLALRRDFRIADHVRDLRSGNPLESLAVAGDPDSAVRRAFE
LSYCRLDPAAAEGFRLLGLLPGPATTVAAVAALWGRAV
>E2Q0K0/282-547
RRAELEQLREIVLGLTRETRVAAVGGMPGVGKTFLAVRTAELVMRHFPDGVLYVDLHGYS
PGEPEPHARILVRILNDLGVSPATPTDDGMASAYRNALANRAVLLVLDNARDEDHVRPLL
PAPGASAAIITSRRKLHGLGVRESAGLVDLLPLDDGESAELLRIRLGEDRTRTALPFLPD
LVDHCGGLPLALCVVAARIAHHPARDVAGIVRELRQENTRLRSLDLASQSESVRLSLELS
HRQLPAPAARLLWQLAVHPGPTFSWQ
>Q0SBB0/335-468
RRNEVAAVKARLSDSRIVTLTGFGGVGKTRLACRVASEVRRTYTGGVWFVDLAAISTPDL
VVSAITEALDIRDGMNADGSHYLFEFLGGRHALIVLDNCEHLIEASGSVAAEVVRRSDRV
EILATSREPLGVLG
>Q0RVV8/356-614

```
EGKTLLSASRLVTLTGIGGVGKTRLALRVADKVQRTFREGVWLVELGDLRDGQLLSQVVA
TALNVRHEGADPLQILVDYLATRELLLVLDNCEQVIDAAAEMSIALLRSCPGLRILATSR
EALGIGGEAVLRVPPLTVPDPDQNPPVPGSPNDEALALFAERAVGAVPGFKFTEETRVTA
AQICYRLDGLPLAIELAAARLRAMTPDQILQRLADRYALLTRGSRGAPTRQQTLQWSIDW
SHDLCTPAEQQLWARLAVF
>Q0S5J6/141-413
EELHRIRVLLGDSRLVTVTGPGGIGKTRLAAEAATAYRRAFADGIRFVELASLRSETLLP
QTILDVLGFDERDAPERSATESLLESLRDKHLLLVLDNCEHLVSACAELVALILRNTEHV
KILVTSREVLSIPDEHVHVLEPLSTTDHRDPDMPGAAIELFENRASAALSGFSITDSSRD
AVRRVCTQLDGMPLAIELACARLTALSVDDLAERLDDRLALLTTGNRGGPSRHRSLHATV
EWSYDLCTTQEQVLWARLSIFSEGFDLAMAESI
>Q0RVI1/339-613
RTELAEAKNLLSASRLVTLTGIGGVGKTRLALQVAAKTQRDFPDGVWLVELGDLRDGSLL
EGMAATALGLRDRSARPAHDLLIEFIAPRQLLLVLDNCEQVVNAVAKLSESLLRSCPQLR
ILATSRESLGIGAEAVLLVPPLAIPDPDHLPRTMPHNDAVALFAERGAAAVPGFELTEDN
KATIARICRRLDGLPLPIELAAARLRGLTPEQILERLTDRYALLTRGSRDAPSRQQTLRM
CIDWSHNLCTPVEQRVWAQLSVFAGSCELGAAERI
>Q0RZ07/24-158
RELAETKRLLSVTRLVTITGAGGIGKTRLACRVASEVLRTFDDGVWLVDLSEIPDGSSLV
AAVSDIVGKPAEYNEHTAPKLAEHLAPKTALLILDCCDQFIASTAALCAFLLRSCPGLRI
LTTTRQALGIGGEAV
>Q0S5S5/336-605
RRHEITEAKNLLASSRLLTLVGIGGVGKTRLALRLATSIQREFTDGAWLVELDEVRTESR
LIDVVASTLGVRDQTDRSLREIILDVLSSRELLLVLDNCEQVVDAVADLATALLQCNPNL
RILATSREPLAIAGEATLRVPPLTVPDPDREPSLQGLPRYDAVSLFTERATTAVSTFTLT
DANKSAVTRICHRLDGLPLPIELAAARLRAMSPEQILQRLSDRYALLTRGSRGAPSRQQT
LRLCVDWSYDLGTPLEQLTWARLSVFPGSF
>Q0SCB4/289-497
RHELTEAKNLLTGSRLVTLTGIGGVGKTRLAIRIASAVQREYSDGVRLVELGELRDAVSL
VDSIAGALGVRDHSTRPLRDVLIEFLAPREVLLVLDNCEHMVDAVAELAGPLLRVSPRLR
ILATSREPLGIGGEAVLRVPPLALPDPERKPSLRGLPKYDAVTLFTERAAAVPGFVLTEE
NAATVAGICHRLDGLPLPIELAAARLRAM
>Q0SD65/355-582
TEAKQLLSAARLVTLTGIGGVGKTRLALRLAADVRPGFGDGVWLVELGELRDGSLLTDTV
AAALGLRDQPARQLDDVVAEFLASRHLLLVLDNCEQVVEAVAASAEMLLRTCPQLRILAT
SREPLGIGGEAVLRVPPLTVPAPDREPSLRGLTGYDAVTLFAQRAAAAVPGFELTDDNRM
TVVRICHQLDGLPLPIELAAARLRAMSPEQILQRLTDRYTLLTLGNRG
>Q0RWB3/343-617
REETAKVTSLLSTTRMVTLTGMGGVGKTRLALRVAGRTQSRFADGVFLIELGELRDDSLL
VAMVADALGLRDRAARPILEVLTEFLAPREVLLVLDNCEQVLDVVAKLSEALLRTSPGLR
MLLTSREPIGISGEIALPIAPLAVPDADHLPRRLPGNDAVALFAERGAAVLPGFEVIDGN
KVTIARICQRLDGLPLAIELAAARLRAISPDEILQRLTDRYRFLTRGRRDAPSRQQTLRM
CIDWSYDLCTPLEQLMWARLSMFAGSFELNAAEQI
>Q0SIL3/24-294
HEIAEIRRLLSVSRLVTLTGVGGVGKTRLALRVAADSSRAFDDGVWSVGLGELYDPGAVV
DTVLSALNLREGPGAPETQLVEYLTPRKLLLVLDNCEHLVGPVANLAETLLRSCPDVRIL
ATSREPFGIGGEAVLRVPPLTLPEARQRSVAGGGLGHYEAMTLFTERAATAVPGFEITES
NEGVVAAICRRLDGLPLAIELAAVRLRVMSVEQILHRLTDRFTLLTTGSRGAPSRQQTLL
GSIDWSYDLCTWAEREMWGRLTVFAGGFELD
```

>Q0SKG2/39-304
TEARRLLSVSRLVTLAGIGGVGKTRLALRVAADASRAFEDGVWLVELGELDDDTTLVDAV
SAALRLREQRSGNPEVLLTEYLATRQLLLVLDNCEHVVAAAAALSETLLRGCPELRILAT
SREPLAIGGEAVLRVPPLTVPEPERSSLQGLPQYEAVTLFVERAATAVPEFELTEDNHVA
VARICRQLDGLPLAIELAAVRLRVMSAEQILQRLTDRYRLLTVGSRGAPSRQQTLRLCVD
WSHELCTEEERELWARLSVFAGGFEL
>Q0RVY5/339-616
RSELSEAKNLLGSSRLVTLTGIGGVGKTRLALKVAASAQRGFADGAWLVELDETVDHSLV
IEKVAAIFGLRDQAARRTEDVLVEFLSSREVLLLLDNCEQAVGAVAGLSATLLRACPRIR
ILATSREALGVGGEAVLRVPPLTVPDPNPPAIRGLSRYDAVALFVERAVTMVPTFTLNED
NYTAVARICHRLDGLPLPIELAAARLRVLSPEQILQRLSDRFALLTRGSRSAPSRQQTLR
LCIDWSYDLCNAAEQLVWSRLSVFVESIDLDAAEQVCG
>D2AQB1/264-516
EEVLGWIVRQAPAAGGAPVHLVLHGPAGSGKSAVAVHAATLLGAEFPDGRLYAALRAGNR
PAGAGAVLEDLLRSLGCPEGAVPTGLDDRVRLYRSMVASRRLLVVLDDAVGESQVRPLLP
TGPGCLTLVTSRSPLYGLEASRAFELGVLDTAGSVAMLARVAGEPRVRAEPQAARRIAEL
CGGLPLALRIAGSRLARRPGWTLEHLAVRLGDERGRLDELTAGDLAVRSSLGLGYRGLAE
EEQRLLRRLGALS
>D2B752/122-371
RDGDNSPLVVSVYGRGGVGKSMLIARFGHEVADRFPDGRLYADLRGAVEAPIQAEEVLIG
FLRALGVRLTTDPGGPAELRKLWLTWTKGKRILIGLDNAQDGDQVKDLIPAEPGCAVMIT
SRQPLFLLNTYDKQLSVFSEAQGVELLARLAGDDRVAADLESAQEIVRMCDHLPLAISIC
GGRLATRENWTLRELADRLRDERRRLDHLELARRLDKSVRASLQLSYDDCTGLQRRLLRL
LSLLTAPDMP
>D2BDC2/135-375
REEILAQLDSAMGGDEPVVVAVVQGLGGIGKSTLAARYAALHHDRFHPVWWITADSPAAL
EAGLAALITALDPRDAEGVDLKARTERATVWLATHSGWLLVLDDVTRPQDIAPLLGRVRS
GRVVVTSRLRQGWQRIGARVLHLDVLSEDEAVDLLTRLAQPEDPGHDVRPGALDLVQELG
FLPLAIDQVGAYLHQTALTPAAYLTLLRAQPEVFFDQAAEGADSDRTVARIWRITLDQLT
G
>D2AS59/291-547
RYAVPDSSTMAVVTAVTGAAGVGKTALALRFGHRTADEFPDGQLYIDLRGHSLRPPMAPL
EALTRMLGSLGVPAEQIPGDEERAAGLYRSHLSGRRILVLLDNAHTADQVRPLLPGAPGC
LTLVTSRDALAGLAASHGARRLSLGMLGHAESLRLLESVIGAERLAAERRTAEEIVRLCA
HLPLALRVAAATLATHPHWSLAGYGTALAAGRLDMLQIDGDMAVRAAFSLSYARLPPPAR
RLFRLLGLVPGPDVTAP
>D2AZV1/261-526
REAELQRLTSMVAENADRTALIVTIDGMAGIGKTAFAVHAAHKLARLFPDGQLFVDFNGF
TPGRKPIPVAEAIATLLSTLGVPDGEIPHDLQGRIAMWRMRTAGRRLLLLLDNTADAAQV
LPLLPGMPGCVTLITSRAPLSGVDGAVLQSLELLTPDESRALLERVVGTMRLATERETVT
TLIEMCGRLPLALRIVAARLNNRPQWSVAHMVDRLGNERRRLSELVVGDRSVHAAIAHSY
GSLRPDQQRLFRLLGLHPGHDYDAYA
>D2B9Q5/21-150
RETEIRELLKLLAETSLVTVTGGGGVGKSRIAVKAAEECRDSYPDGTWLVELSGERNGEL
LASIVAAVLGVREHSARPRIETLAEFLADKHLLVLLDACERLLDACRALVAAILERAPGV
RVIVTSRRAL
>D2B6L3/20-230
RKEEVRQVRRLLQGSRLVTVTGGAGIGKSRVAVEAASGVRRAFSDGVRFVELAGVSDPGH
LEEAVAQALDLADPSDRPDVEALADHLRDRQTLLLLDTCEHLVDACARLAQTLLESSPEL

RILATSRQSLGVPGEHIVALAPMPLPGPRAVESVAVLARCDSVALFLERVTAVDPDYALT
AENAAQVAEICARLDGIPLAIELVAVRMRTL
>D2AUE3/25-299
REGDVEELVELLGVARLVTLCGAGGIGKSRLAVRVASQVAAGFPGGVWLVELAEAVRGDL
VEPRVAAVLGVKAEPPRPLSDTLIDALGDRNLLMIIDNCESLIEESARFCRAVLTVCPAV
RMLTTSREPLRVAGETVWRVPPLSLPRTGVHELAGSEAVRLFVDRAGAASRGFAVTEQNA
GEIASLCEALDGMPLAIELAAALCRVLTVEQIHARIRDRFRLLSAGDRMAPARHQTLRAT
VDWSYQQLNEPERILLRRLAVFTGGWTLDMAEQVC
>D2BEW7/285-563
TGAVADLARALSAKGRPSDEPPSIAVVVGPPGVGKSALAVHCANAVRADYPAGQLYLGLG
GTAAAPADLGELLAEALRALGAGEADLPPTVHERSALYRSLLAERPMLVLLDDAADAAQV
RALLPGNGCAVLVTSRRRITELPSSLRLDLGVMSPPEAEEFLGKIVGAERLSEEREDASA
ILRSCGYLPLAVRIAGARLAGRPGWPLSVLRQRLDDESNRLDELRAGDLEVRDSFDRSYR
QLPDEVARTYRTLGLLGPQSMPGWVVDAVLDRTRAETVM
>D2BEW1/287-570
RVEKLRRLDMLLSEEEGTATVVISAIAGTAGVGKTALATHWGHRVAARFPDGQLYVNLHG
YSRGRATTGAQALDRLLRGLGVVDDEIPHDVDERAGLYRSLLAHRRMLIVLDNAATPEQV
RPLLPGSSPSRVVITSRDALRGLSVTHDVRGIVLDVLPADEATALLNKLLGRNGTDDETD
PVPELARLCGYLPLALRLAAAQLAGEPASRIGDFIAKLRQENRLTVLELREDPGTGVRSA
LELSYRSLPEPARRTLRLLSVHPGPDIDLQAVAALTAMSAEDAS
>D2B1E3/287-555
RDKETAALLDHLSGLLNDPTRPAAMVAGVTGPAGAGKSTLAVHVAHRLMSAYTDGQLYAD
LRGTGAVPESPVRVLGRFLRALGVGKGAVPDDPAERTALYRSLMNGRRMLVVLDDALGEA
QVRPLLPGSSACSVLVTSRSRLVGVSGARIVDLDRFDVDHAIHLLASIIGHERVTAEPDA
AAELVRLCGGLPLAVRICGARLAARPYWQLAGLAAVLRDERRRLDELSVADLAVRDGLRQ
SHSRIGEDARQTLARLARLGPVEFAIGTV
>D2BF08/277-533
RDKLVEQLSGLIADRRPGPIPVSTITGRAGVGKSTLAVHLAHRMIGDFPGGQLYADLRGS
AEQPADPSRVLTRFLRSLGISGQAIPEDADERAELYRTQLAGRRVLVVLDDAADQAQVRP
LLPGSPSCSVIVTSRSRMAGWPGAHAVDLDLLEPHHAGDLLAVIVGAERVAPEPEAATEL
VRLCGRLPLAIRGAATRLAARPHWTLARMAGRMADERHGLDELSDVRATLALGYRRLDGP
AQRALRLLGLLDLPTFA
>D2BDM2/157-408
REAQLAAVLAAVDSSRVVAVDGMAGVGKTCLALHAAHRLAADYPDAQLYVDLHGFTDGRE
PLGPEPALRALLAALDVPSEKIPQEGGIEPLAACWRSELAGRRAVVVLDNAAGADQVRPL
LPGAGHSVALITSRNRLLGLDEVPPVSLDVLTPEESAELLARASGDPGGSDGRLARDPES
AAEVLRLCGHLPLALRLAGARLRHRPGWTVGILVERMAEGAGEFDTALAMSVRQLDRAER
RLFRLLGLLPGS
>D2AY68/285-497
RTEPLAQIRRILAADQDNRTTARAVSICGMAGAGKTTLALHAAHINRAQYPDGQLFADLR
GASATPTPQTDVLASFLRAVGVPDHQIPPSLEERSNLFRTWSNGRRVLVILDDACAASQV
ASLLPATPQCTVIITSREGLQSLPGVQTVELGVMNLTEGVELLGRIIGAGRVAAEREQAE
KIVDLCGHLPLALRSVGARLAAARTWPLQKMAA
>D2ASK6/318-568
LTRALSPVERPPDGPPSIVVVVGPPGVGKSALAVHCAHAVRTGYPGGQLYLDLGGTEYAP
ADPGELLAEALRALGVGEAGLPCTVRERSALYRSLLAERPMLVLLDDAAGAAQVRPLLPG
NGCAVLVTSRRRITELPGALQLELDVLSPEEAEELLGRIVGSERLGREREAASAILRACG
YLPLAVRVAGARLAGRPRWSLGVLRQRLEDEAGRLGELRAGDLEVRGSFDRSYRLLPDDA
ALAFRALGLLG

>D2B7R9/259-461
VAEVRALLETGRLVTLTGSGGVGKTSLALEVARQVAGTHPDGVWLVELAPYDRHTPSLAE
AVLVALDIREDSAGVLPRLVGALRDRRMLLVLDNCEHVVDQAAELTEALLRSAPGLRILA
TSREPLNVAGEALWSVPALELPEGSELLTVAKADAVRMFMARATASARGFTLDARNAEAV
AQLCRRLDGIPLALELAATRVRA
>D2ATJ1/282-548
RTKQIDDIRQRLTLAVDDRSRFAVPIIAIVGKAGIGKTTVAVHSAHSVAEHFPDGQLYAD
LHGGVSRPTSPMQVLERFLRVLGVPGTALPDGLEERAEMYRSLLADRRMLIVLDDAGNES
QVLPLLPGNPASAVIITSRSRLAGLAGAIHVDVDVFDSSQSMDLLSRIAGVERVQSEAES
AAALAELCGQLPLALRIAGARLLARPHWSIEQLVGRLEDETRRLDELKHGDMGIRASISL
TYDGTGDDARRLFRRLAILDSQIFSAW
>D2B796/33-297
RDDLLEQLRKGVTAEVTAVVPHALHGLGGVGKTQVAIEYAYRYRSAYDLVWWVPADQPML
VRSALAGLAPYLGLPSAATTGIEDAATAVLDALRRGEPYDKWLLIFDNADQPEDLNEIVP
RGPGHVLITSRNHRWQGVVDTVAIDVFGREESIEFLSKRVSKAVSREEADRLAEELGDLP
LALEQAGALQAETGMSVDEYLRLLHEQTALLLAESKPSDYPVSMTAAWSLSVSQLVSKMP
EAVELLRCCAFFGPEPIPRDVFAPL
>D2BCU2/310-576
RRQIIARLCTLLSTQGSGDGVPVAAISGIGGVGKTTLAVHVAHALHDLFPDGQLYADLRG
YGEEPVAPESVLAAFLRALGLPADIIPDGLAERSALFRSLLTDRRMLVLLDNARDAAQVS
HLLPGSTGCAAIVTSRGKLADLAAARLVDLDVMEPEEALTLFGTVAGAERVAAERAAAMD
VVAACGFLPLAVRIVAARLAARASWTVASLVPRLADERRRLDEMRVGNLAVEATFALGYG
QLSPAQARAFRLLSLPGGPDISAGAAS
>D9T401/279-542
REEMVDRLLCEVREASGQGPTIRVIDGMAGCGKTTLALHVASRSAERFPDGQLFVDLRGH
STRTRMRPAVALDTLLHQLGVPTGRIPLELPDRQEMWRRELAARSVLVVLDNAADSRQVE
PLLPVSGSAVVLVTSRRRLLALTERPPVSLAVLAEAEALELLATLIGSGRVAAEPEAARQ
LVAICGCLPLAVRLAGTRLAHRPDWRIADLVDRLSHRSIFLPGLRAEARSVVEAFAESYE
PLDEACRKAFRSFGLAAGNHLDTA
>D9TA73/304-498
GGGSPVVISSIGGGGGVGKTWLALRWAHANAGRFPDGQLYVNLRGFDPAADPVAWPAAVR
GFLEALGVEPARVPADPDARASLYRTLVAGRRLLVVLDDARDTATVIPLLPGTPTSAVLV
TSRRQLAGLVTTHGAHPLPLDVLPDDEAHELIVRQVGAARVAGHPDAVADILRFCGGLPL
ALGIVAARAALHPEL
>D9T1P0/481-735
ADLRELRTLLRSSPKVVLSGTGPVALQGMGGIGKSQLALEYAHRYRAAYDMVWWIDADQV
PFIESAIGDLAPYLGVPSSDSNRENARLVLQALRRTDLRWLLIMDNADEVEGVLPYVPDG
KGHVLITSRNLQWVERATTVQVDVFKRAESIQHLTERVPTMRVDQADRIAALLGDLPIAV
TAAAAWLADTGHSVDSYLNEIARFGPGAVMEPNSNVSVEATWELSLNHLRTRNPAAYRVL
QLCSVFAPEISADLV
>D9T090/92-293
RQLDRLLDGGPRRMSSVVVCVLSGRSGIGKTALALRWAHRSAARFADGVLHVQLGGSDPG
RAAVAPAEALGGLIEALDVPGGVPCGPAARTGLYRSALAGRRMLIVLDDARDADQVRPLL
PGVPGCMVVVTSRDRLTGLVVGEAACPVVVDELAAGEAWDLLAARLGERRLTAERAAVRE
IIAHCAGVPRALAVVAARAAVQ
>D9T212/297-482
VATGRAPAVVVVTGPPGSGKTGLAVHAAHAVAGEFTDGQIFVDVAYRASVTPAELLARVL
RALGVAAGDMPDSADERAGRLRSLTAGRRLLLVLDGVTSAAQVRPLLPASPGPALIAVAR
RALGNLDGVRRVALSPLAEPGARELLAALAGPERLAADPGATAELIGLCAGSVLALRVAG

SRLATW
>D9T820/297-580
RAIAEGIADPAGADEHAGVVLVSGAPGVGKSSFSVSSGYDLADLFPDGQLFVSFDSGDEP
PRGSGELIRELLVELGVPLTDVSEDIRERSAVLRCALAGRRLLLIIDDVDCAQQVRPLLP
GAGRSLVLINSRQRLLDLDVGWRLTLGALDRADAVELLATIAGERRVREQPEVFGRIAAA
CDQLPLALRIVGSRLVTQPDVALPGFAAHLEDEENRLSELAVGDISVRGSLSVSYQALDV
DARLALHALARADSFITPASAVEILRLPRQQANRMVEHLIQHNL
>D9T1Q1/253-525
RQAELSALSEALLADAPVNTGLAVVTGMAGVGKTQLAQHWAASVEGDFPDGTLYVDLNGY
TANGSPEQPAQLLARILNDLGVRPRTPTVDGMSTEYRTELARRKTIVLLDNARDVHQVRP
LLPGTGSSVAIVTSRDRLMQMIVREHAHEVRLGPLGHEDAVALLASKLGAARMRAGAEHV
SEIVALCGGLPLALSIVAAQARSRPPEALEEITAALREEGTRLDSLGHRSAELNVRAALS
SSYGTLSAPAAELFARLAVHPGPTISRRAVGCL
>D9T0E9/271-536
RQQTVARLVKEVAEEGSRVQLLDGMAGSGKTTLAVQVASALVDRFPDAQLFVDLHGHSDR
SPLSTAAAAAILLQQLGVPAERVPADLAGRLAMWRSELADRRAVVVLDNAADAAQVVPLL
PNGRDCTVLITSRRRLVGVDAGRPTSLPVLDADEAVELLARVAGAERVDAEPEAAAEVAR
RCGHLPLALRLAGARLAHRPRWRMADLAERLAGAADPLAELTVGERSVARAFALSYEQVP
PVVQRVFRLLGLHPGSRIDNRVAAVL
>D9T1H6/274-540
RTDEMTRLDQLSGGSSATTVWVLSGIPGVGKTALAVRWAHRVRDAFPDGQMFIDLRGFDV
ERPPVTPSAALGQLLGGLGVDPRVVPDDQDGRVSLFRSTMTGRRILLVLDNARDADQVIP
LIPPSGTVLITSRRRLGELVVRAGARSLLLGVLSPTDSVRLLAAMLGDEAVRVEAGAAAR
LAHLCGHLPLALRIAAANLQTSGHPHIAGLAEELDEGGPLTSLTVDGADEGAVTKAFAMS
YRALPTAQQRMFRHIGLVPGGSFTPHV
>D9T288/326-584
VGSRTATRIGFITGMPGVGKTALATHLAHRLRRSYPGGQLYADLNGSSGRALDPAEVLHG
FLRTLGLAEEHIPDDLTERCKTYRSATAGRRLLVLLDDAASVDQVRLLLPSDPRCAVVVT
GRRRLYGLGGLWNIDLDVFDHAESLELLARIIGRDRVDREQRAARTLVDMVGRLPLALRC
IGARLAAVPARSLSGMAEHLQRSREVLDELCLGELDVRSAYDASYDLLNRVEQSTFRLLS
MLPHGEFTAEAVADLLGWE
>D3PWT8/278-533
RDDVVRELTALLTEERDAAPVVVVTGIGGAGKTTLATHVGHRVAADFPDGQLYVDLRGAD
EVPHEPLAAQRGMLRSLGVSTEDIPAAEDECAALFRSTMASKRLLLLLDNAADTAQVRAL
LPGAAGCAAIVTARSTLTGLTGARYVRLSALEPGEAVTLLRRVVGTERVDAESAEALNVV
TACGSLPLAVRIAAARLVARPQWTVGQFAERVRDEQRRLAELRAGDLAVEAAFALSYRQL
DEQHAHAFRLLTVPDA
>D3Q9F1/277-534
ESPLRTLVDAADDSRVIVVDGMAGIGKTTLAVHAARRLAERYPDGQLYLNLHSFTDSVPP
MAPAEALSALLDSLGVPRNAIPESVDARAAKFRSMLAGRRVLLLLDNARDEAQLSPLLPG
DSGCLTIITSRRRLSGLDDIRPISLEPLDLEPSARLFAAAAGIDDLNDDDRAAIDRVVEL
CGGLPLAIRIAAARLRSRPTWSAADLLERLSKDYRLLDELAAGSRSVASTLGLSYRELTD
GQRRLFRLLALCPGSDFD
>D3Q803/276-548
RQEQLARLDALIAKGDNTALLSTVSGTGGAGKTALAIHWAHHNRDRFPDGQLYVNLRAFD
RAEPLTPYDALTRFLAALGVTGGAVPSDVEAAASLYRSLLDGRRMLVLLDNAVDLEQVRP
LLPGSGGNVTLVTSRNRITGLTALHGAELIGVDTMSRTESLEVLGNLVGARRLHADAAAA
HRLAELCADLPLALRIAGANLAVNSHVELSEYVRELAGPNRLELLSIEGDPDSAVASVFA
QSFRALSPEAQRLFARLGWIPGDDFGEELAIAV

>D3Q6R6/279-482
RAAELSELDNALDAPDGARLWTVSGPGGIGKSWLALHWSHRRRDAFADGQLYVNLHGFDP
GTAPTPPETALRGLLETLGVAPAAIPTSLDAMSGLYRSLLVGKRVLILVDDARDSRQVIP
LLPGDDAAFTVVTGRPGLATLATSHGAAGLRLDTLSHNESREAFSRHLGHDRIAAEPEAT
AELVGSCAGLPLALGILAARAAAH
>D3Q6D8/281-542
ELDLLLAASEATPQPAVGVITGTAGVGKTSLAIHWARRVADDFPDGQLFVNMRGYHPEQA
LTPQRVLARFLRTLGVRGQDLPEDIDELTAMYRSVMDGRRMLIVLDNVNHSDQARPLLPG
AAGCLVVITSRDVLLSLIAADNASQISLDLPDTADARRMLARRLGSERLSSEPGAADDII
AASARLPLALAIAAARAVINKDTRLSAVAEQLRAGLDAFDTASPMTDVRAVFSWSYQALS
PDAARLLRLLGLHPGSDVSAAA
>D3Q9E7/109-372
RAEELATLDDALPGLGGGATVVVLTGTAGVGKTALAVRWAHRVAGRFDDGQLYVNLRGFG
PGPPVRTIEVVAGFLAALGVPAARIPFKEEQATALYRSLMSGRRMLVVLDNAADADQVRP
LMPSGRDSLVLVTSRHLLTGLIVSHGAQQLTVAPLSATDASDLVTAIVGASRTAAKPEAV
TALADSCARLPLALRIAAANVRADRYRDIGDYVDELRHGDRLASLSVDGDPTASVRGTFD
WSYARLSAPVQRLFRQLGMVPGPG
>D3Q478/280-559
RDSEVEALCSAVTQRRSGALVVSSVEGMAGIGKTALAVHAARRLADEFPDGQLFIDLHGF
DADAPATDPAVALERLLHSLGVDPRQIPENLDERAGLYRSILSDRSVLIVLDNASGEQQV
GPLLPGGDASLAIVTSRRSLAGLDQARSIQLELLGRAEAIALLAAALGPSRGGAGQKSVP
AVEPGRFAAADEAVAAEIVAVCGMLPLAIRIAAARLRHRPDWTLADLRDRLQQAGLAGLC
AGDRSVTAAFEMSYAELDDLARNAFRRLGLHPGHDFDVYT
>D3Q140/286-549
ALTALTRQFHDSDKSRSCVVVGAAGVGKTALAIHWAHENRDQFPDGQFFINLRGFDSGAP
VSAHEALGRFIRALRDPSVSIPSDVDEAAALYRSLTDGKRILVVLDNAKNADQIRPLIPS
SPNAFTVATSRNRLTGLTAVDDTVPLFLSPLNHLESVDLLAKSASTAGLSIDRGSTRRIA
ELCGHLPLALRISAALLVDGSGRTAKQLADELGNPDRLDLLSVDGDSTIATALDQSFQSL
TAEAQQLLCQLALIPGDDFPQALA
>D3Q578/289-556
RQLSRGCLDAEAAGSVVVCALDGMPGIGKTTLAVHAARLLAPDFPDGQLFLDLHGFSGAG
ERVEPGDALDRMLRALGLAVEDIPAEVEDRAALYRSLLSDRRLLIVLDNAADEAQLRPLL
PGGSRCLVIVTSRRRMSALDEVTPIPLDVMSADEATALFLDAAATTGLDDTAAGIVAEVV
ETCGRLPLAVRIAAARLRSRPNWTLADLRDRLARDGELLSKLEFGQRSVRVAFEMSYREL
DERHAHLFRLLGLAPGSDLSVGAVCALL
>D3Q0C3/269-515
REAEVSELTELLVTDSMTPLVISSINGGGGVGKSALAIHLGHLVRERFPDGQLYVNLQGA
TPNAEPLEPAEVLRRFLRSLGVGGDDGISEVAELANRLRTATNGKRMLFLLDDARDSGQV
RPLLPSEPRCAVLITSRRTLSTLDGSVNRALDGLSDADAVTLLERLVGTDRVAAEPEAVR
RLLGWCGGMPLALRICAARLVARPRWSVASLADELADETRRLSELEVDDLAIRSSFAVGY
RQLCEDS
>D3PUP6/139-400
RDGELAALVAALTRDGGVPVCAVTGMGGVGKTALALHAAHAVAEQFPDGQVYLDLRSHDK
PLDTRAALSQLLRAVGGVVDAVDGTDAVGAYRSVLAGRRLLLVLDNVSGPQQVADLLPGA
TGCAAIVTSRHALDTLPHTLHTRLDVLTEDASLTLLTDTIGAERLAAEPEAARELAGYCG
GLPLALHLAGSRLAVRPQWPIAHLAERLADRVRRLDELERRELGVRACFAVSFELLDAAD
RRRYALLGVLPAGGLSVELTAR
>D3Q4L4/261-484
TRLNDVFAPSIDAGVRTAALVGLSGIGKSSQAAAYVANRADLYDLICWVDAESTSTLTAS

LRQVLAHIRGITRASIMEATPDELRDAVHTELSRYNGRWLIVFDNVTVARDLGAWLPAVG
RGDVLITALDAATRFGKAATIPVEQMSRDEAVTLLRLRLDMSDAEYSSHIGLLERIVNAM
GRWPLAIELATGYMHGCGIPIHQAHHYLDRLKQRALADDMAIPV
>D3PWL2/92-346
HDAVVTALADARLVSLMGPGGMGKTRLAAVVATAVAPAFPAGAAFVDLVPVRPGQVTVAV
AQVLGVTERPPQTLDHTIVGWLKQGRFLLVLDNCEHVVDDVAALAAMVQQRCPNTTILAT
TRRRLAVPGEQVVRLGPLPIEPDAVRLFFDRARAVDAELDIDPQTAAAICRNLDGMPLAV
EIAAARAASLGAEGLRAAAGDRLRLASGTRGVHPRHSSLSAVMSWSYDLLDTEAQAALRA
LSVFTGSFDLSAVAA
>D3PW43/293-571
AHLAALDRLRDSGVRTGIVTAITGIGGVGKTALAVHWGHARRENFPDGQLYINLRGFDER
KPLTPHEAISRLLRTLGQPANTIPSDLEEAAGLYRSLLADKRMLVVLDNARSPEQVGQLL
PEGSGSLALVTSRNRLASLATTHGAEHVNLDTLSPNESLDLFTNILGPRALEDIESTRRV
CALCGQLPLALRVVAANLIQYPNKSLAQLANELEGGSRLSQLSIEGDNTTNLTAVFNLSY
SALSDASQSVFQYLGVIPGDDFTSSLAAAITKTSETTVQ
>D3PX39/74-340
VDKMAELTALVTKPRKSGAALVAIGGLGGVGKSALVARWGNQVAGELFPDGTMHVNLGAH
RANGGVGMNAAFAQLLGQCGCEDWQLAKDLPGRREQLRARLRGKRVLLVADDVNTDAEVA
QLRPEYVGSALIITGHGGGEYAMDQEHLSVEPLDDASAFELVSILIGSGEAEAERAEVTE
LVRLCGGVPKVIGIAVGPVRRHDSIRIADVLAGLRTEIDGAEGPVDQDSILGMVADRAYH
SLTPEAARLYRALSWHPEGDVSKHAIL
>D3Q901/250-510
RQTHLDQLDRLLAGGDRTIVVSGTAGVGKTSLAVHWAHRVAERFGEGQLYLNLRGFDPEA
EPLSAADAILAFLDALGVPGPRVPPGIEAQTALYRSLVAERELLVLLDNARDAEQVRPLL
PGGDRCLTLVTSRNRLAGLVATEGARPVALGEMSELEARVLLRARLGSERVDAETEVVTA
LIQHCGGLPLALSIAAARAEVDPAASLAPLLRQLATAGGDFLTLDDADPKTSVRSVFSWS
YRCLSPEAARLFRRLGIHPGP
>D3PVN3/278-513
AERAQLRHALTPTRGQERPIVALHGSGGVGKSTLAIQVAHDLNPTFPDGQLYVDLQGSTP
GLPPLTPLEILRRLLSALGQPDGEIPTDATEAARRYTDLSDGSQHLILLDNATDPRQVEP
VIRASRSGGLLITGRAPLALSDVQLSLRLDVLPPADAITLLDRIAGRTGADWSDFSQIAA
YCDYLPLALCIAGGRLAREPDLSGKRLAASLSDHRDRLDTLEVDGVGVRSSIRVGY
>D3PY98/266-456
LETLDATGDPIAVVTGPGGIGKTWLVLRWAHDNLDRFPDGQLYLDLRGFDPHSAPLPPGV
AILGLLQGLGVKDDAIPSNLDAQIGLYRSLLADKRMLIVLDNARESSQLTPLLPGGSTCT
TLITSRHVLPALSASHDATRVRLDVMKPDDAHAALAAHLGANRLACEPAVAAELVDHCGG
LPLALGIIAAH
>D3PZV4/270-543
RRKQVVALDELLEQGRNTAVVSAIAGMGGAGKTALALYWGHRVRERFPDGQLYINLRGYD
EAKPVAAIDALGRFLVALGQTSTTVPSDVDEAAALFRSLLSERRMLVILDNAREAAQVRP
LLPGGAGNLAIVTSRDRLASLTALEGAEPIRLDTLSQTESLELLANIVGAGRLDTDPEAA
HRIAELCGRLPLALRIAGASLAAQPDLALGEFTDVLGGPDRLRRLALDGDKLASVSNVLE
LSVAALDDTSRELLLKLAQILGDDFCHGLAVHLS
>D3Q1R7/316-576
REAQLETLDAQLPSASLVTISGMAGVGKTALAVHWAQRIASRFPDGQLYVNLRGFDPSGQ
PTEPADVIRGFLDALAVPPHSIPVSPDAQIGLYRSLVADRKMLILLDNAGEEKQVRDLLP
GTPECLTIVTSRNRLTGLTASHGAVPMPLSEFTPEESRRFLRSRLGEGQLAAEPQAADTI
IATCAGLPLALAVVAARAATMPQVQLEQSAAELRGATGDLEPFVMSDVSTDIRAVFSWSY
RLLGAEAAHFFILLGHHPGPD

>D3Q9Z2/276-542
AQLDSLPKAGATSAILSTIGGIGGVGKTALAIHWAHRNRRRFPDGQLYVNLRGFDREEPL
APLKALTRFLRAFDVPADTIPSDTESAAALFRSLVIDKRLLVVLDNARDVEQVRPLVPGG
PETLTLVTSRNRLVGLTALHGAVPITVGAMSRTESLDVLNNLVGKDRLHAESSASRQLAR
LCADLPLALRIAGANLGTTSELSVAEYVQELEGPQRLERLSIEGEPQTAVSAALSLSVQA
LPVAAQQLFMRVGLIPGEDFHQDLVTV
>D3Q804/277-537
SQLRALDALGDDSVLATITGCGGSGKTALAVHWAHRNRDRFPDGQLYLNLRGFDADAPLS
PQDALTRLLPALGQPADAVPAELDAAAALFRSLLTGRRMLLLLDNARDAAQVEPLLPNEP
GTVTLVTSRHRLTELAAHGATAISLDTLDETDALALLSTLVPDDRLAEDPAATAELVSRC
GGLPLALRIVGANLAGRPYSTVAEFAAEHSGSDRLGLLTVDGDPNATVATVFERSARALD
EDTRRLFLRLGLIPGDEIPED
>D3PUN7/142-408
ERRRIAQLANESRDQATVIVIEGMAGIGKSELALRAAQDLAEAARGARVRLYVNLRGYDP
HEPPADPDAVVRGFLSHLGMPNFKIEALNAATRAARYRELLNARDAVVVLDNAFDAEQVR
HLLAPSAGTVFLVTTRRRLTDLDSAHRMQLDLLPVNDALTLLNRYDPADRVDSAPQSAAQ
LVELCRRLPLELVAVGRQLSGKPEWELSDHVERLKRIPPSEVSRPALAVSYASLRPDEQR
VFRQLSIHPGREFTMDTVVALTDLGRE
>D3PYM4/280-554
DTLINAADAAVARDHDTVAFVGPGGAGKTALALTWAHKLSARFTDGQLFADLRGFSGTEP
APPARVLTGFLRALGVPASRLPAGESELSALFRATVAGRRILIVLDNAVGPRQLRPLLPG
DDGCLTVATSRDNLSGLDRVHAITVAELSSADSQRVLAETLGARPGPVAATLIARLAEQC
GNLPLALRLAASQLSGGSDHELSELVDDLDSGDRLATLSYPEDSPGGVAAAIETSYKVLA
PGPRHLFRLLGLHPSGTADVEALAAMADADLAETE
>D3PZU2/272-529
EAEIEELTRADTPGRRGLVRAVDGMAGVGKSTLAIAAAHRLAPRYPDGQLFADLRGFTPG
VEPSQPGEVLGRLLEGLGVTGDRLPHDTEARAGLWRGMSRGKRILLLLDNAVDANQLRPL
LPADPGCLAIVTSRRRLTDLDDVRNLTLDVLPKPAAVQLFTNVVGASRLSDQEALDEVLA
SCGGLPLALRLTASRFRDRPSWTLSQLAERLRRDRPEVLSSDGVASAFGIAYERLSDERR
RLFRRLSLHPGVDIGSAA
>D3Q9Q8/274-529
AIALLGSGTPIVSVDGMAGVGKTAFAVRVATEVSDKFCDGQLFVDLRGFSDDLAPLPANE
AIGGMLRDLGVPQTQIPADLAGRSAMLRSRLADRRVLLVLDNTIGTEQVLPLLPGPGDSA
VLITSRRKLPDLPDAEPITLDVLPRHEARELFTTVAQRNIDAETDPVNDIVTLAGQLPLA
LRLAAARLRSRPAWTVTDLRDRMASERQGERRSPAGRKLGAAFELSLRALTVEKRETFLS
ASLIPVHDLTAASVAA
>D3QAE7/276-544
REEQLAALDNLLADGRTATVVSAIAGMGGAGKTALAVHWAHHVRDRFPDGQLYINLRGYD
EAAPVSPADALTRFLNALGQPGAAIPTDPDEAGAMYRSLLADQRMLILLDNARDAAQVRP
LLPGGGGNFALITSRDRLTSLVALDDVAPLRIDTLSHEESVDLLSNLVDPVRLHSEPEAT
HQLARLCGHLPLALRIAGANLADRPETNVTQFVAELEGPQRLQKLTAPDDPAVAITRTLH
LSVSALTPAARQLFTLLGILPGEDFSHDL
>D3PZJ5/289-544
SELAALKRDADRTSVLILDGMPGVGKTATAVRLATELAQRYPDGQLFLDLHGYSGDVPAV
EPAEALVRLLRGLGAEADQIPTGLDERSAELRTRLAGRRVLILLDNAATSAQVRPLLPGG
TDCLTIITSRRRLPDLLEAAPASLDVLEPEEAVRLLVAAVDDPKRVAEDSADTAAIVEVA
GRLPLAIRLIAARLRNRRNWTAGFMLGRLRDETILSELSAQDVAVASAFSMSYAELDDGH
RRMFRLLGLFPGQDFD
>D3PUA7/126-400

RKNEGQLILDGLQEAVRSQRPGVAIIHGPAGVGKTELALQLAHHVIREGYCADVQLFADL
HGFDAQRPACSADAVLSGFLRLLGRTSADIATLPSVEQKAARFRHLLTGKATLLIVDNVP
GPESLAPLLPGLASTLVLVTSRNRDGWPGLAAQLALQPLSVDEGMTLLRRFDVGDRVDAE
PEAAGLLVDRLCSGLPLDLVALGGQLADPAESTWTLSDHAARLQRFPRDQVQRPALAGSC
QGLSPDARRAFRLLALLPRDDFTVHETAILSDIEH
>D3PUW9/274-545
RQPEIHALTAAVSGTTRGRARVFAVDGMAGVGKSALVIHAAHRVADRFPDGQLYVNLHGF
THGSQPVSPVDVLDRMLRALGIPADKIPRLTDDRAAVFRSLLANRKLLIILDNAASEDQV
EPLLPAAEGCLVFITSRIRLSGLDDIHLLSLSPLSIEDSMELFRQIAGPDRIGGDTETLA
AIVGICGNLPLAVRIAAARLRDQPQWTISSLLERLRNGDAPLSELAIGHRGVAKALELSY
RHLDAPAQRMFRLLGLAPAMPVDAQAAAALAD
>D3Q6V5/116-315
LTELDAAVGEEPLWIITGAGGIGKTWLALRWAHDNAGQFPDGQLYLNLRGFDPDASPVPT
AEALRQLLYALDVDPSVMPRDTQSRAGLYRSLLADRRILVVLDNARDEEQVTDLLPGTPS
CATLITSREDLMGLTATQGARQVRLSVFDDTEALRALTNQLGEQRVKAEPDAVADLVRHC
GGMPLALGIVAARAAAHPRF
>D3QBK4/87-276
AALADLVTTHRQVTALGPGGVGKTRLALRVAAAVTAHFDDGVWFVDLVSTTDPDLVAAAV
AAALGLGEQPDRGMHESVISALSSQHALVVLDNCEQVVDAVAAFLERLLSACPRLSVLAT
SRARLMVPFEHVYPVPPLSLSGDGDSDAVELFTQRAAAAGQAPDASTREAIAHLCQRLDG
MALAIELAAA
>D3PWH2/128-390
RRHDLDRVLDMTASRPPDDAPLVITIEGMAGVGKTTLAQRVCQELESLGDGYDTRLSVDL
RGYDPTEPGADPDAVIRGFLVHLGMSPQHIDGLSPSERREHYARLLAERRALILLDNAAD
ETQLRPILLSVSPSTILVTSRRRLHDLDGAARLPLEPLSIDDALDLLRRLDPSGRVDTDA
TAATRLVHLCRQLPIELAAVGSQLASKPDWSLSDHVERLKVMPSFDISRPAFAVSYQNQS
AAAQRLFRLLALHPGREFTDDTA
>D3Q859/278-544
DEELAELDRLFDSGAAVTVLSAISGGGGVGKTALAVHWSRNRTERFPDGQLYVNLRGFDH
NEPLKPIDALSRFLRALGTPSAKIPAETEEASALFRSVMNGRNMLVVLDNARTAEQVRPL
LPGGQDNAVLVTSRNRLASLAALNDAKLMALDVLSLTESLELLAELIGADRVNADPDSAR
RLVELCGHLPLALRIAAASLAARSDGSISNLASELDSVSRLEILSIEGDPYSAVTATFDL
SVGALSIEARDLFLRLGMIPGEDIAEG
>D3Q902/268-533
TELARLDALALHPGGESIVAISGTAGVGKTTLAVRWAHRAAKHFPDGQLYVDMRGFGPSE
RVVTPSEAIRGLLAGLGVPADRIPSDFDAQTALYRGHLAGRRVLVVFDNARDAAQVRPLL
PGGAGCVAVVTSRNALTGLLAPDAAASLTVDTMPLPDCRALLANRIGATRGSDPAATDEL
IEACARLPLALSIVAARAVTDVSLTPARLSAQLRDERTRLDHLDAGDNVANVRAVFSWSY
RALSPEAARLFRLLSLHPRGEISAAA
>D3PYK6/284-540
AADDQVARGRRAVAFVGPGGMGKTSLALWWAHRVAADFADGQLFADLRGYSGEEPVPTSR
ILAGFLRALGHNDSDLPTGESELAGMYRTALAKKNVLIVLDNAAGPAQVRPLLPGDGNCL
AVLTSRDDLRGLKVDHDVATIGVGELSTPDAVAILSAHVTAAPEARDQLERLAELCGHLP
LAIRLAASRLPSGSAEELSALVTDLESGDRLATLSRPGETVGGLAATIESSYRRLDPAAR
EVFELLGAHPSGEADAA
>D3Q701/274-495
EQLSELDALLAENPQHSVAVLSGVGGSGKTALAIHWAAENREQFPDGQLYVNLRGFDTTE
PVKPVDALHAFIRALGHNGDSPASIDDAVTLYRSLLARRRVLVVLDNALNADQVRPLLPI
GVNVTLVTSRERLTPLTTTESAQSVSLDALSRSEAFDLLTVMIDTRRLHEDELAVYRLTD

LCGHLPLALRMAGANLANRPHTSVATFVDELDSSSDRLELLA
>D3PZ97/270-468
AEMAALDRASDDGQTLWVVAGPGGIGKTWLAVQWGHERRERFPDGQLHVNLRGFDPAEAP
LDPELAIRGLLVALGVESQAMPPGLEAKSALYRSLLAGKRMLVVLDNARDTQQVLPLLPG
GTSGTTIVTSRADLPALVTTHEALHVGLRPLDDPDAYRTLAKRMGTERIDAEPEAVAELV
AHCAGLPLALGILGVRAAI
>D3PYU6/273-548
RDGEVTELLEWLRPGDRDSPVTAVVSGGGGVGKSALAVRVAHRLAARYPDGQLYLNLHGN
TPDVKPLAHAEALSRVLRSLSVAPPPGGHDVDELAGLFRTATAGQRMLFLFDDARDAAQL
RPLLPSGKHCGVIVTSRDPLYSLDDVRHLELEPLGPADSAALFRRLLPGRRLADEPVAAD
RIVELCASLPLALCIAAARINSRPRWPLADFAERLADSDRRLSELAIDDRAVRASFATSY
EDLDAMQSRLFRLSSLLESPDFTVELAAAALDWRAE
>D3PYT5/276-542
EVARLLDLSTVDTNRPGAIVVGALDGMAGIGKTALAVHVAQRLTASYPGGQLFIDLHGFT
EGVTPVTPGQALDRMLRTLGVALQQIPPDVDERAALYRSLLADRRMLIVLDNAVNEAQVT
PLLPGASGSLVLITSRRRLVGLEGAQYLQLDVLSPDEAVSLLLRLAEISQPSDADRELAA
EIVTLCGRLPLAVRIAAAKLRHRRHWSLRTVRDRLLDERDRLHQLELGERSVSAAFTMSY
EDIDAEARRVFRLLSLFPGSHFDVLVA
>D3PUI5/296-564
RRDQLRQLDRLLDDTEPAPTTLITGPPGAGKTTLAVHWAGRHRDRWPDGQLYIDLRGYGP
EPLVQPIEALAYFLRAIGLPTDQVPPQQAEASALFRSRIAGRRLLIVLDNAATVDQVRPL
LPGGGDCLAIVTSRDRLTGLVAKEGARVLSVDVMADAEAQTLLSDAVGDARLAAEPDAAA
ALARLCGNLPLALRITAADLINHPQRSLTRHVDRLRTGNRLDALQVADDDGTAVRAAFRS
SYARLPEPVRRLFRLLGLFPGSEIGLESA
>D3Q874/128-386
RALTAVEDAWARRSQPVVAFTGPAGMGKTELAVQLAHRLPRKYPDLSRRFFVDLHGFDTR
LEPTPPFEVLAAIVKLLGVRDSAILALSTVEQMWDRLLNTLAGQRALLLLDNVSDAAHLG
QFTSGAPNLAIVATSQERLPLTEPGHGIEVGPLDYPSCVRLLDGFDRLGRVNREPGVTVR
LINELCGGRPFDLVALGGQLSDPAEAAWSLADHADRLAALPRDEASHPILAGSCHRLEPE
IRRVFRLLGLYPGFDFSAY
>D3PWS3/107-328
REAELGRLDAALKPPTSLLCSVTGLGGMGKTWLAVHWATTRLDRFPDGQLYVNLRGFDPV
TEPTPPYVGLRAMVSALGVPVDSVPADVDELAALFRSITADKRLLMVLDNARDAAQVAPM
LPGGDGSAAVVTSRSSLCELVTEYGARSLRLGGLTADDSRRLLGEQWDAERIAREPDAVE
GLISRCAGLPLALGIVAAHGVLNPDHPLGTIYRNLVDDSANS
>D3PXL1/277-528
RDKEIGRLDDGHDADAAPVWVISGPGGIGKTWLALHWANQRLDHFPDGQLYVNLHGFHPT
EEPVPAAAALNQALEALGVPATAIPADTEARAGKYRSLVAGKRMLVLLDNARDGEQVLPL
LPGGGCCTTLITSRRDLPGLGAAHGARSLRLGTLDDGQSWQALARHLGEQRLAEQPAAVT
ELIERCEGLPLALGIIGARAAAHDDFPLTALAEELRERADRLDMMDSGELVVGLRAVFDG
SYRTLASDEAQL
>D3PYV6/273-543
RASQLAALDAMLDQADGASVLATVAGAGGIGKTALAVHWARLRADRFPDGQLFVNLRGFD
HSAPLSAHDVLTRFLRGFGFNSEAIPSDLDEAAALYRTYLHGKRVLILLDNAARVDQVRP
LLPAGPGCFALVTSRDSLAGLTALDGARRVEVDTLGPRESLRLLADLIGQSRLDAEVEAA
TAITELCGRLPLALRVVGANLAARPSERLAEVAAELAGADRLERMVVPGDTRAAVADCIT
LSLPSIDENTRRFFLHLGLVPGTEISASMAA
>D2PM37/271-535
RHDEADAIVDRLTSPSGVPVVAISGPPGAGKTALAVRVAHRLRDEYPDGQWYVRLDGARD

SERDPSEVLRALLELAGAEVLTGDADTLSARLRSLLVDRRVLILLDDAKDSQQVRPLLPG
TRHSAVLVTSRNELTGLSVLDSALCTTVAVLEVDEAVDLLRAVIGSARVDREPEAAAELA
KLCGCLPLALRIAASHLAAQAGDSIASYAEELRAGDRLFALSVAGEPDAAVEVAFDRSYE
ALVPESQQLFALLGIVPGPDVTAPA
>D2PN57/104-341
RVQELAELSRYLIDSPAVSVTAITGMGGIGKTTLAIHVAHEVAEHFPDGQLYLDLRGFGL
GPPLEPLEALTYLLEDLGEVPPADLPTAASRFRTALAERRLVLLLDNAADHEQVRQLLPG
AGGCAVVVTSRRSMAGLGCRQYHLGEPPLHESIEMLRRISGRADAPEDECAEVVRQCGGL
PLAIRMAGARLASRPSWPVAHLADRLADGRRRLDELQLDDSGVRATLVLSIEQLAGSD
>D2Q1J5/266-526
RSEDLRRLTTALTHRQVMPLVCVDGMAGIGKTTLAVRAAREVAGHFPDGRLFVDLLGHTS
GRPPMRPEQALNHLLRGLGTADDQIPSDLTEAAALWRSQTATRRLLVVLDNAPDSDTVRH
LLPGAPGCAVVVTSRSQLTGLDPSVRIGLPALTAEDAANLIATVSGRDRSDPALTGLVER
CGRIPLALSIAGSKLRHRHSWTIAHLNDRMDAKGNRLAELSVDGRSVTTAFMVSYEQLPS
ECRRLLRLVSLVPGRDVDRYV
>D2Q1P0/110-379
DELTETGRRLGERVTRLLTLTGPGGVGKTQLALALTGGIGPKFDDGVCWVPLAPIADQAA
IAPTIAAATGLHPIEGARLVEEVAEQLGRRRILLVLDNCEHLVAEAARICAALLESCPNL
SILATSRELLRVPGESVYVVPPLALPAVEQQPESSPAVRLFVDRASARGNKPTGQIEQVA
RVVRRLEGMPLAIELAAARTNVLTVEELAAELETSFGILAGVSSTAEPRQQSLADAIGWS
HDLLTAPERTLFAELSVFVGGWTLNAAAAV
>D2Q345/279-544
KALARLDSLSSVHERALTILAVHGQGGVGKTSLALHWAHRVAQRFPDGQLYLNLQGYGPG
EPMHPEVALDMLLRGLGVSGAQIPDGGPARTALLRTVLADRKVLILLDNARDAEQVRPLL
PGAGCLVLITSRSQLRSLAAREGAHRIALDRFSADDSTAYLTATFRPHGVRATGDELAEL
AQLCGHLPLALAIAAERVTREPLTGVRSLIAELLDHPDRLKVLDVDEDDDGALRVVFDWS
YQVLEPERAQMFRLLALHPAQDLSLE
>D2Q312/293-559
RTEQVEQLAQSAGDRVMALAAIDGMAGSGKTALAVHVAHQLAPRFPDGQLFIDLHGHTDG
VEPVRPAEALGRLLQALGVPADQLPRHQEDRAGLFRGLLAGRAVLLLLDNAADEAQLKPL
LPGSRTCGVLVTSRQRLAGLDTTATVSLDGLSPDEAVTLFCHVAGRHRLTDTSRQVLIQI
AEQCGLLPLAIRIAAARFRAHPSWEPQQLLDLLRRHRDRLAELEAGPRSVAAAIDLSYRQ
LRPELRRAYRLLGLHPGSDFTAESATA
>D2Q1V9/287-553
REEAIGQLTERLSQDAALPVMVVSGPPGVGKSALAVHVAHRLRDRFPDGQWHVRLAGASA
APRDPAEVLGELLGLAGIDPYAVPADLEARAALLRSTLADRRVLIVLDDARDAGQVRPLL
PGTAGNAVLVTSRNELTALMVTVGARTTRLGMLGEHEAADLLSCMLGAERVAAERQAAAD
LAEVCGRLPLALRIAAGHLVSYPDQPIAQYVELLRTGDRLEELAIGDGPDTAVAAAFALS
YESLPRPARRLFALLGVLPCGDFTAPV
>D2PVM6/275-546
REDEAKELQAVITGADGDIPVAVVSGVPGVGKSALAIRVAHQAAPLFPDGQWYVPLGGAG
REPRKPGPVLADLLTAGGIGPHEVPQGLDARAALLRARLARRRVLIVLDDAADPDQVRPL
LPGTKSSAVIVTSRRRLSGLTALLGARGRHLNQLTAQESATLLTGLVGPGRLHPAEVDEL
TELCGHLPLALRITAARLLVETDVSRHLHDLRQGSRLDGLVLDDDPNIAVRAAFGVSFRS
LGSSAARLFNLLGLVAGPDFTVDVAAALLATK
>D2PQW0/364-627
REDELTTICTALARPDAVSSRVVLINGIAGVGKTALALVAAHRLRAQYPDGQLYADLRGN
ATLLPEPIQVLGRFLRALGVPGRRIGTDETEAAALLRSELADRRVLVLLDNAQDAAQIQA
LLPGAGRSDAIVTSRRRLPGLDTAGVVDLEPLTRPEAIQLIAATARTHRLDADTEGATAL

AEACARLPLALRIAGARLATRPEWTVSDLARRLDDGNRRLTELSIGESSVLNSFQLSYAD
LSLEAQRAFRLCGLHPGDDFSADS
>D2Q4X4/107-353
RTAELEALTRILTADDVVGTVRMAAVTGMGGVGKTSLAVHAAHLTADAYPDGHLYLDLRG
YGPGEPVQPAEALSQLLRSLGIDGHSVPDGVDEAAALYRSRIAGLRMLVLLDNANGAAQV
RPLLPGASGSAVIVTSRRGLTALPGFLQLSLSPLSEADSITLLGRIAGNDRVAAEANAAR
RIARLTGRLPLAVRLIGARLAARPGWPVEHVMNQLQDEQRRLDELGTGKSGVRSNIAASV
EFLAASD
>D2Q1F8/272-525
LAALDRMRARSARTIALHGPGGVGKTTLGLQWATRTASDYPDGQLYLDLRGYGPDEPLEP
LAGLGMLLRSAGVPDRAVPATVAERTALLRTVLSDRRMLVFLDNARDSDHVRPLLPGGDC
LTIVTSRNELLGLAVRDGASRHAVRLLSMAESVELLEATLGVPADRSALEELAELCGHLP
LALTIAALQSSRHDSPVSTTVAALRAQQDRLDLLSDSHDSSTDLRTVLAWSYDALDRQEA
RAFALLGLHPTPDF
>D2PTQ9/267-509
RDAELAELDRVLLSSTGSIAVISGTAGVGKTALAVRWAERASVQFPDGQLYVNLRGFDPT
GELVSPEDAVRGFLDALQMMPGRIPSGAAAQYALYRTLLANRRMLIVLDNARNAAQVRFL
LPGGNTCRVVVTSRNALPGLLAAEGAHPLSLDLMDRDQARSLLGDAVGRQRLQAEPEAAE
ELVTLAARLPLALSLVAAHAAAHPSWSLEAVATDLRRTRQGLDVFAGEDEATDLRAVFSW
SYQ
>D2PN55/92-353
RQSVLAELTAVLTESSQQAAAPPVVVAGMGGIGKTAVALRAAHLVADAFPDGTLFVNLRG
HAGSAPTTAIEALVQLLQELGAAGDSIPGDVAVAAARFRSGLAGRRMLIVLDDAASAEQV
EPLLPGAPGSAVLITSRSWLLGLAGARHLPLDLFDEAEAIDLLREVGVDQVDEDLALAGT
VARLCGLLPLALRIAGGRLAGRPGQSLAELADGLADEQRKLELLTAEDTGVRAAIRLSVD
ALAAADRPLQRSAAAALPLISQ
>D2PUI6/305-516
LRVMDELFDSAADEPAEPEMIVLHGLGGIGKTALALHWVHSVRERFPDGQLFIDLQGYGP
GEPVDPASALHSLLVGVGLPEAQIPDDVEARSSMLRTMLADRQALLVLDNALNADQVRPL
IPGPGSLSVVTSRNELHGLAVREGADRIALDELTSAESKALLRARLATQDVGDNLLAELA
NLCGHVPLALSIAAERSGSAPEAQITALIDEL
>D2Q119/299-563
RERHLEQLRDLVCGRDPVRPPIAVISGAPGTGKSSLAVRLGHLVRDSFPDGQIYLDMRGA
TEPRDPGGALTDLLISLNVPDFAVPADPDRRSAMLRSELASRRVLIILDDVAAAGQVAPL
MPGTGASAVVVTSRNRLMDLAGADPIPLDTFEDGEAAELLQRVAGNARIDRGSPAAEEIL
LACGNLPLAIRIVASRLAQRPDLSPGELARRLRDETHRLDELSIGELAVRTSADLSYDAL
NPEEARLYRLIGHFAVGEFSARVLE
>D2Q133/101-304
RTTELDTLVALLRAPHAAAPGIVISAIGGMAGIGKTTLAVQAAHRVADVFPDGQLYLNLR
GGGRDPVRPVDALAALLLGLGVPPSGDPQDERIAAARFRTAMAGRRMLVVLDDAAGIEQV
TPLLPGTPSSAVVITSRRRLTALPGVRHVDLDLLTEHEALQLLGEVVGPEWVEAAPNDAR
RIVQRCGHLPLAIRIAGGQVRGSS
>C6WIY7/59-324
EELQRLNGFLNDHLGSDVSGVGLCVVTGTAGVGKTSLAVHWAHRVSAAFPDGQLYVNLRG
YDPGPPVTPAHALDHFLRSLRDPGEPVPADLESRAAAYRSMLAGRRVLVLLDNASDVAQV
RPLLPGTAGCLVVITSRSRLAGLVARDGAHRLVLDVLTEPQALELLHGVTDTHRPPDDPA
QLVELAGLCAHLPLALRIAGERAGRRPHLPLRQLIEELRDESSLWDALTSDADEDGVRTV
FAWSYRALTPDAGKLFRRLGLHTGAE
>C6WCA4/312-552

AALARMDELLRAQRDSGVVLVVTGPAGVGKTALALHWAHRVRDEFPDGQLYADLHGYDPN
QEPLGAGEVLNRFLRTLGVPAEDVPVGVDERSALFRTLVADRRMVVLLDNARGSTELMAL
LTGAPSCVLVTSRRRLVGLVAHAEAHLVELDVLDQDSALELLGRRDPGEMGALRRLAVLC
DGLPLALRIAAARLAAPSLRAAELVAELDDEHGRLAALGLEDEDSTVRAALDASRRALPP
R
>C6WKI7/62-328
RLDRLLRPARAQAGPALLVLVGAPGVGKTALAVHWAHRVRDRFADGALYVDMRGYGPGFP
LDPSAALDGFLRALGTPPDHIPDDEADRASLFRSLLDGKRLLVVVDNARSPHQVRLLLPA
SPLCCTVVTSRSVLSGLVVREGAARVTLDALSPAESADLLGELIGPDEVAADRPAALRVA
ELCGCLPLTLRIAGERASEWPGPRLPRLEAELRDERQRLDALASPDDELSDTRVVFSWSY
RALGPELRAAFRLLGLHPVPDVGVDVA
>C6WD01/100-289
AGAAVLVSAIGGAGGIGKTWLALAWAHHRLERFPDGQLFVDLHGFSPVDAPVEPAMAVRG
FLDALGVAPGRVPADLGAQTALYRSLLAGRRMLIVLDNAATAEQVVPLLPGSPTCTVLVT
GRHRLASLIDRHGARHLSLDGLSHDEARTLLTARLGAARIASASAAVDELIRLCGGHPLA
LSITARNVDT
>C6WHS7/290-546
LAALEADLGGQDSAIGLLVGPAGVGKTALALHWAHRVAADFPDGQLFVNLRGFDETEPLD
PRTALVGFLRALGVDDSQIAVDLEEQAAQFRSLVAGRRVLVVLDNARSAEQVRPLLPGSA
RCMVLVTSRLLLDDLVVTEGATSLQVPQLEEGTAEDLLAAALGRHRIEQEPEAVAELVEL
CDRLPLALRIAGARLASRPRWTIQSLVDELRDEQGRLSGLSLEAGTGVHAALAVSYRELP
EAAARLLRRLGLHPGTD
>C6W924/281-493
RADELDRVTALLAGPAAVVAVCGPPGVGKTAFAVTVGHSVRERYHHGQLYADLRGHSTSP
PLSTTTVLGRFLRALGARPDSIPADEAELVRAYRDRLRGRRVLITLDNAASAAQVLPLLP
DVPECSVVITSRNELAGDVGAAAVRLDVLRGDEAWMLLTRSLAPEAADEQGDALRELARL
CGYLPLALRIALGNLVGAHTTDIRSYVDDLRGG
>C6WNL5/139-392
LDAAVAGPGSLPVVALGGAGGTGKTWLALHWAHHNVDRFPDGQLYVDLRGFDPGGTPVSP
GAALRGFLDALGVRAPSFPTDPAAQVGLYRSLVAGLRVLVVLDNARDLAQVEPLVPGGAS
ATVLVTSRHKLPGLVTTHGARSLRVDVLGPAASRDLLGRHLGAERVAAEPEAVDAVVRTC
AGLPLALGLVAARASTEPDLPLRELARELGRSASSPLDALELDDTGLRSTFSWSYRDLPA
DAARALRLLGACHC
>C6WF44/280-547
SNSVHLVETLLRRSHGVPVVTLSGQPGVGKTALAVHAAHRLRGDFPDGQLYVNLRGHAQG
PPLSAVDVLPRFLRAQGVAPESVPLDPDEQEALYRSRLTGRRVLVVLDDAASAEQIRPLL
PGSPGCAVLVTSRDALRGLAVSHAATNVRLDVLDDGEARALLSGVLGADVVGCQRAATGE
LVALCAHLPLALRIAAANVLSRPGTTVAEHVEELRAGNRLAALSVEGDERAAVQAAFDLS
YAALKPELAQLFRLLAVAPGDLTPDLAA
>C6WJB0/306-565
TGQMAGAGTPVVVVVDGMAGIGKTALALTFAHQVSDRFPDGQLYLNLRGFGPAGTPMAAG
EAVRTCLDALAVAVDDIPTSLQAQAGLYRSLIANRRMLVVLDNAADAEQVRPLLPASPAS
MVIITSRRRLESLVATDGAIPLALEVLNAAQAYALLEAGVGAARLAAEPDEVDELIARCA
GLPLALRIVLARALTRPGFALAALVSTLRAAQDRLGSFDGGDPVTNLRVVFSWSYRDLPA
DTAWVFRSLAVHPGPEMSES
>C6WAZ2/296-556
EELITAAEEVLTGGAGRRAVGVVVVIGRPGVGKSTLAAHLGHRVAEEHFPDGQLYCDLRG
GYGDAGGSADVLGRFLQALGIPGAMIPVEHTARTEMYRTLLADRRVLVVLDNAVSERQVL
PLLPGGGRCAVVVTSRARLTGLPGARQLELDVLDREQSLELLGRVVGERRVAGEPEAAEA

LVRTVGGLPLALRIVAARLAARPHWSLASMVHRLASERHRLDELAHGEMTIRASLSLTHD
GLDQPTRRLFGLLSLAEGPSL
>C6W971/304-544
PDRPAAPVVVVSGAGGTGKSALAVHAAHLLAEQYPDGQLFTDLRGHGAPPSASTVLARFL
GALGVPVEDLPPGLDDRIALYRRHLTGRRLVIVLDNARTEQQVRPLLPTEPGCLVLVTSR
ARLAGLGSAVDLEVFDAGSAVEMLGRIIGSDRVASAPDAARRIATLCAGVPLAIRAAGAK
LLARPHWPLKSLATRLSDERRRLDELTVGDLAIRSCLGLNYAELDERAKHAFHLLCLLDL
P
>C6WLN7/261-486
LAVLDRLAAEDGQAGAPPLGLLVGGVGVGKTALAVRWAHANADLFPDGQLFVDLGGHDPH
HPPSAPGAVLAHLLHALGVPPERVPVAAERPALFRTAMAARRMLLVLDDARDAAQVWPLL
PNTATCRVLVTSRDPLRELVARSGAVPLRLGGLGFDESVALVRGIIGEARAGRDPDALVG
LVELVELCGRVPGALLAAAAHLASKPHWGVPRMVRELNRPRSRLSG
>C6WGN6/287-562
LAWLDRLCDTRDRDATTTAVLDGPPGIGKSELAVAWGHRRAAQFPDGLLFAQLGGHADDE
RARVGPDEVLARFLLALGVPADAVPRGTADRVGLYRSVLAGRRVLVVLDDARDAEHVRLL
LPPGSGSLALVTSRLRLGSLVVSAGARVLTLDVLAEDESARLVDEAVGKPLSEQEPDAVR
DLARLCGNLPLALRIAAARLVSSPEWAVASLVDALADDSTRLRALDLADADAGGGVGVAR
ALALSYRELPDELAEVFRAAGLVPGRRVTAQAVAAL
>C6WRY9/42-304
EFDAAARRAAENGEVALLVVEGGPGTGKTAFALNWAHRNADAFPDGQLFADLGGGGPLGA
PVLPVLLGFLAALGVVPSAAAKGDVASVAALYRGAIAGRRMVVVLDNATGTAQVEPLLPG
AATCAVLVTSRSRLSGLRMLGAVVHRLAPLGRSDSLTLLARYLGDAVEADPASVEVFLDC
CAGLPLALAIVAARAAENPEFDLSVLADELGDVRRRLDALETGESGGGLRAVLSWSYEAL
DAQTARAFRAVGAVPAEEVTAHA
>C6WBP7/159-394
CTPALPGIVVVSGPAGVGKTALVVRWAHRVGRHFPDGTLYLDLRGYDVGRPVSGRAALGA
LLRGLGAAGPPSSAALRTELAGRRALIVLDNARDAEQVCPLLPGVPGCLVVVTSRDSAAG
LVVRHGARRLDLDVLPLSESAALLTALTGGRLDADPAAARRLALRCCGLPLALRLAAERL
LATGGDAAALAEGLTAAPLDLLATTADRRTDVREVLSWSLRHLPPEAVRAFHVVGS
>C6WB03/103-364
AIRAAAARARGTEVVAVHGAPGSGKTSLVVRAAHELAASHPGGCFFFALRGVDATPVRPD
EVTRRVLVALGEDPPHSEAERTDRYRALLRERSTVLVLDNALDEAQVRPLLAEGARSLVL
ITSHQVLGGLEGATRLGLDVLRESESTGLLAAIAGEARTGAEPEATLAVARLCGGLPLAL
RIAGNRLVSRPRWTVAHLAARLGDERRRLSALTAGDLGVRPAFELSYRQLAPLPRALFRA
LSLLPGSGCAPESAAVALDVDA
>C6WNX9/189-454
RDAELDRLDMALHAAQAEGVPPVLALAGAGGVGKTWLAVRWAHRNAGRFPDGQLFVDLRG
FTPDGAPMETSVAVRGFLDALGVEPSRIPTDLHDQAGLYRSLLADRRVLVVLDNAASAEQ
VAPLLPGGRSSAVIVTSRNALRPLFVQHDVHHERLPPLTADEAEALLTARLGAQRVAEER
EAVAELVSLCRGSALALALVASRARLRPRAPLAGFAAELRESGLAALDDDDPAVSLPAVL
SWSYDALPPERRRLFALIGLSPGPDL
>D8HY85/295-549
LAEATGILTRGPDSTALGVLVVVGRPGVGKSTVAALLGHRLGEQHFPDGQLYCDLRGTRA
EPSGAAEVIGRFLVALGIPGPMLPEGLAGRADMYRTLLADRRVLVVLDDAVSEQQVLPLL
PGNPRCAVLVTSRSRLAGVPGARRVELDVLRPEESLELIGRVIGHDRTDREREAAGALVR
TVGGLPLALRIIAARLAARPHWTLASMVQRLASERHRLDELAHGEMTIRASLSLTHDGLD
PRSRRLFGLLSLPEA
>D8HTP7/235-489

LQAAILTGDPLPGPAPVVLTGAPGTGKTALAMHVAHGLADRFPDGQLYVDLAGTGAPRDP
AEVLADFLHALGVTGNTVPPGLGQRAALFRSRLAGRRTLLVLDDAAAAAQVRPLLPADAG
CAVLVTTRGRLPELAGAKHVELPVFGEREAARLLAELAGPDRVDGEPAEAAEIVRCCGYL
PLAIRIAGARLAGRQAWSLRTLHDRLADESSRLSELRVGDLGVRPSFELSLRQLPPSART
AFGRSAVLGAQDFPS
>D8I033/183-335
LLIGLRTALCSGQPAVVQAMNGMGGVGKTTTAIEYAHRYAKDYDLAWWVPSEDPALIAER
LAALAQALDLATDQDPPTIALARLRGTLQTRSRWLLVFDNAEDATALRPLLPDGNGHVII
TSRNPNWTDVGAALPVREFARAESVDLLRSRRP
>D8I4K7/286-492
AELAELDRLLTPEGSAGRTFAVTGMTGVGKTVLAVRAAHAARSRFPDGQLFAEFGPDTEP
VDVLGGFLTDLGVPAADVPAGEAERAKLFRSRTAGGCGLILLDNVSSAAQIRPLLPGGRW
VVLATGRRLTGSLPGVVPMLLEPMSTDDGVTLMSKVVTSRNILHERGTVAAVVHRCGGLP
VALRALGERLSVNRHWPVTKLAARVIG
>D8HMW4/260-523
AELAALDRMLTPDGAVAVISGAGGLGKTWLAVRWATGHASRFPDGQLYVDLRGFDPAHEP
VPAERAVRGFLGALGVASASIPSEPEGQAALYRSLTARRRLLVVLDNARDTRHVTPLLPG
GPSCSVLITSRHELGGLLTTHGASALPLRTLDEDQARELLAHKLGAARLAAEPEAAQEIL
DQCGGLALALAIVAARVAAQPQRRLAALAAELRESRLDALDTGELAASLRAVFDASYRSL
SVGAATAFRLLGLVPTEDIERAAA
>D8HVM4/30-306
AGQLDELDRLLDGSRTSTSVVVSSLSGTAGIGKTALAVHWGHRVRDRFPDGQLYVNLRGY
DVDDPAAPEDVLADLLRPLGIAEAAVPTGLDRRASAYRSLIADRRMLIVLDNARTPDQVE
PLLPGTRSCFVLVTSRDALPGLSIAHGTSRITVDLLPHGEAVELLRRRLGDRADDQPAAA
DALVEQCACLPLALRTVAELALARPWTQLHELARELGDERWKLEMLGSSGHACTAVRSVF
SWSYRQLSAREAQAFRLLSVAPARCSIDDYGLAALAG
>D8HNG2/231-482
TAADHGTLGTAIHVIDGVPGIGKTACAVHAAHQLAAYFPDGQIFLDLHGHRPEEAPATPA
DALASLLLLQGVPTLAIPADLDDRARLWREKLAGKKILLLLDDAVDDAQIRPLLPGGAGS
LVLITSRHRLESLADAGRVPLQTLPPGEAAAMFDRHTSPAQHDPGAVAELMTSCGNLPLA
ISLTAGRLHRHPDWTVRRLADDLDQSRNRLKTLRADDRSVAAAFELSYRDLTPDQQRLFR
RLGLHPGRHLDA
>D8HTX3/134-324
VADGAVLVISAVAGAGGVGKTALALHWAHRELDRFPDGQLYVDLRGFDPAGPPMPPETAI
RGFLDAFGVAPSAIPADLTAQAALYRTLVSGKRMLIILDNARDSAAVEPLLPGSGGCTVI
VTSRHHLTGLVARGAHAMRLPMFTPGEAGELLVKHLGAAKVDAEADASAALVRFCAGLPL
AISIVAARAGS
>D8I888/266-521
AELAALDEPGDVWVITGTAGVGKTALAVHWAHTARTRYPDGQLYVNLRGFDAEDEPLTPA
AALAQLLRTLGVDLRDVPPGLDDQSKLYRSLLADRQALVVLDNARDTAQVLPLLPSSGRV
LVTSRHRLDELVARVGARSLSLAQLREADSRALLTALLGDRTAAEPEAAAELARLCGHHP
LALRIAAANTGVASIGELVDELRGDPLAHLGFDGAGESAVAKAFSVSYQALPPALRQAFR
LLALVPGADFTAIAAA
>D8HNI6/282-538
AELDRLPPGGASGIVVIEGTAGVGKTSLAVHWSHRVRDRFPGGQLFLDLRGHSADTPVTP
GAALAGFLRALGVPAETLPSTVEERSALLRSRLAGSRTLMLLDNARDADQVRPLLPGPGN
LVVVTSRNQLRGLVARDGARRIALRSFDDRDATALLAGSVGSQRLAAEPGAVAELVQLCG
RLPLALALAGERASRFSGVSLAGIVEELRDQRLRLDTLRDPEDAGTDLRAAFSWSYKALR
PAAARFFRLLGLHPGHG

>D8HWW6/314-593
HTAPTAVVIEAIDGMAGIGKTTLAIHAAHRLAGHYRDAQLFIDLHGHTSGQAPITPAAAL
DTLLRALGVAADRIPLEPDARAALWRAELAGRSVVVVLDNAADAAQVRPLLPGSPGTLLL
ITSRRRLVGLEAAHILSLDVLPEATAVALFSGIVADDRPAAEAAAVRDVVALCGHLPLAI
RIAAARLRTRPAWTVAHLADRLRQAGRPLAELSAGDRSVAAAFALSYEHLDEAGQRMFRL
LGLNPGPDIDVPAAAALAAVAPAEAERLLESLVDDHLLQQ
>D8I8G8/237-514
IAKLDSLLVEPGASGPGETTIFLIVGAAGMGKTALAVHWAHAVRSQFTDGDIYIDLHGYD
PVAPTPLEQILERVLRALGVTTRNMPADLEGQTALYRSLLADRRVLIVLDNAATPEQVRP
LLPGSATCRVLITSRHRMSGLVAREGAARLTLDPLHPTEAYSLLGQIVGEDRVAVEGAAA
AEITRACAYLPLALRIAAERVAAHPRLTLAELARQLSGERDRLDLLAADDDESTAVRVVF
SWSYRALPPAVSHVFRLLGLHSGPDISLPAAAALTNLT
>D8HNX5/267-535
AAIRAAVTRRTDASPLVLVTGMPGIGKTALAVHAGAASVHTYPDGQLYADLRGADARPAA
PHDILAGFLRALGVPARSMPATVEERTAEFRSRTAGKRVLVVLDNAATAGQIEPLLVAEP
GCATIVTSRSVLPDVESAVRVPLGPLPDEVAGTVLANVAGAARLGDGDGDGTADGNGSGN
GNGTVATVIEACGGVPLALRIAGAQLAAYPRLSVAELAARLSDESRRMRELVAGQRSVRH
SLDTSFRLLDRNARAAMLALAAIGAPTFT
>D8HQH9/418-670
RQELLRELSERVMSGTTAVLPSALHGMGGIGKTQMAVEYIYGHLREYDIVWWISATEAAQ
IRVALTELAQALNLPGSGEANTAIPAVREALRIGRPYRRWLLVFDSAEDVAMVRQFFPTN
GPGEILITSRNAEWASVARPLEVAVFERAESVELLRRRGPEIDVAEAEQLAERLGDLPLA
IEQAAAWRAETGMPVQEYLRLFDEKVAEILDTSSPADYEVSVAAAWNVAFEELGKRNPMA
HQLLQICAFFAPE
>D8HRL5/266-522
REAELAELTGVLGAGSGVPVAVLSGEPGAGKSTLAVRAAHRLRARFPDGQLYVPLAGREI
GEVLADLLRALGVPGPAVPDDVRARAAVFRGRLTDRRVLVVLDDAVDPEHVRTLLPGTPG
CAVLVTSRRRLSGLAGAHRLAVGPLSGADAAELLHRLAGARVARERADAERIITACARLP
LALRIAGSRLAIRPHLRLGELAGRLEDEVRRLDELTVSDLAVRSSIALSYEGLRPPARRA
FRLLGRCRLADLPAWAV
>D8HXB8/269-521
RREDVDHVLRGLRPAGSAARVCAVSGKPGSGKTTLAVHAAHRIRDQYPDGQLYATLHGNH
PEPADPDEVLARFLHALGVADAAIPGEPAERVDLYRSLLADRRVLVVLDDAAGEKQVRPL
LPPGGGCAVLVTSRVRLAALEGAALVDLHELREEETLELLAKLVGPRLAAEPEAALEIVR
LCGHLPLAVRIAGARLAARPDWTLARLAQRLGRKQRLLNELVLGDLEVRGSLAVSYDGLG
EQERAALRRLGMT
>D8HQX6/316-582
QLAELDALLRRDDGDTGARIAVLSGTGGVGKTALAVHWAQRVPAEFPDGQLYLDLHGYGT
VRPVDPGDALSGFLRALGVPGADIPAEPDERAAKFRTALTGRRMLLVLDNANAVGQVRPL
LPGSPTCLVLVTSRDALPGLVARHGARRVLVDLLTEAEARDLLRALLGPRVDDEPEAAAA
LIGYAARLPLALRLVAELALSREGERLAALAAELADERRRLDLLDGGGDPLTAIRAVFSW
SYRNLAPDAARVFRLCGLHPGRDLTPA
>D8HPX6/349-609
VGRVAETGLLTGAAGPAAVYGICGMAGVGKTALAVHLGHRFADRFPDGQLFADLHAHGPD
GPLAPEAALDSLLRQLGVDGARIPERLDQRAALWRSLLVGRNALVVLDDAADAAQVRPLL
PGGPGSLVLITSRARLTSIDVLTTVSLDVLAPAEAAQLLTRVAGPDRVGPDPAAGEITRL
CGYLPLAVRIAAARLRDRPLWTVSHLAGLLRDEHRRLAELSTGDRSVGAAFTLSYRQLTE
PQQRLFRLLGEFPGTDVDAVA
>D8HM70/286-550

RGALIRDVVTLLQERADAGTPAVVVLSGAPGVGKSAVAVRVAHAVRDEFPDGQLHVDLAG
TSSSPRAPMSVLAELLRTLGIPDAGLPRELAERSALLRSRLAGRRMCVVLDDAGSEAQVR
PLLPGAGACAVLVTSRIRLPGLAGAKPVDVDLLPEAEAARLLEGIVGAERVAAEPESAAA
ILRQCGHLPLAIRVAGAKLTHRPGWTLRLLADRLGDERRRLDELRVGDLAVRASVTLSYD
LLPMSAATAFRGLGLLGPVQFPTWA
>D8HUZ4/212-444
TDKLIDIIVRSTGNVLADQVTSIVGAGGIGKTAIAYEAVRELRRRGHFTKVAWTSAANPQ
ATGCSVTTEGFTTVYWLELLRDLAAQLDFDLGPSRTLWESEFARQVAGLPSGDQLLVVVD
NLETLPDATDAVGRLRRMGVFKPHALLVTTRWELQSHLPDIAEFRVRPLSPEDSVLFIKH
LSMADPDLQEAGDRALAPILDATEGNPFLIKLAVQQYLSSHRPLDHVLRDLRD
>D8I5M3/26-162
LGEAKRLLATARLVTLTGAGGVGKTRLALRVAADVRRAFPDGVWLVELADVGDPHLVPNT
VATAFGLRVTSDQAAGLAEYLEDKQLLLVLDNCEHVADECATLVAKLLAVSAGLRVLATS
RHTLHVEGEHLLHVEPL
>D8I6U5/245-499
TVTAEAEKGTLGMAIHVIDGMPGTGKTTFAVHAAYQLAAHFPDGQIFLELHGHSPSQVPV
TPADALASLLFLRGVSTLGIPADLDDRARLWREKLTGKKILLLLDDAVDGEQIQPLLPGG
AGCLVLITSRHRFESIADAGRVSLQTLPAAEAAMFDRFTSPEQHEPGAVAELMTLCGNL
PLAISLTAGRLRSHPSWTLRQLADDLNHSQNRLKTLRADNRSVAAAFELSYRDLDPDQQR
LFRRLSLHPGSHIDA
>D8I315/268-460
VARALRGPAPVVLHGAAGAGKSALAVQAAWRARRRFPDGQLTASLRAPDGTPAEPAAVLA
GFLRRLGATPAELADRAGLAGLWRGYTADRRLLVLFEDAASEAQVRALLPSGPGCATLVT
TRRRLPGLCGARAIPVPSLSTEDAWALLAAIAGEARLRAEPEAAQRLLEHCAGLALAVRI
AGVKLATRPDRRV
>D8I3F7/220-467
RAMEHRLLDKLSVESRLLVLSGPPGVGKSTVAMQWCHQVVDHYPDGQLYAHLGGPAGSVT
VEQVLGHFLRGLGVAAPRVPVGLAEQSALFRSLTAGRRLLLVLDDVESADQVRALLPSSA
VSTTVVTSRSRLSGLVANGGRLIEIAPLPEEDGVELLARLVGDGRIERERPCAQQLASLC
AGLPLALCVAGARLVSHPRWSVEKVVAELVDEGRRLAGLSLAGDLSVQVVFDMSSETLSP
PAARLYRL
>D8HYP2/281-532
VAGRQRASVVVLAGMAGAGKTALALNWAHRRRDSFPDGQFFLNLNGFAGLPATSPADALT
QLLGALGVSVADLPKSVDEAGALFRTLVADRRMLVLLDDAASADQVRSLLPGGRACFTLV
TSRGSLNGLVALDGARRITVGSLSTRESCVLLSEIAGADRVANEHPATLEITRLCARLPL
AVRIAGATLAENGSLSVTGYAAELATDRRLAALQVADDPDAAVRVALDRSYANLRPPARR
LHALLGLLPGPD
>D8HQA2/365-620
LTAAMRAQAEPGGTVVISAIGGTGGIGKTWLALHWAHQHIDRFPDGQLFVNLRGFDPTGD
PLTTQTAVRGFLAALSVEPSAIPAELDAQVGLYRSLLAGKRMLVVLDNARDTAQLAPLLP
GSPSCAVLVTSRSRLSGLVTAHDAHPLTMDVLSESAARQLLQARLGAQRPAAEPGAMAEL
IACCGGFPLALSIVASQARAHPQFPLAALAAELRDASTRLGALDDTDQAVGVPAVLSWSY
RALTPEGARTFGLIGL
>D8HX90/40-308
REAVLERLDEIWARRGTRRPLVVLNGIGGVGKTTVAVHWLSRRRAEFPHGLLYANLSEVD
GSPVPPENTLHGLLTSLGVAAEDIPSGPVGRAGAFRAITAGRSLALLLDDAVSAAQVRVL
LPAAPSVVVLVTSRRRLAGLGIDDATLVDLEPLGPGASARLLDTAIGAERLAAEPAAAGS
IIATCGGLPLALGVVAARLRARPLRRLEREARTYARYLREANGLPEDIQDVQAVFDVTYA
DLPGGAARLYRVCGLHPGPEVAIEVLAAV

>D8HJL1/278-545
WLDGLVTRAEAGQTTIAVVTGTAGVGKSSLVVWWAHRVAPRFPDGVLFASLRGFDPHHPP
LEPAELLTQFLLGLGVETAKVPELLHERVALYRSLIAGRRMLVLLDDARTAEQVRPLLPP
SARTMTVVTSRSRLDGLAVSNAAKQRVLGTLAPDDAVALIEELAGPASLNHALARLCGYL
PLALRIAGARLSASAQRTAEELVDELGNERTRLAALQVDGADDSVRAAFDVSFRGLPGEV
AETFLQLGAVPGVMVGPHVMAAVAQITV
>D8I649/52-309
SAQSGRAVVISAISGTAGVGKTALAVMWAHRVRSSFPDGQLYVNLRGYDPGPPATPGEVL
DGFLRALDVPAAKIPPALDDRAGQFRSVVAGRRLLIILDNANSAEQIRPLLPGSSSCMVV
VTSRDSLTGLAVSVGMHRITIDRLPAEDAVTLLRTIVGSHRADSEPEAVADLARWCDRLP
LAIQIAAQRAVAHPHVALAELTTELADDAQRLEVFSVGVDEYTAIRPVFSWSYRSLPEAQ
ARMFRLLGVAPGLDISVP
>D8HX92/304-553
AFTPAVVTLTGPPGVGKTSTAVWWAHRAADRFPGGVALLDLHGVGPAPRVESADVVDNLL
SALGYPVDRIVGAAARATRLRGLLCDRPMLIVLDNAQDTAHIEPLLRILADCAVIITSRQ
RLTQLARRHHCPVLTLEPLSSDSAVELLDRRLGTRLDREPDVLANLARLCGGIPLALTIV
AERAATRAGNRLRTLVDQLRDPALLLTIGDDGDGEASNLLSAFSWSYHGLDPSAQETFRL
FGLHPGLEVG
>D8I8R7/266-536
TAELARLDVVLAEVGQPDAVVVATLSGMAGVGKTTLALQWAHRVADRFPDGQLYLNLRGY
DPGNQVVNPADALRVFLAALGVPAERIPAPLEERTALYRSLVARRRVLVLLDNARNAEQV
RPLLPGSSGCLAVVTSRDQLFGLTVSEAAHPVVLTPLPVAEARQLLTNRLGDVRVATEPT
EVDEIISLCGRLPIALAVVAARAATNDGFKLAAIAGELRESEAVLDVLSGGDPATDVRAV
FSWSYQGLSDPAARLFRLLALHPGPQIGVRV
>D8HIM9/138-384
FAEGGRVAVIAGMAGVGKTALAVHAGHELLRTGRCDNALFVNLRGFHPDPGRPPVDPAAV
LDSFLRLLGVPGHAVPADLAGKTRLYQERSAGRRLLVVLDNAGSEEQIRPLLPGERGGPV
LVTSRLAFAGPAGAAHLPLDVLSPDDALAYLRRTVGAERVDQAPGVARRITAELGRLPLA
LSLAALWMAAPAHRSWLLADHLERLEQRRAHLRLDNAVDASISLSYDQLAEPERRALRLL
ALHPGAD
>D8HSB9/97-358
RDAEVEALTDHLKEVGGRQAAQVCVLHGLPGVGKTSIALWVADALRDDYPDGRLYLDMQG
YHPDSKPVSEEEALDRILRRLGVPGELVPRNTDDRAALLRQKLVNRRVLLILDSVKGARE
LARLLPPNGRSAVIVTSRQPLTALGPSFHQQVRTLAPAAAAELFRSVAQLPQGSDERGDR
AVITEITRMCYQLPLAICIVASRFRDNPVRRLEDVAARLADEAARPREFDDGERSITAVF
AASCATLSDGQQAMLAMTALHP
>D8HNS6/131-395
REQDLAALDELLSGSPGTRVALVTGMPGAGKSALAIRWAHRAADRFPDGQLFVNLRGFAD
ARPADPVDVLSGFLRALGTAERETPREADRLAACFRSAVAGRRLVVVLDDASSAEQVRPL
LPGSPSCAVLITSRTKLVGLVAREGAVPVELGPLGTDDAVALVRHAAGPDRVDADPAATA
ELAEACGRLPLALRIAAANARVRPGLPLAAMVEQLRSADPLAVLDAAVDRETAVQNAVAL
SYRALNSPAQRVFRLLGLFPVDGIA
>D3D0G9/768-1023
RAELLDALHERLQTGTTAVLPEALHGMGGVGKSQLAIEYVYRQLADYDVVWWIPSERTTQ
INQALVELAQRLGLGVGQEANAAVPAVIEALRVGKPFSNWLLIFDNADDPRAVRDFFPAS
GTGRILITSRNAQWAGAARALEVDVFQRGESIELLRRRTTSIGEVDADRVAAALGDLPLA
IEQAATWLAETGMPADEYLRLFEDKRQELLGTAPPLDYRMPVRAAWNVSLDRLATSNPAA
LRLLQVCAYFAPDPIS
>D3D821/195-419

AVDDLAHAVDGAGTAVACQVLAGMGGVGKTQLAAHHAHQMWQSGAVDVLVWVVAASREQI
QTVYARAAADLTGTDPTDPTAAEALLGWLADTEKRWLIVLDDVADPADLRGLWPPDRPNG
RVLVTTRRKDAALTGPGRHRVDVDLFTSEEASAYLTAILAVHERDDDPAQIDRLAADLGY
LPLALSQAAAYLVDQDLDCATYRDLLADRAQPLADLAPDDSGLPD
>D3D3D8/375-641
AAVLGELAAGLISPVVALTGMSGAGKSTIARKAVASQEVRDRFADGLIWITAGPGADPTA
CQSWVLEACGDRFPVRTVDEGVRRLRKVLGGRRVLIVADDVWTAEQVRALDAQVPGSGLL
VTSRNAEVVSPGDGLVCVGPLEPDEARNLLARYAGVDADALPDEADDVVTTCGRLALALA
IAGGMVADGHQWSYVLARLRNADLRRLRHRFRDYPDHPTLFAALDTSLAALPPADRVRFL
ELSVFEGQTPVPHEAVRRLWRLSGSLD
>D3D136/462-718
REDLLAELHARLVEGATTVLPEALHGTGGVGKTQLVTEYIYRHLGDFDVIWWIPAEQSVQ
IGIALAELGEQLKIDLDVERSIAVRQVVEALRIGEPYGRWLLVFDNADDPAIRTDYFPAS
PTGRVLVTSRNPGWGNVSHPLEVTVFRREESIKLLRSRGGPLDDDSADQVADVLGDLPLA
VEQAAAWRADTGMSAQEYLRLLEEHRGDLLGTPPADYPVPVTAAWNISLDRLAVNNPAAL
ELLQVCAFFAPEPIPRQ
>D3CUZ8/272-539
RDRDIERLRAMLAGARVVSIVGPGGLGKTRLANVLARDSALPVVRMVELVGVAAPEDLLG
EVGSALGVRDSVSSRTVLTPPQRADLRARIAQQLSSGPSLLVLDNCEHLVAAVAELVAFL
VATTADLRVLTTSRSPLSISGERVYPLGALSSADALELFRERAVSARPGVRLDDAAVAAV
VARLDGLPLAVELAAARVRVMSVEEIDRRLADRFALLRGGDRSAPDRHRTLLAVIDWSWN
LLAEPERQALRWLAVFQDGFTLDTAEEI
>D3D0J1/302-533
DRDLAGRLHEAAAAGQTAVLTQVVAGLGGVGKTQLAADLARRLWQDGHGELDLLVWVSAL
SRDAVVSRYVQAALRLGLGREDDDPNKLVENFLSWLENTRQRWLVVLDDLAEPAHIRGLW
PPDRPTGRVVVTTRRRDATLLARGSAVELGVFTPAEARQYITRKLAAHPLLADDVDRVVD
LLGALPLAISHAAAYMVDLEMPCTSYADQFVDRSRKLQEMFPEEAANFDEYA
>D3CVM9/28-170
QERADLRQRLGRFRLVTLVGVGGVGKTRLALRTAAEVRGEFPDGVWFVELAQLRDPDLLA
YQLAATLGCSAPADRTVLSTLSRHLNERRLLLVLDNCEHLADACADLVDRLLRACPRLRV
LVTSRESLRVEGECTYLVTPFSM
>D3D353/34-288
RESQLAELRRRIASDEGEVTAVLPHALQGLGGVGKTHLAIEYAYRYQAHYDLIWWIPADQ
PVLVRSTLASLAPRLGLSDGGLLRIDDSVAAVLEALRRGTPYRRWLLIFDNADQPELIRG
LMPHGPGHVLVTSRNRRWQSIVDTIEVDVFDRRESLEFLHRRVPGIAEIDANRLAEALGD
LPLALEQAGALQFETGMDVREYLQLLKDASSKLLAENPPADYSRPVAAAWSLSVAQLREQ
APFALELLRRCAFFG
>D3CWG3/1-248
MGGAGKSTLAVALIHDPVVCSLFPDGIVWERIGSEPDIAGILRHLLNAFGDSSHVSDVDV
GVRRLRSLLSGAQCLIVLDDVWDIAIVEALRLPSSVRVLVTSRTRAAWYADAAAYELAML
DEHTARRMLAKYAGLTVDELPAVADEIVERCDGLALALALVGSMFRLGSRWQYIVERLRG
ATLHKLTARFPGYLYQNLFAALDVSVQALPPSESDRFRDLVVFAGRGPVPIDIVALLWGA
TGGLDSLD
>D3CRG6/213-423
RPELAERLVGMLTAESEEPLRVVALHGGPGFGKTTLAKLVCHRDDVRAAFPGGLLWTSLG
QDHGQLTGKINALVVRLAQELGRGSQEPLPSEPEQAGMRLGELLDALPPERRTLLVIDDV
WSAAQLGPFLSGGSRCTRLVTTRDQRTLTGDPSVVWIDMMRQAEARELLVGAAPDLPATV
ADRLARLCGSWPLLLDLAAKNLRRRVTGGAP
>D3D107/227-440

LLAPAAHGARPVTVVSVSGMGGIGKTALALRAAADADTRGYFPGGVYVTDLQGYGAGPDR
GALGGRRSSGPAHVYGPLLALLRPDDPVPQSPMEQHGAYHQALTVLAERGQPVLLVLDNV
ADPGQIVDLLPRQQAHRVLVTTRDLGLGDFYGLPLAGLDVGAGVEVVDEVLRRRRPADRR
ATRQSESARRLVDLCWGLPLALRISAALLAEVPS
>C7Q9I5/276-495
REDQLAALAKVLTAPEPGNPPAVVVVTGPGGIGKTSFAVRLGQRLRPDFPDGQVFVRLGG
LRAPRRPTELVAEVLRALGVAEIPGDPDRRTALLRSTLADRRVLLVLDDATDPAQIRRLL
PASAPAAVVVTSRRRLPGLAGHVPVELGRLSAEQAAAMVGNIIGADRTAAEPEALARLVE
ACGGLPIALRICGARLALRRGRSIASLVARLEAVGKRLEG
>C7QC27/157-360
AALGAALDERTRADDRPRVCAIGGMGGIGKTWLALHWANQHLDRFPDGQLYVDLRGFDPA
GQPMAPTAAVRGFLEALGVAPSAIPAARDARFGLYRSLTAGRRMLILLDNARDTAQVTPL
LPGSDACTVLITSRPQLAGLIATHGVSSVALDVLPRDEARRLLSRYLGRARLDAEPQAAD
ALLACCAGLPLAVGIVAARATIHS
>C7Q5E3/295-561
RENLTRVLAARISTTVGQSVAVCALSGLGGVGKTALAIHLAHSVREEFPDGQLYVDLRGG
DPTPADPAPVLAAFLRGLGISEGETAPGLEERAAAYRSALAGRRVLIVLDNARDAAQVRP
LLPGAPGCAVIVTSRPKLTGLAGATFADLDVLDPGEAMNMFTRIVGEERLGMEHTAAIDV
VSLCGYLPLAVRIAAARLASRPRWRIGSLAARLSDERRRLGELAVGDLAVRAAFELGYHQ
LSPAQADVFRRLSQLNSADVSAAAAAA
>C7PW80/364-622
ALAERLGAELSAGAAGTTPTVLAIAGMGGVGKSTLALHVAHRARPAFPDGQLYADLRGTG
ATPVPPQAVLEDFLHALGVATEQIPEGTAARSSLFRTLLDGRRLLVVLDDAANAAQVRPL
LPGAGGCAVLVTSRARLVALPKSAQVWLDVFDDEEALGLLGRVAGPERPHAEPEAARLLV
DACGRLPLAVRIVAARLAARPAWTVASLAGRLADERSRLRELRIGELAVAPAFEVGYQQL
TAAQAQAFRLLGAVEAAEI
>C7PW86/39-285
PAHTAALFVVTGSGGTGKTALAVRAARLAGDSFPDAHLYADLGGYTGSPTPVPDVLRRFL
RALGAAGPLPEDRDELVGMFRTALAGQRALVVLDDAADEAQVRPLLPTGPDALAIVTSRN
WLGGLAGALPLRLGPMPDAEAAAMLARVLGARWTGTEDVDGLLALCGHLPLAIRIAAARL
LSRPQMTVDDLCDQLRDERRRLKRLTAGDITVHGVLTSSYDALEPGDRKVFRRLALLRGF
DFCTEAA
>C7QC35/300-572
RDHELERIKTLLSHDPPGSVPAPGVCVIAGAGGTGKSALAVQVAHAVRDRFPDGQLYLDL
RGADRHPVDPGHALAEFIRALGDGGSALPEGVADRSAVFRTMLADRRVLILLDDAGDVQQ
VRPLLPADPRCCVIITSRSRLPGLEDCARLELGSLSPQDGASLFGKVVGDERPQSEPAAV
ARIVELCGGLPLAIRIAGSRLAVRRTWRLESLAARLGDTARRLDELRTDDLQVRATLDMS
YQHLTGDQARAFRLLAVPDVDSLSVWHAAVHLD
>C7PXB0/490-707
RDAMLEKLRDGIVGSSSAVILPLALHGLGGVGKTQLALEYAHRFKADYDLVWWIEAEQPD
FIDTSLADLAIRLGLRGGDNVPEAAEAAREALRRGTPYNRWLVVYDNALEPEVLTPYLPD
LPGDGTGHILITSRIQSWSRVANSLEVDVFTPEESVRHLTRAVPGLAQADAAAIAELVDN
LPLAVESAASWLATTGTPVATYLESLAEETTRVLSLGR
>C7QFS8/80-331
SAAGGSVLIVGTAGVGKTSLAVGFAHRIRNDFPDGQLFVNLRGYDPGPPLSSAAALERFL
RALGVPPGAIPVDLEERAELYRTLLADHRILIVLDNAATVGQVRPLLPGEPGCLVLVTTR
GRLSGLSAREGTHRISLGLLDQAEAVALVEETTAPYRDPDPPEQVSELVALCARLPLALR
IVAERAAVRPHMPLRELISDLRRESSLWDALSAEDSADPDADAVRSVFAWSYRALPPAAA
KAFRRLGLHPGP

>C7QFS4/283-538
ILAAVAADLRPGKRGATFLIDGLAGIGKTTLALQAAHLLRSHCPDGALQLNLHSHDPYLP
PLDQRQALTQLLDAIGTPYRELARADTVPALGALWRKRTSGRRLLILLDDVLDTAQIELL
IPATAGTIVLITSRRRLTGTPGNRQYTLGPLPDSAATALLSHITDRTLPEDDDLASFTQC
CGGLALAITVAAGHLRSRPVWTVGDLVSRLSTTSQSLADDPLTSPIHTAFAMSYQTLSPT
LRDLLRYIAAHPGPDI
>C7QJ36/118-379
RADEVQAVCQTLTGQLGHPRAAGICVISGMPGIGKTALARCVAHLLADRFPDGCLCLDLQ
GFAPGVTPLTDFDALGLVLAMLGVPDEQIPAGRQARVVRYHREIAGRRLLLLLDDAVCAQ
QISCLMPPTAGCSVLVTSRNRLTALDDAHRVVLPVLPPEQAAELFCAVAEYRGGDQTSID
GILAACGGVPLALRIVAARCRPDSGLTPADLAARLAHPRSRLTHLADNERAVSDAFGASL
DLLPSAQRRLFLLLGLHPAKPL
>C7Q011/124-389
REAELAAMLEPAAGGSQTVVISAINGTAGVGKTQLAVHAAHELVRSGRFADAQLYVNLRG
FDPEVPPMDPAAVLEGFLRALNVPARHIPAALDERAAMFRDRLQDKQAIIVLDNAADDRQ
VRDLIPAAPSCLVLITSRRSLAGIDGARLVDLDVFDPDESLALFTSVIGPDRIAAEKEAA
EQLIAATGGLPLAVALVASRLRARPAWSLAEAAHALHSRRLDGVQLGARSLRPLIDLSFQ
GLSAPAKAVAQAIGVHPGTDYTVPAL
>C7Q847/404-677
VWSRQLLDLMQPGEAMPLAVLSGIGGAGKSTLAVHTAHLAAAKFPDGQLFAALRGTDREP
ADPGGVLGGFLRALGTDPGAVPDTVRERSELFRSTLAGRRVLIVLDDVRDAEQIRPLLPG
TPGCAVLATSRSRLTGVPGARLLELGAFHPDEALALFRAVAGPDRVTGSEPAVRRAVAVC
GHLPLAVRILASRLAARPHWTAETLAGRLCDEARRLDELRAGDLAVEATFRLGYEHLRPD
QAHAFRLLAVPDGPDIGVEAVAALLCCPQQEAED
>C7Q4V1/340-603
TAALAVLEEFLAAAGEGDQPLIALVGTAGVGKTALAVHWAHRIAYRYPDGCLYVNLRGFD
PSQEPVTPEQAIRGFLQALGLPRQELPALFADQVGRYRSLAAERRLLIVLDNARDAEQVR
ELLPGNPACLTLVTSRDRLTGLVAVDGARPLRLDTLPADEAFDLLARRLGGRHAAEEPDA
IREIAELCARLPLALNIAAARIATNPHLPIEMFVQELREAGATLRTLDAGDRAASVRTVF
SWSYRQLGGPAARLFRLLGVHPGP
>C7PXN7/333-536
RDHELTRLARLLTSESDAPRAAAVTGPAGVGKTSLALIWAHEHAGAFPDGQLFVDLHGYD
HSEAESPEGVLERFLLALGIPGHQIPPGLPKREDLFRSAMAERRMLLVLDNARDYRQISP
LLPGSAHTRTLITSRIRLGSLVADTGALPVPLDVLPLEESVEVLTRIVGAESVAAAPQSA
RDLARLCGGLPLALRISAVRLLEE
>C7QEQ7/49-311
TDRHAVHERLDQLADREVGMVQLGGVAGVGKSSTAMRYLRSRSERYRGGVYYADLGGGRA
GRGVSVAEALDGWLVAKGVLSSEVPPSLAARSALFQRITADEPVAVLIDDPASAAQVAAL
CPTSQGSLIMVTGHHELAGIRTSHSAEFLRVPMLDHAFALELLTDLVGEDRVQAEQAAFD
QLAAFSEGHPLMLRVIAAELSRGRWDSAGELAQRLADTRSRLRASDQIMSSGGDYSVNAA
LELSVRGLPESGRALLRALASHP
>C7Q7X2/299-508
REDAVHRIADASADRCGGAFAVVIDGMAGVGKSALAIHAAHRLADRFPDGQLYADMLGTS
ADPAQPDAVLAVFLRLLGIAPDEVPGTCVERAGLYRSFLAERRVLVVLDDVRDAKQMAPL
IPASPGSAVLATTRDRQVGVAGALHVSLSTLTQDELRTLLTRIVGADRIAAEPEAVEEIL
TACAGLPLALRAVGGRIAARPTWTIADFAS
>C7QI68/338-588
PVVHTLDGPAALPVTVLYGQGGVGKTTLAVHVAHRLAESYPDGQLYARLRDGDQSVAPAD
ILERFLRSLGVAGPSLADGLEERAEMYRNLLGDRRVLVVLDDAMTEHQVQPLLPGGSGCS

VIVTSRRRLTGVPAAVRLEVGTFSDDSAVALLSRVADPARIHAEPEAAAQLCRLCGHLPL
ALRIVAARLAARPHWSVRALVDRLIDESRQLDELNHEGVGMRASISVTYAGLSADARRLF
RRLALFGGPDF
>C7QEQ5/77-332
VAGTQGPVMVVSGTAGVGKTSLALHWAHRNRDRFPDGQLHANLHGFDQGSPAAPADILGH
FIEALGVPAAMTPSDTDARAALFRSLVADKRLLIVLDNAADSEQVRPLLPGAAGCVTLIT
SRSRLSALIAREGAARITADVFAEHDAVMLLTTAIGAQRADDEADVAELAGLCARLPLAL
RIAAERAAARPRMPLTDLIADLRNESSMWDALSSEDAAEADAVRTVFAWSYRALREPASR
MFRLLGLHPTAEFAVG
>C7Q4W3/274-479
RRAELKALDGVLTGSQAASAVLISAISGTAGIGKTTTVVYWAHHAARQFPDGQLYVNLRG
FDPTGPPMKPEEAIRGFLDVFAVPKERIPHGLDAQAALYRSLLAGRRMLVVLDNARDADH
VRPLLPGSPGCLVLVTSRSRLTGLVVGHGATPITLGLLDDAEAEHLLSRYLGAERVAAEP
DAVRVLIQRCARLPLALAVAAARALM
>C7PWI4/317-578
DAATLDAADRDGAAIAVIAGMAGIGKTTLAVQWAHRAASRFSDGQLYINLRGFDPGGAPV
APDHAIRVFLEAFGIPPARIPTTAQARAGLYRSLVADRRVLILLDNARDVEQVRPLLPGT
PACLVLVTSRNRLTGLVTAEGAHWIPLDLPDPPQARELLARRLGSDVVAEQPEAIAELVE
LTARLPLALSVAGARLAMNPLLPVSAFLASLRTTRSRLTVLNGGDITTDLRAVFSWSYQQ
LAPAAARMFRLVSLYPGPDVSL
>C7PY74/334-592
RAAELSRLTRLLGSDNDSPYIAVVTGPAGVGKTALALLWAHRQADAFPDGQLFVDLHGYD
RIEAENADSVLERFLLALGIPGHDIPSGLPKREDLFRSAVANRRMLLVLDNARDYQQISP
LLPGSALSRTVITSRARMGSLVADTGALTVQLGVLPLDESVEVLARIVGPDAVAGAPDAS
RELARLCAGLPLALRISAVRLLEESTAGIAGLAAELTSEEHRLSALDLLDDGRTVSQALE
HSHRGLSAEQGRLFRLLSR
>C7QDR7/286-549
AELAAMDEAAASGEVLVVSGLAGVGKTALTTHWAHQAAPRYPDGQVFVGLHGFDPHSVPM
TAHTACSILLESLGLATSEIPADPDARTALYRTVVAGRRLLLVLDDAWDAAQIRPLIPGT
AGSQVVVTSRNRLAGLVAADGARPILLAPLDNGRSMELLARRSGIRPRPDEPADTAAAEA
LAAACAGLPLALTIAAARLQLDPDLSWSALTERLHDRRGALSTLDVGEASGSLRAVFSMS
YQRLSRSAAALFRLLGIHPGPDIA
>C7QIP3/136-394
RLQLDQLRDMLRRTANDGRGAPVLALYGMGGVGKTSLAVQFAEEVRSEYPGGVLHLSLSA
GGTSGLDAPDPATALGRLLAALGVRAGEVPPEEEARRRLYFDRLVGRRVLVVLDDAKSAA
QVRPLLARVRGCATVITSRRPLGLLEAVPFTVGGLDDDSLRELLIRHVGARRVEEEPDAA
RQIIRACGGLPLALRLVAARLAADGHRTLAELADRLSLDEFRSGDVRVRDAIEVSYAELS
ARSRQAFRLLTLFPSISLR
>C7PX42/184-396
RDKELKALHARLWDAPRGQARVAVTGLGGVGKSQIAVEYMYRYQSEYDAVWFVRATRTAI
ARQDVVNVGAELGAPSADDLQAGIDGALRALLEDARTRRWLVVIDDARSAAAVKDLLPSF
PGQHGHVIITSTDENWTDIAAELPIQPMSAEESFRYLRSRLPETGDEDLQALVGYCDGLP
IAMDLAAAQVRGAAQSVRAFLQSASERGVDQYQ
>C7QCA1/279-545
RHEPIAALDQWIATAGRTAGTVAVVSGPPGVGKTALAVHFAHTIADRFPDGQIYLNLRGF
DPLEPPVAAATAMRDVLVALGMPSGAVPTEPAALLALYRSRLSGGRMLLVLDNARDAAQV
RSLIPAGPGSIVVVTSRDRLFGLIAVDGGVALPLDALTPAESAQLLAGRLGATTVREHRA
AAEEMAQLCSHLPLALTIAAARAAAHPTIPLANWVSELRRADRRLDMLTTGDRDSNVRTV
FSSSYHALSTSAGTVFRFLSLHPGPEI

>C7QJ39/109-371
RNAEFDQVRSELVERPDAVGAASVCVISALAGVGKTALAIHSAHRLAGRFPDGCLFLDLR
GFTPGHAPLSSFEALGALLALLDVPVATIHSTEPARSAQFQAETAGARLLLVLDNAADAH
QVRLLLPSAPGCRVLVTSRNRLVALDEAVHLDLRPLAEVDAAALFRMVSAGGRVSQSTVD
GIVARCAGVPLALRIAAARCAPGGAFDPELLAAELSRAEDFIGQLDDGERSVRAVFDASF
SLLPRELRQVLALLGTRLLTVFD
>C7Q118/110-352
AEVETVSRLARAGQAVAIDGMGGVGKTALAVKVAHLLTPEFPDCQLYVDLHGFTPGRDPV
EPTEALLGLLRALGVPGGRIPDDLAGRSAQWRSELARQRAIVILDNAADADHVRALLPGA
GRNTVLITSRVRMVGLDGVQPLSLAPLGPADAADLFTVALGPGTAADPETVAELMRRYGG
LPLSIRVAAARLRHRPAWSVADLLESPPPSDEAGLGKVIDASLARLGGDQRQMFLLLGLY
PGT
>C7Q771/129-378
ELATLVTTPGVWVVDGMAGVGKSTLAVRAARALAEAERRRTLYLHLHGHTSHRAPLAPEA
ALTALLSAIGVPDKRIPEDPDLRAALWRGEAARRTPVVVLDDAVDSAQVRPLLPGAADAV
VLVTSRRRLVSLEGARSLTLAVPSLPECRELVDVIAGPERRAAEPEAVDAIIEACGRLPL
AVQLCAARLRHRPAWSAAFLAERLRDEERRRRELAADGGGVGAALALSVAHLRETERAGF
ALLGSLPGLD
>C7QA79/156-407
NTSAPQTVVVSAIEGMAGIGKTQLVIRAAHELVRAGRFADVQLYVNLRGFDAEAAPADPS
DVLDSFLRQLEVPARRIPEDRDERAAMFRDRIAGRDALLVLDNAADEAQVADLIPADPRC
LVLLTSRRNLAGLDGARLFRLDVFPPRDALELLARVVGQERVAAEPEAAAEVVRLCGLLP
LAVSLAAARLRSRPAWSLERLAAHLRDSGLDAVRAGSRELRPVFDLSYRDLPAATARAFR
LLSLHPGRGFTS
>C7QK63/313-577
SGSGAHRLRIVLIDGMAGSGKTALAVHAAHFLQESHPDGQLYLDLHGFTPERDSVDTHEA
LGILLGALGISGSDVPVDPEARIARWRTATVNRRMLLVFDDAESAAQIRGLLPSSSESTV
LITSRVRIKGIDGARAVSLGVLSPAESLSLLERVLGRERVANESEAAMRLADLCGHLPLA
LRISAARLASRDHWTIARLASRLSNESRKLVELAVEDRSIRACIKSSLEALDQEHLEYLR
YFCLHPGDDVEIHAAAALTGLDVYG
>D6TBP7/147-360
REEELALLARWIGEDRCRVVSVLGMGGIGKSALAISVMHQVARQFEVVIWRSLRDSPTCA
TLVESCFQVLDPQAQSAVPDALEERLRLLMEQLRARRVLLVLDNAETLLEEGTGTGRIRA
GALGYAQLLRLMGETRHQSCLLLTSREKPADLGPLEGKRSPVRALRLAGLDDLAGAQVLT
EKEVVGSPQDLVRLVKVYQGNPLALKIVAQTIVE
>D6TX29/62-331
REGLLVRLHEQLHAGQATALSQAISGLGGVGKTQLAVEYAYRYQEEYRYILWVHAESQES
LISSYVSLAHLLNLPQRNEQNQNVSVQAVKTWLQNNDNWLLVFDNADEPKVLPAFVPASP
KGHCLYTTRASTLGSLARSLTVECFTDEQGALFLLHRAKLIKSDASLEQVSPQNHNLAFQ
LTRELGGLPLALDQAGAYIDEAEVGLAEYLELYHQHRSELLQRRGFSIDYPETVATTWLI
SFKRVEERNAAAADLLRFCAFLAPDTIPEE
>D6TS07/106-379
RDALLQRLKELLQPISPHRSTRLVLQGLPGVGKTTLAMELAAHEEIRSWYPDGILWAGLG
PRGQALTVLNRWGNLLSLNEQECRSLHTLQDWTQRLHALIGGRRLLFIIDDVWDITEATY
CIIGNTHSACILTTRLPEIAYTLGGEVIQVPELNEQESLQLLRHMIPTLHNTAQTEIQEL
AHAVGGLPLALTLIGSYLLIQTRHQQSRRTQQALSRLKQVEHRLSLTQPGIRISRLGSDA
LHETDVSLQAVIEMSISILEKTEREALYALALFP
>D6TBB9/149-360
AELALLCQWIGEERCRVVSVLGMGGIGKSALAITVMHQVARHFDVVIWRSLRDSPTCATL

VQSCLQILDSQAQPEVRDPLEECLRRLMEQLRARRVLLVLDNVEVLLEEGTSTGRMHADA
QEYARLLRLLGETRHQSCLLLTSREKPADLVPLEGNRSPVRALRLAGLDDLAGTQILTEK
EVVGSQQNLVRLVEVYQGNPLALKIVAQTIVE
>D6U459/168-455
DLAEIKQLLVHTQFVTLTGAGGCGKTRLAQRVGKDMRDQYVDGVWLVELATLTDAALLPG
LIATTLAHQEQPGHPLLETLVAVLQTRRMLLILDNCEHLVEACASVAEHLHAVCPSLALL
ITSREALNVAGETVWQVAPLTVPARSLQPVTAAQIRHTEAVQLFLARVRLLLPHFELTDQ
NALVIASICQQLDGLPLALELAAARMNLLSLEQLAERLEDRFHLLTAGRRTALPRHQTLR
ATLDWSYESLSQQERRLFRRLSVFAGDCTLEAMEAICAWPATETDPAE
>D6TUK2/104-328
RQTLLRSLKTCLCDQELPACAALYGLPGIGKTSLAITMARDQEITAHFCDGTLWASLGPS
PDLVALLRHWGRVLGINAIFLERSENPETLAMLLRATIGPRRLLLLLDDAWTLEDAQTLQ
VGGPHCAYLLTTRIPALAYYFANIVSFQVPELSLEESLDLLASFIPGIREERYQSLRPLV
SATGGLPLALCLLGRYLQAQANGRQPRRLQHALEYLSHASARLQL
>D6TZB0/26-312
RLDEIEAGCELLKRDTIRLLTLTGPGGVGKTRLALRIAEYLSPTFAHGACFVPLAAINDV
DLVLPAILQSLSLKENGSQSILELLQESLRPLHVLLVLDNFEQVIGATPEIELLLRSCPH
LQLLVTSRARLHIQGEYEYIVAPLVLPDALEPISVSSLESYPAVALFCERAREAHFDFTL
TPENQNDIIELCRRLDGLPLALELAAAHAKLLSPRALLERLKRRQPLPSTRLRNIPARQR
TLRDTIEWSYELLNDEERTLFRRLGVFCASSTIEAIEGLYALLSEPE
>D6TDE0/107-363
FSAAKTGAVSRSVALRGIGGIGKTQTAVEYAYRYFQTYRAVFWLAAETPESLITSLVGLA
GLLELPEQREQEQGRVVVAVLHWLNTHRDWLMIVDNVSSVDVVKPLLPTAREGSLLFTTR
LPELNTLAFPLSLQPLSCAEGTRFLLQRTGFNSPSSLAQPQAEWALEEIVTALGGLPLAL
EQAGAYIQKTQCSLAEFLQLFRDFPLEVLQEQDTAADHPFSVARTFALSFELLQHESPLA
AEMLTTCCLLAPDAIPE
>D6TNY5/473-757
RTAQRQRHNPHHIAVTGSAGIGKSALALEVVRRHQDKFPGGVVGVSLEGGKTFHEALIEI
IHYLHIPTRLQSAVDSEHRARLVMGTLRSLASRELPCLLLLDAFEEVGDRNELETWLQFI
CALPAEVTVLVTSQSNPENMLALENAHCRWYEYRVGKMTDSDQLQLFMELAQASGLDQRI
HLDDPYQQEILREICSLLDGYPLGAELIFGTARTIEGQVYTPEAATRSLEEVRDELRSSP
LAGILTVLEVSSRRLSASARLLLAYLSAFKLPFSREQIALLFQDT
>D6THX8/115-343
RDDLLQSIRDQLSDATRGPAFVALNGLPGVGKTSLAVALVHDPSLQQTFSDGILWAGLGP
HPEVLEHLSRWGTFLHVDEASLPNKESCESWAQAIRFAIGQRRILIVIDDVWQVEDALSC
QIGGPNCAFLLTTRFPHIALHFAAGNTTPVHELAENDGLALLARLAPYVVQDETESALAL
VRSVGGLPLALTLIGKYLRIHSYGRQPRRINAALTRLRDARERLQISEP
>D6TKB9/97-353
EALLEQLHTSFASGQALALTQSYTLSGLGGIGKTQVALEYAYRYHQHYAATFWLAAESSE
TLTSSFLSIAQTLRLPERHEQDQHKIIASVLQWLNSHKNWLLIFDNVEDIGVLKPFLPTT
RQGSFLFSTRLHTLGHLAQALELSRLPRDEGIAFLLHRTHIRPANSPMTQAAASEVELAA
TIVDLVDGLPLALDQAGAYIEATQCSLEDFLHLFQTYPIQLLDERDAHAQHPHSVTTTFR
LAFEQVQRNKNSAAIEI
>D6U340/160-375
RESELVELERWIVDERCRLVALLSRGGYGKTALSVKLTQQIAHHFDVVIWRSLQNAPPLE
RLLADYLTFLSEQHTTDLPESTGERMTLLLEYLRTARCLLALDNVETLFQERSRAGTYRA
GYEAYGQFFQRVGQTAHQSCLLLTSREKPKELAPLEGDHAPVRTMTLAGLELEACRQILK
DSNLAGTEGEYRSLADRSAGNPLALKMVAATIRDLF
>D6TQG2/158-268

RNQELATLEQWVAQDHCRIAAVLGIGGVGKTTLTSMLKERLRETFDFVYWRTLQNALPLE
EFLVDCIRFLSGLEQVELPEDVGELIKQLGSYLRAHRCLLILDNVETILAS
>D6TYJ5/110-384
REALLERLHTRLQASPTQIYALCGLGGIGKTQTALEYAYRYYSAYQSILWLPAETAEMLA
RRCQGLARRLGTTTILEEQAPEASLELIRIWLETHESWLLILDNVEQVAVIEPLLPSLFN
GHILLTTRAQATGPLAQHIDLKQMSDEESALLLLRRAKYLAVSASLHTIKEQDVHEARAI
GKLLGGLPLALDQAGAYIEETGCSLADYLARYQRHRKDLLARRGNNPMEHPLSIAGTISL
CIERLEQEQQAAAELLYCCAFLHREGITEELLSLL
>D6U864/151-361
LDLLSAWVVGERCQVVSVLGQGGIGKSALATKVMHRVAEHFEVVIWRSLRDVPSCEALLD
SCLQVLAPQALSDASMSPEKRQDLLLECLRTRRVLLVYDNLESFLEEGEGIGSMRAGYEG
FSRVLRRVATTEHQSCLLLTSREKPGDLVPMEGNRSPVRALRLARLDIEACQQLLEEKEV
TGTTAEQEQLIEAYAGNPLALKIVAQAIVEL
>D6U731/84-303
REDLLTTLANIFRTSKGAFTSLALSGLGGIGKTQIALEYAFRFVADYKYVFWARAAASES
MIADYVTFATLLSLPEKDEQDQLKIVEAVKQWLITHDRWLLILDNADDLVMVRSFLPTSP
TGHILLTTRAEVTAPLAQRIEINTLSIDTAALFLLQRASILDIGQPLNQASPADQALARR
LSTELGGLALALDQAGAYILEEGISLSGYLQKYEASRAQL
>D6U1H0/88-304
REEILTTLHTSLYVEQIAFTQAYALQGLGGVGKTQIALEYAYRYAQEYQAVFWIGAETAE
SIIFGLLRIAESLQVPERDGKDQQQVITAVHHWFTTHDRWLLICDNVEDLGMLKRFLPPA
RQGAILFTTRLRTLGTSAEGINLLPMEHEEGILFLLRRAKVVKAQATSEQVRQLSTQMSL
QYAAGVELVTATGGLPLALDQAGAYLEETQCGLPAYL
>D6TBW8/89-318
DDILASLHTSLSIDQEAVYTQVYALHGLGGVGKTQIALEYAYRSARKYRAIFWVRAETAE
SIAFDVRHVADVLQLPEQDDKDRQRVIMAVRNWLTLHDQWLLICDNVEDLNVLDHVLPAV
RHGATLLTTRLQTLGTRGRSIHVSPMEHEEGMLLLLRRAKVLEAEATSEQIRQLAARLPS
QYEAATGLFTVMGGLPLALDQAGAYLEETQCGLSAYLELFSTRRTLLLQR
>D6TMN9/109-393
RQKLIDELIGLICNGGTAYAITGLPGVGKTALVTTLAQHPHIQRNFKDGILWVGLGRQPR
ILELMSRWGRVLGLNELETGKLTTPDAWAKYVHDYIGMRRMLIIIDDAWELTDASIFRLG
GRNCVHLLTTRFPALGVSFAYNNVVRLQELTDEDSHKLLEQLVPEIVPHNVQDIQELIKV
VGGLPLALTLIGHYLYLQAYSGQQRRLHSAIDRLRLHATERLNIANPHLVWHHSSTLPVG
TPYSLQIAIDISLSSLTEQARQALYALSIFPPKPHSFSEEAATTI
>D6TI77/97-378
RSREIAEVKQLLDTSRLLTLTGTGGTGKTQLALRVAAEEASAYADGVCFVDLTPLSDYTL
VAKAIASVLGVVERPMEPLPDTLKRALAQRELLLLIDNFEHVIKAAPLVSKLLTASSRLK
VLVTSREPLRIAGEQEYLIPPLSLPLAEAPSVESLTKSEAGLLFLRRAQMTLPRFTLNEV
TAPAIGRICIRLDGLPLAIELAAARCKLFTPQALLERLEGTREGSPLRLLAGGSRDAPPR
QRTLRDSIEWSYNLLDEDEKHLLARLAVFRGGCSLEAIERIC
>D6U002/35-321
REREIASAVTLLREPAMRLLTITGPGGVGKTCLALQVVAELEPAFSDGVRRIALASTSSS
ELFIPTLAQSLGLVEFDEMPLLERIKRYLREKHVLLLLDNFEQVIAAAPLITELLATCPA
LKILVTSREVLHLRAEHEFALPPLALPDLKLTTDLEALAHNAAVALFVQRARSVKTDFQL
TAENAQTIAEICTRVDGLPLGIELAAARIKILPPGKLLARLEHRLQVLTHGARDLPRRQQ
TLRATLTWSYELLDPQEQKLFRSLSAFVGGCTLEAVEHLCQAIDADE
>D6TLW5/120-394
RDEELADIKRRLFTGGNVALTALNGLPGVGKTTLSIALAHDRDVRAHFRDGILWAGLGPK
PNILGLLSRWGTLLGISATEMASLSDVHAWARAIHNAIGTRYMLLVIDDAWSTEEALAFK

VGGSNCAHLLTTRYPEIASFIALDGAKKIQALNDEESMTLLRLLAPGVVDREVQKAQDLV
HAVGGLPLALTLMGNYLRKQTYSGQTRRITAALERLSNAEERLQIQEPHGPVEAHPSLTT
DIQLSLHSVISVTDQQLSPATRSAFYALSVFPARP
>D6TUE9/14-301
REQEIAEISALLDNPSCRLLTLVGPGGIGKTRLAMEVATHKRASFSDGVYFVPLAALDQA
DELFTAIAEAMPFRFQQDQRSPREQFFAYLSEKQAQCLLLVLDNVEQLLHGVDLISDILA
VTTNLKILVTSRETLNLQEEWVRQIGGLTYPRQAEGDPLEEYSAIQLFLDRARHIRGDFD
LAEVRKSVVDICRLVEGMPLAIELAAGWLKTLQPADIAQEIQHNLNLLATRSRNLPERHR
SIRFVFDHSWQLMTEHERNVFQRISIFRGGFTREAAQVVAGASLDTLA
>D6TSL9/88-325
REEILHHLHERLNREHTTALTQSWAISGLGGIGKTQIALEYAYQYRQDYRYIFWVSAATR
ESLFADIVTIVDQLQLPERNEQDQKKVVAAIKQWFASHQEWLLILDNADDITIVSDFIPT
KRSGHMLLTTRAQALGALAQRIDVATMGMAEGTLFLLRRAKVVPPDMLLDQVEEETLAAA
ETIVTEMDFLPLALDQAGAYIEEVGCNLSAYLELYRTHRKELLQRRGHISTDHPEPVA
>D6TK87/165-377
REVEVVKLTQWIVQERCRLIAILGMGGIGKSTLVSLLGQRLASQFEAVLWCSVRDAPSCE
ELVADCISFFSETPPTAFPSSLEQRINQLVARLQARRCLLVLDNLETLLVSGNLESGYLP
GYEGYGRLVGRLAESEHQSCVLLTSREKPREIEPLEGARGHVRSLRLQGLDEQAAHELLA
DKELNGTFSAWQHLVASYGGNPLALKIVAQGVS
>D6U7N6/83-366
REQEIHAVSSQLRMENTRLVTLTGPGGVGKTRLSLEIARTLQDAFPRSIFFVSLAPLRER
EQILLAIASAFGVHEGGKETLTERLEAYLNERRCLLLLDNFEHLLKGADLVAELLATAPQ
LKILVTSREPLHLYGEQEIEVLPLPLPRQAENAYVPREESAALRLFVERAQAVKPSFTLT
QENYATIAEICQRLDGLPLALELAAARIKILSPQALLTRLSSRLTLLVGGPRNLPQRQQT
LRNALNWSYDLLTEAERRAFRRLGSLIGTWDLGAAAACMAISSE
>D6TKC5/57-329
RASALKEMHSRLRSKDTDMLALCGPAGVGKTQLTQEYANHYKKEYKDILWFNARNRSTFF
ASCNLIAFEADITSYEDKNQSALIKDLRAWLEKQHDWLLIADDITDLELLQELLPAKSQG
HVLLTTREPLPEDVASTLAVEALTPEDGALLLLRRASRIVLGDPLGKALPEDRELALQIA
RDLAGLPLTLNLAGAYIKESKLSLADFQQMYQKALQASVAVSQELAAFSLALFAVFSLTF
FSIEALHPAAQALLRFCAFLAPNDIPERLFQDE
>D6TEE5/106-400
RQKEIQHIAECLCSLQGGTYALTGLPGVGKTALTIALAHHPQVRQHFRDGILWVGLGPTP
RVIDSLSRWGSLLGLNASEVQTLTTADAWAKYLHQRIGNRRLLLILDDAWDLAEAVIFHL
GGSQCTHLLTTRFPSLAYAFAREQVTILHELTETDSLALLHQLVPQAFEEHLPELRVLAQ
TVGGLPLALLLLGHTLQVQALSGPPRRLSQALHELNQNMYARLQMAEPLPSWKEIPGYTA
GSSISLQLAIAMSVRQLPDAVQEALRALAVFPPKPYSFSEVAALAVCNTSVQILD
>D6TK04/124-336
RDVEQELLRGWILEERCRIVAFLGMGGAGKTTFARVIVEQLKSAFSYVHWFSLQNAPTPE
TYMRACLQHLGALTPTETLSFEASLARLLQCCAEKRCLLILDNMESILQGGEQAGRYLTG
YEAYGQLLTHLGTHPHQSCLLLTSREKPKEITRLEGGSARVRSLVLPALGVAESQQLLQE
KELTGSDAEWEALLQLYAGNPLALKLVSASIRE
>D6TRS4/108-391
EALLSQFVSQLCGGKGVFALTGLPGVGKTSFAYALASHPEVRQHFVDGILWASLGQEPHV
QEELNRWAHLLDIPLGGMRGLRTSQDWVRMLREYIGERRMLLIVDDAWTLAEAALFQVGG
SRCVYLVTTRFPAIALSLGREQTTRLEELPEEEGLKLLHHLVPPLKQCALEDLRTLTNAV
GGLPLALTLLGRHLYVHAHSGPPRRLQKAILSLQEDINERFQLGDPGPIRKASAQSSVGA
TQSLYAAISISVQDLPTKARKALRALAVFPPKPHSFSEESALAV
>D6TD49/168-452

RDQEAHEVAELLRATHVRLLTLTGTGGIGKTRLSLQVATELLDTFADGVYLVLLATVTVP
RQVPIFIANALGLGEAGEGALVERLYAFLKEKRLLLILDNFEQVIAAAPQLVELLARCPQ
VKILMTSRAVLHVVGEQEYPVLPLPLPVQDQVDTQVLAQSAAVQLFVQRAQAVQPGFRLT
AENAATIAAIARRLDGLPLAIELAAARMKVFSPQALLQRLEKRLPLLTSTTQDVPERQQT
LSNTLDWSYDLLAPREQRLFKRLSIFAGGCTLPMIEHLHASLEQA
>D6TLZ0/48-279
RANDLELLKQHLWEDGNASLTTLNGLPGIGKTTLAITLAYDEEVLESFADGILWAGLGPS
ANIPSILSHWGRLLGLSLTEQSADYTSWTRALHAAIGSRRLLIIIDDAWTLEDVLPFKIG
GPHCAHLVTTRFPAIAAHFAVNGPLTLPELNKEESMRLLRHLAPTVFRHDSHKESDLVQA
VGGLPLALTLMGNYLRKQASHGDTQHIQNAIQSLSNTRQRLELSEAYDANLE
>D6TCI4/111-336
RDVLLTELKQQLLAHKNWALYGLPGIGKTALAAALAADPEIQHRFHDGILWVGLGTEPNV
LSLLSHWATLLGISLTHVENVSNWESWGMAVHAAIGTRCMLIVIDDAWKAEEALAFQIGG
PNCAHLVTTRLPHIAVTFSGPGVVEVPQLEETDGFALLARFAPEITTQETESAYALVRSV
GALPLALTLIGKYLGSQACTRQPRRLHMAITQLQDAQQRLLLSVPA
>A9B5L8/99-375
RQTELATLQNYLNAEHIRMITLTGPGGVGKTRLALQIAQHSHKHFPDGVYFVDLAQASSL
ADIGLALSQTLNLPSSKYAWQRHIQLHYQQARILLILDNVEQLVSAAEHFRGLLDHTSQL
KLLLTSRTLLHCAGEYAIPLTPLRLPTAEASLNELKTNPAVQLFVQRAQTLNPQFALTNH
NAEAIKQLCWQVDGLPLALELAAARTRLLTPEALLAYLQPPLALLSTNDPTAPARHQSMY
NAINWSYQQISPKQQQLLRQLAIFQAGCTLDAIQAIV
>A9AWA4/120-398
RGAEQQRILQWLNNPTIRLISIVGLAGIGKTQLGLQCLHQFASQSEQQCVFVDLVTANDP
ESMVQAINKALEISESPDEHPLSLAISQLEQQPSCLLLDNCEQIQDASRVISLLLSEVPT
LKLIITSQVALRLSAEHVLQLTPLAVPNLLALPPLAELAQIEAMALLLARLQVHNPKLEL
TAKNALALAALCVRVDGVPLALELVAASGRLFDPEALLSELASHFLSMRRRGRDLPSRHY
SVTTALTWSYQQLDSASQRLFERLSVFVSGWTVEAALAV
>A9B5G3/49-337
REAQLYQLAQAMLRSDPTLITPTALATGMGGIGKSSLALEFAHRYGSYFAGGVFWLYAAT
NETLQASLDRCWDSLKPDEWRYEVKPETRLRVVRELFNQPIPRLLIFDNCEDPALLTAYR
PQASSGCQLLVTSRRSQWQGTNLITLDTLPPLESRQLLQQLAAQPNINNYLSDTDADQLA
ELVGHLPLALHLVGSSLKFYFRKPAAEYIAALQNQRIASLQAMVKPTSKLHQNTINNFWS
VRDTVEVSYGLLPAELGQACRRLLLMMAYCAPNVVIPWELLQAASGYDD
>A9B7Y1/100-236
RDSAIESICVQFQAQKARLVTIVGSPGVGKTRLAQAIGQQLLTHFSDGVFWISLDPIVNA
SLVPSLITRVLGIHENPNQSIEETIFNWLKNRHLLLILDNCEHIIELRQFVNQLLSYCPT
LSILATSREVLHLRWEQ
>A9B3C3/146-429
RENEQQRLLQLLRQPHIRLISVLGVGGVGKTRLVLQSAMQWLPIFHHGVHVVHLAALRDP
ELLIQTIIQSLAIKTTSQLPLLKQLKDFLYDKQLLLILDNFEQLLDAAPIVTDLLAHAPQ
LKLMTTSREALNVYAEQQFELMPFSVDCSQQLMLRQQPAVNLFLSRAQALQATIAYNDAE
LATIAQICQRLDGLALAIELAASRISLFSLTNLLERLSQRLSFINSGPRDLPARHQTLQA
VIEWSYVLLTPQEQALFVQLSVFVGSFDLPAASAICARAEQSAV
>A9B792/114-390
RDADVATINERLANEHVRLLTLVGPPGVGKTRLALQVAQQQLERFRHGVFVVALAPVTNP
QDVLSVIAQTLGIKETGIRRSFEDLKNFLYDRELLLVLDNFEQVLPAASSIDQLIQACYG
LKVLVTSREALRLRRERRFAVAPLAIATPVSDEPSATFSPAVALFIERAQAVNPDFEINE
TSLHDISAVCRQLDGLPLSIELIAARSMLLAPKAMLRHLEHQLTVLTSRSSDHPPRQRTL
RDAIRWSVDLLEPSDQQMFMHVGVFPQSCTLESLAAV

>A9AXT1/49-340
REDLLKQLAAAMASTTPTMIVPTLAITGMGGIGKTSLALEFAYRYGHYFAGGVYWINADY
TPIATTAATILPSVDRLWQKLFPQRDSSQISPEQRLNEIKSFFNSPIPRLLIFDNCEQQW
IFESYRPGPQSGCRVLMTSRNAVWSSSNVRAIAIDLLTPAESRQMLQKLAPRVTDAEADD
LAKLVGYLPLALHVMGVALGTLEPSLPVANYYQRVQQALVAELETSANTLQNLHRSPTNH
QWSVVATVRVSYGLLKRSYQDEAKLRHLLLLLACCAPNAPIPIDLLVRATEQ
>A9AX39/100-384
REELLAHASQLLLQSSTRLLSIVGPGGVGKTHFASQLAQQIQAHFSDGSFFIGLASLHDA
EQLPTLIAQTLEIPQPTHQSTLEQLIGAIDQRSILLMLDNLEHVMMVVPIISQLITQCNK
LKMLITSRFALKLHDEHLIDLPPLDVPQHPPEHQASAETNYSAVELFELRAKMVQPQFSL
TAQNRAIVGEICRRLDGLPLAIELAAARIRGLPPQAMLARLDRRLELFDQGNSDLPERHQ
TLRNLIAWSYTLLTPNEQTIFRTLSLFANHWTLGAAEYLCQAQIA
>A9AUP6/285-554
RTHELALLHSLIEQKQQRLVTLLGLGGIGKTSLALAYAHAAQAAFDAVWFVSFAGSAGES
LASDHHRLSATIATTLGLSQQLHTPQAALLHYLGQRSVLLVLDNLEHLVHEALHVQAILD
ACPHVVVLVTSREPLNIQAEQRVQLHGLALANADQAFAASAQLFLAHGTNATSQTLADPA
SMEWIDRICRMLDGNPLAIELAARWVHYLGLDEIATAIEQDMDFLQTSVRDLPDRHRSMR
AVFDGSWRLLSRHEQRVLSQASLFRGSWSL
>A9AZ49/18-303
IQEMAQLEQLLPDPTIRLVTLVGPGGAGKTRLALEAGRTVCEQFADGALFVSLAHVYDVD
LVLPTLAQAFNLSRLGNQSLLASVGAWLADQELLLILDNLEQVIDVAALLVQLLALAPKL
TLLVTSREVLNVQGEYRLPVPPLSLPPSSQPMAIEQLSEFSATRLFIERAKAARPHIPLN
ANDAQSIATICQRLDGLPLAIELVAAYTKVFTPQELLTRFSYSLDVPVGGARDLPNRQQT
LRQCIDWSYQRLTPEEQTVFCYLSVLVSSWTMAAVEQICAGQANVV
>A9B5D0/153-417
REDETATLVKWLQLDRCRLVAIIGLGGMGKTSLATRVAQQAQDDFKVIVWRSLQQGQQAN
DFLLECLHRIMPSPNSAYPSQFEHRLSVLIDYLRTTRCLLILDNIEAILQPQYPAGRYRE
GYEQYAQLFQAISERSHESCLILTSREKPYEFNRLEGVHTRSMVLTGLMRDDAQMLLDNQ
ELYGTPQLWQELIKHYTGNPLALKLVAQVIKTMFFGQIAEFLQHEELIFGDVRTILAQQF
ERLSDQEQELLYWLAIERHTVKLAE
>A9B6Z7/14-225
RNRVVAALVALFQEQAHRLVSLIGASGTGKTRLGLEATETIRDSFTDGCYFINLAPVDDA
VFVLPTIAHTLGVHETANQSLLDSVVNFLRGKRVLLILDNFEQVKRAADELKLLIERTDQ
AQFMVTSQVALGLAAEYEFSVPPLEVPEQSNLPSNQLLEYSAIALFVDRMQAIQPRFVLT
DTQAKAVVEICRLLHGLPLAIELIAAHSSALS
>A9B7X0/117-395
REREVETLTKLLQHPQHRLITVIGPPGVGKTRVAQAVGWASLGHFCDGIWYVEGIQCTTI
ADFWVDIANMLGRSANSSMTLIEQISALIGQKNSLLILDNCEHLSEINLGLAQLLAQCSG
LKILVTSRTSLKLRIEHLFWLHPFPTPDPQSSNLSAIWQNPAVQLFCQRAQASNHEWQIN
DSQAATIAQICQHLDGLPLVIELAAVRTQFVTPTTLLARLSNRLGILTNTMRDAPAHQST
LRRTLEWSYQLLDSNEQQIFARLSVFATDSDFEAIVAVC
>A9B5Z3/117-378
RQAVLQELLELCREYRLVTLHGIGGIGKTRLAIALASYIANAGFAQEVVFIDLRNEYTVH
DSWHALLNRWLGDPKADLTSYIQQSNRRTVLIIDNCEHIRAVAEMLLPLLNFGNISIITT
TQIALSINGERRFPVPALSLEEGILLFEQRARDLNRQVERRQTEQIVQRLAGHPLAIEIA
ASQLLLVSLNDILAMTNVEMLEIESLANGSASHRTLRQMVEYTFSLLHDDVQAACLRLAL
FEHHFSLAQATQAFKVNWRVAS
>A9B053/118-408
RDSLVRQVLQHLSDREGYWVIAIDGMGGIGKTALAMDLMQRFVAQQPEYRPIWISAEPQQ

GGGILPNQPLTFDSLITNLARQLALADVAQLSVEEKFQRLQQVLKHQPILLVLDNLETSG
EPQQVLLEKLRPLLQPSKVILTSRQRFKGEVFSVHLIGLEAEQAALFIRQDANEKGVQWL
QQASLDDLQPIIKATGGSPLAMKLVVSQLASLPLDVVLQHVQSVTQLNPGDEDAYVRFYM
FLFQRSWTLLELSAKQLLVSLARFVPSNGCDWRAMQQISALPAAELAHSID
>A9B5K7/19-306
TRARTLVIDLLLDAQARLVTLYGQSGAGKTRLSLEVAEQVGEIFRDGRYFVALAPVSQAQ
FVLPTIAATLGVEESQHEAILDSLILALADKQILLILDNFEQVAGAASELLELIRRAPNL
TCLITSRQALEVAGETAIMVPALQYPELGEDYQLEDLEQHSAIGLFVDRMRTRQPRFRLS
ADNAGALVDICRLVQGLPLAIELIAAHSASLTPQDLLFFVRNHLSMAALNPKQSARQAII
KPVLAWSVSMLPADAKDIFAQLGVFAGGATVETIKQVGLVETMPFESS
>A9B9A2/133-378
RDDMLLSIASTFFSCHSDAPIPTIGLVGMGGIGKTQLAVEFVYRYGSYFAGGIFWLSFAQ
PDSINTEVIDCYKYYCPQVIEDSAEKQIAYMKSLWMNPLPRLLVFDDCNEVDLLEKWRPQ
SGGCYVLVTSRRQQWPATVELSLLSVSTLDLAGSLDLLCLYRPDIREDQALGQKIAQKLA
NLPLAIHMAGSYLAHYKLKLEVYLAQLDQGITHESMKGRGTFHQPTNHESVNVTFNMALN
NLSNHE
>A9AWZ0/132-410
HAALTTLKTLLADDHRWITLIGAGGVGKTRLAMELAQQSIGSCCDEVHMLRFADVQRVED
VGLACIQQLQLVITEAQSLEQTLQQFFQQRRSLVIVDNLEHIPAAGLWLAQVFQAVPQQQ
VIATSRVRLNVPNEQLYEVAVLDCPAEKASFEQLNQSPAVQLCLDRMEAVRLVDRTNHQL
LDLVGQICRQVAGLPLAIELVAARTAEYSLETILAAITTDLEFIAEGPLDLDARQQTMAA
TIGWSYQLLPAQSQHIMQQLAVFRAGWDAAAAAAICGLE
>A9B062/124-396
NEQQTINRLFAKGQRLISILGFGGVGKTRLALAIAEIQQAHYRDGVCFCGLASISQPQLV
LATIAEALGVAIGPQQTPEKALQQFLAKRQILLILDNVEHVVEGVAAIGQLLREAPQLQI
LATSRVPLNLYGEYMLQLQPLVVPTKPIASQDLAETPAIALFIERAQSHAARFSLDDASL
EAIRQICSQLEGLPLALELAAAHTRVLSPQRLVQQLSNHVLGLKTSIRDLPERQRSLRNL
ISWSVDLLAPSQQQALQALAIWPAGWTLSSASF
>A9B8D1/137-388
IAHNAAQGAVINGIQGMGGVGKTELAIYLAHQLIPHFPDAQIVLNLYGSREQPLTIEQAL
GTVIALFKPNAKLPEQREKLLEIYHEVLADKRVLILADDARDLAHVQDLTPPVGSCLLVT
SRLRFAMPLMAQLHLTEFQEPEAIALLQQICPRLEAETAQQLAVACGYLPLALRISASIL
AQNPELAVAEYLIQLRDQQQQLAALEYPDDPQASVAASLALSYARLPSELQALARQLSLI
VADFSSAMGLAT
>D8FWF7/147-263
ALELTTLEQWIVRDRTRLIAILGISGIGKTAIALHLLPQIQHQFDYVIWRSLRTSPTLET
TLKNLIKFISDRADSDLPVSTDDKLSILIQYLRSHRCLIIILDDVQTILSSGQIAGNY
>D8FWA7/97-233
TKELTDLENLILKNRLAVLTGMGGIGKTALVGKVWEKQIKGKGEFDYVIQRSLRTSPPLP
ELLKDIVEFLSDGEQQEGGISQLMEYLRKQRCLLILDNVEAIMSIGTNAGTFRPEFQDYS
ELVKRVKSEQHKSCLLL
>D8G5X5/152-292
RTPQLSTLKQWILHENTRLVAILGITGIGKTAIAVHLVEQIKHEFDYIFWRSLSTSPPLK
TLQTNIVQFCRGGAPVPAPNSVPAPNSEERAMPTAGYAYAGGLPLLDYLRKYRCLLILDD
VQTLNSSGQLAGNYKPDHENY
>D8FZZ8/123-230
RTEELAALKQWILSDGCRLVALLGLGGTGKTTLAVKAAKLVQDEFDFIIWRSLRACPSVQ
DLLASLISIFSPQQKADLPLDVNARISRLVEYFRKHRCLLILDDFEMV
>D8FYJ9/144-270

RTPELQTLTNWIVQQHSRLIALTGISGIGKTSLAVQLVQQIKDEFDYIIWRTIDASHTLD
EFQHELIQLFSESEKLDSPATNPKRLPLIKYLQKYRTLIVLDDVHHLFSSGELAGKYKPE
HEEYRSF
>D8FZF7/113-333
RETLINELIQKLQGNCRILSIVGLTGIGKTSLAKQLAKQPQISQRLPEKEISFFQEDPKF
QVVAERILGAEANSPQLQQNTEHLVNAMVDRLKSQPILLILDMIEVILEPDGKSGHQFKD
ELFAKFLDRVVLADTMPSRIVLTSQDRPPIMAQGRYPNDRFFEQPLRGLSEAEAIALFRQ
WDVTVEGERTQEYLKRIFAVYEGHPLALSAIAGEVRESPYE
>D8G6G1/169-466
RYEEIAQLKQWIVGDRCRLVAIVGLGGIGKTYLSVKLAEQIQDCFQYVIWRSLRNAPPLQ
QILTSILQSIANSQEIDLAATVHEKISCAIDFFRNHRCLLILDNVETILQGGVYTGYYQE
GYEDYGEFFKCLGEGRHHSCLLLTTREKPKEISIMQGETLPIRCLRLQGLSTSAGLQLLR
LKGCYWTSEQEGFALVEQYAGHPLALKIIASTIRELFEGNISEFTQHNLLVIDEIRTLLE
EQFNRLTDLGKALFYWIAINLEPVSAEELYSDIYPLVSKPRLVETLKSLVQRSLIEQT
>D8G427/116-386
RTKEIDILKATLINSDIAAISIVGLPGIGKTALISQLIRQIHTENTPFTAVAWQSLQSAT
GKALPFDWTIDSLLFTLSNGDITTAVTTQDDSLKKIEKLIKIIKTKPCLIVFDRTETLLK
TKQAQTAGYFADDSAEYPWLFQQLLETEHQSKILFTSRESLAELPPTVTREIQLNGPNQD
AAITLLQSLNLTANQEELAELSHRYQGHPKALQLVAALIRDDDEFQGNVGKFLQDRDWLL
IRDIENLIDEMILRLSELEQTCLSRISVYQT
>D8G4G7/144-266
TPQLTTLETWILQDRSRLIALLGISGIGKTTLALRLIEQIKTNFDYVIYRSLQFSPTLNT
TLTNLLQIFSEKTDIPQNIETQISQLLDYLRKHRCLIILDDVQMLFSSGQLAGQYKSGYE
DYQ
>D8FWQ7/148-270
TSQLTTLENAIAHQNCRLLTITGMSGTGKSAIARHLIPQIQTHFDRIIWRSLRTSPPLET
TLKNLIQFLSNQNPPFASYQGEVGVLPENIDTQLEILIESLHTHRCLIILDDVQYILNSG
QLA
>D8G8D8/135-254
RTSELHTLKTSILTEKIQLITINGISGIGKTALVTQLVQQIKNEFQYVIWRSLETSPTLL
ELQTNLIEFSSQPTDINSPATNLKPLPLIKYLQKDRCLIILDDIHHLFSSGQLAGQYKPG
>D8G518/102-293
RENAIAHLSTRINQGSKIILIQAPGGVGKTTLAQEYLKSQGFDLILELLMAKEKENITLV
ESVIEEWLKRDFQEEPGREFGVTLGRLKRQLQTRRVGVLIDNLEPALDGQGKFIEPHRRY
VELLRVLADSSVQSLTLITSREPLAECVGVTNYPLPSLDEKAWQDFFSHRDIEIDAITLK
EMHKAYGGNALA
>D8FXG7/405-663
RERELETLHQQLQENERVAVSAIAGMGGIGKTELALQYALICKQTYQGGICWLRAKGLNV
GTQIVQFGRSRLQLQPPEDLDLTGQVGFCWTHWAAGKVLVILDDVTDYEVIKPYLPPAES
RFKVLMTTRLRLGKSVKQLEIDVLDESAALALLESLVGAERIQQQRDNAQKLCAWLGYLP
LGLELVGRYFDRKPDLSLAEMQQRLEKKRLDERSLSKPDADMTASLGVAAAFELSWDILD
EPTKQLGCLLSLFALTPIP
>D8FVA9/148-422
RAEELAQLEQWIVRDSSRLIAVVGLGGIGKTALLTKLTQQIQSYFDCVIGRSLHNAPPLE
SILADLLKSLSHPHEPELSEKVDERIVQLISYLRDRRCLLVLDDVQALLRSNDVYGRYLQ
GYEGYGALFRRIAEEQHQSCLVLISQEKLREISLLENPSRSIHSLKLEGLKPEDAQQILL
GKNLTGKQSWQELIQHYRGNPLHLNLIAATIENIFNRDVGEFVKLKTTVSNTGILDEPFQ
RLSALEKQVMQWLAKEDKALSFQQLRAKIEEIATS
>D8G596/129-391

```
RESELESLIQWIKNDNCRLVMLLGMGGIGKTALSVKLGEKLQDHFDFLIWRSLHNAPPLE
ELLPQIIQFLSQQQETAANLPKTISGQISRLCHYLREQRCLLIIDNLETLLKSGSPAGRY
REGYQDYGELFRQIAEISHQSCLVLTSREKPQGIASIEGESLPVRSWHLKGLSDSEAEKI
FIAKGVQSNTIQVSILTELYQGNPLALKIVATSIQELFVGNIAEFLTQGTTVFNGIRSLL
EQQFERLSPLEQQILYWLAINRE
>D8FUI2/154-230
RTTEIANLKQWILQENSRLVAILGMGGIGKTALASKLRNMIKDQFECLIWLNLSHAPLLA
DTLVNLIECLSDRPLSD
>Q7ND80/122-335
RTEELATLEGWLVRERCRLVALLGMGGIGKSTLAVKLARQTQEHFEHLVWRSLRNAPPLG
ELLGELIECLCDSPQVNLPAGTEGRLSKLMECLRRSRTLLILDNGESLLSGAEQTGTYRE
GCEAYAELFRQVGEVPHASCLILTSREKPKEVASLEGASLPVRSLRLGGLREDEGEAILE
AKGLSGSPDERRSLVECYRGNPLALKIVSTSIQE
>Q7NH14/122-355
REAEIAQCLEGLSPQERGWGVVIDGQGGLGKTSLALAVAHFCRREGCFGAYLWVSAKTTV
LTPRGVQKDTLAPTSLDALLDELGRLLGTEVHRLSSTAQKRQHLERTLQGTRALLVLDNL
ETLSAEDRQEVGEFLRRLPRDCKAIVTSRRRSGESAVTVRLDRLPWQTAQELFGRLAEGD
AAVRELVDTLGEVGTQKLYEAAGGSPLALRWTVRLMTEKDYSCARVLDLLGQAA
>Q7NJ67/119-229
REQELATLKQWIVQERSRLVAVLGMGGIGKTCLCAELLQQIHLEFEYVLWRSVRNGPLPK
ELLADLVRFFSHPQELELPASFEGQVYSLLAHLRKHRCLVVLDNIETFMSS
>Q7ND85/121-225
GELAILENWLVEERCRLVAVLGMGGIGKTALAVKLARQSEAHFERLIWRSLRNAPPLADL
LAELIVFAGDEQAPALPTGIEGRILRLLECLRRNRCLLVLDNAES
>Q7ND05/121-379
RAEELAALTSWIVHDKCRLVALLGMGGIGKTSLSVKLAETVKENFDYVIWRSLRNAPPLQ
EILVDVFQFVGLTQSSVSYRVPNVKQSQLIDLFRRHRCLLVFDNVETVMDSARKAGYYRE
GYEDYGELFKSVGSSAHNSCLVLTSREKPKDLGPLEGKTLPVRSLRLTGLQENDGLELLK
ERGLDVATGDSKELLRYYGGNPLAIKMVASTIRTLFGNNISNFVTQGTSIFGDVRDLLSQ
HFNRLSELERQVMYWLSIH
>Q7NKV0/322-598
RQQEILAAGELLTREEVRLVCFTGVGGTGKTRLALAVAQARRQNFAEGVWFVGLAALDDA
ALLPVAIAHALAVQESGAGTLLQRLVAFLRERQALLVLDNFEHITQAAGIVAELLSACPL
LKILITSRTRLRLYGEWEFGVPPLALPTSEGPLTYERICASEAVQLFAARAEAARRDFVL
TPAVAPAVAAICTHLDGLPLAIELAAARNARLDPPALLEQLDKRLPFLVDGPANVPNRHR
TLRQAIAWSYNLLPCNERQLFRRLGVFAGGWSVESAL
>Q7NCT8/18-121
RTDELATLERWLAREHCRLIAVVGIGGVGKTALSIRLARRVQPHFERVLWRSLRNAPPLS
DLLAELIELIAGSPQTSLPVTVEGRTTQLLAQLRRCRCLLMLDN
>Q7NH82/121-226
RERELATLESWLVEERCRLVAILGMGGMGKTSLSVKLAQQVQAYFEWVIWRSLADAPPMS
ELSAEWVALMGGGGVELPDTAGAQISLLLRYLRAHRCLLVLDNCET
>Q7NDZ0/89-350
RDAQLNKLHEILQKAGKAALSGMPGVGKTQLAIEYTYLCRDEYQHVFWVKAETDSELMAS
FVEIATLLNLPTLRQDDQSQIVKAVKRWLEQNDGWLLVADNADNLSMVQKHLPGAHQGRI
LFTTRDSATGQLQCIKVEKFKPEDGDGALLLLCRAKLLCQGANLDDAALEERELAKQIDR
EMDGLPLALDQAGAYIEEVPSSLAEYLQRYRQAGDQLRKRRGDLAPDHTSVTVTFNLAVK
QIELQNPAAAQLVRACAFLSPD
>Q7NLE9/120-233
```

TEELALLRRWIVEERCRVVSLLGMGGIGKTALAARFVEGQRPAFERVIWRSLRNAPPVEQ
ILSEVLRFLEEGPEAEAPSHLDGKLHRVLQLMQQQRCLLVLDNIETVLREGGWA
>B2JBW8/114-330
RTTELETLSQWIVRDRCRVVTLLGMGGMGKTALSVKLAEQLQGEFEYVIWRSLRHAPFFQ
DKLTDCIKILSHQQVTTLPSDPHEQITCLIEYLRKSRCLLILDNFDTLLQQGKQTGCYRE
GYESYGELLWRLGETQHQSCVLLTSREKPAEIAALEGDGLPVRTLALSGLEVADGQTILT
LKGLSGAEDETRQLVECYRGNPLALKIAATSIRDLYE
>B2J5X0/131-397
RQAELAQLQQWILVDQCRLVAVSGMGGIGKTSLSIKLAQQLQSDFKWVIWRSLRNAPPVQ
EILTELLKLLSNQQEIDYPETVEGKISRLLHYLRSQRCLVIFDNIETILQHNEKSKSSYI
KGYEYYGEIFRQIGEIRHQSCLVLTSRDQPPEVGLLEGASLPVRAFQLGGLKKTEAQELL
HLKGNFQGSTEEWNRLVEGYAGNPLALKIIATTIQNLFDGSISDFLNQQAFVFGNIRNLI
GQQFERLSESEKTVIYWLAIYRDPASF
>B2IU85/136-351
READMKKLLERLSSVHGAHMITVHGIGGVGKTALVLAAAYLCLKASNENSSDAPKFDAII
FTSAKQQELIPTNSILWRQQGQRNLRDIFREIANALDDPTILQSPPNDQFDRVRQILSKR
RTLLIVDNMETIEDRNEVIEFLYNLPICIKVIITSREQIALLPIRLRNLPQDDGLQLIRQ
QAEEKGISINDEDSKQLYDRTGGIPLAIVYSLGQLS
>B2J749/411-682
EILRLERAFRQNHVVLVQGMGGVGKTELVAGFARWLDDTQGRTSGMFFTSFEQGAGLSQV
INQIGRALGGERFSQMMPDKQEDVVQQYLQTNPCLLIWDNFEPVNGFPTGNEPLLSGEER
NKLKRFLKELRVGKAWVLITSRREEPWLDCGYSLINLRGLSEADAQELAAKILQTVGVER
KNLPAEYLELLKLLGGHPLSLRVVLPHLKTQTPVQLIEALRRGLDTFRGQEEEGREKSLT
VSLDYSFAKLSERTRQHLPFLALFSERVDAGW
>B2IYR9/140-253
RTQELAMLKKWLVNDSCRLVAVVGMGGIGKTTLCVHSIHQTQDEFEFVIWRDLRSAPSVS
KLLAELTQFLSPQSKNYFTEEDIDAQISDFIGLLRQHRCLIVLDTTTVIQQSSH
>B2JAJ0/130-367
RKDEINYFKKQITLFKERCIVFTGVGGIGKTLLASRLVEEITFDSLSSIYECIIWKTINH
SLSIDELVIDLNKIFQVDIEASENSFIDSISLLSKQLHLHRCLLVIDGFEKLLLADDFGK
RLQYEKFLLKLTEGKHESCIIITSQLPLKEFASVTTKLPIRSFKLEGLDVNAGMQILQEK
GLTGQECKRLIENYSGNPSSLEALADRINRFFEGSVKTFFRYQTTMIDPQLETMLHQQ
>B2J4D1/107-374
RTEELTTLEEWILNEHCRLVSLLGIGGIGKTTLSIKLAQQIQDNFEYVIWRSLREAPPIT
IILSNLIQFLSDEQETESNLPENFSDKVSRLLYYLQNHRCLVILDNAESILRSGSRAGLY
REGYEEYGELFRRIGEATHQSCLILTSREKPKEVALLEGQAIPVRSLPLSGLKIAEGQEI
LKLKGLSAVEDEWKVMIERYGGNPLALKIVATTIKDIFGGNVTEFLQQDTAVFGDIRDVL
EQQFERLSDLEKDIMYWLAINRESVTLS
>B2IVX2/106-312
RQAELQTLATWIGNEHCRFVGIFGLGGIGKTTLSVKLAGQIQSQFEYVIWRSLRQALPLN
TLLAEILPILMGSEATINSSISILMQQLRQKRCLLVFDNVESILQSGNRGGQYQQGFEGY
QQLFERICDELHQSCLIVTGREKPGGIAVRSGKKLPVRSLSLSGLSAIDGQQILMDKGLD
TTPQHQTLVNYFGGNPLALKIAATAIQ
>B2JBK1/159-419
QQELYHLKKLINNHRCISLAGVPGVGKTALSAKLLAELSLDSTQHFDFLVWKSVTHSVRL
QDLLSELIDLIQPDTSLNLPEYTQALITALIKHLQSHRCLLVLDGFEVLFKTPNLEQRLD
YKIFMRRLLEEEHKSCLLLTCRALPNEIYAMSKDDRSILYFRVDGLDTDAALNFLSDQGL
TDKNDCLDLIKTYRSNPSELATVAKKIKHFFSGSTEIFFQHKTTFITDEFQSMLDETFGE
SLEQTERYIMIYLAQRLSLDP

>B2J000/135-353
RAQEIMRLMELLDFQHTAHLISVDGIGGVGKTTLVVEVAYRCLEVSNNEHFAPSLPTFEA
IIFTSAKQNHLTSIGILPRLTRERTLWDICREIARVLDLSEIINLPLEEQFQPIREKLSQ
TKTLLIVDNLETIEDQQEVLSFLYDLPPTVKIIITTREQALFVPIRLGCLPKEDALRLIQ
HEAKEKSITLTTKESQQLFEGVSGIPAAIIYAVGQMAAG
>B2J7B5/116-269
RTEAIAHLNTLVNQGSKVIVIQGEGGLGKTTLAQQYLQTQGFDLVLELLMAKETQNITPP
ERVVEEWLKQDFDREPGVEFGVTLGRLKRELHNRRIGVLIDNLEPALDRQGGLIASHRNY
VELLRVLADARVQSVTLITSRDRLCEPGLNVNHY
>B2IZC8/105-358
RNQELAQLEEWLTSQNCKLVTINGIAGIGKTALALALVDRIQLKFDCLIWKSLQTSPSLI
SLLNSLLNSFEQGVVVQNIQQGTAQLIQQLQKHHCLLILDGLEAIFSQPEDLSYGQFIQQ
LSRERHQSCILISSREQPNNIETNTKIYHCLNLKGLPKTEAVELLQSRGFTGKELGLSVL
IQLYRGNPLVLKLVTPLIQSVFGGNVAAFLSQHTLIVGDRLRVILKQQFEQLSGLEQDIL
YWLAIWQEPVSFSR
>B2J557/110-325
RTEEIATLEQWILKERCQIVAILGMGGIGKTSLSVKLVEQIKENFEYIIWRSLQDAPPLN
TLLASLIQFLSDERETEAILPESTAPRLTRLLHYLREHRCLLILDNMESILRSGSRAGLY
RDGYEGYSELLKRLGETEQQSCLILTSREKPKEVASMEGGGLCVRSFLLKGLLANDGQEI
LKIKGISASDDELRTLVARYGGNALALKVVATTIQD
>B2IWV6/7-273
REEELQNLHQLMQDNKPVAIAAISGMGGVGKTELALQYAIQHRNTYNGGLCWLLAKTGDV
GIQVVQFARTQLDLKPPEDFDLLAQVQYCWRHWREGEVLLVLDDVSNYEQVKPYLPSSSS
RFKVLMTTRQKLGRIAKLSLDVLQPEAALELLKSLLKETPERIERELALANQLCKWLGYL
PLGVELVGRYLARKQDLSLTEMLRRLKNKGIDERSLSKSKSETDMTAQRGVLAAFELSWQ
ELEDSDKQLGCLLSLFATAPIPWKLVE
>E0U6S1/161-378
RSVELTTLKQWILNDNCRIVALQGICGIGKTALAGKLVQEIGQEFDQIIWRSLRSAPPIK
ILLDNWLNTLSPGLNCDNHCSLSNIIKLLREKRCLLVLDGIETILQTGELVGKYRESYQD
YADLFKSIAEQSHQSCLLVTGVEIPKEIILKTGAVSSVRSLVLWGLEKPHAYALLQSDAL
KDAESWSILIENYHGHPEALRIVAKLIGEVFNGNVHEF
>E0U714/100-298
RHEELDQLEQWIINDRCKLVVIVGLAGIGKTSLALALSDHIQQKFEKIIWKSLQTPRPLI
SLLDSLLNTLDNPTAAQIEQGIIQLLDYLQKHRCLLILDGWETTLEAEQKPTYHQFLQRL
SHARHQSCLLITSREKPSVIEIETKARCLSLKGLQAADALMLLKAAGFNGQELGLSALIK
IYRGNPLALKMVTPLIQSL
>E0UKG1/3-219
LVCSWIERLGDYSYKPTTVEVASIHLRTLLYEQSVLLVVDDVWNPEDVEPFRVGGAGCRV
LVTTREAKILGAERYDLDVMTPEQAEALLTHAWRAKLTEKQKKQAQTLAKTVGYLPLALE
LAAAQAADGVSLRELIEDLQGETARLETLDLYGAEDIPQEEKRKKYSLLASFNLSLGRLS
KDYLDKFAWLGIVPEDVSITSQMAATLWNCQPRKARE
>E0UGF2/111-387
RTKEIEELKKRIIEDKCPLVAIWGFRGLGKTTVAAKIAKDPNIDQEFEYIIWHDLRGVPS
LKTILRSIIKVISQQKQLDSFNTIEEGIDSLITYLNEHRCLIVFDDFESICQDKNDKRSE
DDSTDCQEYEQLFDKIAGEQHKSCLLLTCRKLPENWYNLNPDKIHSLELEGLNPEEARTI
LRKKNIFDLPEEEKELIEKCSGNPTFLEMVASNIWEYYQGDITEFLDSNVLSKIESVEQF
LEQEYDKSLSTLEQRVVQWLAILQEPVNEKELANICL
>E0UKE4/67-352
RKEDLERLHNLLQSGRNVCLVSGMGGVGKTELARFYAASEDCKTLFPGGVFYIDVRSGKD

LAADLVVLTKYHFKGEIQHDLSPQQKLMACWDVWKRQTDKVLLILDDLSGLGKNVRQYLP
PSDLVTVRLLMTSRETPDKPIPQELKLEVLLPSAARELLSSIIGRQRVDAELQETDKLCE
ELGYLPLALELVGYYLDDEDYQELSLSAMRVKLKEKVQHPSLSPEDVPMGMKATRGVQAA
FDLSWDELTPQAKHLACVLGAFGDAAIHWGLVTAIYKILQGESFSE
>E0UML3/105-373
RKDLITSLTEKLQGAFRFLFILGLTGIGKTALAERIILERQKTSSLTKSYDLKRVNFEAL
GNATDFMTVAIQWLESWNIPVASQQRNPEQVIWHLTQYLQHNTVLLLFDSFENLLVGNEE
EGWGNLADNWWEKFFLSLLSAQSCQSRIIITSQDLPLSLVEQRYNQYWYCHLLTGLDENE
QENLWEITGFNIDDNLEEKVLLMRLGKAYQGHPLVLRVISGEIWESFGGNVKAYWEDVKS
KIETVETALAEAENEAQKQMGSEDNWKLH
>E0UL31/189-439
IKLGKGGIGKTDLSLKLAQEIQDEFEYIVWRSLRNAPLLTTLIADLLKFLSNQQEINLEN
SLEEQFSKLVEHLRSHRCLLILDNVETILQTGPEGQEYRPGYGEYSELFQLIALSSHQSC
LILTSREKPENLARLAGQNKPVRFLELKGLDYLNGKKIFTDIENFTGTDAEWQKIIEFYD
GNPLALELAAHHIKDCYGNNISVFLQQGKPIFEHLNHLLNWHFERLKENEKEVLYWLAIN
RKPVSIIDLKE
>E0UCI1/52-330
RDEALKKLDKQLQESERIAISTLTGMGGIGKTELALQYALADRDKVEEERNYQAGICWIN
VSEETSNIEEKTNVGIQILDFAKNYLQIQIPEENELKLKDRIQVCWQKWRKGDTLIIFDN
VKYLEQIEDYLPPQSKKFKLIITTRNINLRNNFNLLELDLLDEESAIKLLGSFIGDKRIE
QEREDAEKLCNWLGYLPLSIELAARYLEDTGEKIENYYQKLQQQKLKDKSLERPKDQLMT
AKRGVIATFELSWQELSEDTKQLGCFLSLFEAQPIPWKF
>E0UMG8/165-427
REKELALLKEKFFQNTTPCKIIGILGIAGIGKTSLICKLIEQIHEEFRHVIYWSFLNPPL
PLEFLENTSNIISQHISFNAEDKFKIKLIKFIKILAKSRCLLFLDNIDAIIEDENYREYS
EILQAIGQSKHKSCLIITSRKIPQELESLAGNKTSTYFLELQGLDYQTGQQIFRDIDTFH
GSDDEWRFLIQQVCHGNPLFIKSIAHHIQRLHHNNLSDFLLKGNLLIEKIESILNSYFEQ
LSDLEKAIIFELAINRNPVCLSE
>E0UDA2/1-283
MLHEDLQRGNYVAITGMGGVGKTELASQYIHRYGEAYEGITWFNDRQKGLAAEVLEYFSL
QLNYEIPQQLRGKRLTLSEQVRECWGQYAASELPILLVFDDVTNLDNLREVVPSENRFRV
LVTTRLQYLDPNFISEVGLEVLSPEYEPGKALELLQALLGKKDRRIYREPEAAADICRCL
EYLPLGIILVGGYLVQDPDLSLEMMLGQLQERKLAEKSLQVRESLNQYQRGVRAAFALTW
EELDPLSQQVGMFLSLFSPKLIIWDLVVWVALRKWHEEIANEE
>E0UN54/144-284
RSQEITLLTQWIIQDHCRLVAILGMGGMGKTTLSVKLAQNLESHFDFVFWRSLRSAPDLS
ALLKEIVSFLSQQQEINGNLFDILHYLKSSRCLIIILDNWETLLDPHHGGQFLAKYQDYSE
LLEKIGTISHQSCLLITSREK
>E0UC73/105-234
NEKEFCQQILCQQKSAKAGQRIAIIGESSSGKTVLLHQITAWILDNTQDLPIWISMAELG
ETPLYEYLQQKWLKRVAKTQDELTRQWQNSLDELLKSGRVWLLLDGVEQMPLEQLEKLPV
TVNSQTRGLI
>E0ULM4/107-323
RIEELEILEKWLIQDRCRLVTILGIGGVGKTCLSVKLAEQVQNQFDCVIWRSLRDAPSLS
CILNDLIQFLSYEQESITDIADSPKEQITRLIKWLRASRCLVVLDNLESLLQDQTRAGQY
RDNYQEYANLFKRVGESDHQSCLLLTTREKPREIAFLEGEVMPVRTFRLRGVKNEEGTEI
LKAKGVHGLESQFAQLTKRYAGNALALKVVATTIQDL
>E0UHN6/148-324
LGQALIIYDDVQDYHEVIPYLPPSPSQFKVLLTSRNRPGVSIPRIELDVLSKDAAIKLLR

SLVGDARIEAELEQVQELCEKLGYLPLGLELVGKYLDEHRGLTIKELIKRLKKKGLDEKA
LNPSDTAFMTAQRGVKAAMELSWEDLSSEAKKLACFLSLFAASAIKWEWVEKCLLEV
>E0UMH5/111-401
RESVQSELLNKLKGDCRIMVITGITGIGKTALAYKLAEVLCQDGFPPEVPIDFDGFNQPD
NQQSQPTAQNFTSVAIELLHRVDEPVTGTQAKNPQQLLNKLVDNLVAHRRLIWLDSVENL
LEGKTEDGSNRFKDPLWRHFFQKIVEAEAFPSRIVITSQDKPADFKTFGQTNYWHIEAIT
GLNAEERIQLFQRLFEQAEVDLILDGTNRAHLKYIGEIYEGHPLALRLIAGDIINRLNGN
IVRYWEENKDRFRRVKRMIDEAEQADINDYYKTNILSNKSEKENLIKKILE
>A0DE94/145-297
INDSNAEVNEFLLDEKKTVLLIHGVAGSGKSTTAKKIEEFIWKLHNKNKKIRNQILIPVY
ISLPTLRDPLFQAVEEALHQDEYGFDELQIKECKEMLDKKVFRFLLIMDSYDEMKLENIQ
KNLYMNNKVKQNWSDPLVIFTTRSEIFTSSNYA
>P30429/133-440
REYHVDRVIKKLDEMCDLDSFFLFLHGRAGSGKSVIASQALSKSDQLIGINYDSIVWLKD
SGTAPKSTFDLFTDILLMLARVVSDTDDSHSITDFINRVLSRSEDDLLNFPSVEHVTSVV
LKRMICNALIDRPNTLFVFDDVVQEETIRWAQELRLRCLVTTRDVEISNAASQTCEFIEV
TSLEIDECYDFLEAYGMPMPVGEKEEDVLNKTIELSSGNPATLMMFFKSCEPKTFEKMAQ
LNNKLESRGLVGVECITPYSYKSLAMALQRCVEVLSDEDRSALAFAVVMPPGVDIPVKLW
SCVIPVDI
>D1M871/127-410
RNDCIQKVNNALCNLKSEGWVVLHGMAGCGKTVLAISALNNERMIHSCFPGGVFWINLGQ
INETRLLMKMQNLCLLLDLVHSNQRVPQNLEEARDRLRILFSHQHPRSLLVIDDLWSSSH
AKYFNIHVRTLVTTRYSAVADQLLEDIYKVPVMEQLHLGQSRQILSKWVNISIPDLPKEA
DLIIEECKGSPLALSMIGALLKIHKNRWNYYLTQLREQKTSKVRSKVAYEYPSLYDAIAV
SFNDLEEVIKKHYESMVVFEENSLIPSKTLEIYWELDEVETEDI
>A7SNU7/111-386
RESECQAVFTKLTTGPCQIVTITGPPGFGKSCVAIHVAHELFSLGICVYYLSLRNCSSLS
CMANQLLGALGIIASPKPVEQAMHCVMELTKDTVIVLDNAEDMLVEEVRDPFCNYVETIA
VRAMHVRLLVTSRLCMMFFTVESFSLVLTALEPEHAKTLLQKSDPNCLESRDADLLVEYC
GGVPLVLRTTAALLAKSINPKALIMEFQKSPVTALKNFNLMTLSSDHQIFQCLNISFCRL
SPELQIDLISLSLYPTTFVAADVYYLLTDHSELTTQ
>A7RLM8/119-400
RARDVHKVRTALRSLKEHDGWVILHGMAGCGKTVLAAEALRSPGLLDQLFPGGVFWIRIG
SVSQAKLLMQMQNLCVRLDHDHSRAPPRNLEEAKDRLRMLFAHQYPRSLLVLDDLWNAAD
VKYFDIRCRTLVTTRDASITDSVGGSKVKVRVSEGFSDKEALQILSLWTRTFDLPPEAIK
LVNLCNGSPLAISMIGAMLKEHPNRWKYYLNQFQKKKVSKLKTKFAYEYPSLSEAILMGI
NNLSEEMRTYYIDFALFDDGSKVPAQVLCILWDEELEIVEDL
>C3YQA7/235-502
TSEDVKRVRRAMGDRQFVIVLSGITGSGKTQVALSQAELFVERHPTAVVWRLDGHDKKSF
LTDKQNLLEKLKEKVPTDDSQVDASVAKALDNRKTPVLLILDDLDDGLFLSPDLLKPREE
SKVLLITHRKRLQQPANVSIPEDAYILINGFSNDEAKDFLRMQLPYHPPDELACLASKFS
GLPLGLAAARSYLRQTKTSVENYLTLLEKKETASKLEEKADEWMSQFYEKPELQQTGRNL
FAALRLAVSKLDQQTKSMFQLTGYMDQS
>C3YRD0/148-404
DQTEHVKVLCISMFYVVSITGLGGVGKTTFAHHACARQEREEYFIDLREVTSVENVHLKI
MREFGYPLIDCEPERVYEEIRSYSGQEAVIILDNADVLLEKRDTRDEFLQTLANFEKQLN
NQIQIFVTTRVHLIDDEHESPFSNLILCQLDVLREGGAELVQKLAGRNIITPDDAKILAD
RCGNWPLAIKVVCSQLRARTVTPGDMLSRLQTLQNIKEIREFMLQAYDSLPEPLRHVLVQ
ISVFAGPFTKEAAEKIL

>C3ZSR5/388-674
EDFKKLSEKMSGSSICIVHGQLGIGKSQAVMKFVEQFQAKTKSYVSWFIGHTDFSDSITN
KETGLSEETVSEKLLELVEELKDSGRPVDIKEGEKDVAKICKALKASKTKCLLVFDDVQD
MSVIPRQFLAPWPEMTVLITTMDETLATGSNFPEIPRLKMNGFSPSDVESFLTLGDNDSV
FTKTNKALLKELANQFANLPLGLSLAKSHILDSNTSVREYLHHLEQHKTAMGRENQHLSE
PERNIFAAVQLLLIHRMDEVPRQVLQIMAFLMPNNVPTAILKEAYWH
>C3YQ93/224-488
EDAKRVNDAMKGEQFVIVLSGITGSGKTQVALSQAELFVERHPTAVVWRLDGHDKKSFLT
DKQNLLEKLKEKVPTDDNQVDASVAKALDNRKTPVLLILDDLDDGLFLSPALLKPRDESK
VLLITHRKRLQQPANVSIPEDAYILINGFSNDEAKDFLRMQLPYHPPDELARLASKFSGL
PLGLAAARSYLRKTKTSVENYLTLLEKKETASKLEEKADKWMSQFYEKPELQQTGRNLFA
ALRLAVSKLDQQTKSMFQLTGYMDQ
>C3Z7M2/115-399
SQDVRKVNEAMGGAQSVIVLSGITGSGKTQPALYEAELFVERHPTAVVWKLDGQNKTRFL
TDKQNLLRALKKDVPEDDSQVDACVAKALDTRKTPVLLIVDDLDDGRLLSAELLKPRNDS
KVLLITHHKRLQQPINVFIPEDSFVNINGFAFEEETDEAVDFLRMKLQQHPTDQLQRLAQ
KFSGLPLGLVAARSYIEKSHTSVESYLTLLEKRKNALKLEEAANKAMSQFYEKPGQEEAG
RNLFAALRLAVDKLNTKAKSMFQLAAYMDNRRIPIVVLKDVVVDS
>C3ZGI6/334-599
EKVTNALKMFDGLENFDRRHFEIIWINGFPGVGKTTFAHHLCLQMEQRRREIFISLAKVT
SVDNMLTLIMREFGVTEINGDGNTQVEATVAHLIGTRDGEDFSLVLDDADSLLEAAESRR
RFVGFLAKVQDKQNPKVHVIVTSRSSVMSGPGLGDVPSLEATIVNLEELADHDGTDLVRR
LAGKSNITKEEAREVCKRCKGWPLAIRIACGAIRKDHLRPAEMIKQLENLTEIGDIRGFL
TDVVSTLSRELLATLKLVSVFAGPFT
>C3Y5Y4/139-414
SARELSAESLIRQTGDNVVGIWGMGGAGKTVLALMVAQKLEKSRQVFWVTMGEQRPRVLE
NLALLASGMANTPRSFSNISDAVSDIFQQTQYKDDLIVLDDVWNLNEAQVITEAVSGTCQ
VLVITRNKDVLVSLKVRHLFELKPLTKSDAEKFVLNAAGFSDASDHRDQELSRSVQRILK
ACQCNPLALSVVASSVTKPENVQQWIDTADELDKVGIDALGGDKERIVEASIDVSYRALS
SNIDQKRFVMCGIFPEDTDIPEETLVLLWCYPDSTC
>C3ZCE0/79-363
RVTEIARLVKELGKTSRNVQLVCLTGIAGMGKTGLAREACYRLQRRVFFINLRDKGNMQD
VFMTVAAALELRLDLQRGNLNDWIMVLKDWILACEEDVTIFLDNVDDLLEPGSSRMPNIN
RQNFLNLLSEVLKIKSKHVKILLTSRYKLKMEEYNGDVVEITLTRLDTEDGVRLLKKCAG
LVGEGMDDEECNNIVEGCGHSPLAIRVIARHLKDGRISPAEALSQLIAKGKKTQQALRPS
AVSEEENIFDCLRITFDALPDKLKNILVSLSVFQGSFTCDAANEI
>C3ZAU7/180-453
TSAGGGAQHVVGIWGMGGSGKTVLAVLVAQALAQEDRREVFWMTSGRRNSVLENLVDLFA
DMTCSYLPFTELSDIISAIRHHTHDKDYVIVLDDVRNVEHAEDIIDAMAGRCQVLVTSRD
VQVFRALQVSQVHNLKTFEKRESETLLLKKIGLSYALDPFDERRVMVDVILQACGGLPLA
ISVGAATIQNSEDVNVWRSTARALKSHGPSTKEGTGNRHVSNVLKSFAVSLNALTPALQR
RFVLCGVFPDNVDIPMDTLAMLWRTECTNLSEVE
>C3XVB7/175-459
REQELHDLSEGLQKNRCMVLSHAVAIRAHGGTGKTSLALQYAHNHKGEYPGGLFWVVATT
NRLTSDFANLANVMNHEEATKLKKAEEKIKFVQRELRTREGWLLILDNADEVSAHTQLEK
LLPPRHQLKGGHIIITTRCSYVEGLKVATNIELPSLTETESIRFLQRRTGRQDEENSDKV
AIKEIVSRFGGLPLLLEQAASYINKTRCTFDEYLKAIEIRGNIPLDGPSHGTNRVKRVEN
TFTLNFDEMPQVSRDIFHILAFCDPDEIPFEIFSVAWNSSSLADE
>C3ZSR6/375-628

SKGFTGSGKTEAANKIAESFQMNNPSNLTWFFGGTKDDHEGLSESSVDEDLRELAKELDQ
PTDRESALVDIVYDALNALAATGVKILLIFDDVQNISVVPREFLRNRKGAMKMIITTRDS
NLHQYNGHPFASHAMSRFTDDEVVAFMNPNGGAAVEDRSKIVELAQHFDGRPLGLSLARS
HILAVDTNVTRYLEKVRMEGTVDEQLSVPEQRVYNAVALSVNNRMGEKEGTMFKMAGFLM
QNNIPILILDHAYE
>C3YZV6/132-413
PVQSQQIRNALQQLKDEPGWVVVHGMGGSGKSVLASEVVREQELLEECFPGGVFWVSLGQ
VDKPMLLMKMQNLCSLLDKEHQAPPRNAEEAKDRLRLLFTHQHPRALLILDDVWTARDAK
MFDVRCRTMVTTRDSSVTDAVRGNISMVPIREGFSKEQSDEVLATWTETKVSDLPPEAGQ
IYTECGGHPLAICMIGALLKDHPNRWQYYLKLLQNKKMSKFKKTLPYEYQSLAEAIAISV
DHLDTEIRDLYQQFAVFDNDTLVPTQVLSILWAEEIEFVEDT
>C3ZCF4/25-304
RKGHLAAIKKNFQDGRQIGLISGMPGVGKTRLAKEVAFQMSLDCKNQNVKLQVNTFNVAK
FTSTNLILDTICASMLHKEHTTVSSKPEDLTTALQKLKLDEDYYLFICDNADTILEDGGL
RTELLDFISQIVEMTPNVLFLVTSRRRFRLAREYRLFFDVELSPLEPDEAKDLLAKIAPK
VPLQTHGEEIAKICGYLPLALVIAGVELQRGEDGYTPEELLELLRKNVLESPLSAESYSR
SEQVSHVLQSAIDKLTDVIKSHFASLNYIPGSFGASAAAA
>Q9I9H8/132-416
LLNLIREMLYQLRDTPGWVTVFGMAGSGKSVMAAEVVRDRSLIKECFPDGVHWLSVGQCE
RADLLVRMQSLCFRLEQCQSSDTSQRPPSTVEEAKERLRFLMLRRFPRSLLILDDVWDSS
SLRSFDIQCRVLLTTRNRALTDSVSGVRYEVPVENGLDEEKALEILALYVNGKMHKLPEQ
ARSIVSECKGSPLVVSLIGALLREFPDRWSYYLRQLQQKQFKRIRKSSSYDYEALDQAMD
ASLQVLEAEHQELYRDLSVMQKDIKVPAKVLSVLWGLELEEVEDV
>E7F2V1/133-417
LLNLIREMLYRLRDTPGWVTVFGMAGSGKSVMAAEVVRDRSLIKECFPDGVHWLSVGQCE
RADLLVRMQSLCFRLEQCQSSDTSQRPPSTVEEAKERLRFLMLRRFPRSLLILDDVWDSS
SLRSFDIQCRVLLTTRNRALTDSVSGVRYEVPVENGLDEEKALEILALYVNGKMHKLPEQ
ARSIVSECKGSPLVVSLIGALLREFPDRWSYYLRQLQQKQFKRIRKSSSYDYEALDQAMD
ASLQVLEAEHQELYRDLSVMQKDIKVPAKVLSVLWGLELEEVEDV
>F1QQ06/132-416
LLNLIREMLYRLRDTPGWVTVFGMAGSGKSVMAAEVVRDRSLIKECFPDGVHWLSVGQCE
RADLLVRMQSLCFRLEQCQSSDTSQRPPSTVEEAKERLRFLMLRRFPRSLLILDDVWDSS
SLRSFDIQCRVLLTTRNRALTDSVSGVRYEVPVENGLDEEKALEILALYVNGKMHKLPEQ
ARSIVSECKGSPLVVSLIGALLREFPDRWSYYLRQLQQKQFKRIRKSSSYDYEALDQAMD
ASLQVLEAEHQELYRDLSVMQKDIKVPAKVLSVLWGLELEEVEDV
>A5WVJ8/132-416
LLNLIREMLYRLRDTPGWVTVFGMAGSGKSVMAAEVVRDRSLIKECFPDGVHWLSVGQCE
RADLLVRMQSLCFRLEQCQSSDTSQRPPSTVEEAKERLRFLMLRRFPRSLLILDDVWDSS
SLRSFDIQCRVLLTTRNRALTDSVSGVRYEVPVENGLDEEKALEILALYVNGKMHKLPEQ
ARSIVSECKGSPLVVSLIGALLREFPDRWSYYLRQLQQKQFKRIRKSSSYDYEALDQAMD
ASLQVLEAEHQELYRDLSVMQKDIKVPAKVLSVLWGLELEEVEDV
>Q1JPV6/132-416
LLNLIREMLYQLRDTPGWVTVFGMAGSGKSVMAAEVVRDRSLIKECFPDGVHWLSVGQCE
RADLLVRMQSLCFRLEQCQSSDTSQRPPSTVEEAKERLRFLMLRRFPRSLLILDDVWDSS
SLRSFDIQCRVLLTTRNRALTDSVSGVRYEVPVENGLDEEKALEILALYVSGKMHKLPEQ
ARSIVSECKGSPLVVSLIGALLREFPDRWSYYLRQLQQKQFKRIRKSSSYDYEALDQAMD
ASLQVLEAEHQELYRDLSVMQKDIKVPAKVLSVLWGLELEEVEDV
>A5YM44/129-414
RKKLVNAIQQKLSKLKGEPGWVTIHGMAGCGKSVLAAEAVRDHSLLEGCFPGGVHWVSVG

KQDKSGLLMKLQNLCTRLDQDESFSQRLPLNIEEAKDRLRILMLRKHPRSLLILDDVWDS
WVLKAFDSQCQILLTTRDKSVTDSVMGPKYVVPVESSLGKEKGLEILSLFVNMKKADLPE
QAHSIIKECKGSPLVVSLIGALLRDFPNRWEYYLKQLQNKQFKRIRKSSSYDYEALDEAM
SISVEMLREDIKDYYTDLSILQKDVKVPTKVLCILWDMETEEVEDI
>O14727/129-414
RKKLVNAIQQKLSKLKGEPGWVTIHGMAGCGKSVLAAEAVRDHSLLEGCFPGGVHWVSVG
KQDKSGLLMKLQNLCTRLDQDESFSQRLPLNIEEAKDRLRILMLRKHPRSLLILDDVWDS
WVLKAFDSQCQILLTTRDKSVTDSVMGPKYVVPVESSLGKEKGLEILSLFVNMKKADLPE
QAHSIIKECKGSPLVVSLIGALLRDFPNRWEYYLKQLQNKQFKRIRKSSSYDYEALDEAM
SISVEMLREDIKDYYTDLSILQKDVKVPTKVLCILWDMETEEVEDI
>C9JLV4/129-414
RKKLVNAIQQKLSKLKGEPGWVTIHGMAGCGKSVLAAEAVRDHSLLEGCFPGGVHWVSVG
KQDKSGLLMKLQNLCTRLDQDESFSQRLPLNIEEAKDRLRILMLRKHPRSLLILDDVWDS
WVLKAFDSQCQILLTTRDKSVTDSVMGPKYVVPVESSLGKEKGLEILSLFVNMKKADLPE
QAHSIIKECKGSPLVVSLIGALLRDFPNRWEYYLKQLQNKQFKRIRKSSSYDYEALDEAM
SISVEMLREDIKDYYTDLSILQKDVKVPTKVLCILWDMETEEVEDI
>A7E2A2/118-403
RKKLVNAIQQKLSKLKGEPGWVTIHGMAGCGKSVLAAEAVRDHSLLEGCFPGGVHWVSVG
KQDKSGLLMKLQNLCTRLDQDESFSQRLPLNIEEAKDRLRILMLRKHPRSLLILDDVWDS
WVLKAFDSQCQILLTTRDKSVTDSVMGPKYVVPVESSLGKEKGLEILSLFVNMKKADLPE
QAHSIIKECKGSPLVVSLIGALLRDFPNRWEYYLKQLQNKQFKRIRKSSSYDYEALDEAM
SISVEMLREDIKDYYTDLSILQKDVKVPTKVLCILWDMETEEVEDI
>E1BS49/129-415
RPKLVDDIKQKLRSLGSDPGWVTVYGMAGCGKTVLTAEALRDHQLLRGCFPGGVHWISVG
KQDKAGLLIKLQNLCSRLEHDSTLPQRPPLNIEEAKDRLRLLMLRKYQSRSLLVLDDIWD
SWVLKAFDNQCQVLITSRDRSVTDAVSGNKYEIHVESGLTHEKGLEVLALFVNMKISELP
EQASCIVTECKGSPLVISLIGALLRDFPSRWEYYLKQLQNKQFKRIRKSSSYDYEALDEA
MSISVEQLSNDLKEYYKDLSILPKDVKVPTKVLCILWDMETEEVEDI
>F1P1P3/130-416
RPKLVDDIKQKLRSLGSDPGWVTVYGMAGCGKTVLTAEALRDHQLLRGCFPGGVHWISVG
KQDKAGLLIKLQNLCSRLEHDSTLPQRPPLNIEEAKDRLRLLMLRKYQSRSLLVLDDIWD
SWVLKAFDNQCQVLITSRDRSVTDAVSGNKYEIHVESGLTHEKGLEVLALFVNMKISELP
EQASCIVTECKGSPLVISLIGALLRDFPSRWEYYLKQLQNKQFKRIRKSSSYDYEALDEA
MSISVEQLSNDLKEYYKDLSILPKDVKVPTKVLCILWDMETEEVEDI
>E1BR73/129-415
RPKLVDDIKQKLRSLGSDPGWVTVYGMAGCGKTVLTAEALRDHQLLRGCFPGGVHWISVG
KQDKAGLLIKLQNLCSRLEHDSTLPQRPPLNIEEAKDRLRLLMLRKYQSRSLLVLDDIWD
SWVLKAFDNQCQVLITSRDRSVTDAVSGNKYEIHVESGLTHEKGLEVLALFVNMKISELP
EQASCIVTECKGSPLVISLIGALLRDFPSRWEYYLKQLQNKQFKRIRKSSSYDYEALDEA
MSISVEQLSNDLKEYYKDLSILPKDVKVPTKVLCILWDMETEEVEDI
>Q1WWG7/129-397
QPYLKLRQALLELRPAKNVLIDGVLGSGKTWVALDVCLSYKVQCKMDFKIFWLNLKNCNS
PETVLEMLQKLLYQIDPNWTSRSDHSSNIKLRIHSIQAELRRLLKSKPYENCLLVLLNVQ
NAKAWNAFNLSCKILLTTRFKQVTDFLSAATTTHISLDHHSMTLTPDEVKSLFLKYLDCR
PQDLPREVLTTNPRRLSIIAESIRDGLATWDNWKHVNCDKLTTIIESSLNVLEPAEYRKM
FDRLSVFPPSAHIPTILLSLIWFDVIKSD
>Q9U8R4/129-397
QPYLKLRQALLELRPAKNVLIDGVLGSGKTWVALDVCLSYKVQCKMDFKIFWLNLKNCNS
PETVLEMLQKLLYQIDPNWTSRSDHSSNIKLRIHSIQAELRRLLKSKPYENCLLVLLNVQ

NAKAWNAFNLSCKILLTTRFKQVTDFLSAATTTHISLDHHSMTLTPDEVKSLLLKYLDCR
PQDLPREVLTTNPRRLSIIAESIRDGLATWDNWKHVNCDKLTTIIESSLNVLEPAEYRKM
FDRLSVFPPSAHIPTILLSLIWFDVIKSD
>B5RJS4/129-397
QPYLKLRQALLELRPAKNVLIDGVLGSGKTWVALDVCLSYKVQCKMDFKIFWLNLKNCNS
PETVLEMLQKLLYQIDPNWTSRSDHSSNIKLRIHSIQAELRRLLKSKPYENCLLVLLNVQ
NAKAWNAFNLSCKILLTTRFKQVTDFLSAATTTHISLDHHSMTLTPDEVKSLFLKYLDCR
PQDLPREVLTTNPRRLSIIAESIRDGLATWDNWKHVNCDKLTTIIESSLNVLEPAEYRKM
FDRLSVFPPSAHIPTILLSLIWFDVIKSD
>Q7KLI1/129-397
QPYLKLRQALLELRPAKNVLIDGVLGSGKTWVALDVCLSYKVQCKMDFKIFWLNLKNCNS
PETVLEMLQKLLYQIDPNWTSRSDHSSNIKLRIHSIQAELRRLLKSKPYENCLLVLLNVQ
NAKAWNAFNLSCKILLTTRFKQVTDFLSAATTTHISLDHHSMTLTPDEVKSLLLKYLDCR
PQDLPREVLTTNPRRLSIIAESIRDGLATWDNWKHVNCDKLTTIIESSLNVLEPAEYRKM
FDRLSVFPPSAHIPTILLSLIWFDVIKSD
>E9H1Q9/132-421
RHVKVAQIRECLQGMDQGQFVVIHGMAGAGKSVLAAESIRDIDLLVNTFENNVYWLTVGQ
TGTDTLLTKMQALCERLDHTVLPPLSVDQATERLRRLMTDPSRKKSLIILDDVWRGEVVR
AFDFGCRILMTTRDASVTEVVNGRVLLVKVQDGFTQEESLSYLANCLNISEQDLPEQADQ
IYSESKGSPMVLSLLAPLLAERGDRQRIDVHRWDHYLGKLRDRKYTQIRRHGSYNYETIN
EAISISFDHLDEDTRHSLKDFVIFLDDVNIPAPVLQVLWKCSRYEMEERI
>C4Q6J8/207-368
LERLRRSLIRKQQRTSWGPDENGNLITSLLLIILDDVWDIEVGRVLSNLPGAFLVTSRDL
DILERVETPVDRLHLHNDLSEDEVASLLSMWTGYSMEQFIPSSKSSQNLKDSDISLPAIS
QMTYGLPFAVSLLGSLLYNQIHRLSDYVISVNGTGSKFLDWV
>B3SDU4/1-254
MAGSGKTTTVCQSVRQATKKGLFKSNGCYWMKIGNISNIELRQKLKTLGIRLNMEWKENI
QSMSEICAYLSSSLEANRRLSETLFIFDDIWKKDHYEYLSFAKKSISTSRFKYLENELDH
HCIRLSENLTYDEAIELLALLAVNDNDQTLRQNPVVKNVIDSCQGLPLAITLIGGLDLKT
DEEWNQAKDIIAKKSADIELAHYGFNLYGTLELSVNSLENENKQLFEQLAVFKRVSIPIQ
SIASLWNYNVIEAR
>B3S2Z5/28-301
RRDLIQRIDEQLHSPDVINQGMAALVGIGGVGKSCLALHYAHSNIQQYNIVAWLNAENEI
SLQNDMVKLGQRIVDSMSDGSHRIPPTLHTLQTLMEQYQKTPAIRHDLTSDSLKRQSRDT
QLLERIIEVSKDALDQQLIGPWLLIYDNVHRVKMVNKYIPTMRTGHIILTSRHRSWDDMI
EIDIFNRKTSIEFLHRVVEPHGHSHQPIENDDAWDNIADILGDLPLALAQAAQYIRRKKI
SITTYVDQFKSLYQNLWQQEAPSSSYKQYLSEND
>B3S2R7/142-412
AREKISKKLSGLLQIALKNRKCVVVHGEAGCGKTTAAYLTLFVNDLSLANKLHKGGVIWI
KLSNSNSAEILLQMQHLLTSISNDESRLPSTIEECKNQISCIIDDVLDKNFIKCFDFHCA
TLVTSRQPDIADNTYESPNLFEVEPFQLNELKEIILYDLESVSEKENCITTKMARILEYY
HNLPICLNSLRILNLTNSQEVNRHLLALGSKIDRAMGHNKTLQTTLDFIIRSLDETKRSH
YQLLVVFANSDKVPITAVSLLWSLDIKVIKK
>B3SB47/148-432
REKLAKDLVKALFSLRDKNGWIVIRGSEGSGKTVVANLAVRDERTITDNFPAGFIWFKIG
CTDINELLIKVQEFYLILNHNEKPPPSTIETVIESIRLLIADQYYGMLLILDDVRDGKIL
EYFDLPCTKLVTTRVSAISFQTTNYRINLSTEDGYTNSEAEKAFSAWFENDLTSITSDAS
SITEELKCAPMAIALIGGNILHDDIQFQNQFKMNGSSLENGYATKSVKSAKVEKIKFALG
KAFDTVDVELASKLRQLVTFQKCPKIPLNPLNVLWQLNDGQVENI

>B3SCE1/279-460
KIVNSRISDTLFIFDDIWIKDHYNYLSFAKKSIATSRFLYYENELTHYCIRLPEQLAFDE
AIELLSLHRHDRDTQVLRDNPIIKNVIDSCAGLPLAINLIGGLHLDTNEDWNKAKAIISN
KSKKVRLANYNFNLHGTLQLSVESIEENDRRLFESLVVFKRVNIPIESIASLWNCDEQDT
DD
>B3SCE4/141-292
KSIATSRFLFSEIELNHYCIHLPEKLTYDEAIELLALHRNDHDAQILRNNEVVKNVIESC
AGLPLAINLIGGLQLQADEAWKEAMAIIADKSMQIQLANYSFNLYGTLELSANSLKNGKK
ELFQDLAVFKPADIPTEAISTLWNLSHQKTNS
>B3SBJ1/129-411
REKALETLRNGLQSLQNQEGWVILHGMGGSGKTVLAAESLRSAKLIGTCFPGGVSWLTMG
KYHCLISQSMQNLCSRLDQEYQYLPTNIEAARDRLRMTIARRYAKMLLILDDVWSWRVLA
AFNLRCRTLVTTRDSTIADRVGGHKVLVPLQDGFTTDQSKTILASWIKQPVESLPAEASA
IIDECKSSPLAISMIGALLQRRPDRWGYYLELLKNRQVSRLRKSMSYQFDTLGEAISMSV
DNLDPELRQQYEQLAVFESNVKIPASVLAILWDKDEAFVEDDM
>B3SCE9/198-380
MKIGNSKLSDTLFIFDDIWKKDHYKYLSFAKKSIATSRFLFYENELSHRCIRLLERLTYD
EAINLIALLRHDQDDQILRRNPLVKKVIDSCAGLPLAISLIGGCRLQTDKEWSEAKAIIS
RKSGKIKLANYDFNLYGTFKLSFDVLDEKERKLLESLSVFKRVRIPIQSIASLWNCDEQD
ADD
>B3S8J3/29-316
REQFVHQIFRQLQDKNFIQGHLLVYGLPGTGKTIAVSQAVSLWSLSAPQEEDRPIFRVAY
WTKIGNVDNGNLFHILIQLCIQMGLSWEKRPRDLDEVTPCIEQFLQANSHKFEILFIFDD
IWDKSHLDYLTFAKKAVLTSRFNYPQWQENYHSFIKAPEQFTSDEVIQVLDGYIYNHNSQ
IVLESELMTRVIQSCQGLPLAISLLRSLTLETEEHWKEVIDIMESKPVEPKFPDYEFNLY
DFIMMCINNLEPEKNELFQLLGVFKRIPIPFRSIMCLWKRNEAEVYEM
>B3S5F3/164-299
NKLQGQFELQQSWQLLASFQPNIGLSTITANQVIASVIEYCRCYPLAITLLGGARLQSEE
EWKEALVFIQQNKEDSHGPSENYPASLCRVFQFVINRLPSPVGAYYRLLGAFKKSVKIPV
AAIATLWDQSQQLESI
>B3RXR5/167-385
EFVTKLRSLYIQITKNIQNICQLPDKVDEMVACLQAYLEGDKNMKKTLFIFDDVASKHHF
DKLKFAKNTISTSRFNHFRNKPTDSQHYIQLSELLSEGEAMQILAKYYSEEKLSQHRQLV
LQAINKCAGLPLAISLIGGLNCKTSNDWENAVNTIERRDMIDTQREYNFNLYTAFDLSIN
HLDHKERVLFQLLSVFKRVQIPKTSIMALWNEDKHYAEL
>B3S930/554-858
RQDLLQLMKQNLDVNNNCSVSMVSLVGFGGAGKSTLALQYAHNHIKSRSYDVIAWIRAET
LVSLQYGLVSLAIQMSQHLSHTSSTCPALHTLTSILAKFQADSPIKSSSCRSAILKINAS
TQEIESVVSNAIKILETFHNLKYLLVFDNAENKELVSEYFPLYGNGHIIVTSRNLSWQHQ
IDVSLFDRALSIEYLTSALSSKIQDLTNNAGLNALADELGDLPLALSQAANYMKQNRIVI
DKFVKKLVTQKIAALQVSASALNDYTQYSQKQFMNNGSQHQLAINYQENQEQMTVYMHRR
SVSTT
>B3S808/372-679
RDHYIHELHENLKRKHQCQITQASTIGMGGVGKSSVAILYAQQYQENYDLIRWFHAETEI
SLQYGMVILAKDIVDKGKNIDPIRYEKIARVRYWFNEYSKQHPEIFKTSDDGKMITVASE
SYNDKGGIIEKLVTATMEFIVSYCRYLLIYDNADSKDKVQRYLPKKTGNGHVVITSRHRE
WTDDIDYVEVEVFNREESTAYLLQRTKSDDKEAANAISELVGDLPLALAQVAACIRRTGL
KLTGYLSKLKNEEQNLWKGKFGPSYYRKYIETSTGQVKLSEPLTHITTWTLTLSQLLEQH
PIVEHIMI

>B3SCR6/97-367
RKDIVNKICDKLKNIDESPGHALIHGMAGSGKTIAVCQSVRQAVSNGCFKSNGCYWMKIG
NISNDELSQKLKVLGVSLGTVWNKNIKSIREVCEYLDNYLRENSKIANTLFIFDDVSKKD
HYKYFLSFAKKSIVTSRLTYDGIQLNFDCFNMPERLTPEESVELLALHHVAHSEVLRANP
IIKNVFESCAYLPLAISEIGGLNLSTNEEWNTAKNSITGVNTNLYGVLQSSIESLSESDR
ELFKLLAVFKRASIPIESISSVWSCDDKDAN
>B3SAE5/123-403
RNEFVNTICSELKRIHENSGHILVHGMAGSGKSVAVCQSVRRSIEADNYFKPHGCYWIKI
GNISDEKLLSILKNLSVRLGIVWVQSPQTLDEISAKIDRFLQDNSEIARTLFIFDDIWNE
RHYDYVAFAKKSVATSRFNYARNKISHILIPTREQFTDDEAMALIAKRREYDCHQTFLNS
DTVKNVIKSCAALPLAISIIAGLDLQSDEEWEEIRKRICSKDLMNDLPDYELNVFNTLDL
SVSKLNDSEKSLFRLLGVFRAVEIPLASVICLWKIEITKGI
>B3S5G6/136-338
DTTCDICNILQKIHQQGGHLIVHGRSGSDEMERSNALFVFDDLWDESYYQYLTFANQSVT
TSRFNISENNLTVIRIEAPAILTDHEAIDLLSKHGSFQDLEIKLENLLFAVVDKSEKNPF
ILDLMGALLRQNSEICDNLISETHGIDNIYNNTYGAKEIINLSIQQLDDNQRQRFRCLGV
FRPVQITISSVAAIWKCSQDEAI
>B3SE48/18-199
IANRRLSETLFIFDDIWKKDHYEYLSFAKKSISTSRFKYLENELDHHCIRLPENLTYDEA
IELLALLAGNDSDQILRQNQVIKNVIDSCQGLPLAISLIGRLGLKTDEEWNKAKDIIAEK
SAPTKLAHYDFNLYGTLELSVNSLDNENRRLFEQLAVFKRVSIPIQSVAALWDCEKSAAH
LH
>B3SBM0/286-557
KLYLQTISERFEGKKGQGVIVPLYGLGGVGKTYLALKTIADLSSNYSCIAWFNARSQESL
REQFLEFAIEHDILLQSSVSIDQKIEKIKGWFVNNPNSLLIFDDAESYTTLKLFLPRDVV
HVLVTSLNSKDWSNGISIDLMNKDDAVCLLKREAGIELSDQSQNEQIDILIETLDRLPLA
IAQAGVYICASSISVAHYCQLYESTRQSMLKDRTLYEKRDEHESVYVTFELSIQKVKASL
SSACDLLHYCSYFNPNNIPREILKKVPSVQGA
>B3SCD5/148-422
RNDFVNQTANAIMKIHARGGRLLVYGPSGTGKTVAVSNCLQYVIRQKQQFLPHGVYWITI
GKVDKQKLFEILSNLMIKLEMRPQTFPSLKQLNQAISNHLEALSDLSFTLFIFDEVEEPS
YMDYLTFAKNSVVISTQSSQAVIEKFDYNIQSQESFTQQEALQIIASFQENAKTQSWVND
DIVKQVVGKYRSSPQAIALVGGAQLKSLDEWKEVQALPEAKRTTKQSENTFTLFTHIMNK
LPERTRKLFTLLGACFRVKIPVAVITILWNLPADQ
>B3RR66/871-1132
RQKDLIKMKEKLFSPDPKKKEEEKKLLTLLGLGGVGKTYLALKFLHNAKDHHESIKCRLF
FNADDEETLTNSYIELARHLNILSGKLQIVSQIRKVKGWLCNNTGWIILFDNATMKYKDL
IKYIPQKGGYIVITTRCDSFDVNKIQIDIMTIEDSIELLEKEIGDFENVLEMNKDELNEL
VQLLDCLPLALVQAAGYIKFKYMKIRAYINLYKENYEKRKEMLEFNTPERPNQHIPVYLT
WDMSFNVIKNTDYAENIITLCA
>B3S837/112-361
KEFTKNIGKQLKRIDQDGGHILIHGGVGSGNISDFQLFLKLNHLLLDLEWGEDNLPDDLE
TFKKKVQYFHKGIENCNTLFIFDDIRKEHHYEFLDFVKKSIVTSQYSKPQNMDKYEYYKM
PEQFTFDNALQVLAQHQNDSNLPKSRQNREISRVIEVCKNHPSAIDIISGLHLRNVKDWQ
QVASIIDNQAQNNESSTTDFKFHRIFDLSIQQLDKNDRHLFRSLGAFKKLPIPIDSIQSL
WKCSQSETVS
>B3RJQ1/132-412
RQTFVDKICSHLKDIDNSQGHLLIHGMAGCGKTIAVCQSVRMVFEQGYFQSHGVYWAKLG
DIGKDKLFTILKSLCIKIGVTWKEPPQNLEEIDAYAKQYFEENQDKNNILFIFDDIWQKS

HYNYLRFATKSIATSRFFHPENKLHYKCISASDRFTYEEAIKVLAKNRNIQDLQSLYNNR
NIALAIESCGGLPLAIALIGGLHLQTDKQWESVIKVIEKKEIGDLPANYDFNLFGTFSLS
IKELEGKKKQCFLLLGVFKKVKIPIGSIMSLWKQNECSTIS
>B3SEL4/5-240
VRKLGKRLEKEANKTAENKDNWMSSSSSNKQVKSTLPDDITPKQIEPVNTANSRLSETLF
IFDDIWKKDHYKYLSFAKKSISTSRFKYLENELDHHCIRLQENLTDDEAIELLALLAVND
NDQTLHQNQVKTLGQNRAVKNVIDSCQGLPLAISLIGRLGLKTEKEWNQAKDIIAKKSAP
TKLAHYDFNLYGTLELSVDSLENKRLFEQLAVFKRVSIPIQSVASLWDCEKSEAHL
>B3SDK9/10-204
GNISNIELCQELKTLANSRLSETLFIFDDIWKKDHYKYLSFAKKSISTSRFKYLENELDH
HCIRLPEKLTYDEAIELLALLAVNDNDQTLRQNPVVKNVIDSCQGLPLAITLIGGLDLKT
DEEWNKAKDIIAKKSADIELAHYGFNLYGTLQLSVDTLNDEIRPLFEQLAVFKRVGIPIQ
SVASLWNYDEIEARN
>B3RTW2/124-405
RTDFIAVISSKLKEIDETGGRILVHGMAGSGKTIAVSQAIRHTAEVDRCFETGGVYWVKI
GDISEDKLFSKLKGLCIKLGIEWKQVPQDLEEIESYLSLYLENNYETSMALFVFDDVWRE
SYYPYLKFAKKSIITSRFIYPDNELDHYCIPASDRFTDDEAVQLLAKYRRAEDVKNLQEN
PYVIRAIKSCGGLPLAIALIGGLRLNSDKQWIDAVAVIERKGNRNRLANYEFNLYGTFSL
SIQQLDDEARRLFLLLGVFKKVAIPTNSIMALWRCDRITAES
>B3SED8/75-255
KIANSRLSETLFIFDDIWKKDHYKYLSFAKKSISTSRFKYRENELDHHCIRLQENLTYDE
AIELLALLAVNDNARTLLKSSVVEKVIDSCQGLPLAITLIGHLGLETEKEWNRAKGIIAK
KSADIELAHYGFNLYGTLQLSVDSLENENKQLFEQLAVFKRVSIPIQSVASLWNYDVIEA
R
>B3SCW1/496-705
ILNKLTYEISINIPNELRKSLPEFCNAIEGALYEKYGSTSEILFIFDDIWSENYIKYLKF
TAKAIVISRLQTDFMKSMHNHVVKASENFTFNEAVHVLHSYQSSLNLKDFLESEYISKII
DYCKGLPLAIALIGGQRIETAEGWRNVLSKIQRSDAVSLPYYRFNLYETFAASIEELSAT
EKILLRKLAVFIKGNIPLRIIGSFWELSRE
>B3SEH0/11-232
DISNIELCKKLKILGIDLNMEWKENIQSIGEIRAYLSSSLEAKPQFSETLFIFDDIWNKD
HYEYLSFAKKSISTSRFMYRENELDHHCIRLPEKLTYDEAIELLALLAVNDNDQTLRQNP
VVKNVIDSCQGLPLAITLIGGLDLKTDEEWNKAKDIIAKKSADIELAHYGFNLYGTLQLS
VDTLNDEIRRLFEQLAVFKRVGIPIQSVASLWNYDEIEARNL
>B3SB46/3-179
ENQPIRPFVVLDDVREANQLLYLNFNCDTIVTTRNADIASKLNREKCVIHIKELLTVEHC
RNVMASTAKIPISTLPKAVDQFIEIVDHSALILTLFGILVQHDKDKWNISDNKWLNQYQS
QYDTKTMLHKLIDMLLSNLNESDLNLILKLDIFEENTIIPINTLSMYWGVNQHYCIS
>B3SBU4/9-293
RHECMDLLLSALKNIDRKSGHVFVYGMAGSGKTTIVSKAIRHLVSNINDLPYGAYWIHIG
NIDQETLFETLRSLCLRLRMSADELPQKLHQVVKCLNQFLKRNRKTSESVFIFDDVWDAS
YFRYFQFASKSIVITRNVDPELKLNYTLVKIPNFTYAETVQILSSKGKQPMISKKHHANS
HHADQYMEIYQIYLGLPLAVSIIRRIEFHDRYKWNDIKAIIDEEEISIGRHKSLNDKLDV
LISCALNNLSEERRELFRLLGVFRSIRIPISMITILWNYSEHETI
>B3SCR8/10-230
GNISDTELCEKLEILCISFDMEWKEETKSIDKICAYLSKRLEENSKLSDTLFIFDDIWKE
SHYKYLSFAKKSISTSRFKYEENGLNHHCIRLPKDLTYDEAIELLALPAGKDTAQTLRDN
QVVKKVIDSCQGLPLAISLISRLRLKTDEDWNKAKDIIAKKSIKIKLAGYDFNLYGTLQL
SVDSLNDEIRQLFEQLAVFKRVSIPIQSIASLWDCEEWEAD

>B3S7Y3/132-414
REDFIDTICSYLEDLNSSNGHVLIYGISGCGKTTSVAQSVKLMIEEQDCFKPHGVYWVKI
GDIQEDELLYILINLCHQLDIQWDRQPQSLKEMDAYLRRYFRGHKSQSEIIFVFDDVRNG
IFFDYLAFANKSIVITPLLYPQTRENYDHIKAPEQFTYQEAIQVLALYQNKRYTKKLRQN
ELVKQILENIRYLPLAVVILAGLRLKTDQEWQEVLAKTEVQDSSFKLKFVEYRFNLFEII
TICINKLDIKKRDLFRQLGVFKRVKIPIHSIMCLWQCNQNEAV
>B3S5J2/191-416
LVHGLPGSGNINENELLEILRNLAVKLRVRWDKSPRNLQEITQYLEQFFSVNKKHSNILF
VFDDIRNQYHLNYLKFAKKFIATSRTLHDSTSCRIISSPISFTQDEAMKILAQHYSKKTL
EEYRNCIDQVIRTCESLPLAVAILGGLKLRNKREWQQILVILAGKDGENNLTDNTSLLYK
LFDYSIHRLDYANQDLFRNLGVFKRVKIPITSIMALWKLDELATRQ
>B3SB07/479-758
REKWITKICDQLKEIGTNGGQVLIYGSPGIGKTVAVAQAVERSVIELGHFQSYKAYWLDI
GKINEDQLAEKLKSLYQILSGSKVFSNELPSILDNINRLFEEDKKVGNNLFVFDDIWHPS
YYNYFKFAKKSIATSREINPNNKLQPSIEISEGFTDIEVREVFKKYEKIENAEICKEDPW
IDDIIIASGGLPLAIGLIGGLGLRTNEQWKNALNSIRDAKCPIKIPNYENNLWGTVELSI
KNLGEHEQKFRQLGAFKYPKVSIDSITSLWQCEKEVGQSI
>B3SE17/216-362
NITSRDKMKSNHNFVRENLTYDEAIELLALLAGNDNAQTLRENPVVKKVIDSCQGLPLAI
ALIGGLDLKTEKEWNAAKDIIADESIDIGLAHYDFNLYGTLQLSVDSLDNENKRLFEKLA
VFKRVSIPIQSVTSLWYYNEINKIKAQ
>B3S2Z6/186-372
TLDEQHPRMILILDNIWDSKLISYLDLKCRTLATTRDSTTAKRINSPCNILQIEPGFHIA
EAKAVLSSWIGIPSIELPSQANSLISKCKGLPLAISIFASLLSKNPHRWDYYISLLTNST
PNHLRLIYQSIHHSHQLEEAIRISIDSLDADLQSYYYDLAVFENDIHIPSRVLNLLWNQD
IELVEDK
>B3S2Z6/129-193
RIAMQDQIRAQLNQLRDQCGWVILVGMAGCGKTVLASRIVREGQLIEQVFPGGITWLTLD
EQHPR
>B3SDZ8/1-254
MAGSGKTTTVCQSVRQATKKGSFKSNGCYWMKIGNISNTELCQKLKRIGIDLNMEWKENI
QSIGEIRAYLCSSLEANSKLSDTLFIFDDIWEKDHYKYLSFAKKSICTSRFEDRAKERGD
GLIRLPKKLEYKEAIELLALLAVNDNARTLLKSSVVEKVIDSCQGLPLAIAIIGRLGLET
EKEWNRAKGIIAKKSADIELAHYGFNLYGTLQLSVDSLENENKQLFEQLAVFKRVSIPIQ
SVASLWNYDVIEAR
>B3SDU7/480-760
RKEFVGEIVSKIVEIQNSSKRSILIYGIGGGGKTVAVSQAVQQVAKMPKYKSRLSAYWIT
LGDSSDDELLTKLKLLAYSLGISFADSDCNDLNVLGCLIRKHIRENSSGPEILFIFDDLW
DDSYMSYLDFYRKAIIISRNKTRAMLKTDQIVIEANTKLTKSEAFEVFRLNQPSRPIEDF
TKNRYISEIIERCQGLPLMISLIAAQKLTTEAEWLEYLIDLQKNLQIKNDDSYFSTLHKN
FNASIEKLQPDDQKLFIMLGIFRKSKISVNLIGSLWKLPLS
>B3SDV6/25-165
SNGCYWMKIGKFEKLTYDEAIELLALLAVNDNDQTLRQNPVVKNVIDSCQGLPLAITLIG
GLDLKTDEEWNQAKDIIAKKSADIELAHYGFNLYGTLQLSVDTLNDEIRPLFEQLAVFKR
VGIPIQSVASLWNYDEIEARN
>B8CD55/1074-1365
RDHELEQLQSRLLAQDLGRRAVVRVEVAGMGGVGKSQLVTEYCYRHFPSEYGLVVWLNAE
TSDTLVADYRQLLADLAADIDVDDINKSTDDIIGEVKTRLFRSQVPWLLVFDNIEDHSLL
DKFVPHGAGSKGHVLITTRHLDTVSAGEGSGNLILGCFDTSESLELLRRSAGDHNMEGEQ

NKAAATELCERLGNLPLALGMAAAYTRRCDVQISEYLDRYIMSEKSGQSMMHGKLNDYAL
TVASSLSLSLGEIEKESETSREVLQLLSFLAPDQHTKSLIRHLLSAKRNLDE
>B8C2I8/409-682
SSDGGIGKSTLAGLVCVRGDVRSRYTQGVAWLNMRRQPDASLNFEQYSKTLSDICHQIGI
QPHHLKLSPFVRTPCEDEAVANVRMRLHMKEARIAMGKLLSSSRFRKKARSSRKTSLLIV
LDDVADESDIEWFRFHHRGDGEKVLNDVLVTSRLGQISTATPITVPALSEQEGMNLLLTE
SDLPSNHPLAKNVSAAKLVKKCLYHPITVKFVGRWLNLKRITSGGMKGFDETLKEINNAL
KDVDQSTNAVDVLYAVLNGACSPLIKGKASQIIK
>B8LCZ1/616-947
SDKAGNGKTTLAVAAIQTVEVREFFSDGIAWIHLGRTPLGEREIRRLYEQLYDQLLGGDD
DDGENDDGGKDDDDSSNSSSGSNGYKEADDFQKGPPGRGKSNVSSAASENKLAKSRRSFQ
GGELEGMKEDLARLLLARRVLICLDDVCRMEDAKWFLFGTRSDGDNSFEDTPHRVLITTR
IPGLVGPSITHEVFVRIFSEHEAVKLLLTAAGRRPNGLPKTSPVFAQARIIVKGCGNSPL
ALRIAGGMLRSRNRNWTLSSPAWKLLVEQCRTSLEEASKIRSFANSVQRLIDLSFATVLD
LKFRSCLRQCFVAFAMVFHDTDSLKVGKGISR
>B7FR32/222-467
RYDCVNQLLDRLKDETDVCVAIRSELEGTGKTTLAALVASHPSILRVFRVLWLPMDQRDV
TYTAYAKLLSNLCDQLGVAPSWPEYVTRFEEPALRQLREKEYMEEARAQMSEILLNVDEN
ILLILDDVQNASQIKNFRFNDRQSIIVTTPDPNLAGVDWTVELDPMSEEEAIELFLAEAD
LPPAHILGCTDEMKAIVRKCNCHPLTVRTTARWYHLKQVTAGLPKAMEELVIDVNRISML
TSVAAS
>B7G1C5/517-835
IITCVTSRHGDKAGNGKSTLAIAVIQTVEVRERFPNGIAWLKLGRGPLSERDIRRLYEDL
YRQLVVKQADIEGSVSEADTVNFHESFASTGSARTEPSETRIDRTVDRADSVRRFEGGDL
EGIKEDLGRMLVRQKVLVCLDDVWRVEDAKWFIFETPSYSAPPRNSPYKILITTRTPSLL
GAGAVQEVFVRILSEHEAVKLLLSTAGRRPYGGRNSTVFNQSKLIVKGCGNSPLGIILVG
SMLREYNRNWNLTSPVWTGIFNQCSLNLEEAAQLRSFRNAFNRVVDTALFTIEDSFFRIA
LRRCFVYFAMGFRANDWML
>B7FQL5/1068-1354
NALGVAGVGKTTLAAMVADHEDVRRFFHDGIAWLHIGQKELNYTRYVQCLRDLVAQLGVD
EHDEPLFPELLNTPGESKAKRRRREEGFMIFVRETMLEFLRYRNVLIVLDNVCFEPDLDW
FDFGPSPVEGDQENEEEFACVVLITSRCRNLLPAADTIEVDMLDEAESITLLIQESGKLP
HSLMAGSPETRAVVLECANHPLAVKSVGRWLNLKHATAGAVSSVEEIHEDVVKSMENVLK
TGGQEDADMMYEILNMSLTPSMNGEPTVIIKFCFSAFVLVFCDQKLL
>F0XVW7/127-382
RAAAADAVVSGLLSETARLAAVAGRGGNGKSVLAASCVADLAGHFVDGVAWVAVGQSAAP
AAVLRSVAAKLLGPYRAAALGTAAALRAAVKRYCASRACLVCLDDCWAPELAASALKLLG
PRSRLLAKDALGPLATDAAAAVVELDKMDAGEGLALLRSVAGAVDGAAGARVVAGARASP
LCLSMVGAALASGAEAGAVADALEAAGDGGYGFADASVAAAVAALPGAAREKYLALAAFP
DDCDLPAEALDLLFGA
>D0N0B3/379-648
NTETYLMVKSLRENAVTACLGAPGIGKSSLIISVAHFVHSRRMFSDGVFYVDLEGQKLST
VRYAIAQSMGMPAADTDEEVFAELGTKNCLLVLDKVEELLDEDENKGEELLHQLISVAPN
LKLLLASRRNMHIPSVTPYSLSISELPLHTATELLCLVAPGCSTSLAERLARICGCLPLA
IRVVGRALANARMTVTPERMIEYLERDEHRFETIRELNQVGHKECVDRCIRSSFCHLDEP
LRLAFMAFGFFRGSFEIEAAEAVLSSVFTE
>D8LPV9/254-544
RPGVQEAMGDLMDPEKALAPYMIVGMGGGGKTVLASALVRKSSVLEHFRGGIFWMEMGRG
ANKSLLHRLQGLAREMGAAPTDAPHGVPHVFDSLEQVMQHLAAVVSRGSSPRLVVLDDVW

EREVVAAFLGVGLKVLVTTRDRSIVGVPGGLLELGDMAEDEALELLWKTSGTVGQPGDGV
RTQMTKVVDRCGRLPLVLAIAGSMPVVKGKGLKDGAWEELIEEFENVTTIMWEPGEESRN
LDMVLGASFNALAARKREEFLRMAVLAPGAVAPFEMLRNLWEIQDVEGTKE
>D7G1G8/42-235
TGGLRSTLLVLDDVWEAKVVEAFETVGLSLLVTTREPLIARMASSHNAAGVSRLIGPLGP
LSGAEATSAAAKAAATRRAPPKEAKQLLDKTGRCPLAVGVVASTLKQAQGPLDWVSTSSK
VKKTFAGLHRRRRVKASTAEQDAEDRVTASIAAGFSNMPEETQMLYSALCILPPGLPVSC
RLLEQVWETGKRTT
>D8LT49/290-589
RGRSRASGEPPVVGVAGPSGAGKSTIASMIVARHDVRGHFSQKGVVWLSVGQGARDRLLD
VMYRLAVMVHELADSSSSPDDGPFRPPRTPDIGVEPEDGAAYVREILAADEGERSSGGFG
ELLVVADDVWEAEVLAELRNTGASILYTTRSEEDLLAASGGSSVLRVDEVTEGEAEELLR
RAAALLDYSKLPDAAHDIIRRGGSVAMDVAYVGRWDIVRGKNDQAAWAAVLAHVIDAQNF
MKGAALLPWRSAVLRVGVTRLGLADQGTKELYLSLGVLPRNLSFSVDDVTALVSDGSAEE
>D7FUD7/61-350
ERAAVQEVADGLTNPEEPRTPYTVVGIGGGGKSVLASAVVRMPSVREHFRGGISWVRVGR
GAKNSLLPLLQGLAREVGAAPTDAPHGVPHALDSLEQVQQHLAAVASTRNSPRLVVLDDV
WEREVVDAFVPLGFKLLLTTRDRSIVCDPAGRLELGDMTEEEALELLRKTSGTVDPGDDV
RMKMTKVVALCGHLPLVLAIAGSMSAVKGKGLTAVAWEELAKEFENVAKKMRARGQQSSS
IKVVLETTFDSLATRKQKEFLMMAVLAAGALAPIEMLSNLWEIEDAEGTH
>D7FZZ7/223-520
RRYVMDAVFEGLASDGGPRLVGLVGDSGSGKTTAASETVRSTEVQEAFSDGIVWLSVNDG
AKERLSSLMLQLARVVYEDIGCSVGRRPTSSDDCAAYIRQRMKKGTGGKKLKCLVVADNV
WEKEVISKLLETGMWVLLSTRNKALVTEAHGEVVGVDELSEADAMSVLRRAAELPPQGKM
PSDAVDLIELCGRVAMDLAFVGRWSTVRGRQDRTAWSDAASKVREEMGKVGSGAASDKLA
ETRDARRKAILSAGFEDLAIGSDDERVQRLYLSLAVFPDGHAFTVRDAAVLLYDRNPS
>D7G722/276-567
ERAAVQEVADGLTTPEESRAPYTVVGMGGGGKSVLASAAVRKPSVREHFRGGIFWVRVGR
GAKSSLLALLQGLAWDVGAAPTDAPHGVPHVLDRLEQVQQHLAAVASTGTSPRLVVLDDV
WEREVVDAFVPLGFKVLLTTRDRSVVGLPAGRLELGDMTEEEALELLRKTSGTVGQPGDD
VRTKMTKVVALCGHLPLVLAIAGSMSVVRGKGLTAVAWEELAKELENVAKKMRARGEQSS
SMKVVLETSFDSLAVRKQEEFLKMAVLAAGALAPIEMLRNLWEVEDAEGTRD
>D7FZZ6/115-408
RRHVMRAVCDALHGEGGPRLVGLVGESGSGKTTAAAEIVRSTEVREAFSDGIVWLSVNDG
ATERLRALMIHLARMVYEDIGGSVGRRPAGLDDGAAYIRQRMQSGHGGKGLKCLVVADNV
WEEAVTSKLLETGMSVLLSTRDEALVRSAEGGVVVGVDELSEEDAMSVLRRASELSPELR
LPDDAVELVELCGRVAMDVAFVGRWSTVRGRSDRTAWSDAADKVRSKIERSRVSDNVWDV
RAVRRKAVLQAGFEDLAIGSDDERIPRLYLSLAVLPNGHAFTAKDATALLYDRV
>D7G5Y4/44-270
SFPGEQPRMVGLAGPGGAGKSTVASMVVTQVDVQAFFRGRVLWLSVGKGAKDRLPALMFE
LANRVRETVLQKMARRPREATVGINSEDGAAYIREEAGKGDTRFLVVADDVWDAEVLGEL
RRAGAWVIYTTRKTELFPGTPAIRLEEVLEEEAEMMLRRAADLGEQARLPPAAYELMKRC
DFAVLDLALVGRWGNIRRRSCESAWRAALDSIIETQRAGGDGGQLLP
>D7G2X0/1-168
MVGLAGPTGCGKSTVTSLVVAREDVRAHFRDGIVWLPVRGKGAEHRLPDLMLLLANMVYE
TVLKSGEASWSSKKLRPPRTPGGVACDRENGAAYIRGALGITRPGGEEGDGGGESHEQRR
WRPRYLIVADDVYEPEVLEELQGIGAWVLYTTTRSASFIRRNGERSEG
>D8LL58/247-527
RRDLFKAVVKDLVATDRATNAAHVLRGMPGGGKTIAAKAVVRCEDVRRSFKDGIFWVQVG

QVGTGNPMALLQGLARDLAHAPSHQPHTVPHEFVDVEHAVSHLEGVRKERNLRCLVVLDD
VWDAQIVPLFLCGGFHSLVTTRDLAVIPRDLQGVCTEVEMLTNAEALELLKNASRATAAI
PTNEGLKVAKDCGFLPLPLAIVGAMGSSRADPDSAETWRGIHALLQQEPELVQDPVGSVL
AVSFCGLKGAARTRFRKLGVLAKGARAPVDMVAHLWELDHD
>D8LJY6/1210-1455
REKLTEQIEATILHPFLPLEIAGIGGPSGSGKTVLAAAVVRDATVRCRFGDRVFWLHAGK
GANHRLVSVLQGLADTVYAWLTDGEHAGGASSSALLRRSVGSGGGGSDAGGGGGDPGAAL
ADPNLREPVRFRDQDQAVNYVADLCRGPLLAGLRCLVVVDDVHEREVVDALWKSGCQLLV
TSPVKGLLQAVGAEATMAQPLGVEVARQLATRAAGEVALCEEGGRLVDLCRGCPLALAMS
GAITQA
>D8LCK2/610-889
GAGGKGETVVTFAGSSGAGKACLAAEVVGRRDVRAKFGDSVLWLQVGRSGGTDLACLLHR
LAHAYHRVVLCKRLRNPPPLPPISRFRGPRNGEAAAEAAAAAAAAAETPGDGGSEEAAAA
TAAAATAASAEATATAAATWFTPYMSGLGLRCLVVLEDVWEAEVVAAASAAGFDLVVTTA
FRDLVPDSPLCTRLDVGGLARDEARSLLRRCSGLAWQAPLPPSADGVIKACGALALPLTI
AGSLPAVRCRSDEAAWANLYSILSHKATRLGESSSCSENH
>D7FZW8/194-474
RRHAMATVLDALNGSDRACVVALAGYSGSGKTTVAAEIVRSTEVREAFSDGIVWLPVNEG
AKKRLPSLMLQLARMVHEEIGGSVGRVPSASEDSATYIKQRLEEGHKGRRLKCLVVADNV
WEEDVVSKLVETGMWVLLSTRDEAVVKRSRGKAVVVDELSQADAESVLKRAAELPRESRL
PDDAVDLIKLCGRVAMDVAFVGRWSTVRGRHDPASWSDAASTIRTEMEKVGHDPDNDSAE
NIPAKRRKAILQGGFEDLAIGSDDERVQRLYLSLAVMPDGH
>D7G6W2/234-411
LLGRAIGYLTNTAVGDAPCVLTGMAGAGKSVLASAVVRDEKVREHFHAGMFWVRARDAKD
QLHACLEGFALRVASTSGTTSPGLGSVEEVTRYLKAVCADSVSPRLVVLDDVWEREIVDA
LKLTGLQLLVTTRDRSVVSMPGECVEVGDMEEDEALEVLRVGCGASKNLELPRAEALQ
>D8LGG5/233-525
ERAAVQEVADGLTAPEEPRAPYTVVGLGGGGKSVLASAVVRKPSVREHFRGGISWVRVGR
GAKNSLLPLLQGLAREMGVAPTDAPHAVPQVLDSLEQVQQHLAAVVASTGTSPRLVVLDD
VWEREVVDALVPLGFKVLLTTRDWSVVVVPARRLDLGDMTEEEALELLRKTSGTVGQPGD
YVRMKMTKVVALCGHLPLVLAIAGSMSAVKGKGLTAVAWEELAKSFENVAKKMRARGQQS
SAIKVVLETSFDSLAVQKQEEFLRMSVLAAGALAPIEMLRNLWEIEDAEGTRD
>D7FZX2/198-494
RRNVTETVFQALNADGGPHLVGLVGDSGAGKTTAASAIVSSTEVREAFSDGIVWLPVNEG
ALDNLPCLMLQLAQMVFEDIGGSVGRRPSGSDDGLDYVKQQVENGHGSKGLKCLVVADNV
WESDVVSQLLETGMWVLLSTRDEELVKRAKGEVVGVDELSEAEAESVLRRAAELSPEARL
PDDAMDLIELCGRVAMDLAFVGRWSTVRGRQDRTAWSDAACKVRAEMNKERVNAESNGSE
DSRVNRRRAILQAGFEDLAIGSDDERVQRLYLSLAVLPDGHAFTVKDAAVLLYDREP
>D8LFN6/236-519
RDRVVDAVYEHLSCGQTPQVVGLVGRSGSGKTTCAASIVGESTGRRHQNETGDQMLRRLN
RVREHFRDGVVWLRVGRGGGSAERVPALMSQLAKTVFEDLKHSYGFAPGASPTGGGSAFI
SDFVRGKGSAAGRKRCLVVADDVWEVDILEELRHTGMWVLFTSRDPDLVERVEGSVVPVD
QLSKAEAECLLRGAAELSTGSSLPSAAAQVIERCDRMANRLEFVGRWSTVKGSDDEEDWA
EAVSAIDAEMSAMAGEANAQKSDEDLSGVKRVAILRAGFLDLAA
>D7G5K4/214-315
RRHVMDTVVEALTGEAGPHLVGLVGDSGAGKTTAASELVGSNVVRESFSDGIVWLAVNYG
ANKRLPSLMLQLARMVHEDIGGCVGGRPNESGDNADAYIKQR
>D7G7W8/217-501
RNSLISEVVGNLTAANAPRSPYVLQGSGGAGKTVLATAVVRNEEVRKRFGRGIFWLDVGR

HGNLKLPALVQRLAREMDVVSENMDGLDDCVRDIAFAVARDNSVPRLLVLDDVWHEEVMD
ALRPTGLQILVTTRLASVGRGMLVGDMEEGEARELLSKKSGAVALPVPEANQVARDCGCH
PLTLVIAGSLRCVREAPDSALAWRELHSEIERIKRSSRGLEMATDSDEEPSQSSLFPVLS
LSFERLKVQEQDSFLSLAVLARGVPAPVAMLANLWDKDEKGAKKE
>D7FQM2/44-129
SFPGEQPRMVGLAGPGGAGKSTVASMVVTQVDVQAFFRGRVLWLSVGKGAKDRLPALMFE
LANRVRETVLQKMARRPREATVGIIR
>D7FZP7/146-425
GKSEFFEMPRVVGMSGPGGSGKSTLASMVIARQDVREYFYKGVLWLPVGEGAKHCLPELM
LHLARMVYETVLRKACRPPRLAGVGVDPEDGAGYIHEVVNESNHRFLVVADDVREPEVLE
ELKWAGVWVLYTSRRDDLLTGAPLLRLDAVTKEEGGMVLRRAAGLGDDAALPPAAHELMQ
RCEYGAMQLAFAGRWGVVRGRSDEEAWRAALGHIAEAQKRGGNGRLLAWRDAMLRIGPDE
LEADSLHTKELYLSLAILPKGLAFRSKVAAVLLYGDECSA
>D8LRB0/548-881
RTAMQERVAAGLLGRVSPWDPPMVITGPPGAGKTVLSSAVARRSDVRRHFRDGIFWLPAG
KDATERLPWLLEYLAVQLSLVLSSGQAGESKANRMAALTGRGAKQDDEVEVELPWGTRGP
REGEVAAFLAAHLATRGLTCLLVVDDLWDRESLDQVRDLGFHVMVTANSKGLLWTQQGAG
LAATLGSEVVAVDRMDDDECKLLLQRCSMIETVDWAALRDTEPVKSIIKLCRYGPHALVM
VGTTLQGQGRSFSWDATRDSLARTVDSSRSLLQNGASQDPDYVSLYSAGKVCTLKLPDHI
RNRYLHLAILPKHVPAPEQMLESLWDTESDEVFD
>D7FH19/249-521
RTAMLEEVVDDLTHPHRSASATHCLLGMGGGGKTLIASSVVRDDRVRARFKRGIFWVPVG
REGKDVALLLEHLAVELSRVPTDTPRSCPNRFSGAEEALRHLSSVCAEDGLRCLVVLDNV
WNVEVVNAFASTGFHVLVTTRQRAVISAVHAGVWTEVGDMSEEDALEVLSKASQADGPLP
AEEARQVALDCGMLPLALGMIGALAKDQPLDPLSWRAVHKKLQEKRAMFRDVENGKLFST
MDTSVCNLPSNQREQLQLMAVMAFWSRRNLGNA
>D7G720/259-550
ERAAVQEVADGLTAPEESRAPYTVVGMGGGGKSVLASAVVRKPSVREHFRGGIFWVRVGR
GAKSSLLALLQGLARDVGAAPTDAPCGVPRVLDSLEQVRQHLAAVTSTGTSQRLVVLDDV
WEREVVDAFVPLGFKVLLTTRDRSVVGVPAGRLELGDVTEEEALELLRKTSGAVGQPGDD
VRTKMTKVVALCGHLPLVLAIAGSMSVVRGKGLTAVAWEELAKELENVAKKMRARGEQSS
SMKVVLETSFDSLAVRKQEEFLKMAVLAAGALAPIEMLRNLWEIEDAEGTRD
>D7G247/42-119
RDSFERNNSAENQRVVFGLAGPGGAGKSTVASMVITREDVRASFHKGVLWLPVGQGAKDR
LPSPMLDLAGMVHKTALS
>D7FZY7/217-505
RLYVMERVFEALTAVGGPHLVGLVGDSGSGKTTAASEIVRSNEVRQAFSDGVVWLTVNHG
AKDRLPSLMLQLAHMMCEEVGGRRPATSDDGAAYIKEKMQRGHGGKGLKCLVVADNVWEK
EVVWKLLETGMWVLLSTRDEELVVGSEGEVVGVDEMSEVEAESVLRKASELPPETRLPDD
AVDLIELCGRVAMDLAFVGRWSTVRGRSDRTAWSDAAGKVRAEMGKIEGERVSGTVVETR
DARRKAVLQAGFEDLASGSDDERVQRLYLSLAVLPDGRGFTVKDAAVLL
>D8S7E6/1-188
MEETLERVVSDLLESPISRKWVGVHGAGGAGKTLLAKRVCDNDQVKGRFDPVLWLTFGQS
VQVEAKQDELAKKIQLEGDKISNEALRRGLVGKRCLLVLDDVWKFNDVNLFDVVQENGSK
IFITTRKHDVLDSRGAIKTEMGLLGKEDSMKLFAVHAFPNQNHAQVSKAVVEQVVEKCGG
LPLVLEVI
>D8T4X4/1-159
MLVLDDVESHAQLLKLLPPLGRSSRVIVTSRDRDTLRSLTFIHEVGQLKHEDARMLFKQH
AKPDAGLDRSLVKEIVGECKGVPLVLEVAGLKLQGKSDERDWRGAKSKLSSYPEVVDKLR

ISLEPLHQREREIFLHICCFFGEVSRASVFWIWEAMRQR
>D8T419/171-466
ASKARKIRALLEDETSSSRKVVLIHGLSGMGKSCLARYVAADPPKRFVHGAVDLLLGQGC
SRRSGTPEYHSRLAAKLCHLLRVLGRKRGEIDGLDLEEACQLLQETLLGRSILVVLDDVW
EPDIIARFTRLYDNECRFLATTRNQAVYETTTEAEKVEIGTEDVSELSRGILMQHSQLSE
EELPATTELLIQRCGHHPLTLAVLGKALFKETRPEQWDKALDDLSTYAAQAPVPVHYLND
KEAESAATVFGSFDYSLHAMTTHARDLFLSLAALCWATPIPEPCLEAIWQALHQDT
>D8SZ68/4-195
MEETLERVVSDLLESPVSRKWVGVHGAGGAGKTLLAKRVCANDQVKGHFDPVLWLTFGQS
VQVRSLSVEAKQHELGEQIELEGRVSNETLRRGLAGKRCLLVLDDVWKSIHLDLLDVVQE
NGSKIFITTRKRDVLDSRGAIKTEMGFLGKEDSMKLFTVHAFPNQKHAQVSKEVVEQVVE
KCGGLPLVLKVI
>D8SY91/412-707
LRSKVQEVCDLLASSPHKVVLVHGLSGIGKSSLAHFVGASSLPTRFVDGSLKVLLGYGCS
RAALGNNTKEYQKDFAEKIVHLLRTQLGYKKHDLDGISLKEAFVILEETLKEKNYLIIVD
DVWEADVVIRFMKLQGNRCKYLVTTRLYAMVSSDVDRVEVTKEDVAQVGKEILRHHSQVQ
DLPELADELLHRCGHHPLTVTVIGQALEGESRHEQWLQAINDLSIYASRAPVPNKDLMDD
DVSNAATVFGSLEFSLKAMEKETREFFTAFAALSWVEPIPEPCLEEMWRALGLQGT
>D8SYM5/187-312
SSQDCWALLEEVVAESSYLIVLDDAVASKLLRHCSHHPLAVAILGEALRGSWRRAEWNAV
ANHLEVCSGNHLNWLAGVMSTIELSTVGLKHDTHDLFTSLAAISWDEPVPVECLQALWSA
LGKKSP
>D8SYM5/109-219
PSSSMCSQHSVIIVHGPSGVGKTSLAAYIVANPPQCFADSAVEIRVGKIAGSDRRLKSKL
KSLLQELNCQKELLQRAGSSQDCWALLEEVVAESSYLIVLDDAVASKLLRH
>D8S7E3/4-206
MEETLERVVSDLLRSPASSKWVGVHGAGGAGKTLLAKRVCDNDQVKGCFDPVLWLTFGQS
VQVRSLSQHELAKRIGLEGDQISNETLGRGLVGKRCLLVLDDVWKSIHLDLLDVVQENGS
KIFITTRKRDVLDSRGAIKTEMGLLGKEDSMKLFAVHAFPNQNHAQVSKAVVEQVVEKCG
GLPLVLKVIGRRMAATQDWDYVL
>D8S7M5/1-82
MGLLGKEDSMKLFGVHAFPNQNHAPVSKEVVEQVVEKCGGLPLVLKVIGRRMAATQDWDN
VLSRLKEQQLLALDEEQQVLEG
>D8T4X5/159-214
SEGRVAGILSRFRKSGQMVTSVGITGQGAIGKTRLAKEVFNRIQGNFEASCFVRVR
>D8S792/237-344
VLSRGAIKTEMGLLGKEDSMKLFAVHAFPNQKHAQVSKVVVEQVVEKCGGLPLVLKVIGR
RMAGTQDWDYVLSKLKDQRLIALDEEQQMVANIAEATNVLERLKNHAL
>D8S792/160-245
MEETLERVVSDLLGSPVSSKWVGVHGAGGAGKILLAKRVCDNDQVKGLFDPVLWLTFGQS
FQVEAKKHELAKKIRLEVLSRGAIKT
>D8RCG2/1-141
SRVIVTSRDRDTLGRLTFIHEVGQLKHEDARMLFEQHAKLDAGLDRSLVEEIVGECKGVP
LVLEMAGLMLQGKSDERDWRGAKSKLSSYPEVVDKLRISLEPLHQREREIFLDICCFFGE
VSRASVCWIWEAMEDVEESMM
>D8S4H8/1-171
GGSGKTLLTQTVTNSLSVNNHFGGKVYWLTVARNPNIARLQSRLRKHIGRGEQLFSSEEA
GRKTLINCLKNSKVLLILDDVWDGNVLQWFDVVDAPGSKILLTSRNNSVFDKVPSTVIEV
KLLSDEHSWNLFCGCAFPGQDRQQLEHDMEPILAERVKQVVSECKGLPLAL

>D8RWT3/380-661
DEEFSRFKSLLLDEGRPERIGLFGPSGVGKTFLVTHLCRDGEIASHFDGIIWISFDYRER
FDTLVTMQADILSRLTGNDRRVASVANGSREIFNFITRSSLKRYMLVLDNVWKKNWLHLL
DQEGDMGRGNKVVAIASNAKLFPEDRWTSVKLDPLSFDDGYKLICMQAFGGEDQVPDTLD
RDYVEKVVWLCNGVPKALQVMGRYMRRNKDRPWFYWQRLLEKINRWELDEWKGIFEMVKL
SFMELDEDAQLLFLSLALLRDPWDESSLFHLWRAMRCSEDDN
>D8S968/187-312
SSQDCWALLEEVVAESSYLIVLDDAVASKLLRHCSHHPLAVAILGEALRGSWRRAEWNAV
ANHLEVCSGNHLNWLAGVMSTIELSTVGLKHDTHDLFTSLAAISWDEPVPVECLQALWSA
LGKESP
>D8S968/109-219
PSSSMCSQHSVIIVHGPSGVGKTSLAAYIVANPPQCFADSAVEIRVGKIAGSDRRLKSKL
KSLLQELNCQKELLQRAGSSQDCWALLEEVVAESSYLIVLDDAVASKLLRH
>D8SB17/1-181
MDPEKGKWIGICGMGGSGKTTLASLVNNDARVLEHFEGRVYFLGVHRNASVENLQRALFN
LLDDDRGRALDQPKSTLHDLLGGQRTLLILDDVWEGAVLDGLDVLDRANGSKMLVTTRNS
GLLERRKAEVITSAGLLSSADNKKFFCMHAFKDAGSEQPGIDDLVGEMAAACKGLPLALK
V
>D8RI45/179-256
HELAKRIRLEGNQISIETLRRGLDGKRCLLVLDDVWKFIHLDFFDVVQENGSKIFITTRK
RDVLDSKGAIKTEMALGK
>D8S9M7/1-198
MLERVVSDLLGSPVSSKWVGVHGVGGAGKTLLAKRVCENHQVKEHFDPVLWLTFGQTCQV
EAKQAELANQIRLESRISNETLRRGLAGKRCLMVLDDVWTSGDLLDLFDVVQENGSKIFI
TTRKLDVLDSRGAIKTKMGLLGKEDSLKLFTVHAYPNQKYAQNQVKKIVMEQVVEKCGGL
PLVLKVIGRRMAGKQDWD
>D8S7E8/1-266
MEETLERVVSDLLGSPASSKWVGVHGAGGAGKTLLAKKVCDNDQVKGCFDPVLWLTFGQS
VQVEAKQDELAKKIRLEGDQISNETLGRGLVGKRCLLVLDDVWKSIHLDLLDVVQENGSK
IFITTRKRDVLDSRGAIKTEMGLLGKEDSMKLFVVHAFPNQNHAQVTKAVVEQVVEKCGG
LPLVLKVIGRRMAGTQDWDYVLSKLKDQRLIALDEEQQVLERLKLGYDALLPLRIQQHFL
FFAAFPEDYPIGFWELFRCWKGEGLV
>D8S7F2/1-266
MEETLERVVSNLLGSPVSRKWIGVHGAGGAGKTLLAKRVCDNDQVKGRFDPVLWLTFGQS
VQVEAKQHELAKKIRLEGDQISNETLRRGLVGKRCLLVLDDVWKFIHLDLFDVVQENGSK
IFISTRKRDVLDSRGAIKTEMGLLGKEDSMKLFAVHAFPNQNHAQVSKAVVEQVVEKCGG
LPLVLKVIGRRMAATQDWDYVLSKLKDQQLIALDEEQQVLERLKLGYDALLPLRIQQHFL
FFAAFPEDWPIRFWELFRCWKGEGLV
>D8T728/370-665
LRSKVQEVCDLLASSPHKVVLVHGLSGIGKSSLAHFVGASSLPTRFVDGSLKVLLGYGCS
RAALGNNTKEYQKDFAEKIVHLLRTQLGYKKHDLDGISLKEAFVILEETLKEKNYLIIVD
DVWEADVVIRFMKLQGNQCKYLVTTRLYAMVSSDVDKVEVTKEDVAQVGKEILRHHSQVQ
DLPELADELLHRCGHHPLTVTVIGQALEGESRHEQWLQAINDLSIYASRAPVPNKDLMDY
DVSNAATVFGSLEFSLKAMEKETREFFTAFAALSWVEPIPEPCLEEMWRALGLQGT
>D8S7Q2/171-466
ASKARKIRALLEDETSSSRKVVLIHGLSGMGKSCLARYVAADPPKRFVHGAVDLLLGQGC
SRRSGTPQYHSRLAAKLCHLLRVLGRKRGEIDGLDLEEACQVLQETLLGRSILVVLDDVW
EPDIIARFTRLYDNECRFLATTRNQAVYETTTEAEKVEIGTEDVSELSRGILMQHSQLSE
EELPATTELLIQRCGHHPLTLAVLGKALFKETRPEQWDKALDDLSTYAAQAPVPVHYLND

KEAESAATVFGSFDYSLHAMTTHARDLFLSLAALCWATPIPEPCLEAIWQALHQDT
>D8T4X2/195-327
PSENGVNVLLVLDDVESHAYLLKLLPPLGRSSRVIVTSQDRDTLRSLTFIHAVGQLKHED
ARMLFEQHAKPDAGLDRSLVEEIVGECKGVPLVLEMAGLMLQGSATGEAPRAKVSRASVC
WIWEAMEDVEESM
>D8SZ17/109-224
MEETLERVVSDLLGSPVRSKWVGVHGAGGAGKTLLAKRVCDNNQIKGRFDPVLWLTFGQS
FQVEAKQHELAKKIRLAVRSQRIPKPKSCSSQQATQDWDYVLSKLKDRQLLALDEE
>D8S0D0/3-195
VSEKLEEIIRLLKDPEKGKWIGICGMGGSGKTTLASLVNNDARVHEHFEGRVYFLGVHRN
ASVENLQRALFNLLDDDRGRALDQPKSTLHDLLGGQRTLLILDDVWEGAVLDGLDVLDRA
NGSKMLVTTRNSGLLERRKAEVITSAGLLSSADNKKFFCMHAFQDAGSEQPGIDDLVGEM
AAACKGLPLALKV
>Q9AXD4/191-479
RDRIVDFLLGKTTTAEASSAKYSGLAIVGLGGMGKSTLAQYVYNDKRIEECFDIRMWVCI
SRKLDVHRHTREIMESAKKGECRRVDNLDTLQCKLRDILQESQKFLLVLDDVWFEKSHNE
TEWELFLAPLVSKQSGSKVLVTSRSKTLPASICCEQEHVIHLENMDDTEFLALFKHHAFS
GAEIKDQLLRTKLEDTAEEIAKRLGQCPLAAKVLGSRLCRKKDIAEWKTALKIGDLSDPF
TSLLWSYEKLDPRLQRCFLYCSLFPKGHVYRPQELVHLWVAEGFVGSCN
>C4J2M9/7-117
EEYSDPQIIRDILGECEQFEFCMKILAHSFYARPKRSNQELTKLRDSLQAVSPKSFEGVA
AKMLKFSYNDLPKEYKSCLLYLAIFPPGRSIRRSTLVARWVVEGLINRDDW
>B6STS5/145-283
QQLAELESMAFRDCGAGKLNGVGIVGMGGIGKTVLAQLLFSSPRAKGRFFPRIWMCMSRT
ASAGADRRKEVLQGMLMALGHEEDAILSMNGSDSLTELTIAVHEQLEGKRFLIVFDDVWH
IDSWYSDVVGVGPQNAMRR
>B6STS5/286-405
DLSVLSERLAFALPKGRGGLVIVTSRLEQAAEAMVRKSCLYRVRPLVDSASWAIFMDVVL
SQPQEKKAVDLVTVNHMKQEILETCGGFPSAAKTMGDIFASSSVSPPASTSTSEELGKSD
>Q7XY07/168-456
RDRIVDFLLGKTTTAEASSAKYSGLAIVGLGGMGKSTLAQYVYNDKRIEECFDIRMWVCI
SRKLDVHRHTREIIESAKKGECPRVDNLDTLQCKLRDILQESQKFLLVLDDVWFEKSHNE
TEWELFLAPLVSKQSGSKVLVTSRSKTLPAAICCEQEHVIHLENMDDTEFLALFKHHAFS
GAEIKDQLLRTKLEDTAEEIAKRLGQCPLAAKVLGSRLCRKKDIAEWKAALKIGDLSDPF
TSLLWSYEKLDPRLQRCFLYCSLFPKGHRFEPDELVHLWVAEGFAGSCN
>Q9ZTI9/1-98
LLVLDDVWNEDPEKWDRYRCALLSGGKGSRIIITTRNKNVGILMGGMTPYHLKQLSNDDC
WQLFKKHAFVDGDSSSHPELEIIGKDIVKKLKGLPLAL
>B7ZYM5/472-680
SKGYSGGANIVCINGSPGIGKTELALEFACRYSQRYKMVLWIGGEARYLRQNILNVSMNL
GLDISAEAEKERGRIRSFEEQEFDAFQRVKRELFRDVPYLLIIDNLESERDWWEGKDLHD
FIPRNTGATHVIVTTRLPRVMNLEPMQLPQLSYIDAMALIQGKRKKDYLPEEAEVLRKFD
ERLGRLSFGLWVVGSLLSELMIAPSTLFE
>D7PSF6/191-477
RDRIVDFLLGKTTTAEASSAKYSGLAIVGLGGMGKSTLAQYVYNDKRIEECFDIRMWVCI
SRKLDVHRHTREIIESAKKGECPRVDNLDTLQCKLRDILQESQKFLLVLDDVWFEKSDTE
TEWELLLAPLVSKQSGSKVLVTTRRETLPAAVCCEQVVHLKNLDDTELLALFKHHAFSGA
EIKDQLLHTKFEHTTEEIAKRLGQCPLAAKVLGSRLCRKKDIAEWKAALKLGDLSDPFTS
LLWSYEKLDPRLQRCFLYCSLFPKGHRYEPNELVHLWVAEGFVGSCN

>C0PEA3/196-479
HGDVERVAALVLGDPDGGTSYAVVPIVGMAGVGKTALMQHVCGMETVKSCFELTRWVWVS
QDFDVVSVTRKIVEAITRSRPECGELSTLHELIVEHLAGKRCLIVLDDVWDDNPSHWNSL
TAPLSHCAPGSAVAVTTRSNKVARMVSTKVYHLKCLSDEDCWLVCQRRALPNSGANVHKE
LVEIGERIAKKCHGLPLAAEAAGSVLSTSAVWEHWNEVLNNDLWADNEVKNLVLPVLKVS
YDHLSMPLKRSFAFCSLFPKGFVFDKDLLVQLWTAQGFVDAEGD
>Q9ZTI4/1-167
GVGKTTLLNKFNNDFLINSHDVNVAIYIEVGKDFDLNDIQRIIGDRLGVSWENRTLKERA
GVLYRVLSKMNFVLLLDDVWEPLNFRMLGIPVPKHNSQSKIVLTTRIEDVCDRMDVRRKL
KMECLPWEPSWELFREKVGDHLMSASPEIRHQAQALAMKCGGLPLAL
>Q5BMB3/183-470
IDENKQTLISSLKFEDPSLRIIAVWGMGGVGKSTLVNNVYKNEGSNFDCRAWVSISQSYR
LEDIWKKMLTDLIGKDKIEFDLGTMDSAELREQLTKTLDKRQYLIILDDVWMANVFFKIK
EVLVDNGLGSRVIITTRIEEVASLAKGSCKIKVEPLGVDDSWHVFCRKAFLKDENHICPP
ELRQCGINIVEKCDGLPLALVAIGSILSLRPKNVDEWKLFYDQLIWELHNNENLNRVEKI
MNLSYKYLPDYLKNCFLYCAMFPEDYLIHRKRLIRLWIAEGFIEQKGA
>Q8GS26/172-455
RDQAKNQIISKLIETDSQQRIKIVAVIGLGGSGKTTLAKQVFNDGNIIKHFEVLLWVHVS
REFAVEKLVEKLFEAIAGHMSDHLPLQHVSRTISDKLVGKRFLAVLDDVWTEDRVEWERF
MVHLKSGAPGSSILLTTRSRKVAEAVDSSYAYDLPFLSKEDSWKVFQQCFGIAIQALDTE
FLQAGIEIVDKCGGVPLAIKVIAGVLHGMKGIEEWQSICNSNLLDVHDDEHRVFACLWLS
FVHLPDHLKPCFLHCSIFPRGYVLNRCHLISQWIAHGFIPTNQA
>Q8H6V0/172-455
RDQAKNQIISKLIETDSQQRIKIVSVIGLGGSGKTTLAKQVFNDGNIIKHFEVILWVHVS
REFAVEKLVAKLFEAIAGDMSDHLLLQHVSRTISDKLVGKRFLAVLDDVWTEDRVEWERF
MVHLKCGAPGSSILLTTRSRKVAEAVDSSYAYDLPLLSMEDSWKVFQQCFGIAMKALDPE
FLQSGIEIVEKCGGVPLAIKVIAGILHGMKGIEEWQSICNSNLLDVQDDEHRVFACLWLS
FVHLPDHLKPCFLHCSIFPRGYVINRCHLISQWIAHGFVPTNQA
>C0HGT8/175-446
LSMETKDDDALESHQALKIVSIVGVGGLGKTTLAKTVHDMLKKQFDCSAFISIGRTPNLN
RTFEKMLLKLDREYKQVDMARWDLEQFKNELDEFLKDKRYLIVVDDIWDVDSWEAIRYAL
KDNNCGSRIIMTTRNFGIVTKLEEVYRLKPLSNANSKKLFYKRIESQEGESLDGELSSKI
IHKCGGIPLAIIAIASLLVERSREEWSEVYDKIGLGNEDNTTKIMSYSYYDLPPYLKPCL
LQLSIYPEDCIIDTNSTIWKWIGEGLVHLEKE
>Q8S458/191-479
RDRIVDFLLGKTTTAEASSAKYSGLAIVGLGGMGKSTLAQYVYNDKRIEECFDIRMWVCI
SRKLDVHRHTREIIESAKKGECPRVDNLDTLQCKLRDILQESQKFLLVLDDVWFEKSHNE
TEWELFLAPLVSKQSGRKVLVTSRSKTLPAAICCEQEHVIHLKNMDDTEFLALFKHHAFS
GAEIKDQLLRTKLEDTAVEIAKRLGQCPLAAKVLGSRLCRKKDIAEWKAALKIGDLSDPF
TSLLWSYEKLDPRLQRCFLYCSLFPKGHRYDPNQLVHLWVAEGFVGSCN
>Q8H6U6/172-453
RDQAKNQIISELIETDSQQKIVSVIGLGGSGKTTLAKLVFNDGNIIKHFEVVLWVHVSRE
FAVEKLVEKLFKAIAGDMSDHPPLQHVSRTISDKLVGKRFLAVLDDVWTEDRVEWEQFMV
HLKSGAPGSSILLTTRSRKVAEAVDSSYAYNLPFLSKEDSWKVFQQCFGIALKALDPEFL
QTGKEIVEKCGGVPLAIKVIAGVLHGIKGIEEWRSICDSNLLDVQDDEHRVFACLSLSFV
HLPDHLKPCFLHCSIFPRGYVINRRHLISQWIAHGFVPTNQA
>Q7X8H3/168-456
RDRIVDFLLGKTTTAEASSAKYSGLAIVGLGGMGKSTLAQYVYNDKRIEECFDIRMWVCI
SRKLDVHRHTREIIESAKKGECPRVDNLDTLQCKLRDILQESQKFLLVLDDVWFEKSHNE

TEWELFLAPLVSKQSGSKVLVTSRSKTLPAAICCEQEHVIHLKNMDDTEFLALFKHHAFS
GAEIKDQVLPTKLEDTAVEIAKRLGQCPLAAKVLGSRLCRKKDIAEWKAALKIGDLSDPF
TSLLWSYEKLDPRLQRCFLYCSLFPKGHRYESNELVHLWVAEGFVGSCN
>Q8S454/192-477
DHIVDFLLDKTTTAQASSAKYSGLAIVGVGGMGKSTLAQYVYNDKRIEECFDVRMWVCIS
RKLDVHRHTREIMESAKKGECPHVDNLDTLQCKLRDILQESHKFLLVLDDVWFEKSDTET
EWELLLAPLVSKQSGSKVLVTTRCETLPAAVCCEQVVHLKNLDDTEFLALFKHHAFSGAE
IKDKLLHTKLEHTTEEIAKRLGQCPLAAKVLGSRLCRKKDIAEWKAALKLGDLSDPFTSL
LWSYEKLDPRLQRCFLYCSLFPKGHRYEPNELVHLWVAEGFVGSCN
>Q9AT66/191-479
RDRIVDFLLGKTTTAEASSAKYSGLAIVGLGGMGKSTLAQYVYNDKRIEECFDIRMWVCI
SRKLDVHRHTREIIESAKKGECPRVDNLDTLQCKLRDILQESQKFLLVLDDVWFEKSHNE
TEWELFLAPLVSKQSGSKVLVTSRSKTLPAAICCEQEHVIHLENMDDTEFLALFKHHAFS
GAEIKDQLLRTKLEDTAEEIAKRLGQCPLAAKVLGSRLCRKKDIAEWKAALKLGDLSDPF
TSLLWSYEKLEPRLQRCFLYCSLFPKGHRYDPNQLVHLWVAEGFVGSCN
>B4FET8/36-220
RQVEAELVINFLLHTQPHSSEELEVLPIIGPCRVGKSTLVAHVCKDERVQDHFSEILWLN
DLAFTDDERAFRQGYAMEHQNQVSNWNKDKRSLVVIELSGNLHEDAWNRFYLACKRSFCS
GSKIVVTSSSDRAAKFGTAQALALKHLSHEAYWYFFKTLAFGSMDPETHPRLAQVAMEIA
RTQNR
>B6SPM3/169-391
RQVEAELVINFLLHTQPHSSEELEVLPIIGPCRVGKSTLVAHVCKDERVQDHFSEILWLN
DLAFTDDERAFRQGYAMEHQNQVSNWNKDKRSLVVIELSGNLHEDAWNRFYLACKRSFCS
GSKIVVTSSSDRAAKFGTAQALALKHLSHEAYWYFFKTLAFGSMDPETHPRLAQVAMEIA
RTQNRSINAAYIICYLMRNNFNIHFWCKVLRFFRVFVQKHVSR
>Q8H6V2/172-453
RDQAKNQIISELIETDSQQKIVSVIGLGGSGKTTLAKLVFNDGNIIKHFEVVLWVHVSRE
FAVEKLVEKLFKAIAGDMSDHPPLQHVSRTISDKLVGKRFLAVLDDVWTEDRVEWEQFMV
HLKSGAPGSSILLTTRSRKVAEAVDSSYAYNLPFLSKEDSWKVFQQCFGIALKALDPEFL
QTGKEIVEKCGGVPLAIKVIAGVLHGIKGIEEWRSICDSNLLDVQDDEHRVFACLSLSFV
HLPDHLKPCFLHCSIFPRGYVINRRHLISQWIAHGFVPTNQA
>Q9ZTI8/1-102
LLLLDDVRKRFRLEDVGIPTPDTKSQSKLILTSRFQEVCFQMGAQRSRIEMKVLDDNATW
NLFLSKLSNEAFAAVESPNFNKVVRDQARKIFSSCGGLPLAL
>Q7XY06/169-457
DRIVDFLLGKTTTAEASSAKYSGLAIVGLGGMGKSTLAQYVYNDKRIEECFDIRMWVCIS
RKLDVHRHTREIIESAKKGECPRVDNLDTLQCKLRDILQESQKFLLVLDDVWFEKSHNET
EWELFLAPLVSKQSGSKVLVTSRSKTLPAAICCEQEHVIHLENMDDTEFLALFKHHAFSG
AEIKDQLLRTKLEDTAEEIAKRLGQCPLAAKVLGSRLCRKKDIAEWKAALKIGDLSDPFT
SLLWSYEKLDPRLQRCFLYCSLFPKGHRFEPDELVHLWVAEGFVGSCNL
>Q9ZTI7/1-156
LLLLDDVWNRDIDKWRKLKACLMQGIGCAILVTTREQQIAQFMGTVVNGSWAKSYHEVAI
LDEEYIQEIIETRAFSSPKSKSDYLVKLAGLITERCAGSPLAAKAIGSVLRNKTTDGEWE
DVLQRSTICNDETGILPILKLSYNDLPIDMKQCFAF
>Q9AXD3/190-478
RDRIVKFLLGKTTTAEASSTKYSGLAIVGLGGMGKSTLAQYVYNDKRIEECFDVRIWICI
SRKLDVHRHTREIIESAKKGECPRVDNLDTLQCKLRDILQESQKFLLVLDDVWFEKSHNE
TEWELFLAPLVSKQSGSKVLVTSRSETLPAAICCEQEHVIHLENMDDTEFLALFKHHAFS
GAEIKDQLLRMKLQDTAEEIAKRLGQCPLAAKVLGSRMCRRKDIAEWKAALKLGDLSDPF

TSLLWSYEKLDPCLQRCFLYCSLFPKGHGYRPEELVHLWVAEGFIGSCN
>Q9ZTI5/1-160
LLVLDDVWFEKSHNETEWELFLAPLVSKQSGSKVLVTSRSKTLPAAICCEQEHVIHLENM
DDTEFLALFKHHAFSGAEIKDQLLRTKLEDTAEEIAKRLGQCPLAAKVLGSRLCRKKDIA
EWRAALKFGDLSDPFTSLLWSYEKLEPRLQRCFLFCLLLY
>Q8H6U9/172-453
RDQAKNQIISELIETDSQQKIVSVIGLGGSGKTTLAKLVFNDGNIIKHFEVVLWVHVSRE
FAVEKLVEKLFKAIAGDMSDHPPLQHVSRTISDKLVGKRFLAVLDDVWTEDRVEWEQFMV
HLKSGAPGSSILLTTRSRKVAEAVDSSYAYNLPFLSKEDSWKVFQQCFGIALKALDPEFL
QTGKEIVEKCGGVPLAIKVIAGVLHGIKGIEEWRSICDSNLLDVQDDEHRVFACLSLSFV
HLPDHLKPCFLHCSIFPRGYVINRRHLISQWIAHGFVPTNQA
>Q9ZTI6/1-96
LLMLDDVWTADVFFRIKEVLVDNGFGSRVIITTRIEEVASLAEDSCKIKIEPLGVDDSWH
VFCKKAFPKVENHICPPELHQCGINIVEKCDGLPLA
>B4FQI0/170-366
DKEAGEVIDALVAAADGSGARMFRAAGIAGIHGSGKTALARKVFVHDKAKDNFALRLWVC
VGPPDSEDRFNLLYRMLDNLGLDTDKVEEVVDKSSVVRDARGRTEAELRKAAARPAEQKK
AEGSVVVTEAGAGGDANRQAKDEELLKEKVENSRAVEKSKIGVLLYILNMVLSKTSYMIV
FDDIRAYRHDDDDAQGC
>B4FQI0/377-489
AEGEWGDRLAYGLPKTKHRGAVLVTCRKEDDAKTMARTGLVVRPPKLEGDDAWKLFRREY
DQAKDDKRNKDGGGKEGSKGEEEDLLLKQLQEMKKEIVGKCLGLPVAIIEAAR
>Q9ZTJ0/1-263
GVGKTTLARMVYTDRRCQKHFELRMWHCVSGNFGAASVVRSVVELATGERCDLPDAGRFW
RARLQQVVGRKRFLLVLDDVRDDEEREKWEGELKPLLCTCIGGSGSVILVTTRSQQVSAV
MGSLPSKELARLTEEDSWEFFSKKAFSRGVQERPELVAIGRRIVHVCKGLPLALSTMGGL
MSSKQEAQDWEAIAESCSSDTDTSTGSGTDDEVLSMLKLSYGHLPDEMKQCFAFCAVFPK
DHEMEKDRLIQLWMANGYVGGEG
>Q9AT73/192-477
DHIVDFLLDKTTTAQATSAKYSGLAIVGVGGMGKSTLAQYVYNDKRIEECFDVRMWVCIS
RKLDVRRHTREIMESAKKGECPRVDNLDTLQCKLRDILQESHKFLLVLDDVWFEKSDTET
EWELLLAPLVSKQPGSKVLVTTRRETLPAAVCCEQVVHLKNLDDTEFLALFKHHAFSGAE
IKDQLLHTKLEHTTEEIAKRLGQCPLAAKVLGSRLCRKKDIAEWKAALKLGDLSDPFTSL
LWSYEKLDPRLQRCFLYCSLFPKGHRYEPNQLVHLWVAEGFVGSCN
>Q93Y95/3-280
VGGLVPRMLREGKKKVDVFAIVGAVGIGKTTLAREIYNDDRMTENFPICVWVDMSKNLSE
LDFLKTIIRGAGANVGVTENKEELLILLASALSKRFLLVLDDLESPSIWDNLLKDSLGDG
VVRGRILITTRNEEVATSMKATIHHVDKMDPESAWALLCNQVDAECNSEELATLKDVGIK
IAEKCDGHPLAIKVIAGILRSRGNSKAEWEMVLNNDSWSMCPILPEVPQAVYVSYVDLSS
QLKECFLHCSLYPEEFPIQRFALVRRWIAEGIVNARDK
>B6TB31/167-330
KKCSYVRELLERDGAHRVVLIVGLSGIGKSCLARQIASDPPLSFVDGAIEIGFGRWCSRA
ACNGSRSEYHKRLARKICTFLVKIGSMTLKEETGIDLDDVCCLLQTALVGRSMLILLDDV
WEQDIVDRFTRLYDNDCRYLVTTRDEAIYEIAEAEKVEICKDDI
>Q9ZTJ5/1-172
GVGKTTVASSVCKNQKIRRTFDCHAWVTVSQTYQAEELLREIMNQLIEQRASLASGFMTM
SRMRLVEMIQNYLRDYFIVLDDVWEKDAWLFLNYAFARNNCGSKVLITTRRKDVSSLTVH
SRVIELKTLNYTESWELFCKKAFFALEGNICPKNLTSLAEKVVDKCQGLPLA
>Q8S455/191-479

RDRIVKFLLGKTTTAEASSTKYSGLAIVGLGGMGKSTLAQYVYNDKRIEECFDVRIWICI
SRKLDVHRHTREIIESAKKGECPRVDNLDTLQCKLRDILQESQKFLLVLDDVWFEKSHNE
TEWELFLAPLVSKQSGSKVLVTSRSETLPAAICCEQEHVIHLENMDDTEFLALFKHHAFS
GAEIKDQLLRMKLQDTAEEIAKRLGQCPLAAKVLGSRMCRRKDIAEWKAALKLGDLSDPF
TSLLWSYEKLDPCLQRCFLYCSLFPKGHGYRPEELVHLWVAEGFIGSCN
>Q8H6U7/172-455
RDQAKNQIISKLIETDSQQRIKIVSVIGLGGSGKTTLAKQVFNDGNIIKHFEVILWVHVS
REFAVEKLVAKLFEAIAGDMSDHLLLQHVSRTISDKLVGKRFLAVLDDVWTEDRVEWERF
MVHLKCGAPGSSILLTTRSRKVAEAVDSSYAYDLPLLSMEDSWKVFQQCFGIAMKALDPE
FLQSGIEIVEKCGGVPLAIKVIAGILHGMKGIEEWQSICNSNLLDVQDDEHRVFACLWLS
FVHLPDHLKPCFLHCSIFPRGYVINRCHLISQWIAHGFVPTNQA
>Q9AXD6/191-479
RDRIVDFLLGKTTTAEASSAKYSGLAIVGLGGMGKSTLAQYVYNDKRIEECFDIRMWVCI
SRKLDVHRHTREIMESAKKGECPRVDNLDTLQCKLRDILQESQKFLLVLDDVWFEKSHNE
TEWELFLAPLVSKQSGSKVLVTSRSKTLPAAICCEQEHVIHLENMDDTEFLALFKHHAFS
GAEIKDQLLRTKLEDTAEEIAKRLGQCPLAAKVLGSRLCRKKDIAEWKAALKLGDLSDPF
TSLLWSYEKLDPRLQRCFLYCSLFPKGHGYRPEELVHLWVAEGFVGSCN
>Q9SWT9/191-479
RDRIVDFLLGKTTTAEASSAKYSGLAIVGLGGMGKSTLAQYVYNDKRIEECFDIRMWVCI
SRKLDVHRHTREIIESAKKGECPRVDNLDTLQCKLRDILQESQKFLLVLDDVWFEKSHNE
TEWELFLAPLVSKQSGSKVLVTSRSKTLPAAICCEQEHVIHLKNMDDTEFLALFKHHAFS
GAEIKDQLLRTKLEDTAVEIAKRLGQCPLAAKVLGSRLCRKKDIAEWKAALKLGDLSDPF
TSLLWSYEKLDPRLQRCFLYCSLFPKGHRYDPNQLVIDRYLCKDCPFMK
>Q9AXD5/192-479
DHIVDFLLDKTTTAQATSAKYSGLAIVGLGGMGKSTLAQYVYNDKRIEECFDIRMWVCIS
RKLDVHRHTREIMESAKKGECPRVDNLDTLQCKLRDILQESQKFLLVLDDVWFEKSHNET
EWELFLAPLVSKQSGSKVLVTSRSKTLPAAICCEQEHVIHLENMDDTEFLALFKHHAFSG
AEIKDQLLRTKLEDTAEEIAKRLGQCPLAAKVLGSRLCRKKDIAEWKAALKLGDLSDPFT
SLLWSYEKLDPRLQRCFLYCSLFPKGHRYEPNELVHLWVAEGFVGSCN
>Q52QI0/176-459
IEDNRRRLTEWLYSDELDSTVITVSGMGGLGKTTLVTNVYEREKTNFSATAWMVVSQTYT
IEALLRKLLMKVGREEQVSPNIDKLDVHDLKENIKQKLDNRKCLIVLDDVWDQEVYLQMS
DAFQNLQSSSIIITTRKNHVAALAQPTRRPVVHPLRNTQAFDLFCRRIFYNKEDHACPSD
LVEVATNIVDRCQGLPLAIVSIACLLSSRTQTYYIWKQVYNQLRSELSKNDHIRAVLNLS
YHDLPGDLRNCFLYCSLFPEDYPIPHESLVRLWVAEGFALSKEN
>Q8H6V1/172-453
RDQAKNQIISELIETDSQQKIVSVIGLGGSGKTTLAKLVFNDGNIIKHFEVVLWVHVSRE
FAVEKLVEKLFKAIAGDMSDHPPLQHVSRTISDKLVGKRFLAVLDDVWIEDRVEWEQFMV
HLKSGAPGSSILLTTRSRKVAEAVDSSYAYNLPFLSKEDSWKVFQQCFGIALKALDPEFL
QTGKEIVEKCGGVPLAIKVIAGVLHGIKGIEEWRSICDSNLLDVQDDEHRVFACLSLSFV
HLPDHLKPCFLHCSIFPRGYVINRRHLISQWIAHGFVPTNQA
>Q7XY05/169-457
DRIVDFLLGKTTTAEASSAKYSGLAIVGLGGLGKSTLAQYVYNDKRIEECFDIRMWVCIS
RKLDVHRHTREIIESAKKGECPRVDNLDTLQCKLRDILQESQKFLLVLDDVWFEKSHNET
EWELFLAPLVSKQSGSKVLVTSRSKTLPAAICCEQEHVIHLENMDDTEFLALFKHHAFSG
AEIKDQLLRTKLEDTAEEIAKRLGQCPLAAKVLGSRLCRKKDIAEWKAALKIGDLSDPFT
SLLWSYEKLDPRLQRCFLYCSLFPKGHRFEPDELVHLWVAEGFVGSCNL
>C0P2E1/89-275
SFWDRHRRCLMIFEDVDMCRKQMLEEFLQNHRRSEAAGVVKMIVTTNNRRVANDVGTVEP

IVLRPLPCPEYWFFFKAHAFAGRDVEENPRLISAGKAIARKLNGSFFGAKIVGGLLTDHP
DPRVWCKVLRSSIGGMSPLGDGVGYISDLAENLLPGHVNMCRVTLSKDPAFPPQTATPQL
AMFKDLH
>C0P2E1/2-73
SRKERIINFLLHEDVYRRTTELGVLPIVGDSGVGKTTLVQYACDDARVRAHFPVIMLYSF
TSTYDVKNNQGT
>Q9ZTJ1/1-83
LLVLDDVWTKDRMELEQFMVLVRSGASGSRILLTTRNSDVAEAVESSYLINLPLLSLADS
WQLFIQSFGTTVEGFDREFLDVG
>C0HGU4/170-319
IDEEAKELMNNLFEDGDEPAKKIKTVSVVGFGGLGKTTLVKAVYDKVKKEFDCSAFVSIG
QKCDLKKVFKDVLYDLDKQNHENIIASEMDEKQLIDKLQEFLADKRYLVVIDDIWDISTW
KLIRCALVESNPGSRIIITTRICEVAKKSG
>Q9ZTJ4/1-96
LLILDDVWDKDAWLFLNYAFVRNNCGSKVLITTRRKDVSSLAVDQYTIELKTLQYAESWE
LFCKKAFRASKDNQCPENLRFCAEKIVARCQGLPLA
>Q9SWU0/191-479
RDRIVDFLLGKTTTAEASSAKYSGLAIVGLGGMGKSTLAQYVYNDKRIEECFDIRMWVCI
SRKLDVHRHTREIIESAKKGECPRVDNLDTLQCKLRDILQESQKFLLVLDDVWFEKSHNE
TEWELFLAPLVSKQSGSKVLVTSRSKTLPAAICCEQEHVIHLKNMDDTEFLALFKHHAFS
GAEIKDQVLRTKLEDTAVEIAKRLGQCPLAAKVLGSRLCRKKDIAEWKAALKIGDLSDPF
TSLLWSYEKLDPRLQRCFLYCSLFPKGHRYESNELVHLWVAEGFVGSCN
>Q8H6U5/172-453
RDQAKNQIISKLIETDSQQRIVSVIGLGGSGKTTLAKQVFNDGNIINHFEVLLWVHVSRE
FAVEKLVEKLFEAIAGDMSDHLPLQHVSRTISDKLVGKRFLAVLDDVWTEDRVEWERFMV
HLKSGAPGSSILLTTRSRKVAEAVDSSYAYDLPFLSKEDSWKVFQQCFGIALKALDPEFL
QAGIEIVEKCGGVPLAIKVIAGVLHGIKGIEEWRYICNSNLLDVQDDEHRVFACLLLSFV
HLPDHLKPCFLHCSIFPRGYEINRCHLISQWIAHGFVPTNQA
>Q8H6V4/172-455
RDQAKNQIISKLIETDSQQRIKIVAVIGLGGSGKTTLAKQVFNDGNIIKHFEVLLWVHVS
REFAVEKLVEKLFEAIAGHMSDHLPLQHVSRTISDKLVGKRFLAVLDDVWTEDRVEWERF
MVHLKSGAPGSSILLTTRSRKVAEAVDSSYAYDLPFLSKEDSWKVFQQCFGIAIQALDTE
FLQAGIEIVDKCGGVPLAIKVIAGVLHGMKGIEEWQSICNSNLLDVHDDEHRVFACLWLS
FVHLPDHLKPCFLHCSIFPRGYVLNRCHLISQWIAHGFIPTNQA
>Q8H6V3/172-455
RDQAKNQIISKLIETDSQQRIKIVAVIGLGGSGKTTLAKQVFNDGNIIKHFEVLLWVHVS
REFAVEKLVEKLFEAIAGHMSDHLPLQHVSRTISDKLVGKRFLAVLDDVWTEDRVEWERF
MVHLKSGAPGSSILLTTRSRKVAEAVDSSYAYDLPFLSKEDSWKVFQQCFRIAIQALDTE
FLQAGIEIVDKCGGVPLAIKVIAGVLHGMKGIEEWQSICNSNLLDVHDDEHRVFACLWLS
FVHLPDHLKPCFLHCSIFPRGYVLNRCHLISQWIAHGFIPTNQA
>Q9ZTJ3/6-168
TTLWKVDFDSAAWITVSKAYQVEDLLKQIIRGFQKSDLKGELRVDIIDMEKRSLVEIIRD
YLHGKSYVLVLDDVWGVDIWFKIRDAFPTNSTSRFIITSRIHEVALLANGNCIIELKPLE
AHHSWELFCKEAFWKNENKMCPLELNNLAQRFVDKCNGLPLAL
>B7ZZ31/160-454
RGEECNSIISRLVDDCEETCRPVSPVIAVVGHGGIGKTTVAQCVYNDARVEARFDLRAWV
CVWDRSDEAELTREILQSIGCADDKPCDDGLASLDSLQEKFENLVARKRFLLVLDDVWID
EGKTEKENRSIWNRVLVPLRSATTGSKVLLTTRMKLVAEVLNAGYLVSLDGLRSSDCWLL
LKEVALGGETMDFPPELQEVAGTLVAMVKGSPLAAKAIGQMARSTRSTRKWRTLVNTEIS

NDIIISSIQLSYKHLPGHLQRCFAYCSIFPSTWRFSRSQLVNMWIALGFIQSSAE
>C0P8B0/171-456
FDGKVEDVSKTVMDAGGSNGLRIVSIVGMAGSGKTTLANAVYRRLQADNTFQCSAFVSIG
PKPDMVKTVKDMLSRLGDGHRGGEDISQLIPRVRGILEKKRYLAWIDDIWSSEQWGVIRC
CFPDNSLGSRIITTSRNDALPTNHHYGSSKFVYKIGLLTDNEARELFLKKAFSSRNDCPQ
HLVDAFTKVLRRCAGLPLAVVSVAAKLAHKQSREEWEKHGLNLLYSSHSDGSDGLKQILH
LSYSDLQPQLRSCLLYLSIFPENSEVETDRLVRRWIAEGLIAASNE
>Q8H6U8/172-453
RDQAKNQIISKLIETDSQQRIVSVIGLGGSGKTTLAKQVFNDGNIINHFEVLLWVHVSRE
FAVEKLVEKLFEAIAGDMSDHLPLQHVSRTISDKLVGKRFLAVLDDVWTEDRVEWERFMV
HLKSGAPGSSILLTTRSRKVAEAVDSSYAYDLPFLSKEDSWKVFQQCFGIALKALDPEFL
QAGIEIVEKCGGVPLAIKVIAGVLHGIKGIEEWRYICNSNLLDVQDDEHRVFACLLLSFV
HLPDHLKPCFLHCSIFPRGYEINRCHLISQWIAHGFVPTNQA
>Q6PW75/172-455
RDQAKNQIISKLIETDSQQRIKIVSVIGLGGSGKTTLAKQVFNDGNIIKHFEVILWVHVS
REFAVEKLVAKLFEAIAGDMSDHLLLQHVSRTISDKLVGKRFLAVLDDVWTEDRVEWERF
MVHLKCGAPGSSILLTTRSRKVAEAVDSSYAYDLPLLSMEDSWKVFQQCFGIAMKALDPE
FLQSGIEIVEKCGGVPLAIKVIAGILHGMKGIEEWQSICNSNLLDVQDDEHRVFACLWLS
FVHLPDHLKPCFLHCSIFPRGYVINRCHLISQWIAHGFVPTNQA
>Q6PT59/191-479
RDRIVDFLLGKTTTAEASSAKYSGLAIVGLGGMGKSTLAQYVYNDKRIEECFDIRMWVCI
SRKLDVHRHTREIIESAKKGECPRVDNLDTLQCKLRDILQESQKFLLVLDDVWFEKSHNE
TEWELFLAPLVSKQSGSKVLVTSRSKTLPAAICCEQEHVIHLKNMDDTEFLALFKHHAFS
GAEIKDQVLRTKLEDTAVEIAKRLGQCPLAAKVLGSRLCRKKDIAEWKAALKIGDLSDPF
TSLLWSYEKLDPRLQRCFLYCSLFPKGHRYESNELVHLWVAEGFVGSCN
>Q9AT65/191-477
RDRIVDFLLGKTTTAEASSAKYSGLAIVGLGGMGKSTLAQYVYNDKRIEECFDIRMWVCI
SRKLDVHRHTREIIESAKKGECPRVDNLDTLQCKLRDILQESQKFLLVLDDVWFEKSDTE
TEWELLLAPLVSKQSGSKVLVTTRRETLPAAVCCEQVVHLKNLDDTELLALFKHHAFSGA
EIKDQLLHTKFEHTTEEIAKRLGQCPLAAKVLGSRLCRKKDIAEWKAALKLGDLSDPFTS
LLWSYEKLDPRLQRCFLYCSLFPKGHRYDPNQLVHLWVAEGFVGSCN
>Q9ZTJ2/1-254
GVGKTTLAKQVFNDGNIINHFEVLLWVHVSREFAVEKLVEKLFEAIAGDMSDHLPLQHVS
RTISDKLVGKRFLAVLDDVWTEDRVEWERFMVHLKSGAPGSSILLTTRSRKVAEAVDSSY
AYDLPFLSKEDSWKVFQQCFGIALKALDPEFLQAGIEIVEKCGGVPLAIKVIAGVLHGIK
GIEEWRYICNSNLLDVQDDEHRVFACLLLSFVHLPDHLKPCFLHCSIFPRGYEINRCHLI
SQWIAHGFVPTNQA
>B4FEU1/158-361
EDLDELLDLLVRRPDNELDKVLKVMVISVVGFGGIGETKLCHTVYTDVQESRRFSLHAYV
SAAGKDCSIVLEEIIEQFRLQEDPQDSSGGFFHRFAGAFPGARRTDQVHGLPEYLQRKRY
FVVVDGVESEELVSGIASAFPDNSMGSRIIMGMRTAVGRDAERCVGHRHKMWPLEDKQSV
VCFLNEAERRRRRHEQDLSDFAVD
>Q8S453/191-479
RDRIVKFLLGKTTTAEASSTKYSGLAIVGLGGMGKSTLAQYVYNDKRIEECFDVRIWICI
SRKLDVHRHTREIIESAKKGECPRVDNLDTLQCKLRDILQESQKFLLVLDDVWFEKSHNE
TEWELFLAPLVSKQSGSKVLVTSRSETLPAAICCEQEHVIHLENMDDTEFLALFKHHAFS
GAEIKDQLLRMKLQDTAEEIAKRLGQCPLAAKVLGSRMCRRKDIAEWKAALKLGDLSDPF
TSLLWSYEKLDPCLQRCFLYCSLFPKGHGYRPEELVHLWVAEGFIGSCN
>A9RME3/534-819

REQYLSRIKKECVNKMKVLCLIGMGGIGKTTIAKAMLADVKDIYDASCFVECIENGVDCF
TTSCNILEQFKVKSKPRNVEEAQKMLKSFLMKNKTIFVFDNVKNQSQIEDVVPMDDIYAS
NGSTLVTTTRDSKTIEHYGKEVCIINIEELNEETSMKLFNTHSCGQENLPNELVEVGEKI
VKACHGLPLSLKVMGAFLRENKRLRCWERALQKLKRGRELDGDENNSNYKIWKILRVSFD
NLKDEEKKMFMDICCFFSSDVYPQGMSKGRALRMWANSQKNIFEQD
>A9TGN5/4-225
LKNDYLVELNKILKSLDVKDEAKDLIEGKQLLAKELHIKNYLLILDDIPNFEVLQQLIDV
TMFDKKRSKLIVTSRKSDVLKNYISEGGKMELLSLTKNEAMEIFSKHAFEDECSKIPYLD
KISIEIVDACSGHPLSLEIVGSSLNGQTRIRVWEQALQRLKRAMICHNSNDIWTRLKPYF
DDLNNKEKTMFLDIACFFSKDIWSEIIPKHTIIHFYEYEIEN
>A9T452/1-176
KCKAMNLEEGKQILIKKLQEKRLLIIFDDVLKHEDMKKVVDIAMLGIPRSKFIVISREWN
ILKKFISESGKIELSPLDESTSMKILLENIFKDGPFYMPHLYEFSTEISKACDGHSLSLE
FVGSSLKGQTRLRVWEQTLHRLKRANIYVDGEDLLARLKPSFDDLGEEEKIIFLDL
>A9T3B5/545-828
QYLSQIRKLCANKVKVLYLVGMGGIGKTTIAKTTLINVKNMYNASCFVECIESGGDCYTT
SCNILEQFQVKEKPKDVKEAHKILKSFLTKNKTILVFDNIKNQSQIEDVVPMDDIFASNG
STLIATTRDSNVMKDCGKEIYKINIEELDEETSMKMFITHSCGQKSLPIELVEVGKKIVR
ACNGLPLSLKVMGAFLREKKRLRCWERALQKLKRGRKLDGDENNSNYKIWKILRVSFDNL
KVEEKKMFLDICCFFCNDVCPQGMSKERALRIWVNNQNNILEHE
>A9T1I0/321-521
LETKFHGKCQNYLQKEVRRKETFLAIDNVSPETATQASVYLDFGFHENSVVLVTARTRSI
LTSHLQIDERDCMEMPDLEKNEATRLFLKRVGLGDDSVWIKDHDNIVQKCVEECLYSKGD
DADHHYHPLSLLVLGGQLRSVDPSKWQEILAEEPDIKFNLSGEMPHPVFSIFERSYYSLR
RAEQLLFMDVALFTPERYLTL
>A9RMM6/538-824
RQEYSNKLLELCKNGVKAICLIGMGGIGKTTMAKFMYNDVKGMYDASCFVENMQRSENSY
TICCYILKELKVNQIPETLEDAQVLLKSHLRSKKTILVFDDVKEQSKIRDIVPMDEFCTS
NGSTLIVTTRSWNCMKDCSIKTHRVDIAELEEGASLELFISYSHGKQVELPRELDDVGKK
IVKACNGLPLSLKVMGAFLRGQKRVRCWERALQRLKRARNVDGDEENSDHKIWTILRISF
DQLKVKEKDIFLDICCFFTSDVYPHGMSKERALRIWTNNEKLGLEQD
>A9RYV0/1-242
MKALCLIGVGGISKTIIAKAILVNIKDIYNASCFDECIENGVDCFTTSCNILEQFNVKSK
PKNVEEAQKILKSFLMENKTILVFDNVINQSQIEDVPMHDIFSNNDSTLVVTTRDPKVIK
DCTKEVCIINIEELDEEISMQLSIIHSCDKDYLPNKLVKIGEKIVKACNGLPLSLKVMEA
FLRKNKRLRCREHALQKLKKKRELDGDKNNSNYKIWKILRVSFDNLKDEEKKIYYFFCSH
VS
>A9T4B7/179-451
DQQASEFLSFIRSQSQKSCRIGIFGMGGIGKTTLAKEIFKRIKDEFPVSSFLEDVARETS
KGDQGVASLQLQLLRDMGNADRVNIVEDINDGKSKLRQCLQSKRAFIVLDNVESPLSIKA
LCVDENLGVGSCCILTSRDEWICSVFSDFTYEMPFLKPAEAKQLFCWNAFGSIFAAQGFQ
ELANEVALACGGHPLTLELMGSLLRREKDLLVWDAVLQHLRKHDSLQNHDKMLQRLKISF
DSLEPRHKEMFLDVACFLLGSPPQLCKDLWTSL
>A9RKC9/2-182
GPVLIVLDGVHKIGQFEFIIPFAKDLHAGSRIIITSRDGSIFNNIAGRAGSVVRRLFMVS
KLGPSNSNKLFNWHAFQAEEAPEDYRELAEDIVKSCGGLPLALKVFGSSLCNRTADRDRE
RIWPEAVRALRQSGDVMDVLRWSYDILTESEKHMFVDITCLFYGRPLKEAKPYWKSCEDC
A
>A9RB93/1-170

GGAGKTLAAQRVFDDDAIRAHFTGGCFWFTVGGDLSVRSLFLALGEKITGPSEVRENIPM
EDLTAQLRNELKDKKNILVVLDDVWKEDVLRLVTCVVPLRAGCKILVTTRIEEVLDKNHV
TKLPFVLLDEENSWKLFCWQAFRGTSHVPPELEKLAKDVMGECGGLPLAL
>A9U4D4/543-828
REQYLSRIKKECVNKMKVLCLVGMGGIGKTTLAKAILAIVKDIYDASCFVECIENGVDCF
TTSCNILEQFKVKSKPKDVEEAQKMLKSFLMKNKTIFVFDNVKNQSQIEDVVPIDDIYAS
NGSTLVLTTRDSKAIEYCGEEVCIINIEELDEETSMKLFITHSCGQENLPNELVEVGEKI
VRACHGLPLSLKVMGAFLRENKRLRCWERALQKLKRGRELDGSENNSNYKIWKILRVSFD
NLKDEEKKIFLDICCFFSSDVYPQGMSKERALRMWANSQKEIFEQD
>A9RWI1/188-462
VLLEGTLGLLEEMEDKVGILGICGIGGIGKTTLAQRVYNLYSTARGFTKHTFLKLEASQD
MKLFQAQVLRDLLNKNEKNLDEEYVNHFEGLAGVKVLVVLDNICSVDHFKELVPNMLKLG
GGSRIILTSRERDTMRAIMNVEPSCAFCPLDMKKLGHEDSFQLFWLHAFQNRDVSTAEGN
VFRPLAAKVVKLCGGLPLALKVIGQHLYGKSEEVWSEASETLRDRPDIIDVLSISYKGLR
DPDKMMFLDVSCQMVGLLEEDAIDIWKSCRSCPSL
>A9T0B5/1-179
MGGIGKTTLAQEVYNHYSTGGKFLYQTFLKIGDIKDTSHLRKQALRDLLNKKEKNLDERY
VNHFEGLAGVKVLVVLDDISNVEHFKELVPNMKKLGVGSRIILTSRERDTMRAIMSEAPS
RFGSYLHEMKPLEATDSFNLFWFHAFHDRELSKAEDEMFRPLALMVVNNLCGGLPLALK
>A9TUP2/49-334
DEVVDEAKQLLLDENTNKLGLWASGGTGKTLAAQRVFNDDAIQAHFTGGCFWLTVGRALS
LGSLLHNLRFSITGLSDARRKIPIEDLSNQLCKELQNKKNMLVVLDDVWEEHMLRSVELV
VPLRAGCKIFVTTRIDQVLNKNHVTKLPVPLLSSENSRKLFCWQAFRGTALVPPELKKLA
QDVTAECGGLPLALKVIGSVFAGKTDRGFWELSLKKLRNADVLDQDHETQLYNRLKLSVD
ELASIHPRLKDCFLYFAAFPEDWKVQVYDDLLPLWTGERIVGENSQ
>A9TC59/262-546
MRILAKVEKTCPSRRDGGRVVGLYGAGGIGKTTFCKELLDELETKFHGKVCHMEIGSHTH
EKFLQLLIRCLTETDSRRGFENWDHGQCQNYLQKEVRRKETFLAIDNVSPETATQASVYL
DFGFHENSVVLVTARTRSILTSHLQIDERDCMEMPDLEKNEATRLFLKRVGLGDDSVWIK
DHDNIVQKCVEECLYSKGDDADHHYHPLSLLVLGGQLRSVDPSKWQEILAEEPDIKFNLS
GEMPHPVFSIFERSYYSLGRAEQLLFMDVAFFTPERYLHLKLLNT
>A9RFS0/315-521
QSSLQSTIVELEQKLFQNYIVGVVGIPGIGKTTLVRNFQVYIERNISTKSISSLYGKDSK
TETSFDDISYLQIIIMWDLKNDYLVELKKILKSLDIKVKAKDLIEEKKLLAKEIHVKNYL
LILDDVPNFEILQQLIDITMFDKKKSKLIVTSYKWDVLKNYVSEGDKMELLSLTEIEAME
IFSTHAFQDKCSKTSYLYKMAIEMVKA
>A9RG57/234-432
WERICKVLSGLFVADEGVEKLGIYGMGGLGKTTICKGMCNYFHGHFFGRVFHLELVSGRK
QLDLQKQMLKTLFRLGDDILQTVASSDEVVTCLNKHAGTHPVLLIIDNVQDDHDSQEEAR
GYLKAKFCEGSKVVITSRSRVALEKLLPGPEFRKAMPELERDEAARIFLRRAVRHEKRLF
SLDPRENKVLDTCLEQCSF
>A9RB86/1-177
MVGFWASGGAGKTLAAQRVFDDDAIRAHFTGGCFWFTVGGDLSVRSLFLDLGKKINGPSE
VRRNISMEDLTAQLCNELKDKKHMLVVLDDVWKEDVLRLVTCVVPLRAGCKILVTTRIEE
VLDKNHATKLAFVLLDEENSWKLFCWQAFRGTSHVPPELEKLAKDVMGECGGLPLAL
>A9RZ21/543-804
AQYLSQIRKLCANEVKVLYLVGIGGIGKTTIAKATLINVKNMYNASCFVECIDSGSDCYT
TSCNILEQFQVKEKPKDAKEAHKMLKSFLTKNKIILVFDNIKNQSQIEDVVPMDDIFASN
GSTLIATTRDSNIMKDCGKEICKLNIEELDEETSMKMFTTHSCGQESLPIELIEVGKKIV

RACNGLPLSLKVMGAFLREKKRLRCWERALQKLKRGRKLDGDENNSNYKIWKILRVSFET
LKIEEKKNVFGHMLHVKRKSFA
>A9RYK4/1-116
IYKMELLNEVEALRLFLFYTFQGANDNKCKQLNDQVKAIIKACGALPLSLEVIGQYLKKY
NLKDIDERKEIWKEALKRLEEAKPFDGYNDDEMLWKRLRISYDNLAMDEKSIFLDF
>A9TD29/1-179
IIFDDVLNHEDMKKVVDITMISVRKSKFIVTSHEWNILKKFLSESSKIELSLLNESSSME
IFFKHAFKDGPFYMPHLYEFSKEVINACAGYPLNLEIVDSSLKGQTRLRVWEQALQRLKR
ASTYLDNKDLLAMIRPSFDDLRDEEKIIFLDLTCFFSKDVWIEDINQDTFFQFYEYEFE
>A9U5R0/6-230
DAKVSDLYEKLGRCCILDNGVSEKFKLIKNQEEQRTFLKNAYAEKKVFLILDDVWEKPKY
DHHDMLYWLDISRGPGSATLITSRSKSVLCKVQAKVEVVLSLPKEESWKLFCCHAFESDG
ALPTTLEELARDVCEECKGLPLALKVIGSAMADKATVAEWRCALHDLRRSKPIVDSNVDD
ELFGRLELSYKELKDDATRICFLYFAAFPEDYEIEADKLLRMWVA
>A9RB87/1-110
MEDLTAQLRNELKDKKNMLVVLDDVWKEDVLRLVISVVPLRAGCKILVTTRIEEVLDKNH
VTKLAFVLLDEENSWKLFCWQAFRGTSHVPPELEKLAKDVMGECGGLPLA
>A9SRB1/375-659
KQCLLQISNLCASKVKALCLIGMGGIGKTTIAKTTLTNVKHMYDASCFVECDESGNDCYK
SSCHILEQLKVKEKPKDLKEAQEMLKLVLTKKRVIIVFDNVTNQSQIEAVVPIDDIFAMS
GNTLIVTTRDWKVIEYSDIKLCKVNIEELDEETSLRLFITHSCGHEGKLSSELVEVGKKI
VKACNGLPLSLKVMGAYLRDKKRLRCWERSFQRLKRGRQLDGDEKNSDHKIWDILRVSFD
NLRVEEKRMFLDICCFFNNDVFPQGILKERALRIWSNIEEKRMED
>A9TDN3/279-637
ICRRILGKFEKTCESLRNGVRVVGLYGTGGIGKTTFCKVLCNELDKKFHGKVCHAELGNP
TLELLKVVIKTLTDTAKQQFEHWTEDQTIWKAGMWYKVRAIVFHVEHSVQVEVDIAAQSG
HKLSPSFLRGDKQPYWLNDLWHSHSGSRWGMQCRNYLKHEVRRRATFLALDNVSIESLDQ
AKMYLDADFHKDSMVLVTARSLSILTSHLKINESHCMEMPELEREEATKLFLNHAAPDYD
SFGDKDHDNDIQRCVEECLYSKGENFGSHYHPLALKVLGEQLRSSDPSEWQDVLDEEPDK
FNLLGETTHRVFSVLKRSYNSLREDDQLFFMDVALYTPEKYIFLEVPGNIFYWLSMVHN
>A9T3B1/552-835
QYLSQIRKLCANKVKVLYLVGMGGIGKTTIAKTTLINVKNMYNASCFVECIESGGDCYTT
SCNILEQFQVKEKPKDVKEAHKILKSFLTKNKIILVFDNIKNQSQIEDVVPMDDIFASIG
STLIATTRDSNVMKDCGKEIHKINIEELDEETSMKMFITHSCGQESLPIELIEVGKRIVR
ACNGLPLSLKVMGAFLREKKRLRCWERALQKLKRGRKLDGDENNSNYKIWKILRVSFDNL
KVEEKKMFLDICCFFCNDVCPQGMSKGRALRIWINNQKNILEHE
>A9RBA9/1-184
LILDDVSSEDILHELIDVTMFGTNRSKLIATSRTWEVLKNIVNERNKIELISLKDNEAME
IFSKYAFQDGRPNIPHLSKISMAVVKACVGHPLTLQIVGSSLHNQTSLCVWELVIQRLKR
ASKFLGSNNPWTRLEPIYEALNDEEKTMFLDIACFHCKDIWNENINEDIIIQFYKYEFKN
AKEI
>A9SMJ1/6-237
DAKVSDLYEKLGRCCILDNRVSEKFKLIKNQEEQRTFLKNAYAKKKVFLILDDVWEKPKY
DHHDMLFWVDIARGQGSATLITSRSKSVLCKVQAKVEVVLSLPKEESWKLFCCHAFESGG
ALLTTLEELAWDVCEECKGLPLALKVIGSAMADKATVAEWRCALHDLRRSKPIVDSNVDD
ELFGRLELSYKELKDDATRICFLYFAAFPEDYEIEADELLRMWVAEKLFGLD
>A9RB95/1-177
MVGFRASGGAGKTLAAQRVFDDDAIRAHFTGGCFWFTVGGDLSVRSLFLALGEKITGPSE
VRENIPMEDLTAQLRNELKDKKNMPVVLDDVWKEDVLRLVTCVVPLRAGCKILVTTRIEE

VLDKNHVTKLPFVLLDEENSWKLFCWQAFRGTSHVPPELEKLAKDVMGECGGLPLAL
>A9TIY4/4-213
FGNKIEDQNLIEEKSFLTNILQKKNIILILDDVSSQDILPKLIDVTKFGTNRSKLIATSR
TWKVLKNTINERSKIELDSLEDNEAIEIFSKYAFKDGRPNMPHLSKMSMVVVKACAGHPL
TLQILGSSLHNQTSLCVWELNIQRLKRASKFLGSNDLWARLEPTYKDLNGEEKTMFLDIA
CFHCKDIWHENINQDTIIQFYEYEFKNAKE
>A9RED8/550-836
RTQHLAQIRELCASNMKALCLVGMGGIGKTTIAKATLNSVKHMYDASCFVECIESGGDCY
KMCCNILEQLEGEKMPKDLKDAQEMLKSVLTKKKVILVFDNVKNKRQVEDVVSMDDIFAS
NGSTLIMTTRDWKIMEHCDTKICMVNVEELDEETSLRLFVTYSCGFQDKLPHELVEVGKK
IVKACNGLPLSLKVMGAFLREKKRLRYWERALQRLKRGRELDRDEENSDHKIWNILRVSF
DNLKVEEKNMFLDICCFFCNDVCSQGMLKERAIRVWTRNKEMIYEED
>A9THG7/2-187
LRIGPAARVIAVLGPGGVGKSTICKALYEHVSHLFTAVSYVEDVKEKSKHPRGLVRMQQQ
IIHDLCKLNDVHIDNPRHGQALIKEHLRGSVKVLIIILDDIDDCMQVENLMLPDALGYGSR
GIVATRDKTVVAQLGIHDTYEVRELTELEAVELFFWHAFMQQKPPHSFDKIATEVAVACG
RIPLEI
>A9RQA0/1-219
MKDMYDTSCFVECDESSSDCYKSLCHILEQLKVEAKPKDLKEAQEILKLFMIKKSVIIVF
DNVIKQSQIEDVVHLDDIFAMSGSTLIVTTRDWEVMEYCGIEHCKLNIEELDEETSLKFF
ITHSCGYEDKLHSELVVVGKKIVKAYNCLPLSLKVRGTYLRDKKKLRCWERVFQRLKRDR
QLDGDEKNSDYKIWDILRVSFDNLRVEEKKMFLDICCFF
>A9SDX0/87-168
KRVHFTRDARQNIPMDDMKTELSEDLKDKKNMLVVLDDVWEENVLRSVASVVLSRAGCKI
LVTTCIELSLMLTKLKEQPAKL
>A9SYD0/129-278
LEWLDIAKGPGSVTLVTSRKQSVLQRANAAEFVVLCLNEEDSWKLFSAHAFGGVGKCPNE
LETVAKLVARECKGLPSALKGIGVAMVGKTRVGHWNLDLQKLRDSCVLDQNVEVQLFHRM
KLSYDELDTIDSQYATVDAQYLLWYWIGEK
>A9SYD0/61-137
DDITTRLKALVLEGQEMGPRAVGIWGKGGAGKRLLAQRIHNDRQVQQHYKTSIIWLTIGR
DASINALLLEWLDIAKG
>A9RFR9/1-90
PLNLEIVGSSLNGQTRIRVWEQALLRLKRAMICHNSNDIRTRLKPYFDDLNNEEKTIFLD
IACFFSKDTKKYESILKHAIIHFYEYEIEN
>A9RB44/1-170
GGAGKTLAAQRVFDDDAIRAHFTGGCFWFTVGGDLSVRSLFLDLGMKITGPSEVRRNIPM
EDLTAQLRNELKDKKNMLVVLDDVWKEDVLRLVTSVVPLRAGCKILVTTRIEEVLDKNYV
TKLAFVLLDEENSWKMFCWQAFRGTSHVPPELEKLAKDVMGECKGLPLAL
>A9SR92/566-850
NQCLLQISNLCISKVKALCLVGMGGIGKTTIAKTTLNNVKHMYDASCFIECDESTSDCYQ
SLCHILEQLKVEAKPKDLKESQKMLKLFLSRKRVIIVFDNVINQNQIEDVVPMDDLFSMS
GSTLIVTTQNGEVMEHCGIELCKINIEELDEETRLRLFITHSCGHEGKLPNELVEVGKKI
VKACNGLPLSLKVMGAYLRDKKRLRCWERAFQRLKRGRELDGDEKNSDYKIWNILRVSFD
NLRVEEKKMFLDICCFFNNDVFLEGMLKERALRIWSNIEEKRAKE
>A9SRP2/14-145
DGNVEETNKLLLDQNTYMVGFWASSGAKKTLVAQRVFDDDAIRAHFTGGCFWFIVCRDLL
VRSLFLDLKMKITGPSKIRGNIPIEDLATQLRNELKDKKNMLKLRNADVLDKGYKTQLFN
RLKLSFDELGTK

```
>A9TL25/1-105
RGCNGLPLSLKVMGAFLRKNKRLRYWERTLQKLKKERELDGDENNSNYMIWKILRVSFDN
LKVKEKNMFLDICCFFCKDVCPQGMSKERALRIWTNSKKNIFEQD
>A9RB89/1-177
MVGFRASGGAGKTLAAQRVFDDDAIRAHFTGGCFWFTVGGDLSVRSLFLDIGKKITGRSE
VRRNIPMEDLTAQLRNELKDKKNMLVVLDDVWKEDVLRLVTCVVPLRAGCKILVTTRIEE
VLDKNHVTKLAFVLLDEENSWKLFCWQAFRGTSHVPPELEKLAKDVMGECGGLPLAL
>A9SRA0/1-213
MYDASCFVECDGSGSDCYKRSCHILEQLKVEAKPKDLKDAQEMLKLFLIKKRVIIIFDNV
TNKSQIEAVVPMDDLFSMSGSTLIVTTRNGEVMEHCGIELCKINIEELDEETSLRLFITH
SCGHEGKLPNELVEVGKKIVKACNGLSLSLKVMGAYLRDKKRLRCWERAFQRLKRGRELD
GDEKNSDYKIWNILRVSFDNLRVEEKKMFLDIC
>A9SPW6/1-166
VDDIDDVGQFENLVPAIEMLGSGSRMVITSRNREALTLATRPAVCKDVYEVKMLNCSGSR
DLFNWHAFLSETPSECFSDSAAAVADACGGHPLALKLMGCMLPWRKRNWEGGNLKENKTL
SDKLLISYDCLGDRKKAIFREKAYLMEGLLEDVAVAIWRSCERCSP
>A9RKC8/1-177
MIVIDDVHKFNQFEALIPFASDLRIIVTSRDWSLLKDVVGRTNLDHHKFDVPTLESHESN
MLFLRHAFNSDKALEGYQELASNVVDACGGLPLALKVIGSILFDKRDNEARDTIWQETSQ
GDVLIESDVIEVLRWSYDSLSESEKLMFLDITCLFHDKCTKDLALAVWESCEVCISC
```
