# Supplementary material for: Diversity and Variability of NOD-Like Receptors in Fungi
Source: Genome Biol Evol. 2014 Dec 8;6(12):3137–58. doi: 10.1093/gbe/evu251 (PMC4986451; doi:10.1093/gbe/evu251)
Supplement: Supplementary Data [file supp_evu251_supplementary_file_S3._Full_list_of_domain_annotations.pdf]

Supplementary file S3: Full list of domain annotations

N-terminus

All domains other than highlighted in yellow or gray (UNK) were included in the OTHER category.

Annotation	#paralogs	#orthologs
**Ascomycetes**		
AA_PERMEASE HELO-LIKE	1	1
ACETYLTRANSF HELO-LIKE	1	1
ALDO_KET_RED SESB-LIKE	1	1
AMIDOHYDRO GOODBYE-LIKE	1	1
AMIDOHYDRO HELO-LIKE	1	1
AMINO_OXIDASE HELO-LIKE	1	1
ANK SESB-LIKE	1	1
ARF SESB-LIKE	1	1
ASP HET	1	1
ASPZINCIN_M35	1	0.666667
CU-OXIDASE HELO-LIKE	1	1
CVNH	1	1
CVNH PNP_UDP	1	1
DAO HELO-LIKE	1	1
DAO PNP_UDP	1	0.666667
DCMP_CYT_DEAM SESB-LIKE	1	1
DDE HET	1	0.5
DUF2841	1	1
DUF3435 PNP_UDP	1	0.25
DUF3632 GOODBYE-LIKE	1	0.333333
EXO_ENDO_PHOS	1	1
FAD_BINDING BBE	1	1
FAD_BINDING BBE HELO-LIKE	1	1
FRUCTOSAMIN_KIN SESB-LIKE	1	0.666667
FUNGAL_TRANS SESB-LIKE	1	1
GLYCO_HYDRO HELO-LIKE	1	1
GLYCO_HYDRO PNP_UDP	1	1
GLYCO_HYDRO_N GLYCO_HYDRO GLYCO_HYDRO_C BGAL_SMALL_N PNP_UDP	1	1
GOODBYE-LIKE	75	598.25
HELO	26	39.3333

HELO-LIKE	69	428.5
HELO-LIKE PFD-LIKE	1	1
HET	21	100.833
LACTAMASE_B	1	1
LAMB_YCSF PNP_UDP	1	1
METHYLTRANSF_PK GOODBYE-LIKE	1	0.333333
MFS	1	1
MFS GOODBYE-LIKE	1	1
MFS SESB-LIKE	1	0.666667
MOSC_N MOSC HELO-LIKE	1	1
NAD_BINDING PNP_UDP	1	1
P450	1	1
P450 PNP_UDP	1	1
P450 SESB-LIKE	1	1
PATATIN	26	35.6667
PEPTIDASE_S8	6	6
PFD-LIKE	17	35.5
PIF1 PNP_UDP	1	1
PKINASE	4	3.33333
PKINASE HELO	1	1
PKINASE HELO-LIKE	1	1
PNP_UDP	50	380.417
PNP_UDP GOODBYE-LIKE	5	6
PNP_UDP HELO-LIKE	1	1
RELA_SPOT	5	15.6667
RER1	1	1
RNASE_H HET	1	1
SESB-LIKE	75	406.333
SESB-LIKE GOODBYE-LIKE	1	0.5
SUGAR_TR SESB-LIKE	1	1
TERPENE_SYNTH_C PNP_UDP	1	1
UNK	78	1266.58
WD40 ANK CORA	1	1
ZN_CLUS FUNGAL_TRANS HELO-LIKE	1	1
ZN_CLUS HELO-LIKE	1	1
**Basidiomycetes**		
ABM	1	0.5

AMIDASE	1	0.5
ATP-SYNT_EPS	1	1
C2	3	9
DUF4646	1	1
GAR1	1	0.5
GOODBYE-LIKE	13	64
GOODBYE-LIKE HELO-LIKE	1	1
HELO-LIKE	4	24
HNH	1	0.5
METHYLTRANSF	1	1
PATATIN	5	16.5
PKINASE GOODBYE-LIKE	1	1
PKINASE_TYR PATATIN	1	0.5
SESB-LIKE	1	45
SMC_N SMC_HINGE	1	0.5
TPR	1	1
UNK	21	1135
WD40	1	1
ZF-GRF C2	1	1

Nucleotide Binding Domain

All domains other than highlighted in yellow or gray (UNK) were included in the OTHER category. In a few cases marked with an asterisk (*) incorrect annotations were assigned due to overstretched domain boundaries. These errors were corrected manually for the association diagrams only.

Annotation	#paralogs	#orthologs
**Ascomycetes**		
AAA	33	62.5
*ANK	2	1.5
ARCH_ATPASE	1	1
CDC37_M	1	1
KAP_NTPASE	3	2.33333
NACHT	80	1884.67
*NACHT ZZ	1	1
NB-ARC	76	445.333
*NB-ARC TPR	2	1.33333
*PFD-LIKE NB-ARC	1	1
*TPR	2	2
UNK	79	966.333
**Basidiomycetes**		
AAA	7	13.5
NACHT	20	891.5
*NACHT ANK	1	0.5
NB-ARC	13	121
UNK	20	277
*WD40	1	1

C-terminus

All domains other than highlighted in pale orange (ANK), pale yellow (WD40), pale green (TPR) or gray (UNK) were included in the OTHER category. Note that unlike the case of N-terminus and NBD, C-terminal domains were assigned the three main repeat categories also when the repeat annotation was accompanied by hits from other non-repeat signatures. In this table an asterisk (*) marks C-terminal domain architectures consisting of repeats from two categories.

Annotation	#orthologs	#paralogs
**Ascomycetes**		
3HCDH_N 3HCDH	1	1
AA_TRANS	1	1
ACATN	1	1
ALPHA_KINASE	1	1
AMINOTRAN	2	2
ANK	76	920.083
ANK AA_PERMEASE	1	1
ANK ADH_SHORT	1	1
ANK CORA	1	1
ANK DUF3669	1	1
ANK EXO_ENDO_PHOS RVT	1	1
ANK F-BOX-LIKE	1	1
ANK GFA	1	1
ANK GLYCO_TRANSF	2	2
ANK HELO	1	1
ANK HET	1	1
ANK METHYLTRANSF	1	1
ANK MFS	1	1
ANK MULE	1	1
ANK PIF1	1	1
ANK PKINASE	8	9
ANK RVT	1	1
ANK SPRY	7	13.3333
*ANK WD40	4	4
ANK ZZ	1	1
ARID	1	0.333333
BETA-LACTAMASE	1	0.333333
CDC37_C	1	1
DDE_TNP	1	1

DNAJ	1	1
DUF1765	1	1
EXO_ENDO_PHOS RVT	1	1
FAD_BINDING BBE	1	1
FAD_BINDING PHE_HYDROX_DIM	1	1
GLYCO_HYDRO GLYCO_HYDRO_C PA14 FN3-LIKE	1	1
GLYOXALASE	1	1
HET	1	0.666667
LYSM	1	1
METHYLTRANSF	1	1
MFS	2	2
NAD_BINDING	1	1
PEPTIDASE_C14	1	1
PEPTIDASE_S8	1	1
PKINASE	3	2.33333
PNP_UDP	1	0.333333
PRO-KUMA_ACTIV PEPTIDASE_S8	1	1
RNASE_H	1	1
RVE RVT	1	1
SESB-LIKE	1	1
SPRY	1	1
SUGAR_TR	1	0.333333
TPR	75	488.583
TPR ACT	1	1
*TPR ANK	1	1
TPR COLLAGEN	1	1
TPR CYS_MET_META_PP	1	1
TPR DDE	1	0.333333
TPR FMN_BIND	1	1
TPR HTH DDE	1	1
*TPR WD40	1	1
UNK	79	1494.92
WD40	60	346.25
*WD40 ANK	6	6
WD40 DUF202	1	1
WD40 MFS	1	1
WD40 RVT RNASE_H	1	1

ZF-C2H2	12	11.5
ZF-C2H2 ZF-H2C2	5	5
ZF-H2C2	10	9.66667
ZF-H2C2 ZF-C2H2	1	1
ZZ ANK	2	2
**Basidiomycetes**		
ANK	7	73
ANK DOT1	1	1
CHAT	1	1
DNAJ	1	0.5
FHA	1	1
NAD_BINDING PYR_REDOX	1	1
PKINASE_TYR	1	1
THIF	1	1
TPR	12	113.5
TPR AHPC-TSA	1	1
TPR DUF4470	1	0.5
TPR PKINASE_TYR	1	0.5
TYROSINASE	1	0.5
UNK	21	737
WD40	17	368
WD40 HIS_PHOS	1	0.5
WD40 OTCACE_N	1	1
WD40 PREFOLDIN	1	0.5
WD40 RRM	1	1
*WD40 TPR	1	1
