# Supplementary material for: Diversity and Variability of NOD-Like Receptors in Fungi
Source: Genome Biol Evol. 2014 Dec 8;6(12):3137–58. doi: 10.1093/gbe/evu251 (PMC4986451; doi:10.1093/gbe/evu251)
Supplement: Supplementary Data [file supp_evu251_supplementary_tables.pdf]

Table S1. Non-canonical P-loops in NLR candidates

Pattern	in NACHT aligned	in NB-ARC aligned
AXXXXGK[ST]	281	12
CXXXXGK[ST]	2	14
DXXXXGK[ST]	156	2
EXXXXGK[ST]	4	3
FXXXXGK[ST]	0	0
IXXXXGK[ST]	0	0
LXXXXGK[ST]	1	0
MXXXXGK[ST]	0	0
RXXXXGK[ST]	2	0
SXXXXGK[ST]	11	12
VXXXXGK[ST]	2	0
WXXXXGK[ST]	0	0
GXXXXAK[ST]	2	0
GXXXXCK[ST]	3	0
GXXXXDK[ST]	2	0
GXXXXEK[ST]	0	3
GXXXXRK[ST]	5	2
GXXXXSK[ST]	1	1
GXXXXGA[ST]	0	0
GXXXXGE[ST]	2	1
GXXXXGM[ST]	2	0
GXXXXGR[ST]	12	0
GXXXXGT[ST]	3	0
GXXXXGKA	3	1
GXXXXGKF	2	0
GXXXXGKG	2	0
GXXXXGKI	2	0
GXXXXGKK	1	1
GXXXXGKL	10	0
GXXXXGKM	1	1
GXXXXGKP	2	1
GXXXXGKQ	0	0

Clan	Phylum	Total	HiC100	HiC100/Total	HiC100 NR99
TPR	Dikarya	17017	162	0.95%	156
	Metazoa	27062	254	0.94%	229
	Viridiplantae	18981	50	0.26%	45
WD	Dikarya	18826	206	1.09%	171
	Metazoa	39370	102	0.26%	94
	Viridiplantae	9989	33	0.33%	30
ANK	Dikarya	6221	144	2.31%	137
	Metazoa	15055	282	1.87%	259
	Viridiplantae	3533	26	0.74%	26

Signature	#paralogs	#HiC	#HiC/#paralogs	P-value
HET	75	46	61.1%	1.0E-17
PFD-LIKE	25	11	43.7%	0.0011
RELA_SPOT	10	3	30.0%	n.s
PATATIN	26	6	22.8%	n.s
PNP_UDP	297	65	21.9%	0.0172
HELO-LIKE	238	39	16.5%	n.s
SESB-LIKE	223	25	11.1%	n.s
GOODBYE-LIKE	316	26	8.1%	8.8E-05
HELO	22	1	4.6%	n.s
C2	7	0	0.0%	n.s
PEPTIDASE_S8	6	0	0.0%	n.s
NB-ARC	343	75	21.9%	0.0136
NACHT	1466	244	16.6%	n.s
TOTAL	2378	387	16.3%	-

N-term	#orthologs	#paralogs*	#para/#ortho
PNP_UDP	50	380.4	7.61
GOODBYE-LIKE	88	662.3	7.53
HELO-LIKE	73	452.5	6.20
SESB-LIKE	76	451.3	5.94
HET	21	100.8	4.80
RELA_SPOT	5	15.7	3.13
C2	3	9.0	3.00
PFD-LIKE	17	35.5	2.09
PATATIN	31	52.2	1.68
HELO	26	39.3	1.51
PEPTIDASE_S8	6	6.0	1.00
PKINASE	4	3.3	0.83

* Number of paralogs was averaged over the number of strains available for a species, e.g. PKINASE was found in N-termini of 1 out of 3 strains of *F. oxysporium*, in addition to 3 other species
