# Supplementary material for: Diversity and Variability of NOD-Like Receptors in Fungi
Source: Genome Biol Evol. 2014 Dec 8;6(12):3137–58. doi: 10.1093/gbe/evu251 (PMC4986451; doi:10.1093/gbe/evu251)
Supplement: Supplementary Data [file supp_evu251_New_Microsoft_Office_Word_Document.docx]

**Figure S1. Venn diagram of inclusion and overlap between the hits obtained with the different query sets**

Query designations are IR (incompatibility related, in red), FV (functionally validated, in blue) and PD (phylogenetically diverse, in green). The distribution of hits in the different query sets is shown for the total set or for the hits picked up by a NACHT or NB-ARC query. For each ensemble, the number of hits is shown.

**Figure S2. Domain annotation of the fungal NLR set in ascomycetes and basidiomycetes**

Pie-charts showing the distribution of domain annotation in the N-terminal, NOD and C-terminal domains (respectively on the left, center and right). Results for ascomycetes are on the top and for basidiomycetes on the bottom. In each pie chart, the light grey corresponds to the fraction of domains with no annotation.

**Figure S3. Diagram of domain associations between NOD and C-terminal repeat domains in fungal NLRs**

For each of the 4 annotation classes of the NOD domain, the types of C-terminal domain that are found associated with it are shown. For the C-terminal domains, the size of the disk is proportional to the abundance of the annotation. “REST” denotes unknown (non-annotated) domains and other annotations (distinct from WD, TPR, ANK). Color coding is red, green and yellow for ANK, TPR and WD respectively.

**Figure S4. Phylogenetic trees for HET and NACHT domains for NLR candidates with a HET-NACHT domain architecture**

The entire set of NLR candidates with a HET-NACHT architecture was analyzed and phylogenetic trees were constructed separately for the HET and NACHT domains and sequences of origin connected by a line. Note that the trees of the NOD and HET domains are not congruent. For each sequence, the gi number and the full domain architecture are given.

**Figure S5. Phylogenetic trees of the NLRs of *Bipolaris maydis***

The tree was constructed from the NOD sequence for the entire set of NLR candidates from *Bipolaris maydis*. For each sequence, the gi number and the full domain architecture are given. Note that the different N-terminal domains do not group as a monophyletic group, but rather are generally scattered in different branches.

**Figure S6. 15 Phylogenetic trees constructed with the NLR candidates from multi-strain species**

For each of the 15 fungal species of our species set, for which at least two different strains were sequenced, phylogenetic trees of the NLR candidates based on the NOD domain were constructed. In each tree, the strain of origin is given (together with a different colour coding for each strain) as well as the domain architecture and gi number of each NLT candidate. Species designation is as follows: AGABI, *Agaricus bisporus*; AJECA, *Ajellomyces capsulatus*; AJEDE, *Ajellomyces dermatitidis;* ASPFU, *Aspergillus fumigatus*; ASPNI, *Aspergillus niger*; BIPMA, *Bipolaris maydis*; BOTFU, *Botryotinia fuckeliana;* COCPO, *cocciodioides posadasii*; FUSOX, *Fusarium oxysprorum*; MAGOR, *Magnaporthe oryzae*; NEUTE, *Neurospora tetrasperma*; PARBR, *Paracoccidioides brasiliensis*; PENDI, *Penicillium digitatum*; RHISO, *Rhizoctonia solani*; SERLA, *Serpula lacrymans*.

**Figure S7. Hypervariable sites in TPR and ANK repeats of two selected fungal NLRs**

**A.** An alignment of individual TPR motifs of gi255934897 from *Penicillium chrysogenum* (AAA/TPR) is shown. Highly variable positions (with 4 or more amino acids found at that position) are marked with a pink dot, other highly variable positions are marked with a yellow dot. The TPR domain of gi255934897 was modelled using the human kinesin light chain 2 structure as (PDB ID 3EDT) as the template. Colour coding of the variable sites is as above.

**B.** An alignment of individual ANK repeats of gi116208038 from *Chaetomium globosum* (PNP_UDP/NACHT/ANK) is shown. Highly variable positions (with 4 or more amino acids found at that position) are marked with a pink dot, other highly variable positions are marked with a yellow dot. The ANK repeat domain was modelled using the structure of artificial ANK repeat domain of the engineered protein OR264 (PDB ID 4GPM) as template. Colour coding of the variable sites is as above.
